# Multiconfiguration Pair-Density Functional Theory for Transition Metal Silicide Bond Dissociation Energies, Bond Lengths, and State Orderings

**DOI:** 10.3390/molecules26102881

**Published:** 2021-05-13

**Authors:** Meagan S. Oakley, Laura Gagliardi, Donald G. Truhlar

**Affiliations:** 1 Chemical Theory Center, Department of Chemistry, Minnesota Supercomputing Institute, University of Minnesota, Minneapolis, MN 55455, USA; moakley@umn.edu; 2 Chicago Center for Theoretical Chemistry, Department of Chemistry, James Franck Institute, Pritzker School of Molecular Engineering, The University of Chicago, Chicago, IL 60637, USA

**Keywords:** complete-active space self-consistent field, Kohn-Sham density functional theory, perturbation theory, potential energy surfaces, transition metals

## Abstract

Transition metal silicides are promising materials for improved electronic devices, and this motivates achieving a better understanding of transition metal bonds to silicon. Here we model the ground and excited state bond dissociations of VSi, NbSi, and TaSi using a complete active space (CAS) wave function and a separated-pair (SP) wave function combined with two post-self-consistent field techniques: complete active space with perturbation theory at second order and multiconfiguration pair-density functional theory. The SP approximation is a multiconfiguration self-consistent field method with a selection of configurations based on generalized valence bond theory without the perfect pairing approximation. For both CAS and SP, the active-space composition corresponds to the nominal correlated-participating-orbital scheme. The ground state and low-lying excited states are explored to predict the state ordering for each molecule, and potential energy curves are calculated for the ground state to compare to experiment. The experimental bond dissociation energies of the three diatomic molecules are predicted with eight on-top pair-density functionals with a typical error of 0.2 eV for a CAS wave function and a typical error of 0.3 eV for the SP approximation. We also provide a survey of the accuracy achieved by the SP and extended separated-pair approximations for a broader set of 25 transition metal–ligand bond dissociation energies.

## 1. Introduction

Transition metal silicides are useful in silicon-based devices due to their high thermal stability, low electric resistivity, and low density [1,2]. As electronics become smaller, the metal-silicon bond becomes increasingly important [3]. Studies involving transition-metal-encapsulating Si clusters show that transition metals (specifically Zr, Nb, Mo, W) terminate the dangling bonds through selective interactions between the d and p orbitals [4], which suggests that focusing on the M-Si interaction could provide detailed understanding to tune properties of devices with such bonds. The nature of metal–silicide bonds has been explored experimentally by measuring bond dissociation energies (BDEs). Early measurements of BDEs for diatomic transition metal, lanthanide, and actinide silicides were done by Knudsen effusion mass spectrometry, which produces BDEs with errors in the range 0.1–0.9 eV [5]. To obtain more accurate BDEs, the Morse group has utilized a precise predissociation-based resonant two-photon ionization method [6,7,8,9] that has an accuracy of approximately 0.004 eV. This substantial increase in experimental accuracy produces benchmark values against which quantum chemistry methods can now be tested, and the accuracy attainable can be a significant indicator of whether the bonding is modelled adequately. This kind of test of theory is particularly interesting because prediction of the energetics of open-shell transition metal compounds is complicated by near-degeneracy effects that produce a plethora of low-lying electronic states [10,11] that can best be modelled with multiconfiguration quantum mechanical methods.

One way to realistically model the multiconfigurational character of transition metal complexes is by the complete active space self-consistent field method [12,13] (CASSCF). A complete active space wave function includes all electronic configurations that are allowed by distributing a specified number of electrons, called the active electrons, in a specified number of orbitals, called the active orbitals or the active space. The number of configuration state functions (CSFs) in a CASSCF wave function depends on the number of active orbitals, number of active electrons, and the spin, and it grows exponentially with the size of the active space; consequently, the CASSCF method becomes computationally costly, often prohibitively so, for large active space sizes. The generalized active space self-consistent field method [14] was introduced to reduce computational cost by partitioning the active space into subspaces. The partitioning may be done by various schemes, and the present study uses the scheme of the separated-pair (SP) approximation [15,16,17]. The SP approximation is like generalized valence bond theory [18] but without the perfect pairing approximation. Using a precisely formulated method of selecting the active space and its subspaces is important because it eliminates the human judgment issue, which one must remove in order to have a theoretical model chemistry [19] that can be systematically validated. We employ the SP and CASSCF schemes with the nominal correlated participating orbital active space [16,20,21], which is abbreviated nom-CPO, which provides a framework for systematic active space selection.

The CASSCF and SP methods both account for static electron correlation energy, but for affordable active spaces they obtain only a small portion of the dynamic correlation energy, which includes the core-valence correlation. One may approximate the missing correlation energy by using perturbation theory, and here we test this approach by using second order perturbation theory with the CASSCF wave function as the zero-order wave function, which yields the CASPT2 method [22,23,24]. We also study an inexpensive alternative to account for electron correlation energy, namely multiconfiguration pair-density functional theory (MC-PDFT) [25,26], which is here applied both with a CASSCF reference function (CAS-PDFT) and with an SP reference function (SP-PDFT) It has been previously shown that the MC-PDFT method has comparable accuracy to that of CASPT2 for dissociation energies, while requiring less computational resources, and it does not suffer from intruder state problems that often plague CASPT2 [15,26,27,28,29].

Some previous theoretical studies on the transition metal silicides studied here have been carried out with Kohn–Sham density functional theory [8,30,31] and coupled cluster theory [31], both of which are single-reference methods. A study [32] using CASSCF and CASPT2 showed that VSi has six low-lying electronic states (two doublets, two quartets, and two sextets) within 0.6 eV of the ground state, which makes the theoretical determination of the true ground state difficult [3]. The main objectives of the present study are to determine the true ground state of three group-5 silicides (VSi, NbSi, and TaSi) and to test the accuracy of computed bond dissociation energies for these compounds when computed with pair-density functional treatments based on CASSCF and SP wave functions (yielding CAS-PDFT and SP-PDFT) as compared to CASPT2. The accuracy is judged by comparison to the experimental data of Morse et al. [6]. Additionally, we calculate the bond lengths and state orderings for each molecule.

## 2. Computational Methods

The computations were carried out using CASSCF and SP wave functions. In the SP method [29], the singly-occupied orbitals and their correlating orbitals are placed in a GAS subspace, and each pair of orbitals consisting of a doubly-occupied active orbital and its correlating orbitals are placed in separate two-electron, two-orbital subspaces. In this scheme, all intra-subspace electron excitations are allowed, but inter-subspace electron excitations are not allowed.

Previous work showed that the nom-CPO active space [16,20,21] provides an accurate treatment of TiSi [29], and so that scheme is adopted here. The nom-CPO active space includes all bonding, antibonding, and unpaired electrons and orbitals. For VSi, NbSi, and TaSi this results in a (7,10) active space. The active space for each molecule studied here includes the 2σ and 2$\sigma^{*}$ orbitals, the singly occupied 3σ orbital and its corresponding antibonding orbital, two sets of π bonding and antibonding orbitals, and a set of δ bonding and antibonding orbitals; pictures of the natural orbitals, including their occupation numbers are shown in Figures S1–S3 in the supporting information. Since VSi, NbSi and TaSi are all isoelectronic, the electron configurations corresponding to specific states are the same. Table 1 gives the dominant electron configuration and number of configuration state functions of both the CASSCF and SP active spaces with respect to the states considered in this work. This (7,10) nom-CPO active space is built from four orbitals in the a${}_{1}$ symmetry: 2σ, 2$\sigma^{*}$, 3σ, 3$\sigma^{*}$, two orbitals in the b${}_{2}$ symmetry: 1$\pi_{x}$ and 1$\pi_{x}^{*}$, two orbitals in the a${}_{2}$ symmetry: 1$\delta_{x}$ and 1$\delta_{x}^{*}$, and two orbitals in the b${}_{1}$ symmetry: 1$\pi_{y}$ and 1$\pi_{y}^{*}$. Seven electrons are distributed in these orbitals based on the dominant configuration of the state in question, as shown in Table 1. The CASSCF calculations use this active space without partitioning it, and the SP approximation partitions the CAS into four or five subspaces, where only low-lying states were considered with this approximation (${}^{4}\Pi$, ${}^{6}\Sigma^{+}$, ${}^{2}\Delta$, ${}^{2}\Sigma^{+}$). Each of these states has a unique set of subspaces, and they are listed in Table 2, along with the number of CSFs for each CASSCF and SP wave function used in this work. The large reduction in the amount of CSFs is appealing for computing properties of molecules that require prohibitively large CASSCF active spaces.

Potential energy curves were calculated for all three molecules with the *OpenMolcas* package [33] (version 18.09 tag 573-g0dff1fb) with CASSCF and SP reference wave functions and using CAS-PDFT, SP-PDFT, and CASPT2 with the ANO-RCC-VTZP [34,35] basis set. When calculating potential energy curves the natural orbitals from the previous geometry were used as an initial orbital guess to create a smooth curve. Scalar relativistic effects were included in all calculations with the second-order Douglas-Kroll-Hess Hamiltonian. For the CASPT2 computations, the ionization-potential–electron-affinity (IPEA) parameter was set to 0.25 a.u., and an imaginary shift of 0.25 a.u. was used to mitigate the presence of intruder states. Additionally, the following orbitals were frozen in the PT2 step: 1s and 2s for Si, up to 3s for V, up to 3d for Nb, and up to 5s for Ta. The CAS-PDFT and SP-PDFT potential energy curves were computed with a number of on-top pair-density functionals: tPBE, ftPBE, trevPBE, ftrevPBE, tBLYP, ftBLYP, tOreLYP, and ftOreLYP. [25,26,29] An in-house version of the *OpenMolcas* code was used for the tOreLYP and ftOreLYP on-top functionals. All MC-PDFT computations were done with an ultrafine grid size.

Heteronuclear diatomics have C${}_{\infty v}$ symmetry, but *OpenMolcas* is only capable of handling Abelian symmetries, so the potential energy curves of the low-lying states investigated here were computed in C${}_{2v}$ symmetry (shown in parentheses after the correct C${}_{\infty v}$ state symmetry): $\Sigma^{+}$ (A${}_{1}$), Φ/Π (B${}_{1}$ or B${}_{2}$), and Δ (A${}_{2}$). The C${}_{2v}$ symmetry designations do not distinguish between Φ and Π states, so they are both labeled here as Π.

The VIBROT program in *OpenMolcas* was used to numerically solve the rovibrational spectra using the Numerov-Cooley method [36] with the potential energy curves interpolated using cubic splines. In this way we obtain the equilibrium bond lengths, zero point energies, and BDEs for all states considered. The bond dissociation energies, D${}_{0}$, were computed as:

(1) $$D_{0}=D_{e}-\mathrm{ZPE}\left(\mathrm{AB}\right)+\mathrm{SOC}\left(A\right)+\mathrm{SOC}\left(B\right)-\mathrm{SOC}\left(\mathrm{AB}\right)$$

where D${}_{e}$ is calculated as the difference in spin-orbit-free potential energy between the molecule at the equilibrium bond distance (r${}_{e}$) and at the dissociation asymptote of the potential energy curve, SOC is the spin-orbit lowering (a negative number), and ZPE is the zero point energy. Taking ZPE and SOC into account allows us to directly compare to the experimental bond dissociation energies measured by Morse et al. [6]. The spin-orbit splittings for individual atoms, SOC(A) and SOC(B), are taken from experimental data [37] whereas the molecular spin-orbit splittings, SOC(AB), were computed as a difference between CASSCF energies and spin-orbit coupled restricted active space state-interaction [38] (RASSI-SO) energies at r${}_{e}$ where the RASSI-SO energies were computed using 2 roots each of ${}^{6}\Sigma^{+}$, ${}^{4}\Sigma^{+}$, ${}^{2}\Sigma^{+}$, ${}^{6}\Delta$, ${}^{4}\Delta$, ${}^{2}\Delta$, ${}^{6}\Pi$, ${}^{4}\Pi$, and ${}^{2}\Pi$ states. This approach yields SOC(AB) results for VSi that are similar to those for other VX diatomics [39]. The SOC(AB) value is zero for NbSi due to the Σ ground state symmetry. The experimental $D_{0}$, SOC(A), and SOC(B) values and the calculated SOC(AB) values are reported in Table 3.

## 3. Results and Discussion

The CASSCF dominant configurations for the ground state of each molecule are reported in Tables S1–S3. In the case of VSi and TaSi, the dominant configuration has a weight of 0.38 and three other configurations have weights between 0.20 and 0.10. For NbSi, the dominant configuration has a weight of only 0.14, with two other configurations with weights above 0.10. This shows that the ground state wave function of NbSi is more multiconfigurational in nature than VSi and TaSi.

### 3.1. State Ordering

Because the transition metal silicides have many low-lying electronic states, uncovering the true state ordering is a challenge for both experiment and computation. Here, we report the states of each molecule that lie within 1 eV of the ground state, as computed with CASSCF, CAS-tPBE, CAS-ftPBE, and CASPT2 for VSi, NbSi and TaSi. The excited states are computed as vertical excitation energies from the equilibrium geometry of the lowest-energy state.

**Figure 1:**
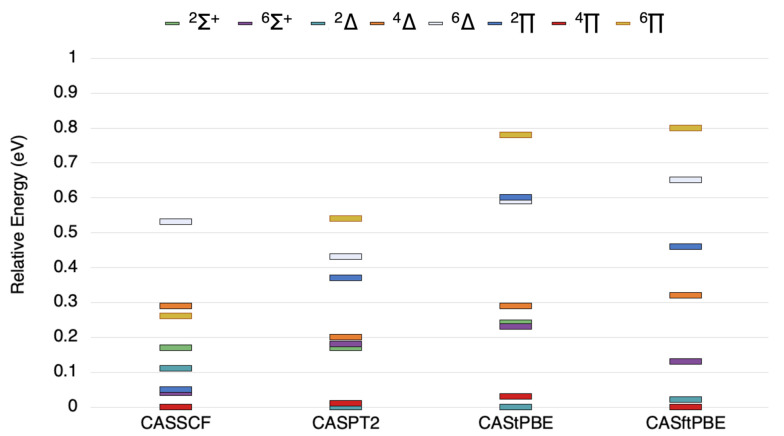
The state ordering (in eV) of the lowest ${}^{6}\Sigma^{+}$, ${}^{2}\Sigma^{+}$, ${}^{6}\Delta$, ${}^{4}\Delta$, ${}^{2}\Delta$, ${}^{6}\Pi$, ${}^{4}\Pi$, and ${}^{2}\Pi$ states of VSi as computed by the methods indicated.

Figure 1 shows the state ordering for VSi for all states within 1 eV of the ground state. CASPT2 and CAS-tPBE predict ${}^{2}\Delta$ as the ground state, and CASSCF and CAS-ftPBE predict ${}^{4}\Pi$ as the ground state. The CASSCF results place ${}^{4}\Pi$ and ${}^{2}\Delta$ within 0.1 eV of each other, whereas these states are much closer together (0.01–0.03 eV) in the CASPT2, CAS-tPBE, and CAS-ftPBE results. These small energy differences are within the typical error ranges of the methods used. CASSCF predicts the ${}^{2}\Delta$ state to be higher in energy than the ${}^{6}\Sigma^{+}$ state, whereas the post-SCF methods all place the ${}^{6}\Sigma^{+}$ state 0.2 eV higher in energy than the ground state. All post-SCF methods predict quasidegeneracy between ${}^{2}\Delta$ and ${}^{4}\Pi$ states, ${}^{6}\Sigma^{+}$ and ${}^{2}\Sigma^{+}$ states, and ${}^{6}\Delta$ and ${}^{2}\Pi$ states, and the relative energy with respect to the ground state varies by up to 0.1 eV.

**Figure 2:**
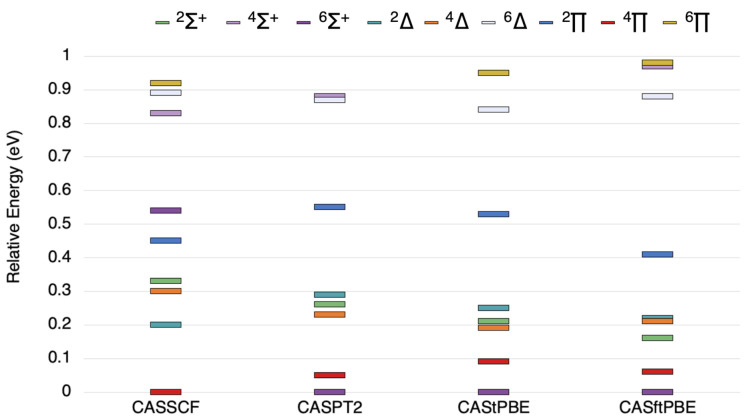
The state ordering of the lowest ${}^{6}\Sigma^{+}$, ${}^{4}\Sigma^{+}$, ${}^{2}\Sigma^{+}$, ${}^{6}\Delta$, ${}^{4}\Delta$, ${}^{2}\Delta$, ${}^{6}\Pi$, ${}^{4}\Pi$, and ${}^{2}\Pi$ states states of NbSi as computed by the methods outlined above.

The state ordering for NbSi is shown in Figure 2 for all states within 1 eV of the ground state. The CASSCF results give ${}^{4}\Pi$ as the ground state, with ${}^{2}\Delta$ and ${}^{4}\Delta$ states within 0.3 eV, and the ${}^{2}\Sigma^{+}$ is about 0.32 eV higher than the ground state. The ${}^{6}\Sigma^{+}$ state lies at 0.55 eV in the CASSCF results, but it is stabilized considerably in all post-SCF results such that these methods all report this state as the ground state. The CASPT2 results have the ${}^{6}\Sigma^{+}$ state as the ground state, and the ${}^{4}\Pi$ state lies 0.05 eV above it. The ${}^{2}\Sigma^{+}$, ${}^{4}\Delta$, and ${}^{2}\Delta$ states all lie between 0.2 and 0.3 eV for CASPT2. The CAS-tPBE and CAS-ftPBE results give the same state ordering, where the ${}^{6}\Sigma^{+}$ is the ground state, but the ${}^{4}\Pi$ state is close in energy at less than 0.1 eV above the ground state, and the ${}^{2}\Delta$, ${}^{4}\Delta$, and ${}^{2}\Sigma^{+}$ states are clustered around 0.15–0.25 eV.

**Figure 3:**
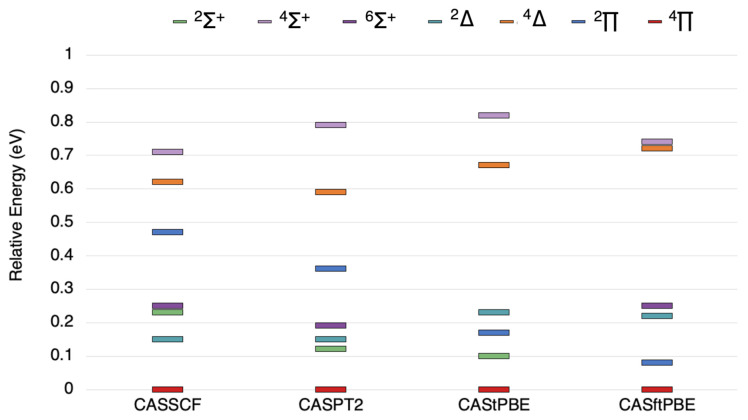
The state ordering of the lowest ${}^{6}\Sigma^{+}$, ${}^{4}\Sigma^{+}$, ${}^{2}\Sigma^{+}$, ${}^{4}\Delta$, ${}^{2}\Delta$, ${}^{4}\Pi$, and ${}^{2}\Pi$ states of TaSi as computed by the methods indicated.

The state ordering for TaSi is shown in Figure 3. The CASSCF results give ${}^{4}\Pi$ as the ground state for TaSi, with ${}^{2}\Delta$ and ${}^{6}\Sigma^{+}$ states about 0.2 eV and 0.25 eV higher in energy, respectively. The ground state is the same (${}^{4}\Pi$) for all methods in Figure 3; however the excited state ordering varies slightly for the post-SCF methods. In the case of CASPT2, the ${}^{2}\Sigma^{+}$ state is 0.11 eV above the ground state, and the ${}^{2}\Delta$ and ${}^{6}\Sigma^{+}$ states are higher at 0.15 eV and 0.19 eV, respectively. For CAS-tPBE, the ${}^{2}\Sigma^{+}$ state is the first excited state at 0.1 eV, the ${}^{2}\Pi$ state lies at 0.18 eV, and the ${}^{2}\Delta$ and ${}^{6}\Sigma^{+}$ are degenerate at 0.23 eV above the ground state. The results from CAS-ftPBE show the ${}^{2}\Sigma^{+}$ state is degenerate with the ${}^{2}\Pi$ state just below 0.1 eV, and the ${}^{2}\Delta$ state and ${}^{6}\Sigma^{+}$ states lie close together at 0.22 and 0.25 eV, respectively. The other states are all above 0.5 eV and the ordering is the same for all methods.

For TaSi, the ${}^{4}\Pi$ state is clearly predicted to be the ground state, but for VSi and NbSi, no definitive conclusion can be drawn at this stage. A post-SCF treatment gives quasidegenerate ${}^{4}\Pi$ and ${}^{2}\Delta$ states as potential ground states for VSi, and a ${}^{6}\Sigma^{+}$ ground state for NbSi (with a low-lying ${}^{4}\Pi$ state below 0.1 eV). These states will be used in the comparison to experimental bond dissociation energies in following sections.

### 3.2. Vanadium Silicide

Table 4, Table 5, Table 6 and Table 7 give the BDEs (D${}_{0}$), equilibrium bond lengths (r${}_{e}$), and zero point energies (in cm${}^{-1}$) for the ${}^{2}\Delta$ and ${}^{4}\Pi$ states of VSi. Both states are included in the discussion since they are almost degenerate. The computed $D_{0}$ are compared to the experimental VSi BDE of 2.234(2) eV; the difference between computed BDE and experiment is reported as $\Delta D_{0}$. The last rows of the tables give the mean unsigned error (MUE)in the MC-PDFT results, averaged over eight on-top functionals.

Table 4 shows the CASSCF results for the ${}^{4}\Pi$ state. The CASSCF BDE underestimates experiment by almost 1.5 eV, but both CASPT2 and CAS-PDFT provide a BDE that is closer to that of experiment. All pair-density functionals have an error less than 0.2 eV; CASPT2 underestimates $D_{0}$ by 0.04 eV, whereas CAS-tPBE overestimates it by 0.04 eV. The best pair density functionals in this case, CAS-ftPBE and CAS-tOreLYP, are both within 0.01 eV of experiment. Previous results computed with B3LYP [6] provide a BDE that overestimates the experimental value by 0.31 eV, which is almost double the error we get with some post-SCF methods. Additionally, the equilibrium bond distance is much longer than what is predicted by CASPT2 and CAS-PDFT methods in Table 4.

Similarly to Table 4, the SP-PDFT results are shown in Table 5 with the ΔD${}_{0}$ value computed with respect to the experimental value. Like CASSCF, SP also underestimates $D_{0}$, but the SP difference from experiment is smaller. The SP-tPBE are SP-ftPBE differ from experiment by less than 0.04 eV. This implies that the SP scheme for selecting SP gives similar results to the much larger CASSCF active space of the same electron and orbital composition at both the equilibrium region and at dissociation.

The results for the ${}^{2}\Delta$ state are similar to those for the ${}^{4}\Pi$ state and are reported in Table 6 for the CASSCF reference and Table 7 for the SP reference. The CASPT2, CAS-tPBE, CAS-ftPBE, CAS-trevPBE and CAS-ftrevPBE bond dissociation energies are all within 0.1 eV of the experiment. The SP-based results are similar with CAS-tPBE and CAS-ftPBE being only 0.06 eV and 0.01 eV below experiment.

**Figure 4:**
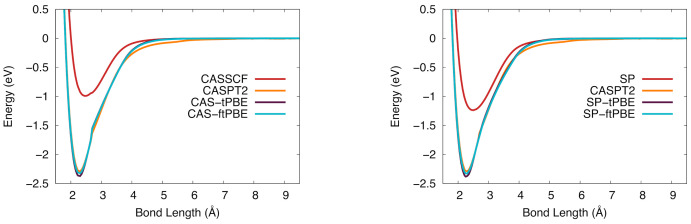
The potential energy curve for vanadium silicide in the ${}^{4}\Pi$ ground state. (**Left**): CASSCF (red), CASPT2 (orange), CAS-tPBE (purple), CAS-ftPBE (blue). (**Right**): Computed with SP (red), SP-tPBE (purple), SP-ftPBE (blue), and CASPT2 (orange).

The potential energy curves for the ${}^{4}\Pi$ state of VSi are shown in Figure 4. The CASSCF curve does not give a good description of the potential energy curve, as determined by the large $\Delta D_{0}$ value in Table 4. The description is improved when post-SCF treatments are used; both of the shown pair-density functionals give similarly shaped curves to the orange CASPT2 one. There is a small discontinuity in the CASSCF-based results near 2.7 Å but the SP-based results are smoother. Like CASSCF, the SP wave function does not describe this process well, but SP-PDFT corrects for the poor description and yields a result similar to that of CASPT2.

**Figure 5:**
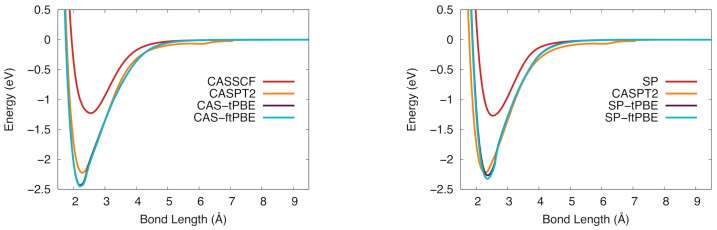
The potential energy curve for vanadium silicide in the ${}^{2}\Delta$ ground state. (**Left**): CASSCF (red), CASPT2 (orange), CAS-tPBE (purple), CAS-ftPBE (blue). (**Right**): SP (red), SP-tPBE (purple), SP-ftPBE (blue), CASPT2 (orange).

The potential energy curves for VSi in the ${}^{2}\Delta$ state are shown in Figure 5. The CAS-PDFT results give a deeper potential than CASPT2, generating a slightly larger D${}_{e}$ value. The SP-tPBE and SP-ftPBE calculations are much closer to the CASPT2 curve than are the CAS-PDFT results.

Even though the ${}^{2}\Delta$ state is reported as lowest energy state at equilibrium for most methods, the computed ${}^{4}\Pi$ bond dissociation energies match more closely with experiment on average.

### 3.3. Niobium Silicide

The results for NbSi are given in Table 8 and Table 9. Table 8 gives CASSCF results for the ${}^{4}\Pi$ state and post-CASSCF results for the ${}^{6}\Sigma^{+}$ state (these are the predictions of the ground state, as discussed above). The BDE computed with CASSCF underestimates experiment by 1 eV, but all post-SCF methods are much more accurate. The CASPT2 results are 0.23 eV above the experimental value of 3.080(3) eV. Among the CAS-PDFT results, the CAS-trevPBE and CAS-ftrevPBE functionals are the most accurate, only 0.09 eV and 0.06 eV above experiment, respectively. All other BDEs computed with CAS-PDFT are within 0.35 eV. The equilibrium bond distances vary within the CAS-PDFT framework by 0.04 Å. Compared to previous B3LYP results, the error with respect to the experimental BDE is at least halved by using CAS-PDFT, and the bond lengths predicted by post-CASSCF methods are longer than what was found with B3LYP.

Table 9 contains BDEs, equilibrium bond distances, and zero point energy computed with an SP reference wave function for the ground state of NbSi. The best functionals for computing the BDE for NbSi are tPBE and ftPBE, which underestimate the experimental BDE by 0.26 and 0.22 eV, respectively. The equilibrium bond distances are very similar to what is reported in Table 8 with CAS-PDFT; the results with different reference wave functions but the same on-top functional are within 0.02 Å for equilibrium bond lengths.

**Figure 6:**
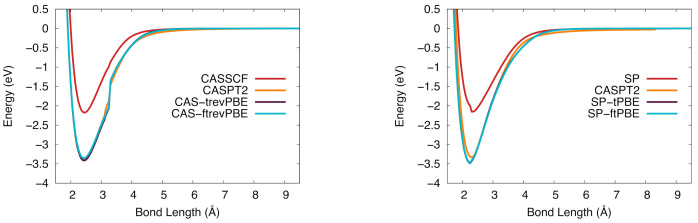
The potential energy curve for niobium silicide in the ${}^{4}\Pi$ ground state. (**Left**): CASSCF (red), CASPT2 (orange), CAS-tPBE (purple), CAS-ftPBE (blue). (**Right**): SP (red), SP-tPBE (purple), SP-ftPBE (blue), CASPT2 (orange).

The potential energy curves of NbSi in the ${}^{4}\Pi$ state are shown in Figure 6. The CASSCF-based results (left) show the CAS-PDFT potential energy curves track closely to CASPT2 at longer bond distances, however the CASPT2 curve is slightly shallower near equilibrium. These potential energy curves show a small discontinuity near 3.5 Å, and similarly in the SP-based potential energy curves (right), there is a small discontinuity near the equilibrium bond distance that is not present in the CASSCF or CASPT2 curve. This discontinuity is likely due to low-lying excited states present in the dense electronic excited state spectrum. The SP-PDFT curve is shifted to a lower equilibrium bond distance than CASPT2, which is also the case for CAS-PDFT.

**Figure 7:**
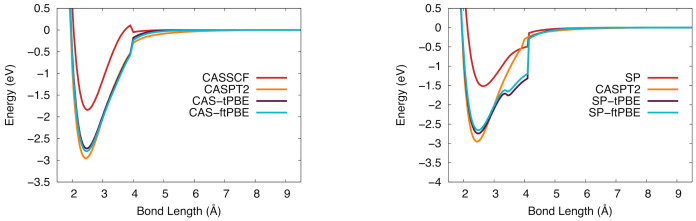
The potential energy curve for niobium silicide in the ${}^{6}\Sigma^{+}$ ground state. (**Left**): Computed with CASSCF (red), CASPT2 (orange), CAS-tPBE (purple), CAS-ftPBE (blue) with a (7,10) active space. (**Right**): Computed with SP (red), SP-tPBE (purple), SP-ftPBE (blue) with a GAS5(7,10) active space. CASPT2 (orange) is included for comparison.

The NbSi potential energy curves in the ${}^{6}\Sigma^{+}$ state are shown in Figure 7. These curves are not as smooth as the ones for the ${}^{4}\Pi$ state. The CASPT2 and CAS-PDFT curves all have a discontinuity near 4 Å. The SP-based results suffer from more severe discontinuities and would probably benefit from a state-averaged treatment in future work. Although the potential curves are not smooth, the BDEs computed with six of the eight CAS-PDFT methods and with SP-tPBE and SP-ftPBE are all within 0.26 eV of the experimental value.

### 3.4. Tantalum Silicide

The BDE, equilibrium bond length, and zero point energy for the ${}^{4}\Pi$ state of TaSi computed with a CASSCF reference wave function are reported in Table 10. CASPT2 yields a slightly shorter equilibrium bond length than any of the CAS-PDFT calculations, and the BDE is 0.15 eV below the experimental value. The BDE results closest to experiment are those computed with CAS-tPBE and CAS-ftPBE, with deviations from experiment of 0.07 eV and 0.10 eV; these methods are closer to experiment than CASPT2. Other CAS-PDFT functionals have higher deviations, with tBLYP and ftBLYP differing from the measured BDE by about a half eV.

Table 11 reports the SP-based results for the dissociation of TaSi. Like that of CASSCF, the SP bond dissociation energy is too small, but most of this error is removed by CAS-PDFT. This is especially the case with SP-tPBE and SP-ftPBE, where the error with respect to experiment is on the same order as CAS-tPBE and CAS-ftPBE (approximately 0.1 eV). In this case, the B3LYP equilibrium bond length is similar to those computed with CAS-PDFT and CASPT2, but the BDE error in B3LYP is larger.

**Figure 8:**
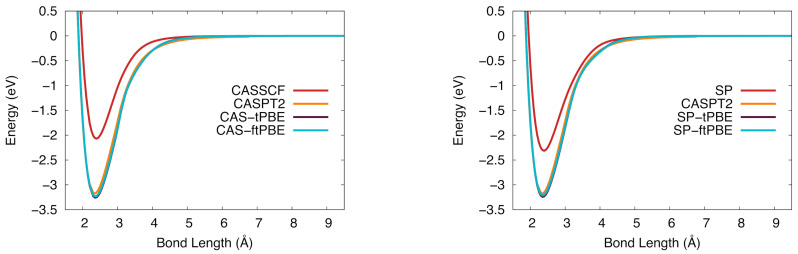
The potential energy curve for tantalum silicide in the ${}^{4}\Pi$ ground state. (**Left**): Computed with CASSCF (red), CASPT2 (orange), CAS-tPBE (purple), CAS-ftPBE (blue) with a (7,10) active space. (**Right**): Computed with SP (red), SP-tPBE (purple), SP-ftPBE (blue) with a GAS5(7,10) active space. CASPT2 (orange) is included for comparison.

Potential energy curves of TaSi computed with CASPT2, CASSCF, CAS-tPBE, and CAS-ftPBE are shown on the left side of Figure 8, and those computed with SP reference wave functions are on the right (with CASPT2 as a reference). Neither reference wave function gives an accurate potential energy curve, which is apparent in the large $\Delta D_{0}$ value. However, both CAS-PDFT and SP-PDFT with the tPBE and ftPBE on-top functionals give BDEs very close to that of CASPT2, although the equilibrium distance is slightly longer. This trend was also seen for VSi where CASPT2 predicts a slightly shorter r${}_{e}$ than CAS-PDFT or SP-tPBE by 0.02 Å.

Overall, the typical error involved in calculating the ground state BDE of VSi, NbSi, or TaSi with a nom-CPO active space with CAS-PDFT is 0.2 eV and with SP-PDFT is 0.3 eV.

### 3.5. Bond Dissociation Energies

Studying diatomic transition metal compounds provides an opportunity to affordably benchmark the SP approximation against CASSCF for active spaces of the same size, as well as to compare both methods to experiment. Here, we compare the accuracy of using CAS-PDFT and SP-PDFT on a set of 25 diatomic molecules containing one or two transition metal atoms. This includes the three molecules studied in the present paper (VSi, NbSi, TaSi) and 22 studied previously [16,17,29]. All 25 data points in this comparison are transition metal diatomic BDEs calculated with a nom-CPO active space [16,17,29]. For two of the molecules the SP method was not adequate, and in those cases we used the extended separated-pair (ESP) method [29]. In summarizing the accuracy for 25 molecules, we use the results for the ESP method when ESP was applied and the results for SP when ESP was not performed. Previous work with the SP approximation includes CrH, MnH, FeH, CoH, FeC, ScN, VN, CrN, TiO, FeO, NiO, V${}_{2}$, Cr${}_{2}$, CrF, NiC, FeS, NiS, FeSe, NiSe, and TiSi [16,17,29], and previous work involving the ESP approximation includes TiC and WCl [29].

The results of the survey of 25 diatomic transition metal compounds are shown in Table 12, and they are quite encouraging in several respects: (i) For a given on-top functional, the CAS and (E)SP results are very similar, with mean unsigned errors typically differing by only a few hundredths of an eV. (ii) The results for the various functionals are very similar to one another, again with mean unsigned errors typically differing by only a few hundredths of an eV. (iii) The mean unsigned errors averaged over 8 functionals, as given in the last column of the table, are only 0.27 eV for CAS-PDFT and only 0.28 eV for (E)SP. (iv) The fully translated functionals are more accurate than the translated ones, with a mean unsigned error averaged over 125 cases (five fully-translated functionals for 25 molecules) of only 0.26 eV for CAS-PDFT and only 0.27 eV for SP-PDFT. (v) The mean unsigned error, over all 25 cases for the best performing method, (E)SP-PDFT/ftOreLYP, is only 0.21 eV.

The good results obtained with the ftOreLYP functional are very satisfying since this functional is based on full translation [26] of the OptX exchange functional developed by Handy and Cohen [40] with the goal of including both nonlocal exchange and left-right correlation energy and the reLYP correlation functional developed by Thakkar and McCarthy [41] with the goal of making a functional more accurate for the correlation energies of atoms with atomic numbers 20–86 (the transition metals considered here have atomic numbers 23, 41, and 73).

## 4. Conclusions

In this study, we report calculations of the ground state state symmetry, bond dissociation energy, equilibrium internuclear distance, and zero point energy for three transition metal silicides. The SP-PDFT bond dissociation energies are very similar to CAS-PDFT results, showing that the separated-pair approximation is a good way to approximate a CASSCF wave function while reducing the number of configuration state functions. The MC-PDFT functionals tPBE and ftPBE give bond dissociation energies that are just as accurate, or better than, the CASPT2 ones while also keeping the computational cost low. Additionally, there is, on average, a twofold improvement over previous Kohn–Sham density functional results. As the transition metal becomes heavier, the MUE increases slightly, but overall, the CAS-PDFT results have a MUE of 0.2 eV and SP-PDFT results have a MUE of 0.3 eV compared to experimental bond dissociation energies.

Comparing calculated bond dissociation energies to experiment is insufficient to reliably determine which state is the ground state for the transition metal silicides studied here because there are low-lying excited states. In the case of VSi, where the ground state ${}^{2}\Delta$ state is nearly degenerate with the ${}^{4}\Pi$ state, the ordering is similar for all post-SCF methods (CASPT2, CAS-PDFT, and SP-PDFT). For NbSi, some methods yield ${}^{6}\Sigma^{+}$ as the ground state, and others yield ${}^{4}\Pi$. The state ordering for NbSi trend with post-SCF methods is: ${}^{6}\Sigma^{+}$<${}^{4}\Pi$<${}^{4}\Delta$≈${}^{2}\Delta$<${}^{2}\Pi$. All results for TaSi are in agreement that the ground state is ${}^{4}\Pi$, and the closest excited state is near 0.1 eV, but the ordering of the excited states varies with the method used.

We also combined the present results for three transition metal diatomics with previous results for 22 other transition metal diatomics to get a broader assessment of the accuracy attainable with MC-PDFT. The mean unsigned errors results are very similar for CAS-PDFT and SP-PDFT, and they are very similar for simply translated functionals and for fully translated ones; if we therefore combine all these into a single data set, it has 400 data, and the mean unsigned error in transition metal bond dissociation energies for this data set is only 0.27 eV. The best method, SP-PDFT with the ftOreLYP functional, has a mean unsigned error of only 0.21 eV.

## Figures and Tables

**Table 1:** The dominant configuration and the number of configuration state functions for the active spaces of each state.

		# CSFs	
**State**	**Dominant Configuration**	**CASSCF**	**SP**
${}^{2}\Sigma^{+}$	1$\sigma^{2}$2$\sigma^{2}$1$\pi_{x}^{2}$1$\pi_{y}^{1}$3$\sigma^{1}$1$\delta_{x}^{0}$1$\delta_{y}^{0}$	3480	152
${}^{4}\Sigma^{+}$	1$\sigma^{2}$2$\sigma^{2}$1$\pi_{x}^{1}$1$\pi_{y}^{1}$3$\sigma^{2}$1$\delta_{x}^{1}$1$\delta_{y}^{0}$	2280	-
${}^{6}\Sigma^{+}$	1$\sigma^{2}$2$\sigma^{2}$1$\pi_{x}^{1}$1$\pi_{y}^{1}$3$\sigma^{1}$1$\delta_{x}^{1}$1$\delta_{y}^{1}$	504	80
${}^{2}\Delta$	1$\sigma^{2}$2$\sigma^{2}$1$\pi_{x}^{2}$1$\pi_{y}^{2}$3$\sigma^{0}$1$\delta_{x}^{1}$1$\delta_{y}^{0}$	3460	336
${}^{4}\Delta$	1$\sigma^{2}$2$\sigma^{2}$1$\pi_{x}^{1}$1$\pi_{x}^{*1}$1$\pi_{y}^{2}$3$\sigma^{0}$1$\delta_{x}^{1}$1$\delta_{y}^{0}$	2320	-
${}^{6}\Delta$	1$\sigma^{2}$2$\sigma^{2}$1$\pi_{x}^{1}$1$\pi_{x}^{*1}$1$\pi_{y}^{1}$1$\pi_{y}^{*1}$3$\sigma^{0}$1$\delta_{x}^{1}$1$\delta_{y}^{0}$	492	-
${}^{2}\Pi$	1$\sigma^{2}$2$\sigma^{2}$1$\pi_{x}^{2}$1$\pi_{y}^{1}$3$\sigma^{1}$1$\delta_{x}^{1}$1$\delta_{y}^{0}$	3460	-
${}^{4}\Pi$	1$\sigma^{2}$2$\sigma^{2}$1$\pi_{x}^{2}$1$\pi_{y}^{1}$3$\sigma^{1}$1$\delta_{x}^{1}$1$\delta_{y}^{0}$	2320	272
${}^{6}\Pi$	1$\sigma^{2}$2$\sigma^{2}$1$\pi_{x}^{1}$1$\pi_{x}^{*1}$1$\pi_{y}^{2}$3$\sigma^{1}$1$\delta_{x}^{1}$1$\delta_{y}^{0}$	492	-

**Table 2:** The SP approximation subspaces listed by state.

State	GAS1	GAS2	GAS3	GAS4	GAS5
${}^{2}\Sigma^{+}$	(2e,2o)	(1e,2o)	(2e,2o)	(2e,2o)	
	2σ,2$\sigma^{*}$	3σ,3$\sigma^{*}$	1$\pi_{x}$,1$\pi_{x}^{*}$	1$\pi_{y}$,1$\pi_{y}^{*}$
${}^{6}\Sigma^{+}$	(2e,2o)	(1e,2o)	(1e,2o)	(2e,2o)	(1e,2o)
	2σ,2$\sigma^{*}$	3σ,3$\sigma^{*}$	1$\pi_{x}$,1$\pi_{x}^{*}$	1$\delta_{x}$,1$\delta_{y}$	1$\pi_{y}$,1$\pi_{y}^{*}$
${}^{2}\Delta$	(2e,2o)	(2e,2o)	(1e,2o)	(2e,2o)
	2σ,2$\sigma^{*}$	1$\pi_{x}$,1$\pi_{x}^{*}$	1$\delta_{x}$,1$\delta_{x}^{*}$	1$\pi_{y}$,1$\pi_{y}^{*}$
${}^{4}\Pi$	(2e,2o)	(1e,2o)	(1e,2o)	(1e,2o)	(2e,2o)
	2σ,2$\sigma^{*}$	3σ,3$\sigma^{*}$	1$\pi_{x}$,1$\pi_{x}^{*}$	1$\delta_{x}$,1$\delta_{x}^{*}$	1$\pi_{y}$,1$\pi_{y}^{*}$

**Table 3:** The experimental bond dissociation energy, D${}_{0}$, and the presently used values for spin-orbit contributions to the energies where A is V, Nb, or Ta, and B is Si. All entries are in eV, and the numbers in parentheses are the experimental uncertainties in the last digit.

Molecule (AB)	D${}_{0}$(exp.)	SOC(A)	SOC(B)	SOC(AB)
VSi	2.234(2)	−0.07	−0.019	−0.014
NbSi	3.080(3)	−0.13	−0.019	0
TaSi	2.999(3)	−0.70	−0.019	−0.421

**Table 4:** Dissociation energies (eV), equilibrium bond lengths (Å), and zero point energies (in cm${}^{-1}$) for VSi in the ${}^{4}\Pi$ molecular state, computed with a CASSCF wave function.

Method	r${}_{e}$	D${}_{e}$	D${}_{0}$	$\Delta D_{0}$	ZPE
Exp [6]	-	-	2.234(2)	-	-
B3LYP [6]	2.433	-	2.54	0.31	-
CASSCF	2.472	0.99	0.90	−1.33	116.6
CASPT2	2.284	2.29	2.19	−0.04	220.3
CAS-tBLYP	2.276	2.14	2.04	−0.19	236.5
CAS-ftBLYP	2.270	2.13	2.03	−0.20	233.3
CAS-tOreLYP	2.261	2.35	2.25	0.01	242.2
CAS-ftOReLYP	2.261	2.27	2.17	−0.07	234.1
CAS-tPBE	2.274	2.38	2.27	0.04	238.1
CAS-ftPBE	2.280	2.33	2.23	−0.01	231.7
CAS-trevPBE	2.265	2.52	2.43	0.20	234.9
CAS-ftrevPBE	2.266	2.51	2.40	0.17	225.2
MUE (CAS-PDFT)				0.12	

**Table 5:** Dissociation energies (eV), equilibrium bond lengths (Å), and zero point energies (in cm${}^{-1}$) for VSi in the ${}^{4}\Pi$ molecular state, computed with a SP wave function.

Method	R${}_{e}$	D${}_{e}$	D${}_{0}$	ΔD${}_{0}$	ZPE
Exp [6]	-	-	2.234(2)	-	-
SP	2.490	1.24	1.15	−1.08	115.0
SP-tBLYP	2.276	2.14	2.04	−0.19	234.9
SP-ftBLYP	2.265	2.07	1.97	−0.27	232.5
SP-tOreLYP	2.271	2.13	2.02	−0.21	235.7
SP-ftOReLYP	2.257	2.18	2.07	−0.16	234.1
SP-tPBE	2.270	2.38	2.28	0.04	237.3
SP-ftPBE	2.276	2.34	2.23	−0.01	230.9
SP-trevPBE	2.261	2.55	2.45	0.21	233.3
SP-ftrevPBE	2.262	2.50	2.40	0.17	224.4
MUE (SP-PDFT)				0.16	

**Table 6:** Dissociation energies (eV), equilibrium bond lengths (Å), and zero point energies (in cm${}^{-1}$) for VSi in the ${}^{2}\Delta$ molecular state, computed with a CASSCF wave function.

Method	r${}_{e}$	D${}_{e}$	D${}_{0}$	$\Delta D_{0}$	ZPE
Exp [6]	-	-	2.234(2)	-	-
CASSCF	2.545	1.14	1.05	−1.19	126.4
CASPT2	2.233	2.26	2.16	−0.07	181.4
CAS-tBLYP	2.241	1.95	1.85	−0.38	195.2
CAS-ftBLYP	2.231	2.03	1.93	−0.31	200.9
CAS-tOreLYP	2.189	2.22	2.11	−0.12	217.1
CAS-ftOReLYP	2.200	2.22	2.12	−0.12	204.1
CAS-tPBE	2.219	2.42	2.32	0.08	202.5
CAS-ftPBE	2.225	2.44	2.34	0.11	196.8
CAS-trevPBE	2.222	2.26	2.16	−0.08	259.2
CAS-ftrevPBE	2.242	2.39	2.20	−0.04	186.3
MUE (CAS-PDFT)				0.16	

**Table 7:** Dissociation energies (eV), equilibrium bond lengths (Å), and zero point energies (in cm${}^{-1}$) for VSi in the ${}^{2}\Delta$ molecular state, computed with a SP wave function.

Method	R${}_{e}$	D${}_{e}$	D${}_{0}$	ΔD${}_{0}$	ZPE
Exp [6]	-	-	2.234(2)	-	-
SP	2.531	1.27	1.18	−1.05	138.5
SP-tBLYP	2.381	1.87	1.77	−0.47	196.8
SP-ftBLYP	2.362	1.94	1.84	−0.39	202.5
SP-tOreLYP	2.365	2.02	1.92	−0.31	200.1
SP-ftOReLYP	2.350	2.08	1.98	−0.25	204.1
SP-tPBE	2.370	2.27	2.17	−0.06	200.9
SP-ftPBE	2.359	2.32	2.22	−0.01	203.3
SP-trevPBE	2.337	2.12	2.03	−0.21	283.5
SP-ftrevPBE	2.373	2.18	2.08	−0.15	197.6
MUE (SP-PDFT)				0.23	

**Table 8:** Dissociation energies (eV), equilibrium bond lengths (Å), and zero point energies (in cm${}^{-1}$) for NbSi in the ground state, computed with a CASSCF wave function.

Method	State	R${}_{e}$	D${}_{e}$	D${}_{0}$	ΔD${}_{0}$	ZPE
Exp [6]	-	-	-	3.080(3)	-	-
B3LYP [6]	${}^{6}\Sigma^{+}$	2.496	-	2.42	−0.66	-
CASSCF	${}^{4}\Pi$	2.449	2.18	2.00	−1.08	190.4
CASPT2	${}^{6}\Sigma^{+}$	2.502	3.48	3.31	0.23	191.2
CAS-tBLYP	${}^{6}\Sigma^{+}$	2.548	3.03	2.86	−0.22	172.5
CAS-ftBLYP	${}^{6}\Sigma^{+}$	2.542	2.91	2.73	−0.35	181.4
CAS-tOreLYP	${}^{6}\Sigma^{+}$	2.521	3.18	3.01	−0.07	179.0
CAS-ftOReLYP	${}^{6}\Sigma^{+}$	2.513	3.16	2.98	−0.10	179.8
CAS-tPBE	${}^{6}\Sigma^{+}$	2.522	3.52	3.35	0.27	181.4
CAS-ftPBE	${}^{6}\Sigma^{+}$	2.517	3.45	3.28	0.20	181.4
CAS-trevPBE	${}^{6}\Sigma^{+}$	2.531	3.34	3.17	0.09	179.0
CAS-ftrevPBE	${}^{6}\Sigma^{+}$	2.525	3.31	3.14	0.06	179.8
MUE (CAS-PDFT)					0.17	

**Table 9:** Dissociation energies (eV), equilibrium bond lengths (Å), and zero point energies (in cm${}^{-1}$) for NbSi in the ground state, computed with a SP wave function.

Method	State	R${}_{e}$	D${}_{e}$	D${}_{0}$	ΔD${}_{0}$	ZPE
Exp [6]	-	-	3.080(3)	-	-	-
SP	${}^{4}\Pi$	2.455	2.42	2.24	−0.84	190.4
SP-tBLYP	${}^{6}\Sigma^{+}$	2.502	2.57	2.40	−0.68	173.3
SP-ftBLYP	${}^{6}\Sigma^{+}$	2.488	2.64	2.47	−0.61	173.3
SP-tOreLYP	${}^{6}\Sigma^{+}$	2.475	2.83	2.66	−0.42	178.2
SP-ftOReLYP	${}^{6}\Sigma^{+}$	2.465	2.86	2.69	−0.39	179.8
SP-tPBE	${}^{6}\Sigma^{+}$	2.481	3.00	2.83	−0.26	181.4
SP-ftPBE	${}^{6}\Sigma^{+}$	2.471	3.03	2.86	−0.22	181.4
SP-trevPBE	${}^{6}\Sigma^{+}$	2.492	2.85	2.68	−0.40	179.0
SP-ftrevPBE	${}^{6}\Sigma^{+}$	2.482	2.88	2.71	−0.37	179.8
MUE (SP-PDFT)					0.42	

**Table 10:** Dissociation energies (eV), equilibrium bond lengths (Å), and zero point energies (in cm${}^{-1}$) for TaSi in the ${}^{4}\Pi$ molecular state, computed with a CASSCF wave function.

Method	R${}_{e}$	D${}_{e}$	D${}_{0}$	ΔD${}_{0}$	ZPE
Exp [6]	-	-	-	2.999(3)	-
B3LYP [6]	2.375	-	2.68	−0.39	
CASSCF	2.391	2.07	1.75	−1.25	191.2
CASPT2	2.344	3.17	2.85	−0.15	205.7
CAS-tBLYP	2.384	2.80	2.48	−0.52	192.0
CAS-ftBLYP	2.377	2.85	2.53	−0.47	195.2
CAS-tOreLYP	2.357	3.08	2.75	−0.25	200.9
CAS-ftOReLYP	2.356	3.03	2.71	−0.29	200.1
CAS-tPBE	2.364	3.25	2.93	−0.07	200.0
CAS-ftPBE	2.362	3.23	2.90	−0.10	200.1
CAS-trevPBE	2.371	3.09	2.77	−0.23	197.6
CAS-ftrevPBE	2.370	3.05	2.72	−0.28	196.8
MUE (CAS-PDFT)				0.27	

**Table 11:** Dissociation energies (eV), equilibrium bond lengths (Å), and zero point energies (in cm${}^{-1}$) for TaSi in the ${}^{4}\Pi$ molecular state, computed with a SP wave function.

Method	R${}_{e}$	D${}_{e}$	D${}_{0}$	ΔD${}_{0}$	ZPE
Exp [6]	-	-	-	2.999(3)	-
SP	2.392	2.31	1.99	−1.01	191.2
SP-tBLYP	2.381	2.76	2.44	−0.56	191.2
SP-ftBLYP	2.373	2.78	2.46	−0.54	194.4
SP-tOreLYP	2.354	2.99	2.66	−0.34	200.9
SP-ftOReLYP	2.353	2.95	2.67	−0.37	200.9
SP-tPBE	2.360	3.24	2.92	−0.08	200.1
SP-ftPBE	2.358	3.21	2.89	−0.11	200.1
SP-trevPBE	2.368	3.07	2.75	−0.25	196.8
SP-ftrevPBE	2.367	3.03	2.71	−0.29	196.8
MUE (SP-PDFT)				0.32	

**Table 12:** The mean unsigned errors (eV) of ground state bond dissociation energies of transition metal diatomics as computed with CAS-PDFT and SP-PDFT/ESP-PDFT with various on-top pair-density functionals.

	tPBE	trevPBE	tBLYP	tOreLYP	ftPBE	ftrevPBE	ftBLYP	ftOreLYP	Overall
CAS	0.30	0.25	0.30	0.26	0.28	0.23	0.28	0.26	0.27
SP/ESP	0.31	0.28	0.35	0.22	0.29	0.28	0.28	0.21	0.28

## Data Availability

Absolute energies for VSi, NbSi, and TaSi potential energy curves. Active space natural orbitals and ground state configurations for each molecule.

## Supplementary Materials

The supplementary materials are available online.

## References

[1] Reader A., Van Ommen A., Weijs P., Wolters R., Oostra D. (1993). Transition metal silicides in silicon technology. Rep. Prog. Phys..

[2] Zhang S.L., Östling M. (2003). Metal silicides in CMOS technology: Past, present, and future trends. Crit. Rev. Solid State Mater. Sci..

[3] Gunaratne K.D.D., Berkdemir C., Harmon C., Castleman A. (2013). Probing the valence orbitals of transition metal–silicon diatomic anions: ZrSi, NbSi, MoSi, PdSi and WSi. Phys. Chem. Chem. Phys..

[4] Uchida N., Miyazaki T., Matsushita Y., Sameshima K., Kanayama T. (2011). New semiconducting silicides assembled from transition-metal-encapsulating Si clusters. Thin Solid Films.

[5] Gingerich K.A. (1970). Mass-spectrometric determination of the bond energies of the molecules AuS, BS, and BS_2_. J. Chem. Soc. D.

[6] Sevy A., Sorensen J.J., Persinger T.D., Franchina J.A., Johnson E.L., Morse M.D. (2017). Bond dissociation energies of TiSi, ZrSi, HfSi, VSi, NbSi, and TaSi. J. Chem. Phys..

[7] Morse M.D. (2018). Predissociation measurements of bond dissociation energies. Acc. Chem. Res..

[8] Sevy A., Tieu E., Morse M.D. (2018). Bond dissociation energies of FeSi, RuSi, OsSi, CoSi, RhSi, IrSi, NiSi, and PtSi. J. Chem. Phys..

[9] Sevy A., Merriles D.M., Wentz R.S., Morse M.D. (2019). Bond dissociation energies of ScSi, YSi, LaSi, ScC, YC, LaC, CoC, and YCH. J. Chem. Phys..

[10] Barysz M. (2016). Potential Energy Curves in the CASSCF/CASPT2 and FS-MR-CC Methods: The Role of Relativistic Effects. J. Chem. Theory Comput..

[11] Barysz M., Černušák I., Kellö V. (2019). Relativistic calculations of AuSi^+^ and AuSi^−^. Int. J. Quantum Chem..

[12] Roos B.O., Taylor P.R., Siegbahn P.E.M. (1980). A complete active space SCF method (CASSCF) using a density matrix formulated super-CI approach. Chem. Phys..

[13] Roos B.O. (1987). The complete active space self-consistent field method and its applications in electronic structure calculations. Adv. Chem. Phys..

[14] Ma D., Li Manni G., Gagliardi L. (2011). The generalized active space concept in multiconfigurational self-consistent field methods. J. Chem. Phys..

[15] Odoh S.O., Manni G.L., Carlson R.K., Truhlar D.G., Gagliardi L. (2016). Separated-pair approximation and separated-pair pair-density functional theory. Chem. Sci..

[16] Bao J.L., Odoh S.O., Gagliardi L., Truhlar D.G. (2017). Predicting bond dissociation energies of transition-metal compounds by multiconfiguration pair-density functional theory and second-order perturbation theory based on correlated participating orbitals and separated pairs. J. Chem. Theory Comput..

[17] Sharkas K., Gagliardi L., Truhlar D.G. (2017). Multiconfiguration pair-density functional theory and complete active space second order perturbation theory. bond dissociation energies of FeC, NiC, FeS, NiS, FeSe, and NiSe. J. Phys. Chem. A.

[18] Dunning T.H., Xu L.T., Cooper D.L., Karadakov P.B. (2021). Spin-coupled generalized valence bond theory: New perspectives on the electronic structure of molecules and chemical bonds. J. Phys. Chem. A.

[19] Pople J.A. (1999). Nobel Lecture: Quantum chemical models. Rev. Mod. Phys..

[20] Tishchenko O., Zheng J., Truhlar D.G. (2008). Multireference model chemistries for thermochemical kinetics. J. Chem. Theory Comput..

[21] Bao J.L., Sand A., Gagliardi L., Truhlar D.G. (2016). Correlated-participating-orbitals pair-density functional method and application to multiplet energy splittings of main-group divalent radicals. J. Chem. Theory Comput..

[22] Andersson K., Malmqvist P.A., Roos B.O., Sadlej A.J., Wolinski K. (1990). Second-order perturbation theory with a CASSCF reference function. J. Phys. Chem..

[23] Andersson K., Malmqvist P.Å., Roos B.O. (1992). Second-order perturbation theory with a complete active space self-consistent field reference function. J. Chem. Phys..

[24] Andersson K., Roos B.O. (1993). Multiconfigurational second-order perturbation theory: A test of geometries and binding energies. Int. J. Quantum Chem..

[25] Li Manni G., Carlson R.K., Luo S., Ma D., Olsen J., Truhlar D.G., Gagliardi L. (2014). Multiconfiguration pair-density functional theory. J. Chem. Theory Comput..

[26] Carlson R.K., Truhlar D.G., Gagliardi L. (2015). Multiconfiguration pair-density functional theory: A fully translated gradient approximation and its performance for transition metal dimers and the spectroscopy of Re_2_Cl_8_^2−^. J. Chem. Theory Comput..

[27] Ghosh S., Sonnenberger A.L., Hoyer C.E., Truhlar D.G., Gagliardi L. (2015). Multiconfiguration pair-density functional theory outperforms Kohn–Sham density functional theory and multireference perturbation theory for ground-state and excited-state charge transfer. J. Chem. Theory Comput..

[28] Oakley M.S., Bao J.J., Klobukowski M., Truhlar D.G., Gagliardi L. (2018). Multireference methods for calculating the dissociation enthalpy of tetrahedral P_4_ to two P_2_. J. Phys. Chem. A.

[29] Li S.J., Gagliardi L., Truhlar D.G. (2020). Extended separated-pair approximation for transition metal potential energy curves. J. Chem. Phys..

[30] Guo P., Ren Z.Y., Wang F., Bian J., Han J.G., Wang G.H. (2004). Structural and electronic properties of TaSi(n) (n = 1–13) clusters: A relativistic density functional investigation. J. Chem. Phys..

[31] Wu Z., Su Z. (2006). Electronic structures and chemical bonding in transition metal monosilicides M Si (M = 3d, 4d, 5d elements). J. Chem. Phys..

[32] Thao N.M., Hanh N.T.H., Tuan T., Tri T.Q., Van Tan T. (2018). An investigation on the electronic structures of diatomic VSi^0/−/+^ clusters by CASSCF/CASPT2 method. Vietnam J. Chem..

[33] Aquilante F., Autschbach J., Baiardi A., Battaglia S., Borin V.A., Chibotaru L.F., Conti I., De Vico L., Delcey M., Galván F.I. (2020). Modern quantum chemistry with [Open] Molcas. J. Chem. Phys..

[34] Roos B.O., Lindh R., Malmqvist P.Å., Veryazov V., Widmark P.O. (2004). Main group atoms and dimers studied with a new relativistic ANO basis set. J. Phys. Chem. A.

[35] Roos B.O., Lindh R., Malmqvist P.Å., Veryazov V., Widmark P.O. (2005). New relativistic ANO basis sets for transition metal atoms. J. Phys. Chem. A.

[36] Dykstra C.E., Malik D.J. (1987). Derivative Numerov–Cooley theory. A method for finding vibrational state properties of diatomic molecules. J. Chem. Phys..

[37] Siderius D. (2012). NIST Standard Reference Simulation Website.

[38] Malmqvist P.Å., Roos B.O., Schimmelpfennig B. (2002). The restricted active space (RAS) state interaction approach with spin–orbit coupling. Chem. Phys. Lett..

[39] Bao J.L., Welch B.K., Ulusoy I.S., Zhang X., Xu X., Wilson A.K., Truhlar D.G. (2020). Predicting Bond Dissociation Energies and Bond Lengths of Coordinatively Unsaturated Vanadium–Ligand Bonds. J. Phys. Chem. A.

[40] Handy N., Cohen A. (2000). Left-right correlation energy. Mol. Phys..

[41] Thakkar A., McCarthy S. (2009). Toward improved density functionals for the correlation energy. J. Chem. Phys..

