# Supplementary material for: Multiconfiguration Pair-Density Functional Theory for Transition Metal Silicide Bond Dissociation Energies, Bond Lengths, and State Orderings
Source: Molecules. 2021 May 13;26(10):2881. doi: 10.3390/molecules26102881 (PMC8152470; doi:10.3390/molecules26102881)
Supplement: Supplementary file 1 [file molecules-26-02881-s001.zip › molecules-1212239-supplementary.pdf]

Supporting Information: Multiconfiguration pair-density functional theory for the calculation of transition metal silicide bond dissociation energies, bond lengths, and state orderings

Meagan S. Oakley,[*,†] Donald G. Truhlar,[†] and Laura Gagliardi[‡]

†*Department of Chemistry, Chemical Theory Center, and Minnesota Supercomputing Institute, University of Minnesota, Minneapolis, MN, 55455, United States*

‡*Department of Chemistry, Pritzker School of Molecular Engineering, James Franck Institute, Chicago Center for Theoretical Chemistry, The University of Chicago, Chicago, IL, 60637, United States*

E-mail: moakley@umn.edu

Example Input Files for CAS-PDFT and SP-PDFT in OpenMolcas

CAS-PDFT:

```
>>export DIST=0.95
>>foreach L in ( 1 ..  250 )
>>eval DIST=$DIST+0.05
&GATEWAY
Coord
2
VSi molecule
V 0 0 0
Si $DIST 0 0
BASIS= ANO-RCC-VTZP
GROUP=Full
&SEWARD
grid input
grid=ultrafine
end of grid input
&RASSCF
LumOrb
SYMMETRY=4
SPIN=4
nActEl = 7 0 0
INACTIVE = 9 3 0 3
RAS2 = 4 2 2 2
>>COPY $Project.RasOrb INPORB
```

```
&MCPDFT
KSDFT=ftblyp
>>enddo
```

SP-PDFT:

```
>>export DIST=0.95
>>foreach L in ( 1 ..  250)
>>eval DIST=$DIST+0.05
&GATEWAY
Coord
2
TaSi molecule
Ta 0 0 0
Si $DIST 0 0
BASIS= ANO-RCC-VTZP
GROUP=Full
RICD
&SEWARD
grid input
grid=ultrafine
end of grid input
&RASSCF
LumOrb
SYMMETRY=4
SPIN=4
LEVS=1.5
nActEl = 7 0 0
INACTIVE = 19 9 3 9
```

```
GASSCF
4
4 0 0 0
3 3
0 2 0 0
4 4
0 0 2 0
5 5
0 0 0 2
7 7
>>COPY $Project.RasOrb INPORB
&MCPDFT
KSDFT=ftblyp
NEWIPH
CIREstart
CIONLY
nActEl = 7 0 0
INACTIVE = 19 9 3 9
GASSCF
4
4 0 0 0
3 3
0 2 0 0
4 4
0 0 2 0
5 5
0 0 0 2
```

```
7 7
Symmetry=4
Spin=4
LEVS=1.5
orblisting
all
END
>>enddo
```

Ground State CASSCF Wavefunction at r_e for VSi, NbSi, and TaSi

Table S1: The composition and weights of the ground state $^4\Pi$ wavefunction for VSi. The molecular configurations are organized in terms of orbital symmetry.

Configuration					
a_1	b_2	a_2	b_1	Coefficient	Weight
0u20	d0	u0	uu	-0.62283	0.38792
0u20	u0	u0	20	0.44518	0.19818
0u20	u0	u0	02	-0.43631	0.19037
0u20	u0	d0	uu	-0.35977	0.12943
0u20	u0	u0	du	-0.12793	0.01637
0u02	d0	u0	uu	0.11969	0.01432
0u00	d2	u0	uu	-0.10748	0.01155
0u20	u0	u0	ud	0.09770	0.00954
0u02	u0	u0	20	-0.08553	0.00732
0u02	u0	u0	02	0.08382	0.00703
0u00	u2	u0	20	0.07682	0.00590
0u00	u2	u0	02	-0.07533	0.00567
0u02	u0	d0	uu	0.06911	0.00478
0u00	u2	d0	uu	-0.06211	0.00386

Table S2: The composition and weights of the ground state $^4\Pi$ wavefunction for NbSi. The molecular configurations are organized in terms of orbital symmetry.

Configuration					
a_1	b_2	a_2	b_1	Coefficient	Weight
00u2	u0	u0	20	-0.37736	0.14240
00u2	u0	u0	02	0.35578	0.12658
00u2	u0	u0	du	0.32134	0.10326
20u0	u0	u0	20	-0.25972	0.06746
20u0	u0	u0	02	0.24780	0.06141
00u2	u0	u0	ud	-0.23867	0.05696
20u0	u0	u0	du	0.22247	0.04949
u0du	u0	u0	20	0.21034	0.04424
u0du	u0	u0	02	-0.19973	0.03989
u0uu	u0	u0	dd	0.19158	0.03670
u0du	u0	u0	du	-0.18213	0.03317
u0uu	d0	u0	20	0.17160	0.02945
20u0	u0	u0	ud	-0.16521	0.02729
u0uu	d0	u0	02	-0.16265	0.02645
u0uu	d0	u0	du	-0.14762	0.02179
u0du	u0	u0	ud	0.13335	0.01778
u0uu	u0	d0	20	-0.13267	0.01760
u0uu	u0	d0	02	0.12575	0.01581
u0ud	u0	u0	20	0.12236	0.01497
u0ud	u0	u0	02	-0.11532	0.01330
u0uu	u0	d0	du	0.11421	0.01304
u0uu	d0	u0	ud	0.10974	0.01204
u0ud	u0	u0	du	-0.10316	0.01064
u0uu	u0	d0	ud	-0.08491	0.00721
u0ud	u0	u0	ud	0.08026	0.00644

Table S3: Configurations and weights corresponding to the ground state $^4\Pi$ wavefunction for TaSi. The molecular configurations are organized in terms of orbital symmetry.

Configuration					
a_1	b_2	a_2	b_1	Coefficient	Weight
20u0	d0	u0	uu	0.62041	0.38490
20u0	u0	u0	20	-0.45712	0.20896
20u0	u0	u0	02	0.42739	0.18266
20u0	u0	d0	uu	0.36010	0.12967
20u0	u0	u0	du	0.13680	0.01872
02u0	d0	u0	uu	-0.09237	0.00853
20u0	u0	u0	ud	-0.08435	0.00712
00u0	d2	u0	uu	-0.08088	0.00654
02u0	u0	u0	20	0.06785	0.00460
02u0	u0	u0	02	-0.06376	0.00407
00u0	u2	u0	20	0.05951	0.00354
u000	d2	u0	uu	0.05666	0.00321
u200	d0	u0	uu	0.05660	0.00320
00u0	u2	u0	02	-0.05583	0.00312
02u0	u0	d0	uu	-0.05352	0.00286

Active Space Natural Orbitals

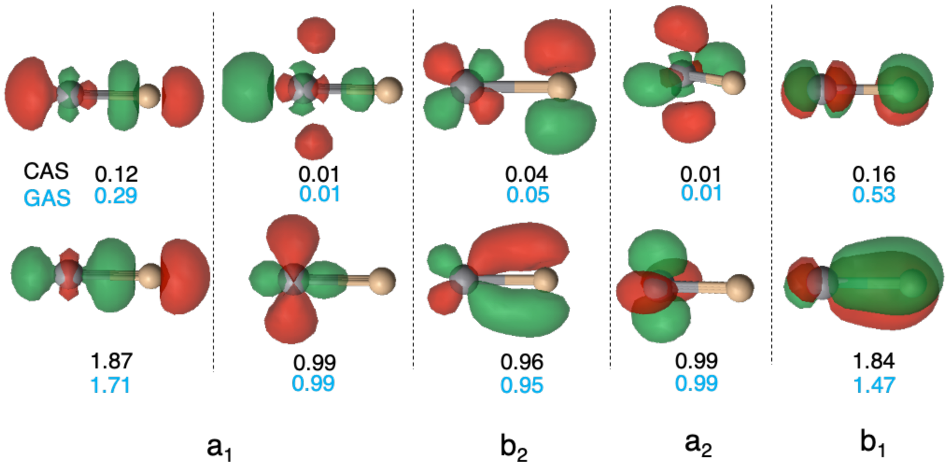

Figure S1: The active space natural (CASSCF) and pseudo-natural (SP) orbitals for vanadium silicide with natural orbital occupation numbers of the $^4\Pi$ state from CASSCF (black) and SP (blue) wave functions.

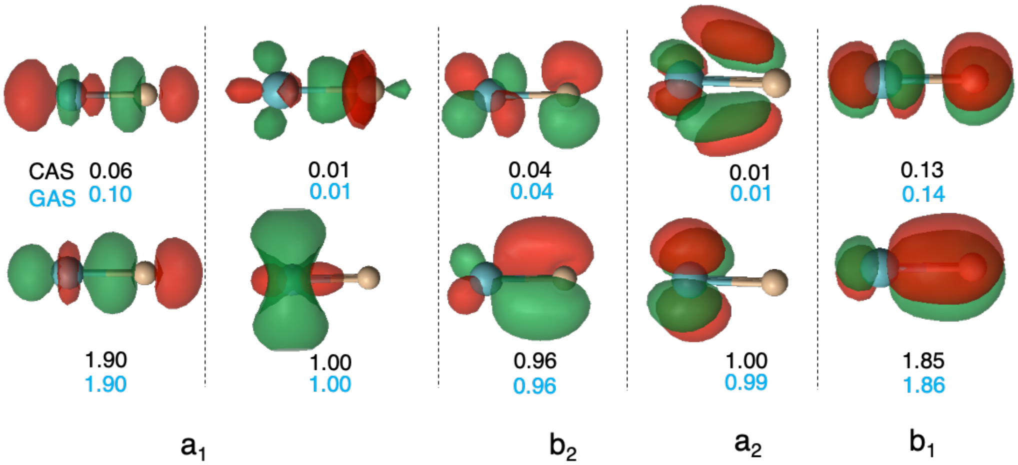

Figure S2: The active space natural (CASSCF) and pseudo-natural (SP) orbitals for niobium silicide with natural orbital occupation numbers of the $^4\Pi$ state from CASSCF (black) and SP (blue) wave functions.

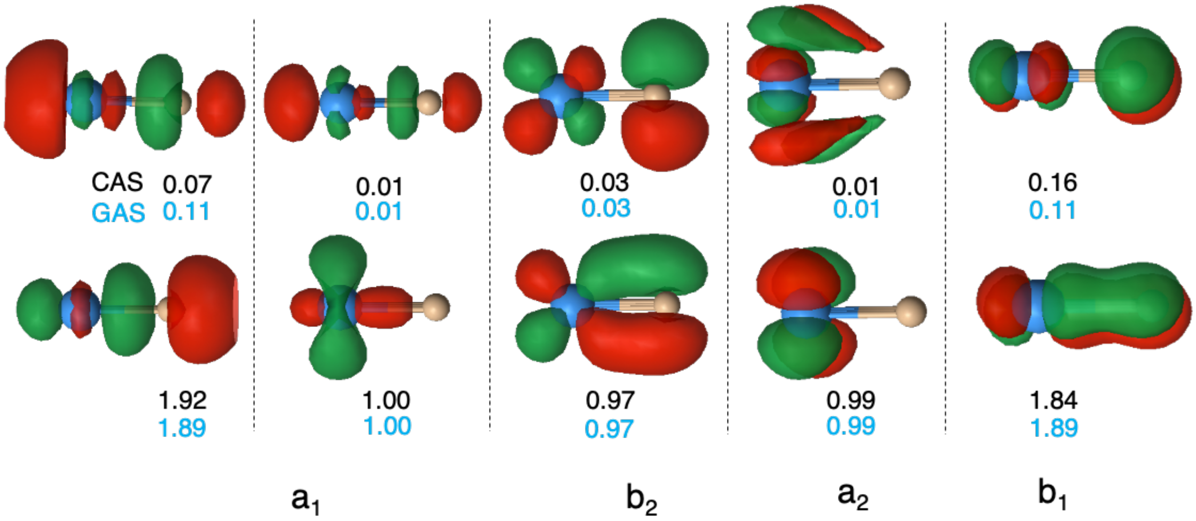

Figure S3: The active space natural (CASSCF) and pseudo-natural (SP) orbitals for tantalum silicide with natural orbital occupation numbers of the $^4\Pi$ state from CASSCF (black) and SP (blue) wave functions.

Absolute Energies for Potential Energy Curves

Table S4: The absolute energies in hartree of the potential energy curve of VSi in the $^6\Sigma^+$ state with a CASSCF wave function and nom-CPO active space.

r (bohr)	CASSCF	CASPT2	tpbe	ftpbe	trevpbe	ftrevpbe	tblyp	ftblyp	torelyp	ftorelyp
1.98	-1235.49304	-1235.49304	-1236.68265	-1236.43293	-1236.88938	-1237.09100	-1237.09530	-1237.29198	-1237.16526	-1237.38538
2.08	-1235.90976	-1235.90976	-1237.10313	-1236.88311	-1237.31102	-1237.51150	-1237.51632	-1237.71194	-1237.58275	-1237.80151
2.17	-1236.23744	-1236.23744	-1237.43120	-1237.30252	-1237.64018	-1237.83992	-1237.84499	-1238.03976	-1237.90825	-1238.12607
2.27	-1236.49813	-1236.49813	-1237.68823	-1237.62985	-1237.89822	-1238.09749	-1238.10270	-1238.29687	-1238.16312	-1238.38029
2.36	-1236.70410	-1236.70410	-1237.85266	-1238.05400	-1238.06307	-1238.26603	-1238.27192	-1238.47035	-1238.31666	-1238.53874
2.46	-1236.86770	-1236.86770	-1238.02563	-1238.22710	-1238.23685	-1238.43994	-1238.44554	-1238.64406	-1238.48825	-1238.71039
2.55	-1236.99856	-1236.99856	-1238.16671	-1238.36858	-1238.37876	-1238.58222	-1238.58713	-1238.78597	-1238.62824	-1238.85071
2.65	-1237.10403	-1237.10403	-1238.28244	-1238.48503	-1238.49535	-1238.69954	-1238.70310	-1238.90247	-1238.74300	-1238.96614
2.74	-1237.16120	-1237.16120	-1238.37896	-1238.58227	-1238.59273	-1238.79771	-1238.79960	-1238.99937	-1238.83879	-1239.06266
2.83	-1237.24379	-1237.24379	-1238.46088	-1238.66435	-1238.67542	-1238.88111	-1238.88092	-1239.08002	-1238.92007	-1239.14470
2.93	-1237.31666	-1237.31666	-1238.53137	-1238.73389	-1238.74639	-1238.95160	-1238.95099	-1239.14868	-1238.98993	-1239.21389
3.02	-1237.37884	-1237.37884	-1238.59122	-1238.79291	-1238.80671	-1239.01127	-1239.01081	-1239.20739	-1239.04940	-1239.27253
3.12	-1237.43130	-1237.43130	-1238.64156	-1238.84268	-1238.85749	-1239.06154	-1239.06125	-1239.25713	-1239.09938	-1239.32185
3.21	-1237.47530	-1237.47530	-1238.68359	-1238.88425	-1238.89993	-1239.10355	-1239.10344	-1239.29877	-1239.14108	-1239.36297
3.31	-1237.51199	-1237.51199	-1238.71843	-1238.91869	-1238.93518	-1239.13840	-1239.13850	-1239.33333	-1239.17565	-1239.39701
3.40	-1237.54241	-1237.54241	-1238.74711	-1238.94700	-1238.96423	-1239.16709	-1239.16741	-1239.36180	-1239.20410	-1239.42497
3.50	-1237.56744	-1237.56744	-1238.77050	-1238.97005	-1238.98797	-1239.19051	-1239.19107	-1239.38504	-1239.22733	-1239.44775
3.59	-1237.58786	-1237.58786	-1238.78939	-1238.98863	-1239.00720	-1239.20944	-1239.21025	-1239.40383	-1239.24610	-1239.46610
3.68	-1237.60435	-1237.60435	-1238.80443	-1239.00339	-1239.02255	-1239.22453	-1239.22561	-1239.41881	-1239.26106	-1239.48069
3.78	-1237.61751	-1237.61751	-1238.81620	-1239.01491	-1239.03461	-1239.23636	-1239.23771	-1239.43055	-1239.27279	-1239.49207
3.87	-1237.62787	-1237.62787	-1238.82517	-1239.02367	-1239.04387	-1239.24544	-1239.24703	-1239.43954	-1239.28175	-1239.50073
3.97	-1237.63588	-1237.63588	-1238.83177	-1239.03008	-1239.05074	-1239.25216	-1239.25397	-1239.44617	-1239.28833	-1239.50706
4.06	-1237.64195	-1237.64195	-1238.83631	-1239.03449	-1239.05553	-1239.25687	-1239.25886	-1239.45077	-1239.29285	-1239.51138
4.16	-1237.64643	-1237.64643	-1238.83906	-1239.03715	-1239.05853	-1239.25985	-1239.26195	-1239.45361	-1239.29555	-1239.51396
4.35	-1237.65183	-1237.65183	-1238.83997	-1239.03808	-1239.05991	-1239.26144	-1239.26346	-1239.45477	-1239.29623	-1239.51463
4.44	-1237.65324	-1237.65324	-1238.83847	-1239.03668	-1239.05864	-1239.26041	-1239.26221	-1239.45343	-1239.29454	-1239.51309
4.54	-1237.65405	-1237.65405	-1238.83594	-1239.03433	-1239.05636	-1239.25842	-1239.25991	-1239.45109	-1239.29182	-1239.51059
4.63	-1237.65439	-1237.65439	-1238.83262	-1239.03120	-1239.05328	-1239.25568	-1239.25677	-1239.44796	-1239.28830	-1239.50735
4.72	-1237.65437	-1237.65437	-1238.82872	-1239.02753	-1239.04963	-1239.25239	-1239.25303	-1239.44427	-1239.28423	-1239.50361
4.82	-1237.65407	-1237.65407	-1238.82449	-1239.02351	-1239.04565	-1239.24875	-1239.24894	-1239.44023	-1239.27987	-1239.49959
4.91	-1237.65351	-1237.65351	-1238.82012	-1239.01934	-1239.04152	-1239.24495	-1239.24471	-1239.43606	-1239.27543	-1239.49548
5.01	-1237.65275	-1237.65275	-1238.81559	-1239.01498	-1239.03723	-1239.24093	-1239.24033	-1239.43171	-1239.27087	-1239.49120
5.10	-1237.65241	-1237.65241	-1238.80781	-1239.00711	-1239.02967	-1239.23336	-1239.23260	-1239.42355	-1239.26252	-1239.48285
5.20	-1237.65153	-1237.65153	-1238.80530	-1239.00457	-1239.02732	-1239.23100	-1239.23032	-1239.42115	-1239.26012	-1239.48044
5.29	-1237.65042	-1237.65042	-1238.80284	-1239.00207	-1239.02502	-1239.22868	-1239.22811	-1239.41884	-1239.25779	-1239.47810
5.39	-1237.64912	-1237.64912	-1238.80041	-1238.99960	-1239.02273	-1239.22637	-1239.22594	-1239.41658	-1239.25551	-1239.47579
5.48	-1237.64767	-1237.64767	-1238.79802	-1238.99717	-1239.02047	-1239.22408	-1239.22382	-1239.41437	-1239.25325	-1239.47353
5.57	-1237.64610	-1237.64610	-1238.79565	-1238.99477	-1239.01822	-1239.22181	-1239.22174	-1239.41222	-1239.25103	-1239.47130
5.67	-1237.64444	-1237.64444	-1238.79332	-1238.99240	-1239.01600	-1239.21956	-1239.21972	-1239.41012	-1239.24884	-1239.46912
5.76	-1237.64273	-1237.64273	-1238.79102	-1238.99008	-1239.01381	-1239.21734	-1239.21774	-1239.40810	-1239.24669	-1239.46699
5.86	-1237.64098	-1237.64098	-1238.78876	-1238.98780	-1239.01165	-1239.21514	-1239.21582	-1239.40614	-1239.24457	-1239.46490
5.95	-1237.63923	-1237.63923	-1238.78655	-1238.98558	-1239.00952	-1239.21299	-1239.21396	-1239.40427	-1239.24250	-1239.46288
6.05	-1237.63749	-1237.63749	-1238.78438	-1238.98341	-1239.00743	-1239.21088	-1239.21216	-1239.40248	-1239.24048	-1239.46093
6.14	-1237.63580	-1237.63580	-1238.78225	-1238.98128	-1239.00538	-1239.20882	-1239.21044	-1239.40077	-1239.23850	-1239.45903
6.24	-1237.63416	-1237.63416	-1238.78017	-1238.97921	-1239.00336	-1239.20678	-1239.20877	-1239.39915	-1239.23655	-1239.45719
6.33	-1237.63259	-1237.63259	-1238.77811	-1238.97716	-1239.00136	-1239.20478	-1239.20715	-1239.39759	-1239.23464	-1239.45539
6.43	-1237.63111	-1237.63111	-1238.77608	-1238.97516	-1238.99939	-1239.20280	-1239.20558	-1239.39611	-1239.23276	-1239.45363
6.52	-1237.62973	-1237.62973	-1238.77407	-1238.97318	-1238.99743	-1239.20085	-1239.20404	-1239.39468	-1239.23091	-1239.45191

Table S4: The absolute energies in hartree of the potential energy curve of VSi in the $^6\Sigma^+$ state with a CASSCF wave function and nom-CPO active space.

r (bohr)	CASSCF	CASPT2	tpbe	ftpbe	trevpbe	ftrevpbe	tblyp	ftblyp	torelyp	ftorelyp
6.61	-1237.62846	-1237.62846	-1238.77208	-1238.97124	-1238.99549	-1239.19892	-1239.20254	-1239.39330	-1239.22908	-1239.45021
6.71	-1237.62729	-1237.62729	-1238.77012	-1238.96932	-1238.99359	-1239.19703	-1239.20107	-1239.39196	-1239.22729	-1239.44855
6.80	-1237.62625	-1237.62625	-1238.76820	-1238.96744	-1238.99172	-1239.19518	-1239.19963	-1239.39066	-1239.22555	-1239.44692
6.90	-1237.62531	-1237.62531	-1238.76634	-1238.96563	-1238.98991	-1239.19339	-1239.19824	-1239.38941	-1239.22387	-1239.44536
6.99	-1237.62448	-1237.62448	-1238.76457	-1238.96390	-1238.98819	-1239.19169	-1239.19691	-1239.38822	-1239.22228	-1239.44387
7.09	-1237.62376	-1237.62376	-1238.76289	-1238.96228	-1238.98657	-1239.19010	-1239.19565	-1239.38710	-1239.22081	-1239.44248
7.18	-1237.62312	-1237.62312	-1238.76135	-1238.96077	-1238.98509	-1239.18865	-1239.19450	-1239.38606	-1239.21946	-1239.44120
7.28	-1237.62256	-1237.62256	-1238.75994	-1238.95940	-1238.98373	-1239.18732	-1239.19344	-1239.38510	-1239.21824	-1239.44004
7.37	-1237.62208	-1237.62208	-1238.75866	-1238.95815	-1238.98252	-1239.18613	-1239.19248	-1239.38424	-1239.21716	-1239.43901
7.46	-1237.62166	-1237.62166	-1238.75751	-1238.95703	-1238.98143	-1239.18506	-1239.19163	-1239.38345	-1239.21621	-1239.43809
7.56	-1237.62128	-1237.62128	-1238.75648	-1238.95602	-1238.98047	-1239.18411	-1239.19086	-1239.38274	-1239.21537	-1239.43728
7.65	-1237.62096	-1237.62096	-1238.75556	-1238.95512	-1238.97961	-1239.18326	-1239.19017	-1239.38210	-1239.21463	-1239.43657
7.75	-1237.62067	-1237.62067	-1238.75474	-1238.95430	-1238.97885	-1239.18251	-1239.18956	-1239.38152	-1239.21399	-1239.43594
7.84	-1237.62041	-1237.62041	-1238.75400	-1238.95357	-1238.97817	-1239.18184	-1239.18901	-1239.38100	-1239.21344	-1239.43539
7.94	-1237.62019	-1237.62019	-1238.75333	-1238.95290	-1238.97757	-1239.18124	-1239.18852	-1239.38053	-1239.21295	-1239.43490
8.03	-1237.61998	-1237.61998	-1238.75273	-1238.95230	-1238.97703	-1239.18070	-1239.18808	-1239.38011	-1239.21252	-1239.43447
8.13	-1237.61980	-1237.61980	-1238.75219	-1238.95176	-1238.97655	-1239.18021	-1239.18769	-1239.37972	-1239.21215	-1239.43409
8.22	-1237.61964	-1237.61964	-1238.75170	-1238.95127	-1238.97612	-1239.17978	-1239.18734	-1239.37937	-1239.21183	-1239.43377
8.31	-1237.61949	-1237.61949	-1238.75127	-1238.95082	-1238.97574	-1239.17939	-1239.18702	-1239.37906	-1239.21156	-1239.43348
8.41	-1237.61936	-1237.61936	-1238.75087	-1238.95042	-1238.97540	-1239.17905	-1239.18674	-1239.37877	-1239.21132	-1239.43323
8.50	-1237.61924	-1237.61924	-1238.75051	-1238.95006	-1238.97510	-1239.17874	-1239.18649	-1239.37852	-1239.21111	-1239.43301
8.60	-1237.61914	-1237.61914	-1238.75019	-1238.94973	-1238.97484	-1239.17846	-1239.18627	-1239.37829	-1239.21094	-1239.43282
8.69	-1237.61904	-1237.61904	-1238.74990	-1238.94943	-1238.97460	-1239.17821	-1239.18607	-1239.37808	-1239.21079	-1239.43265
8.79	-1237.61895	-1237.61895	-1238.74964	-1238.94915	-1238.97439	-1239.17799	-1239.18589	-1239.37789	-1239.21066	-1239.43251
8.88	-1237.61887	-1237.61887	-1238.74940	-1238.94890	-1238.97420	-1239.17779	-1239.18573	-1239.37772	-1239.21056	-1239.43239
8.98	-1237.61880	-1237.61880	-1238.74919	-1238.94868	-1238.97403	-1239.17761	-1239.18559	-1239.37756	-1239.21047	-1239.43228
9.07	-1237.61873	-1237.61873	-1238.74899	-1238.94847	-1238.97388	-1239.17745	-1239.18546	-1239.37743	-1239.21040	-1239.43219
9.17	-1237.61867	-1237.61867	-1238.74882	-1238.94829	-1238.97375	-1239.17731	-1239.18535	-1239.37730	-1239.21034	-1239.43211
9.26	-1237.61862	-1237.61862	-1238.74866	-1238.94812	-1238.97364	-1239.17718	-1239.18525	-1239.37719	-1239.21029	-1239.43205
9.35	-1237.61857	-1237.61857	-1238.74852	-1238.94797	-1238.97354	-1239.17707	-1239.18516	-1239.37709	-1239.21026	-1239.43199
9.45	-1237.61852	-1237.61852	-1238.74839	-1238.94783	-1238.97345	-1239.17697	-1239.18509	-1239.37700	-1239.21023	-1239.43195
9.54	-1237.61848	-1237.61848	-1238.74827	-1238.94770	-1238.97337	-1239.17688	-1239.18502	-1239.37693	-1239.21020	-1239.43191
9.64	-1237.61844	-1237.61844	-1238.74817	-1238.94759	-1238.97330	-1239.17679	-1239.18496	-1239.37685	-1239.21019	-1239.43187
9.73	-1237.61841	-1237.61841	-1238.74807	-1238.94748	-1238.97324	-1239.17672	-1239.18491	-1239.37679	-1239.21018	-1239.43185
9.83	-1237.61838	-1237.61838	-1238.74799	-1238.94739	-1238.97319	-1239.17666	-1239.18486	-1239.37673	-1239.21017	-1239.43182
9.92	-1237.61835	-1237.61835	-1238.74791	-1238.94730	-1238.97314	-1239.17660	-1239.18483	-1239.37669	-1239.21017	-1239.43181
10.02	-1237.61832	-1237.61832	-1238.74784	-1238.94722	-1238.97310	-1239.17655	-1239.18479	-1239.37664	-1239.21017	-1239.43179
10.11	-1237.61830	-1237.61830	-1238.74778	-1238.94715	-1238.97307	-1239.17651	-1239.18477	-1239.37661	-1239.21017	-1239.43178
10.20	-1237.61827	-1237.61827	-1238.74772	-1238.94709	-1238.97304	-1239.17647	-1239.18474	-1239.37657	-1239.21018	-1239.43177
10.30	-1237.61825	-1237.61825	-1238.74767	-1238.94703	-1238.97301	-1239.17643	-1239.18472	-1239.37654	-1239.21019	-1239.43176
10.39	-1237.61823	-1237.61823	-1238.74762	-1238.94698	-1238.97299	-1239.17640	-1239.18471	-1239.37652	-1239.21019	-1239.43176
10.49	-1237.61822	-1237.61822	-1238.74758	-1238.94693	-1238.97297	-1239.17637	-1239.18469	-1239.37650	-1239.21020	-1239.43175
10.58	-1237.61820	-1237.61820	-1238.74754	-1238.94688	-1238.97295	-1239.17634	-1239.18468	-1239.37648	-1239.21021	-1239.43174
10.68	-1237.61819	-1237.61819	-1238.74750	-1238.94684	-1238.97293	-1239.17632	-1239.18467	-1239.37646	-1239.21022	-1239.43174
10.77	-1237.61817	-1237.61817	-1238.74747	-1238.94680	-1238.97292	-1239.17630	-1239.18467	-1239.37645	-1239.21023	-1239.43174
10.87	-1237.61816	-1237.61816	-1238.74744	-1238.94676	-1238.97291	-1239.17628	-1239.18467	-1239.37644	-1239.21023	-1239.43174
10.96	-1237.61815	-1237.61815	-1238.74741	-1238.94673	-1238.97290	-1239.17626	-1239.18466	-1239.37643	-1239.21024	-1239.43173
11.05	-1237.61814	-1237.61814	-1238.74739	-1238.94671	-1238.97289	-1239.17624	-1239.18466	-1239.37642	-1239.21025	-1239.43173
11.15	-1237.61812	-1237.61812	-1238.74737	-1238.94668	-1238.97288	-1239.17623	-1239.18466	-1239.37642	-1239.21026	-1239.43173
11.24	-1237.61812	-1237.61812	-1238.74735	-1238.94666	-1238.97287	-1239.17622	-1239.18466	-1239.37641	-1239.21027	-1239.43173
11.34	-1237.61811	-1237.61811	-1238.74733	-1238.94663	-1238.97287	-1239.17621	-1239.18466	-1239.37641	-1239.21027	-1239.43173
11.43	-1237.61810	-1237.61810	-1238.74731	-1238.94661	-1238.97286	-1239.17620	-1239.18466	-1239.37641	-1239.21028	-1239.43172

Table S4: The absolute energies in hartree of the potential energy curve of VSi in the $^6\Sigma^+$ state with a CASSCF wave function and nom-CPO active space.

r (bohr)	CASSCF	CASPT2	tpbe	ftpbe	trevpbe	ftrevpbe	tblyp	ftblyp	torelyp	ftorelyp
11.53	-1237.61809	-1237.61809	-1238.74729	-1238.94659	-1238.97285	-1239.17619	-1239.18467	-1239.37640	-1239.21028	-1239.43172
11.62	-1237.61808	-1237.61808	-1238.74728	-1238.94657	-1238.97285	-1239.17617	-1239.18467	-1239.37640	-1239.21029	-1239.43172
11.72	-1237.61808	-1237.61808	-1238.74726	-1238.94656	-1238.97284	-1239.17617	-1239.18468	-1239.37640	-1239.21029	-1239.43171
11.81	-1237.61807	-1237.61807	-1238.74725	-1238.94654	-1238.97284	-1239.17616	-1239.18468	-1239.37640	-1239.21030	-1239.43171
11.91	-1237.61806	-1237.61806	-1238.74724	-1238.94653	-1238.97284	-1239.17615	-1239.18469	-1239.37641	-1239.21030	-1239.43171
12.00	-1237.61806	-1237.61806	-1238.74723	-1238.94651	-1238.97283	-1239.17614	-1239.18469	-1239.37641	-1239.21030	-1239.43171
12.09	-1237.61805	-1237.61805	-1238.74722	-1238.94650	-1238.97283	-1239.17614	-1239.18470	-1239.37641	-1239.21031	-1239.43171
12.19	-1237.61805	-1237.61805	-1238.74721	-1238.94649	-1238.97283	-1239.17613	-1239.18470	-1239.37641	-1239.21031	-1239.43170
12.28	-1237.61804	-1237.61804	-1238.74720	-1238.94648	-1238.97283	-1239.17613	-1239.18471	-1239.37642	-1239.21031	-1239.43170
12.38	-1237.61804	-1237.61804	-1238.74720	-1238.94647	-1238.97282	-1239.17612	-1239.18471	-1239.37642	-1239.21031	-1239.43170
12.47	-1237.61804	-1237.61804	-1238.74719	-1238.94647	-1238.97282	-1239.17612	-1239.18472	-1239.37642	-1239.21031	-1239.43170
12.57	-1237.61803	-1237.61803	-1238.74718	-1238.94646	-1238.97282	-1239.17611	-1239.18472	-1239.37642	-1239.21032	-1239.43169
12.66	-1237.61803	-1237.61803	-1238.74717	-1238.94645	-1238.97282	-1239.17611	-1239.18473	-1239.37643	-1239.21032	-1239.43169
12.76	-1237.61802	-1237.61802	-1238.74717	-1238.94644	-1238.97281	-1239.17610	-1239.18473	-1239.37643	-1239.21032	-1239.43169
12.85	-1237.61802	-1237.61802	-1238.74716	-1238.94643	-1238.97281	-1239.17610	-1239.18474	-1239.37643	-1239.21032	-1239.43168
12.94	-1237.61802	-1237.61802	-1238.74716	-1238.94643	-1238.97281	-1239.17609	-1239.18474	-1239.37644	-1239.21032	-1239.43168
13.04	-1237.61802	-1237.61802	-1238.74715	-1238.94642	-1238.97281	-1239.17609	-1239.18475	-1239.37644	-1239.21032	-1239.43168
13.13	-1237.61801	-1237.61801	-1238.74715	-1238.94642	-1238.97281	-1239.17609	-1239.18475	-1239.37644	-1239.21032	-1239.43168
13.23	-1237.61801	-1237.61801	-1238.74715	-1238.94641	-1238.97281	-1239.17609	-1239.18476	-1239.37645	-1239.21032	-1239.43167
13.32	-1237.61801	-1237.61801	-1238.74714	-1238.94641	-1238.97280	-1239.17609	-1239.18476	-1239.37645	-1239.21032	-1239.43167
13.42	-1237.61801	-1237.61801	-1238.74714	-1238.94641	-1238.97280	-1239.17608	-1239.18476	-1239.37645	-1239.21032	-1239.43167
13.51	-1237.61800	-1237.61800	-1238.74714	-1238.94640	-1238.97280	-1239.17608	-1239.18477	-1239.37646	-1239.21032	-1239.43167
13.61	-1237.61800	-1237.61800	-1238.74713	-1238.94640	-1238.97280	-1239.17608	-1239.18477	-1239.37646	-1239.21032	-1239.43167
13.70	-1237.61800	-1237.61800	-1238.74713	-1238.94640	-1238.97280	-1239.17608	-1239.18478	-1239.37646	-1239.21032	-1239.43167
13.80	-1237.61800	-1237.61800	-1238.74713	-1238.94640	-1238.97280	-1239.17608	-1239.18478	-1239.37647	-1239.21032	-1239.43167
13.89	-1237.61800	-1237.61800	-1238.74713	-1238.94639	-1238.97280	-1239.17607	-1239.18479	-1239.37647	-1239.21032	-1239.43166
13.98	-1237.61799	-1237.61799	-1238.74713	-1238.94639	-1238.97280	-1239.17607	-1239.18479	-1239.37647	-1239.21032	-1239.43166
14.08	-1237.61799	-1237.61799	-1238.74712	-1238.94639	-1238.97280	-1239.17607	-1239.18479	-1239.37648	-1239.21032	-1239.43166
14.17	-1237.61799	-1237.61799	-1238.74712	-1238.94639	-1238.97280	-1239.17607	-1239.18480	-1239.37648	-1239.21032	-1239.43166
14.27	-1237.61799	-1237.61799	-1238.74712	-1238.94638	-1238.97279	-1239.17607	-1239.18480	-1239.37648	-1239.21032	-1239.43166
14.36	-1237.61799	-1237.61799	-1238.74712	-1238.94638	-1238.97279	-1239.17607	-1239.18480	-1239.37648	-1239.21031	-1239.43166
14.46	-1237.61799	-1237.61799	-1238.74712	-1238.94638	-1238.97279	-1239.17606	-1239.18480	-1239.37649	-1239.21031	-1239.43165
14.55	-1237.61798	-1237.61798	-1238.74712	-1238.94638	-1238.97279	-1239.17606	-1239.18481	-1239.37649	-1239.21031	-1239.43165
14.65	-1237.61798	-1237.61798	-1238.74712	-1238.94638	-1238.97279	-1239.17606	-1239.18481	-1239.37649	-1239.21031	-1239.43165
14.74	-1237.61798	-1237.61798	-1238.74711	-1238.94638	-1238.97279	-1239.17606	-1239.18481	-1239.37649	-1239.21031	-1239.43165
14.83	-1237.61798	-1237.61798	-1238.74711	-1238.94638	-1238.97279	-1239.17606	-1239.18481	-1239.37649	-1239.21031	-1239.43165
14.93	-1237.61798	-1237.61798	-1238.74711	-1238.94637	-1238.97279	-1239.17606	-1239.18482	-1239.37650	-1239.21031	-1239.43165
15.02	-1237.61798	-1237.61798	-1238.74711	-1238.94637	-1238.97279	-1239.17606	-1239.18482	-1239.37650	-1239.21031	-1239.43165
15.12	-1237.61798	-1237.61798	-1238.74711	-1238.94637	-1238.97279	-1239.17606	-1239.18482	-1239.37650	-1239.21031	-1239.43165
15.21	-1237.61798	-1237.61798	-1238.74711	-1238.94637	-1238.97279	-1239.17606	-1239.18482	-1239.37650	-1239.21031	-1239.43165
15.31	-1237.61798	-1237.61798	-1238.74711	-1238.94637	-1238.97279	-1239.17606	-1239.18482	-1239.37650	-1239.21031	-1239.43165
15.40	-1237.61798	-1237.61798	-1238.74711	-1238.94637	-1238.97279	-1239.17606	-1239.18483	-1239.37650	-1239.21031	-1239.43165
15.50	-1237.61797	-1237.61797	-1238.74711	-1238.94637	-1238.97279	-1239.17605	-1239.18483	-1239.37650	-1239.21031	-1239.43165
15.59	-1237.61797	-1237.61797	-1238.74711	-1238.94637	-1238.97279	-1239.17605	-1239.18483	-1239.37651	-1239.21031	-1239.43164
15.68	-1237.61797	-1237.61797	-1238.74711	-1238.94637	-1238.97278	-1239.17605	-1239.18483	-1239.37651	-1239.21031	-1239.43164
15.78	-1237.61797	-1237.61797	-1238.74711	-1238.94637	-1238.97278	-1239.17605	-1239.18483	-1239.37651	-1239.21031	-1239.43164
15.87	-1237.61797	-1237.61797	-1238.74710	-1238.94637	-1238.97278	-1239.17605	-1239.18483	-1239.37651	-1239.21031	-1239.43164
15.97	-1237.61797	-1237.61797	-1238.74710	-1238.94637	-1238.97278	-1239.17605	-1239.18483	-1239.37651	-1239.21031	-1239.43164
16.06	-1237.61797	-1237.61797	-1238.74710	-1238.94637	-1238.97278	-1239.17605	-1239.18483	-1239.37651	-1239.21031	-1239.43164
16.16	-1237.61797	-1237.61797	-1238.74710	-1238.94636	-1238.97278	-1239.17605	-1239.18483	-1239.37651	-1239.21031	-1239.43164
16.25	-1237.61797	-1237.61797	-1238.74710	-1238.94636	-1238.97278	-1239.17605	-1239.18484	-1239.37651	-1239.21031	-1239.43164
16.35	-1237.61797	-1237.61797	-1238.74710	-1238.94636	-1238.97278	-1239.17605	-1239.18484	-1239.37651	-1239.21031	-1239.43164

Table S4: The absolute energies in hartree of the potential energy curve of VSi in the $^6\Sigma^+$ state with a CASSCF wave function and nom-CPO active space.

r (bohr)	CASSCF	CASPT2	tpbe	ftpbe	trevpbe	ftrevpbe	tblyp	ftblyp	torelyp	ftorelyp
16.44	-1237.61797	-1237.61797	-1238.74710	-1238.94636	-1238.97278	-1239.17605	-1239.18484	-1239.37651	-1239.21031	-1239.43164
16.54	-1237.61797	-1237.61797	-1238.74710	-1238.94636	-1238.97278	-1239.17605	-1239.18484	-1239.37651	-1239.21031	-1239.43164
16.63	-1237.61797	-1237.61797	-1238.74710	-1238.94636	-1238.97278	-1239.17605	-1239.18484	-1239.37651	-1239.21031	-1239.43164
16.72	-1237.61797	-1237.61797	-1238.74710	-1238.94636	-1238.97278	-1239.17605	-1239.18484	-1239.37651	-1239.21031	-1239.43164
16.82	-1237.61797	-1237.61797	-1238.74710	-1238.94636	-1238.97278	-1239.17605	-1239.18484	-1239.37651	-1239.21031	-1239.43164
16.91	-1237.61797	-1237.61797	-1238.74710	-1238.94636	-1238.97278	-1239.17605	-1239.18484	-1239.37651	-1239.21031	-1239.43164
17.01	-1237.61797	-1237.61797	-1238.74710	-1238.94636	-1238.97278	-1239.17605	-1239.18484	-1239.37651	-1239.21031	-1239.43164
17.10	-1237.61797	-1237.61797	-1238.74710	-1238.94636	-1238.97278	-1239.17605	-1239.18484	-1239.37651	-1239.21030	-1239.43164
17.20	-1237.61797	-1237.61797	-1238.74710	-1238.94636	-1238.97278	-1239.17605	-1239.18484	-1239.37651	-1239.21030	-1239.43164
17.29	-1237.61796	-1237.61796	-1238.74710	-1238.94636	-1238.97278	-1239.17605	-1239.18484	-1239.37651	-1239.21030	-1239.43164
17.39	-1237.61796	-1237.61796	-1238.74710	-1238.94636	-1238.97278	-1239.17605	-1239.18484	-1239.37651	-1239.21030	-1239.43164
17.48	-1237.61796	-1237.61796	-1238.74710	-1238.94636	-1238.97278	-1239.17605	-1239.18484	-1239.37651	-1239.21030	-1239.43164
17.57	-1237.61796	-1237.61796	-1238.74710	-1238.94636	-1238.97278	-1239.17605	-1239.18484	-1239.37651	-1239.21030	-1239.43164
17.67	-1237.61796	-1237.61796	-1238.74710	-1238.94636	-1238.97278	-1239.17605	-1239.18485	-1239.37651	-1239.21030	-1239.43164
17.76	-1237.61796	-1237.61796	-1238.74710	-1238.94636	-1238.97278	-1239.17605	-1239.18485	-1239.37651	-1239.21030	-1239.43164
17.86	-1237.61796	-1237.61796	-1238.74710	-1238.94636	-1238.97278	-1239.17605	-1239.18485	-1239.37651	-1239.21030	-1239.43164
17.95	-1237.61796	-1237.61796	-1238.74710	-1238.94636	-1238.97278	-1239.17605	-1239.18485	-1239.37651	-1239.21030	-1239.43164
18.05	-1237.61796	-1237.61796	-1238.74710	-1238.94636	-1238.97278	-1239.17605	-1239.18485	-1239.37651	-1239.21030	-1239.43164

Table S5: The absolute energies in hartree of the potential energy curve of VSi in the $^4\Pi$ state with a CASSCF wave function and nom-CPO active space.

Distance	CASSCF	CASPT2	tpbe	ftpbe	trevpbe	ftrevpbe	tblyp	ftblyp	torelyp	ftorelyp
1.98	-1235.42564	-1235.96998	-1236.69023	-1236.89100	-1236.89620	-1237.10075	-1237.10031	-1237.29211	-1237.16570	-1237.38751
2.08	-1235.84533	-1236.39046	-1237.10394	-1237.30342	-1237.31102	-1237.51431	-1237.51503	-1237.70527	-1237.57712	-1237.79729
2.17	-1236.17293	-1236.71850	-1237.42670	-1237.62503	-1237.63481	-1237.83697	-1237.83872	-1238.02755	-1237.89825	-1238.11683
2.27	-1236.41049	-1236.93635	-1237.64234	-1237.83982	-1237.85063	-1238.05207	-1238.05682	-1238.24633	-1238.10385	-1238.32144
2.36	-1236.62735	-1237.15205	-1237.85711	-1238.05443	-1238.06640	-1238.26776	-1238.27213	-1238.46132	-1238.31725	-1238.53440
2.46	-1236.80103	-1237.32599	-1238.03023	-1238.22733	-1238.24040	-1238.44157	-1238.44573	-1238.63456	-1238.48917	-1238.70591
2.55	-1236.94126	-1237.46702	-1238.17081	-1238.36773	-1238.38181	-1238.58280	-1238.58672	-1238.77524	-1238.62880	-1238.84521
2.65	-1237.05557	-1237.58253	-1238.28616	-1238.48294	-1238.49791	-1238.69878	-1238.70242	-1238.89067	-1238.74342	-1238.95958
2.74	-1237.14966	-1237.60084	-1238.31149	-1238.51428	-1238.52447	-1238.73129	-1238.72968	-1238.92327	-1238.76974	-1238.99454
2.83	-1237.22781	-1237.68813	-1238.39894	-1238.60158	-1238.61229	-1238.81942	-1238.81674	-1239.01006	-1238.85555	-1239.08099
2.93	-1237.29322	-1237.76634	-1238.47604	-1238.67780	-1238.68966	-1238.89622	-1238.89365	-1239.08610	-1238.93177	-1239.15655
3.02	-1237.34832	-1237.83546	-1238.54286	-1238.74361	-1238.75679	-1238.96245	-1238.96050	-1239.15188	-1238.99847	-1239.22207
3.12	-1237.39503	-1237.89409	-1238.59923	-1238.79919	-1238.81357	-1239.01841	-1239.01712	-1239.20755	-1239.05499	-1239.27752
3.21	-1237.43491	-1237.94243	-1238.64661	-1238.84598	-1238.86138	-1239.06558	-1239.06479	-1239.25452	-1239.10221	-1239.32392
3.31	-1237.46746	-1237.98254	-1238.68651	-1238.88541	-1238.90171	-1239.10537	-1239.10499	-1239.29418	-1239.14182	-1239.36283
3.40	-1237.49751	-1238.01590	-1238.71998	-1238.91845	-1238.93559	-1239.13878	-1239.13877	-1239.32750	-1239.17499	-1239.39538
3.50	-1237.52104	-1238.04352	-1238.74789	-1238.94596	-1238.96388	-1239.16663	-1239.16701	-1239.35533	-1239.20261	-1239.42242
3.59	-1237.54159	-1238.06624	-1238.77094	-1238.96865	-1238.98730	-1239.18964	-1239.19042	-1239.37836	-1239.22543	-1239.44470
3.68	-1237.55872	-1238.08473	-1238.78976	-1238.98714	-1239.00647	-1239.20844	-1239.20961	-1239.39721	-1239.24406	-1239.46283
3.78	-1237.57293	-1238.09958	-1238.80491	-1239.00198	-1239.02196	-1239.22359	-1239.22514	-1239.41244	-1239.25907	-1239.47737
3.87	-1237.58464	-1238.11131	-1238.81687	-1239.01367	-1239.03424	-1239.23556	-1239.23748	-1239.42451	-1239.27092	-1239.48880
3.97	-1237.59425	-1238.12035	-1238.82602	-1239.02260	-1239.04371	-1239.24476	-1239.24702	-1239.43383	-1239.28001	-1239.49750
4.06	-1237.60213	-1238.12707	-1238.83275	-1239.02914	-1239.05073	-1239.25156	-1239.25411	-1239.44075	-1239.28669	-1239.50385
4.16	-1237.63316	-1238.13180	-1238.83735	-1239.03360	-1239.05562	-1239.25628	-1239.25908	-1239.44560	-1239.29127	-1239.50816
4.35	-1237.64265	-1238.13631	-1238.84117	-1239.03728	-1239.06002	-1239.26046	-1239.26358	-1239.45000	-1239.29511	-1239.51161
4.44	-1237.64555	-1238.13655	-1238.84083	-1239.03692	-1239.05995	-1239.26036	-1239.26354	-1239.44999	-1239.29479	-1239.51119
4.54	-1237.64749	-1238.13571	-1238.83926	-1239.03539	-1239.05866	-1239.25908	-1239.26224	-1239.44876	-1239.29325	-1239.50961
4.63	-1237.64865	-1238.13397	-1238.83665	-1239.03285	-1239.05633	-1239.25680	-1239.25988	-1239.44650	-1239.29068	-1239.50706

Table S5: The absolute energies in hartree of the potential energy curve of VSi in the $^4\Pi$ state with a CASSCF wave function and nom-CPO active space.

Distance	CASSCF	CASPT2	tpbe	ftpbe	trevpbe	ftrevpbe	tblyp	ftblyp	torelyp	ftorelyp
4.72	-1237.64917	-1238.13156	-1238.83327	-1239.02956	-1239.05322	-1239.25378	-1239.25671	-1239.44347	-1239.28733	-1239.50380
4.82	-1237.64916	-1238.12865	-1238.82933	-1239.02574	-1239.04955	-1239.25022	-1239.25296	-1239.43986	-1239.28344	-1239.50003
4.91	-1237.64873	-1238.12541	-1238.82506	-1239.02160	-1239.04554	-1239.24635	-1239.24886	-1239.43591	-1239.27923	-1239.49598
5.01	-1237.64795	-1238.12198	-1238.82068	-1239.01733	-1239.04140	-1239.24234	-1239.24463	-1239.43183	-1239.27492	-1239.49184
5.10	-1237.64687	-1238.11848	-1238.81631	-1239.01308	-1239.03727	-1239.23834	-1239.24042	-1239.42775	-1239.27065	-1239.48774
5.20	-1237.64554	-1238.11497	-1238.81204	-1239.00892	-1239.03322	-1239.23441	-1239.23632	-1239.42377	-1239.26649	-1239.48375
5.29	-1237.64402	-1238.11149	-1238.80792	-1239.00490	-1239.02931	-1239.23061	-1239.23237	-1239.41995	-1239.26251	-1239.47993
5.39	-1237.64233	-1238.10807	-1238.80395	-1239.00103	-1239.02553	-1239.22693	-1239.22859	-1239.41628	-1239.25869	-1239.47627
5.48	-1237.64051	-1238.10473	-1238.80016	-1238.99732	-1239.02191	-1239.22340	-1239.22500	-1239.41280	-1239.25507	-1239.47280
5.57	-1237.63859	-1238.10147	-1238.79652	-1238.99377	-1239.01844	-1239.22002	-1239.22159	-1239.40951	-1239.25162	-1239.46950
5.67	-1237.63659	-1238.09833	-1238.79307	-1238.99040	-1239.01514	-1239.21679	-1239.21839	-1239.40642	-1239.24836	-1239.46640
5.76	-1237.63455	-1238.09528	-1238.78976	-1238.98718	-1239.01196	-1239.21369	-1239.21534	-1239.40351	-1239.24527	-1239.46346
5.86	-1237.63249	-1238.09235	-1238.78661	-1238.98412	-1239.00894	-1239.21074	-1239.21249	-1239.40079	-1239.24234	-1239.46070
5.95	-1237.63042	-1238.08955	-1238.78362	-1238.98122	-1239.00607	-1239.20794	-1239.20983	-1239.39828	-1239.23960	-1239.45813
6.05	-1237.62838	-1238.08690	-1238.78080	-1238.97850	-1239.00336	-1239.20529	-1239.20736	-1239.39598	-1239.23703	-1239.45575
6.14	-1237.62639	-1238.08438	-1238.77814	-1238.97593	-1239.00079	-1239.20278	-1239.20508	-1239.39389	-1239.23463	-1239.45353
6.24	-1237.62445	-1238.08201	-1238.77562	-1238.97351	-1238.99836	-1239.20040	-1239.20298	-1239.39199	-1239.23239	-1239.45149
6.33	-1237.62260	-1238.07980	-1238.77325	-1238.97124	-1238.99606	-1239.19815	-1239.20106	-1239.39029	-1239.23029	-1239.44959
6.43	-1237.62084	-1238.07773	-1238.77101	-1238.96910	-1238.99389	-1239.19601	-1239.19931	-1239.38876	-1239.22834	-1239.44783
6.52	-1237.61919	-1238.07581	-1238.76888	-1238.96706	-1238.99182	-1239.19398	-1239.19769	-1239.38738	-1239.22650	-1239.44619
6.61	-1237.61767	-1238.07403	-1238.76685	-1238.96513	-1238.98984	-1239.19204	-1239.19620	-1239.38614	-1239.22475	-1239.44463
6.71	-1237.61629	-1238.07239	-1238.76491	-1238.96328	-1238.98795	-1239.19018	-1239.19482	-1239.38499	-1239.22310	-1239.44315
6.80	-1237.61504	-1238.07088	-1238.76306	-1238.96151	-1238.98614	-1239.18840	-1239.19351	-1239.38392	-1239.22152	-1239.44174
6.90	-1237.61393	-1238.06949	-1238.76129	-1238.95982	-1238.98443	-1239.18672	-1239.19229	-1239.38291	-1239.22002	-1239.44040
6.99	-1237.61295	-1238.06820	-1238.75964	-1238.95824	-1238.98282	-1239.18515	-1239.19115	-1239.38196	-1239.21862	-1239.43913
7.09	-1237.61209	-1238.06702	-1238.75810	-1238.95677	-1238.98133	-1239.18370	-1239.19008	-1239.38107	-1239.21733	-1239.43796
7.18	-1237.61134	-1238.06594	-1238.75670	-1238.95542	-1238.97997	-1239.18238	-1239.18910	-1239.38023	-1239.21616	-1239.43688
7.28	-1237.61068	-1238.06495	-1238.75541	-1238.95418	-1238.97874	-1239.18119	-1239.18820	-1239.37944	-1239.21510	-1239.43590
7.37	-1237.61010	-1238.06404	-1238.75424	-1238.95304	-1238.97763	-1239.18010	-1239.18738	-1239.37871	-1239.21415	-1239.43501
7.46	-1237.60959	-1238.06320	-1238.75318	-1238.95201	-1238.97662	-1239.17911	-1239.18663	-1239.37802	-1239.21329	-1239.43420
7.56	-1237.60914	-1238.06244	-1238.75222	-1238.95106	-1238.97571	-1239.17822	-1239.18594	-1239.37739	-1239.21252	-1239.43347
7.65	-1237.60874	-1238.06174	-1238.75134	-1238.95019	-1238.97489	-1239.17741	-1239.18531	-1239.37681	-1239.21184	-1239.43281
7.75	-1237.60837	-1238.06110	-1238.75054	-1238.94940	-1238.97415	-1239.17668	-1239.18474	-1239.37627	-1239.21123	-1239.43222
7.84	-1237.60805	-1238.06051	-1238.74981	-1238.94868	-1238.97348	-1239.17601	-1239.18422	-1239.37578	-1239.21069	-1239.43169
7.94	-1237.60776	-1238.05997	-1238.74914	-1238.94802	-1238.97287	-1239.17541	-1239.18375	-1239.37532	-1239.21021	-1239.43121
8.03	-1237.60750	-1238.05948	-1238.74854	-1238.94741	-1238.97232	-1239.17486	-1239.18333	-1239.37491	-1239.20978	-1239.43079
8.13	-1237.60726	-1238.05902	-1238.74799	-1238.94686	-1238.97183	-1239.17436	-1239.18294	-1239.37453	-1239.20941	-1239.43041
8.22	-1237.60705	-1238.05860	-1238.74748	-1238.94635	-1238.97138	-1239.17391	-1239.18258	-1239.37417	-1239.20907	-1239.43007
8.31	-1237.60685	-1238.05821	-1238.74702	-1238.94588	-1238.97098	-1239.17350	-1239.18226	-1239.37385	-1239.20878	-1239.42977
8.41	-1237.60667	-1238.05785	-1238.74661	-1238.94546	-1238.97061	-1239.17313	-1239.18197	-1239.37356	-1239.20853	-1239.42950
8.50	-1237.60651	-1238.05752	-1238.74623	-1238.94507	-1238.97029	-1239.17280	-1239.18171	-1239.37330	-1239.20831	-1239.42927
8.60	-1237.60637	-1238.05721	-1238.74588	-1238.94472	-1238.97000	-1239.17249	-1239.18148	-1239.37306	-1239.20812	-1239.42906
8.69	-1237.60623	-1238.05693	-1238.74557	-1238.94439	-1238.96974	-1239.17222	-1239.18127	-1239.37284	-1239.20795	-1239.42888
8.79	-1237.60611	-1238.05667	-1238.74528	-1238.94410	-1238.96950	-1239.17197	-1239.18108	-1239.37264	-1239.20781	-1239.42872
8.88	-1237.60600	-1238.05642	-1238.74502	-1238.94383	-1238.96929	-1239.17175	-1239.18091	-1239.37245	-1239.20769	-1239.42858
8.98	-1237.60590	-1238.05620	-1238.74479	-1238.94358	-1238.96910	-1239.17155	-1239.18075	-1239.37229	-1239.20759	-1239.42847
9.07	-1237.60581	-1238.05599	-1238.74458	-1238.94336	-1238.96894	-1239.17137	-1239.18062	-1239.37214	-1239.20751	-1239.42836
9.17	-1237.60572	-1238.05579	-1238.74439	-1238.94315	-1238.96879	-1239.17121	-1239.18050	-1239.37201	-1239.20744	-1239.42828
9.26	-1237.60564	-1238.05561	-1238.74421	-1238.94297	-1238.96866	-1239.17107	-1239.18039	-1239.37189	-1239.20739	-1239.42820
9.35	-1237.60557	-1238.05545	-1238.74406	-1238.94280	-1238.96855	-1239.17094	-1239.18030	-1239.37179	-1239.20734	-1239.42814
9.45	-1237.60551	-1238.05529	-1238.74391	-1238.94265	-1238.96844	-1239.17083	-1239.18021	-1239.37169	-1239.20731	-1239.42808
9.54	-1237.60545	-1238.05515	-1238.74378	-1238.94251	-1238.96835	-1239.17072	-1239.18014	-1239.37160	-1239.20728	-1239.42804

Table S5: The absolute energies in hartree of the potential energy curve of VSi in the $^4\Pi$ state with a CASSCF wave function and nom-CPO active space.

Distance	CASSCF	CASPT2	tpbe	ftpbe	trevpbe	ftrevpbe	tblyp	ftblyp	torelyp	ftorelyp
9.64	-1237.60539	-1238.05501	-1238.74366	-1238.94238	-1238.96827	-1239.17063	-1239.18007	-1239.37152	-1239.20726	-1239.42800
9.73	-1237.60534	-1238.05489	-1238.74356	-1238.94226	-1238.96820	-1239.17055	-1239.18002	-1239.37146	-1239.20725	-1239.42797
9.83	-1237.60529	-1238.05477	-1238.74346	-1238.94216	-1238.96814	-1239.17047	-1239.17997	-1239.37140	-1239.20724	-1239.42795
9.92	-1237.60525	-1238.05466	-1238.74338	-1238.94206	-1238.96809	-1239.17041	-1239.17992	-1239.37134	-1239.20723	-1239.42792
10.02	-1237.60521	-1238.05456	-1238.74330	-1238.94197	-1238.96804	-1239.17035	-1239.17989	-1239.37129	-1239.20723	-1239.42791
10.11	-1237.60517	-1238.05446	-1238.74323	-1238.94190	-1238.96800	-1239.17030	-1239.17986	-1239.37125	-1239.20724	-1239.42789
10.20	-1237.60514	-1238.05437	-1238.74317	-1238.94183	-1238.96797	-1239.17025	-1239.17983	-1239.37122	-1239.20725	-1239.42789
10.30	-1237.60511	-1238.05429	-1238.74311	-1238.94176	-1238.96794	-1239.17021	-1239.17981	-1239.37119	-1239.20725	-1239.42788
10.39	-1237.60508	-1238.05420	-1238.74306	-1238.94170	-1238.96791	-1239.17017	-1239.17979	-1239.37116	-1239.20726	-1239.42787
10.49	-1237.60505	-1238.05413	-1238.74301	-1238.94165	-1238.96789	-1239.17014	-1239.17978	-1239.37113	-1239.20727	-1239.42786
10.58	-1237.60503	-1238.05406	-1238.74296	-1238.94159	-1238.96786	-1239.17011	-1239.17976	-1239.37111	-1239.20728	-1239.42786
10.68	-1237.60500	-1238.05398	-1238.74293	-1238.94155	-1238.96785	-1239.17008	-1239.17975	-1239.37110	-1239.20729	-1239.42785
10.77	-1237.60498	-1238.05392	-1238.74289	-1238.94150	-1238.96783	-1239.17006	-1239.17975	-1239.37108	-1239.20730	-1239.42785
10.87	-1237.60496	-1238.05386	-1238.74286	-1238.94147	-1238.96782	-1239.17004	-1239.17974	-1239.37107	-1239.20731	-1239.42785
10.96	-1237.60494	-1238.05381	-1238.74283	-1238.94143	-1238.96781	-1239.17002	-1239.17974	-1239.37106	-1239.20733	-1239.42785
11.05	-1237.60493	-1238.05304	-1238.74280	-1238.94140	-1238.96780	-1239.17000	-1239.17974	-1239.37105	-1239.20734	-1239.42785
11.15	-1237.60491	-1238.05299	-1238.74278	-1238.94138	-1238.96779	-1239.16999	-1239.17974	-1239.37105	-1239.20735	-1239.42785
11.24	-1237.60489	-1238.05294	-1238.74276	-1238.94135	-1238.96778	-1239.16998	-1239.17974	-1239.37104	-1239.20736	-1239.42785
11.34	-1237.60488	-1238.05290	-1238.74274	-1238.94133	-1238.96778	-1239.16996	-1239.17974	-1239.37104	-1239.20737	-1239.42785
11.43	-1237.60487	-1238.05286	-1238.74272	-1238.94130	-1238.96777	-1239.16995	-1239.17975	-1239.37104	-1239.20738	-1239.42785
11.53	-1237.60485	-1238.05282	-1238.74270	-1238.94128	-1238.96777	-1239.16994	-1239.17975	-1239.37104	-1239.20739	-1239.42785
11.62	-1237.60484	-1238.05278	-1238.74268	-1238.94126	-1238.96776	-1239.16992	-1239.17975	-1239.37103	-1239.20739	-1239.42784
11.72	-1237.60483	-1238.05275	-1238.74267	-1238.94124	-1238.96776	-1239.16992	-1239.17976	-1239.37104	-1239.20740	-1239.42784
11.81	-1237.60482	-1238.05272	-1238.74266	-1238.94122	-1238.96775	-1239.16991	-1239.17977	-1239.37104	-1239.20740	-1239.42784
11.91	-1237.60481	-1238.05269	-1238.74265	-1238.94121	-1238.96775	-1239.16990	-1239.17977	-1239.37104	-1239.20741	-1239.42784
12.00	-1237.60480	-1238.05266	-1238.74264	-1238.94120	-1238.96775	-1239.16989	-1239.17978	-1239.37104	-1239.20742	-1239.42784
12.09	-1237.60479	-1238.05263	-1238.74263	-1238.94119	-1238.96774	-1239.16989	-1239.17978	-1239.37105	-1239.20742	-1239.42784
12.19	-1237.60479	-1238.05261	-1238.74262	-1238.94117	-1238.96774	-1239.16988	-1239.17979	-1239.37105	-1239.20743	-1239.42784
12.28	-1237.60478	-1238.05258	-1238.74261	-1238.94116	-1238.96774	-1239.16988	-1239.17980	-1239.37105	-1239.20743	-1239.42784
12.38	-1237.60477	-1238.05256	-1238.74260	-1238.94115	-1238.96774	-1239.16987	-1239.17980	-1239.37106	-1239.20744	-1239.42784
12.47	-1237.60476	-1238.05254	-1238.74260	-1238.94114	-1238.96774	-1239.16987	-1239.17981	-1239.37106	-1239.20744	-1239.42783
12.57	-1237.60476	-1238.05252	-1238.74260	-1238.94114	-1238.96773	-1239.16986	-1239.17982	-1239.37106	-1239.20744	-1239.42783
12.66	-1237.60475	-1238.05250	-1238.74260	-1238.94113	-1238.96773	-1239.16986	-1239.17982	-1239.37107	-1239.20745	-1239.42783
12.76	-1237.60475	-1238.05248	-1238.74260	-1238.94112	-1238.96773	-1239.16985	-1239.17983	-1239.37107	-1239.20745	-1239.42783
12.85	-1237.60474	-1238.05246	-1238.74260	-1238.94111	-1238.96773	-1239.16985	-1239.17984	-1239.37108	-1239.20745	-1239.42783
12.94	-1237.60474	-1238.05244	-1238.74260	-1238.94111	-1238.96773	-1239.16984	-1239.17984	-1239.37108	-1239.20745	-1239.42782
13.04	-1237.60474	-1238.05243	-1238.74260	-1238.94110	-1238.96773	-1239.16984	-1239.17985	-1239.37108	-1239.20745	-1239.42782
13.13	-1237.60474	-1238.05241	-1238.74260	-1238.94110	-1238.96773	-1239.16984	-1239.17985	-1239.37109	-1239.20745	-1239.42782
13.23	-1237.60474	-1238.05240	-1238.74260	-1238.94109	-1238.96773	-1239.16984	-1239.17986	-1239.37109	-1239.20746	-1239.42782
13.32	-1237.60474	-1238.05238	-1238.74260	-1238.94109	-1238.96773	-1239.16983	-1239.17986	-1239.37110	-1239.20746	-1239.42782
13.42	-1237.60474	-1238.05237	-1238.74260	-1238.94108	-1238.96772	-1239.16983	-1239.17987	-1239.37110	-1239.20746	-1239.42782
13.51	-1237.60474	-1238.05235	-1238.74260	-1238.94108	-1238.96772	-1239.16983	-1239.17987	-1239.37111	-1239.20746	-1239.42782
13.61	-1237.60474	-1238.05234	-1238.74260	-1238.94108	-1238.96772	-1239.16983	-1239.17988	-1239.37111	-1239.20746	-1239.42781
13.70	-1237.60474	-1238.05233	-1238.74260	-1238.94107	-1238.96772	-1239.16982	-1239.17988	-1239.37111	-1239.20746	-1239.42781
13.80	-1237.60474	-1238.05232	-1238.74260	-1238.94107	-1238.96772	-1239.16982	-1239.17989	-1239.37112	-1239.20746	-1239.42781
13.89	-1237.60474	-1238.05231	-1238.74260	-1238.94107	-1238.96772	-1239.16982	-1239.17989	-1239.37112	-1239.20746	-1239.42781
13.98	-1237.60474	-1238.05230	-1238.74260	-1238.94106	-1238.96772	-1239.16982	-1239.17990	-1239.37112	-1239.20746	-1239.42781
14.08	-1237.60474	-1238.05229	-1238.74260	-1238.94106	-1238.96772	-1239.16982	-1239.17990	-1239.37113	-1239.20746	-1239.42781
14.17	-1237.60474	-1238.05228	-1238.74260	-1238.94106	-1238.96772	-1239.16981	-1239.17991	-1239.37113	-1239.20746	-1239.42780
14.27	-1237.60474	-1238.05227	-1238.74260	-1238.94106	-1238.96772	-1239.16981	-1239.17991	-1239.37113	-1239.20746	-1239.42780
14.36	-1237.60474	-1238.05226	-1238.74260	-1238.94106	-1238.96772	-1239.16981	-1239.17991	-1239.37114	-1239.20746	-1239.42780
14.46	-1237.60474	-1238.05225	-1238.74260	-1238.94106	-1238.96772	-1239.16981	-1239.17992	-1239.37114	-1239.20746	-1239.42780

Table S5: The absolute energies in hartree of the potential energy curve of VSi in the $^4\Pi$ state with a CASSCF wave function and nom-CPO active space.

Distance	CASSCF	CASPT2	tpbe	ftpbe	trevpbe	ftrevpbe	tblyp	ftblyp	torelyp	ftorelyp
14.55	-1237.60474	-1238.05224	-1238.74260	-1238.94105	-1238.96772	-1239.16981	-1239.17992	-1239.37114	-1239.20746	-1239.42780
14.65	-1237.60474	-1238.05224	-1238.74260	-1238.94105	-1238.96772	-1239.16981	-1239.17992	-1239.37115	-1239.20746	-1239.42780
14.74	-1237.60474	-1238.05223	-1238.74260	-1238.94105	-1238.96771	-1239.16981	-1239.17993	-1239.37115	-1239.20746	-1239.42780
14.83	-1237.60474	-1238.05222	-1238.74260	-1238.94105	-1238.96772	-1239.16981	-1239.17993	-1239.37115	-1239.20747	-1239.42780
14.93	-1237.60474	-1238.05222	-1238.74260	-1238.94105	-1238.96771	-1239.16981	-1239.17993	-1239.37116	-1239.20747	-1239.42780
15.02	-1237.60474	-1238.05221	-1238.74260	-1238.94105	-1238.96771	-1239.16981	-1239.17994	-1239.37116	-1239.20747	-1239.42780
15.12	-1237.60474	-1238.05221	-1238.74260	-1238.94105	-1238.96771	-1239.16981	-1239.17994	-1239.37116	-1239.20747	-1239.42780
15.21	-1237.60474	-1238.05220	-1238.74260	-1238.94105	-1238.96771	-1239.16980	-1239.17994	-1239.37116	-1239.20747	-1239.42780
15.31	-1237.60474	-1238.05219	-1238.74260	-1238.94105	-1238.96771	-1239.16980	-1239.17994	-1239.37116	-1239.20747	-1239.42780
15.40	-1237.60474	-1238.05216	-1238.74260	-1238.94105	-1238.96771	-1239.16980	-1239.17995	-1239.37117	-1239.20747	-1239.42780
15.50	-1237.60474	-1238.05216	-1238.74260	-1238.94105	-1238.96771	-1239.16980	-1239.17995	-1239.37117	-1239.20747	-1239.42780
15.59	-1237.60474	-1238.05215	-1238.74260	-1238.94105	-1238.96771	-1239.16980	-1239.17995	-1239.37117	-1239.20747	-1239.42780
15.68	-1237.60474	-1238.05217	-1238.74260	-1238.94104	-1238.96771	-1239.16980	-1239.17995	-1239.37117	-1239.20747	-1239.42780
15.78	-1237.60474	-1238.05217	-1238.74260	-1238.94104	-1238.96771	-1239.16980	-1239.17996	-1239.37117	-1239.20747	-1239.42780
15.87	-1237.60474	-1238.05217	-1238.74260	-1238.94104	-1238.96771	-1239.16980	-1239.17996	-1239.37118	-1239.20747	-1239.42780
15.97	-1237.60474	-1238.05216	-1238.74260	-1238.94104	-1238.96771	-1239.16980	-1239.17996	-1239.37118	-1239.20747	-1239.42780
16.06	-1237.60474	-1238.05216	-1238.74260	-1238.94104	-1238.96771	-1239.16980	-1239.17996	-1239.37118	-1239.20747	-1239.42780
16.16	-1237.60474	-1238.05213	-1238.74260	-1238.94104	-1238.96771	-1239.16980	-1239.17996	-1239.37118	-1239.20747	-1239.42780
16.25	-1237.60474	-1238.05212	-1238.74260	-1238.94104	-1238.96771	-1239.16980	-1239.17996	-1239.37118	-1239.20747	-1239.42780
16.35	-1237.60474	-1238.05212	-1238.74260	-1238.94104	-1238.96771	-1239.16980	-1239.17997	-1239.37118	-1239.20747	-1239.42780
16.44	-1237.60474	-1238.05212	-1238.74260	-1238.94104	-1238.96771	-1239.16980	-1239.17997	-1239.37118	-1239.20747	-1239.42780
16.54	-1237.60474	-1238.05211	-1238.74260	-1238.94104	-1238.96771	-1239.16980	-1239.17997	-1239.37118	-1239.20747	-1239.42780
16.63	-1237.60474	-1238.05211	-1238.74260	-1238.94104	-1238.96771	-1239.16980	-1239.17997	-1239.37118	-1239.20747	-1239.42779
16.72	-1237.60474	-1238.05211	-1238.74260	-1238.94104	-1238.96771	-1239.16980	-1239.17997	-1239.37119	-1239.20747	-1239.42779
16.82	-1237.60474	-1238.05210	-1238.74260	-1238.94104	-1238.96771	-1239.16980	-1239.17997	-1239.37119	-1239.20747	-1239.42779
16.91	-1237.60474	-1238.05210	-1238.74260	-1238.94104	-1238.96771	-1239.16980	-1239.17997	-1239.37119	-1239.20747	-1239.42779
17.01	-1237.60474	-1238.05210	-1238.74260	-1238.94104	-1238.96771	-1239.16980	-1239.17997	-1239.37119	-1239.20747	-1239.42779
17.10	-1237.60474	-1238.05209	-1238.74260	-1238.94104	-1238.96771	-1239.16980	-1239.17997	-1239.37119	-1239.20747	-1239.42779
17.20	-1237.60474	-1238.05209	-1238.74260	-1238.94104	-1238.96771	-1239.16980	-1239.17997	-1239.37119	-1239.20747	-1239.42779
17.29	-1237.60474	-1238.05209	-1238.74260	-1238.94104	-1238.96771	-1239.16980	-1239.17998	-1239.37119	-1239.20747	-1239.42779
17.39	-1237.60474	-1238.05209	-1238.74260	-1238.94104	-1238.96771	-1239.16980	-1239.17998	-1239.37119	-1239.20747	-1239.42779
17.48	-1237.60474	-1238.05208	-1238.74260	-1238.94104	-1238.96771	-1239.16980	-1239.17998	-1239.37119	-1239.20747	-1239.42779
17.57	-1237.60474	-1238.05208	-1238.74260	-1238.94104	-1238.96771	-1239.16980	-1239.17998	-1239.37119	-1239.20747	-1239.42779
17.67	-1237.60474	-1238.05208	-1238.74260	-1238.94104	-1238.96771	-1239.16980	-1239.17998	-1239.37119	-1239.20747	-1239.42779
17.76	-1237.60474	-1238.05208	-1238.74260	-1238.94104	-1238.96771	-1239.16980	-1239.17998	-1239.37119	-1239.20747	-1239.42779
17.86	-1237.60474	-1238.05208	-1238.74260	-1238.94104	-1238.96771	-1239.16980	-1239.17998	-1239.37120	-1239.20747	-1239.42779
17.95	-1237.60474	-1238.05207	-1238.74260	-1238.94104	-1238.96771	-1239.16980	-1239.17998	-1239.37120	-1239.20747	-1239.42779
18.05	-1237.60474	-1238.05207	-1238.74260	-1238.94104	-1238.96771	-1239.16980	-1239.17998	-1239.37120	-1239.20747	-1239.42779

Table S6: The absolute energies in hartree of the potential energy curve of VSi in the $^2\Sigma^+$ state with a CASSCF wave function and nom-CPO active space.

Distance	CASSCF	CASPT2	tpbe	ftpbe	trevpbe	ftrevpbe	tblyp	ftblyp	torelyp	ftorelyp
1.98	-1235.49529	-1236.01615	-1236.72563	-1236.93499	-1236.93327	-1237.14354	-1237.14111	-1237.34831	-1237.20454	-1237.43541
2.08	-1235.93159	-1236.43703	-1237.16527	-1237.37080	-1237.37370	-1237.58010	-1237.58032	-1237.78459	-1237.63990	-1237.86694
2.17	-1236.27308	-1236.76397	-1237.50138	-1237.70561	-1237.71080	-1237.91599	-1237.91652	-1238.11988	-1237.97379	-1238.19971
2.27	-1236.54063	-1237.01856	-1237.76407	-1237.96727	-1237.97444	-1238.17874	-1238.17941	-1238.38196	-1238.23483	-1238.45991
2.36	-1236.75126	-1237.21848	-1237.97037	-1238.17265	-1238.18165	-1238.38517	-1238.38605	-1238.58781	-1238.43965	-1238.66405
2.46	-1236.91803	-1237.42319	-1238.13325	-1238.33493	-1238.34539	-1238.54851	-1238.54933	-1238.75061	-1238.60114	-1238.82536
2.55	-1237.04723	-1237.55492	-1238.26352	-1238.46480	-1238.47649	-1238.67934	-1238.67998	-1238.88098	-1238.73039	-1238.95449
2.65	-1237.15901	-1237.66100	-1238.36835	-1238.56947	-1238.58217	-1238.78503	-1238.78508	-1238.98603	-1238.83443	-1239.05874

Table S6: The absolute energies in hartree of the potential energy curve of VSi in the $^2\Sigma^+$ state with a CASSCF wave function and nom-CPO active space.

Distance	CASSCF	CASPT2	tpbe	ftpbe	trevpbe	ftrevpbe	tblyp	ftblyp	torelyp	ftorelyp
2.74	-1237.24683	-1237.74759	-1238.45419	-1238.65516	-1238.66888	-1238.87169	-1238.87110	-1239.07192	-1238.91978	-1239.14412
2.83	-1237.31837	-1237.81907	-1238.52463	-1238.72532	-1238.74039	-1238.94296	-1238.94128	-1239.14191	-1238.99051	-1239.21462
2.93	-1237.37935	-1237.87862	-1238.58512	-1238.78587	-1238.80178	-1239.00440	-1239.00171	-1239.20221	-1239.05095	-1239.27492
3.02	-1237.43021	-1237.92868	-1238.63611	-1238.83684	-1238.85363	-1239.05613	-1239.05265	-1239.25285	-1239.10198	-1239.32554
3.12	-1237.47285	-1237.97041	-1238.67887	-1238.87953	-1238.89723	-1239.09954	-1239.09529	-1239.29502	-1239.14477	-1239.36763
3.21	-1237.50870	-1238.00491	-1238.71426	-1238.91483	-1238.93335	-1239.13542	-1239.13065	-1239.32968	-1239.17994	-1239.40191
3.31	-1237.53870	-1238.03336	-1238.74318	-1238.94363	-1238.96279	-1239.16464	-1239.15972	-1239.35800	-1239.20835	-1239.42952
3.40	-1237.56353	-1238.05668	-1238.76651	-1238.96692	-1238.98650	-1239.18830	-1239.18339	-1239.38115	-1239.23103	-1239.45179
3.50	-1237.58387	-1238.07556	-1238.78514	-1238.98552	-1239.00542	-1239.20723	-1239.20247	-1239.39988	-1239.24897	-1239.46951
3.59	-1237.60035	-1238.09064	-1238.79984	-1239.00017	-1239.02035	-1239.22220	-1239.21769	-1239.41480	-1239.26297	-1239.48337
3.68	-1237.61355	-1238.10249	-1238.81123	-1239.01151	-1239.03192	-1239.23382	-1239.22965	-1239.42647	-1239.27367	-1239.49397
3.78	-1237.62399	-1238.11162	-1238.81983	-1239.02005	-1239.04067	-1239.24265	-1239.23884	-1239.43536	-1239.28157	-1239.50181
3.87	-1237.63215	-1238.11847	-1238.82606	-1239.02623	-1239.04702	-1239.24911	-1239.24566	-1239.44189	-1239.28711	-1239.50733
3.97	-1237.63846	-1238.12340	-1238.83027	-1239.03043	-1239.05133	-1239.25358	-1239.25045	-1239.44639	-1239.29063	-1239.51088
4.06	-1237.64327	-1238.12676	-1238.83277	-1239.03296	-1239.05391	-1239.25641	-1239.25352	-1239.44918	-1239.29244	-1239.51280
4.16	-1237.64692	-1238.12880	-1238.83383	-1239.03410	-1239.05503	-1239.25787	-1239.25511	-1239.45051	-1239.29281	-1239.51337
4.35	-1237.65166	-1238.12977	-1238.83257	-1239.03313	-1239.05389	-1239.25759	-1239.25474	-1239.44973	-1239.29030	-1239.51152
4.44	-1237.65312	-1238.12987	-1238.83040	-1239.03116	-1239.05179	-1239.25600	-1239.25292	-1239.44775	-1239.28760	-1239.50927
4.54	-1237.65417	-1238.12924	-1238.82700	-1239.02797	-1239.04847	-1239.25321	-1239.24979	-1239.44448	-1239.28373	-1239.50593
4.63	-1237.65494	-1238.12788	-1238.82301	-1239.02408	-1239.04457	-1239.24971	-1239.24604	-1239.44049	-1239.27933	-1239.50196
4.72	-1237.65543	-1238.12579	-1238.81960	-1239.02064	-1239.04128	-1239.24658	-1239.24286	-1239.43701	-1239.27568	-1239.49844
4.82	-1237.65558	-1238.12358	-1238.81678	-1239.01776	-1239.03860	-1239.24395	-1239.24028	-1239.43415	-1239.27276	-1239.49553
4.91	-1237.65536	-1238.12143	-1238.81418	-1239.01507	-1239.03614	-1239.24149	-1239.23791	-1239.43152	-1239.27012	-1239.49285
5.01	-1237.65482	-1238.11921	-1238.81162	-1239.01242	-1239.03372	-1239.23905	-1239.23561	-1239.42898	-1239.26759	-1239.49024
5.10	-1237.65397	-1238.11685	-1238.80907	-1239.00979	-1239.03131	-1239.23660	-1239.23333	-1239.42648	-1239.26511	-1239.48767
5.20	-1237.65287	-1238.11436	-1238.80651	-1239.00714	-1239.02889	-1239.23413	-1239.23105	-1239.42400	-1239.26265	-1239.48512
5.29	-1237.65156	-1238.11177	-1238.80395	-1239.00449	-1239.02647	-1239.23163	-1239.22878	-1239.42154	-1239.26022	-1239.48257
5.39	-1237.65006	-1238.10909	-1238.80140	-1239.00183	-1239.02404	-1239.22911	-1239.22652	-1239.41909	-1239.25781	-1239.48004
5.48	-1237.64843	-1238.10636	-1238.79886	-1238.99919	-1239.02162	-1239.22659	-1239.22429	-1239.41668	-1239.25542	-1239.47753
5.57	-1237.64669	-1238.10359	-1238.79635	-1238.99658	-1239.01922	-1239.22409	-1239.22210	-1239.41432	-1239.25308	-1239.47506
5.67	-1237.64487	-1238.10081	-1238.79388	-1238.99401	-1239.01686	-1239.22161	-1239.21996	-1239.41201	-1239.25079	-1239.47265
5.76	-1237.64300	-1238.09803	-1238.79146	-1238.99149	-1239.01454	-1239.21917	-1239.21788	-1239.40978	-1239.24856	-1239.47029
5.86	-1237.64110	-1238.09528	-1238.78910	-1238.98902	-1239.01227	-1239.21678	-1239.21586	-1239.40762	-1239.24639	-1239.46800
5.95	-1237.63919	-1238.09257	-1238.78680	-1238.98663	-1239.01006	-1239.21444	-1239.21392	-1239.40556	-1239.24430	-1239.46579
6.05	-1237.63729	-1238.08993	-1238.78457	-1238.98431	-1239.00792	-1239.21217	-1239.21207	-1239.40359	-1239.24228	-1239.46367
6.14	-1237.63542	-1238.08735	-1238.78241	-1238.98208	-1239.00584	-1239.20998	-1239.21030	-1239.40174	-1239.24033	-1239.46164
6.24	-1237.63360	-1238.08483	-1238.78032	-1238.97993	-1239.00382	-1239.20785	-1239.20862	-1239.39999	-1239.23847	-1239.45970
6.33	-1237.63184	-1238.08241	-1238.77830	-1238.97786	-1239.00187	-1239.20581	-1239.20703	-1239.39836	-1239.23668	-1239.45786
6.43	-1237.63015	-1238.08008	-1238.77635	-1238.97587	-1238.99998	-1239.20384	-1239.20552	-1239.39683	-1239.23496	-1239.45612
6.52	-1237.62855	-1238.07786	-1238.77447	-1238.97397	-1238.99816	-1239.20194	-1239.20408	-1239.39540	-1239.23332	-1239.45446
6.61	-1237.62703	-1238.07575	-1238.77264	-1238.97213	-1238.99639	-1239.20012	-1239.20271	-1239.39407	-1239.23174	-1239.45289
6.71	-1237.62560	-1238.07375	-1238.77087	-1238.97036	-1238.99468	-1239.19835	-1239.20141	-1239.39282	-1239.23022	-1239.45139
6.80	-1237.62428	-1238.07188	-1238.76915	-1238.96865	-1238.99301	-1239.19665	-1239.20015	-1239.39164	-1239.22875	-1239.44995
6.90	-1237.62305	-1238.07012	-1238.76748	-1238.96700	-1238.99140	-1239.19501	-1239.19894	-1239.39052	-1239.22734	-1239.44858
6.99	-1237.62192	-1238.06849	-1238.76587	-1238.96541	-1238.98984	-1239.19344	-1239.19778	-1239.38945	-1239.22598	-1239.44726
7.09	-1237.62088	-1238.06697	-1238.76432	-1238.96389	-1238.98834	-1239.19193	-1239.19666	-1239.38844	-1239.22468	-1239.44601
7.18	-1237.61993	-1238.06556	-1238.76283	-1238.96242	-1238.98690	-1239.19048	-1239.19559	-1239.38748	-1239.22344	-1239.44481
7.28	-1237.61906	-1238.06426	-1238.76140	-1238.96103	-1238.98554	-1239.18911	-1239.19456	-1239.38656	-1239.22227	-1239.44367
7.37	-1237.61828	-1238.06306	-1238.76005	-1238.95970	-1238.98424	-1239.18780	-1239.19358	-1239.38568	-1239.22116	-1239.44260
7.46	-1237.61756	-1238.06195	-1238.75876	-1238.95844	-1238.98301	-1239.18657	-1239.19265	-1239.38485	-1239.22013	-1239.44159
7.56	-1237.61692	-1238.06094	-1238.75756	-1238.95725	-1238.98186	-1239.18542	-1239.19178	-1239.38407	-1239.21916	-1239.44064
7.65	-1237.61633	-1238.06002	-1238.75642	-1238.95614	-1238.98079	-1239.18434	-1239.19095	-1239.38333	-1239.21827	-1239.43976

Table S6: The absolute energies in hartree of the potential energy curve of VSi in the $^2\Sigma^+$ state with a CASSCF wave function and nom-CPO active space.

Distance	CASSCF	CASPT2	tpbe	ftpbe	trevpbe	ftrevpbe	tblyp	ftblyp	torelyp	ftorelyp
7.75	-1237.61580	-1238.05917	-1238.75537	-1238.95510	-1238.97980	-1239.18334	-1239.19018	-1239.38265	-1239.21745	-1239.43894
7.84	-1237.61532	-1238.05839	-1238.75439	-1238.95414	-1238.97888	-1239.18242	-1239.18947	-1239.38201	-1239.21670	-1239.43820
7.94	-1237.61489	-1238.05768	-1238.75348	-1238.95325	-1238.97804	-1239.18157	-1239.18880	-1239.38142	-1239.21602	-1239.43751
8.03	-1237.61450	-1238.05703	-1238.75265	-1238.95242	-1238.97726	-1239.18078	-1239.18819	-1239.38087	-1239.21540	-1239.43689
8.13	-1237.61415	-1238.05643	-1238.75188	-1238.95166	-1238.97655	-1239.18007	-1239.18762	-1239.38037	-1239.21484	-1239.43632
8.22	-1237.61383	-1238.05589	-1238.75117	-1238.95096	-1238.97590	-1239.17941	-1239.18711	-1239.37991	-1239.21434	-1239.43581
8.31	-1237.61354	-1238.05539	-1238.75053	-1238.95032	-1238.97532	-1239.17882	-1239.18664	-1239.37949	-1239.21390	-1239.43536
8.41	-1237.61327	-1238.05493	-1238.74994	-1238.94974	-1238.97479	-1239.17828	-1239.18621	-1239.37911	-1239.21351	-1239.43495
8.50	-1237.61304	-1238.05451	-1238.74940	-1238.94920	-1238.97431	-1239.17779	-1239.18582	-1239.37876	-1239.21316	-1239.43459
8.60	-1237.61282	-1238.05413	-1238.74892	-1238.94872	-1238.97388	-1239.17735	-1239.18547	-1239.37844	-1239.21285	-1239.43426
8.69	-1237.61263	-1238.05378	-1238.74847	-1238.94828	-1238.97350	-1239.17695	-1239.18515	-1239.37815	-1239.21258	-1239.43398
8.79	-1237.61245	-1238.05346	-1238.74807	-1238.94787	-1238.97315	-1239.17660	-1239.18486	-1239.37788	-1239.21235	-1239.43372
8.88	-1237.61229	-1238.05317	-1238.74771	-1238.94751	-1238.97284	-1239.17627	-1239.18460	-1239.37764	-1239.21214	-1239.43350
8.98	-1237.61214	-1238.05290	-1238.74738	-1238.94718	-1238.97256	-1239.17599	-1239.18437	-1239.37741	-1239.21197	-1239.43331
9.07	-1237.61201	-1238.05265	-1238.74709	-1238.94688	-1238.97232	-1239.17573	-1239.18416	-1239.37720	-1239.21182	-1239.43315
9.17	-1237.61189	-1238.05243	-1238.74682	-1238.94661	-1238.97210	-1239.17550	-1239.18398	-1239.37702	-1239.21169	-1239.43300
9.26	-1237.61178	-1238.05222	-1238.74658	-1238.94637	-1238.97190	-1239.17530	-1239.18382	-1239.37687	-1239.21159	-1239.43288
9.35	-1237.61168	-1238.05203	-1238.74637	-1238.94615	-1238.97173	-1239.17512	-1239.18367	-1239.37673	-1239.21150	-1239.43278
9.45	-1237.61159	-1238.05185	-1238.74617	-1238.94595	-1238.97158	-1239.17495	-1239.18354	-1239.37661	-1239.21142	-1239.43269
9.54	-1237.61150	-1238.05169	-1238.74599	-1238.94577	-1238.97144	-1239.17481	-1239.18343	-1239.37650	-1239.21136	-1239.43261
9.64	-1237.61142	-1238.05154	-1238.74583	-1238.94561	-1238.97132	-1239.17468	-1239.18332	-1239.37640	-1239.21131	-1239.43254
9.73	-1237.61135	-1238.05141	-1238.74569	-1238.94546	-1238.97121	-1239.17456	-1239.18323	-1239.37631	-1239.21126	-1239.43249
9.83	-1237.61129	-1238.05128	-1238.74556	-1238.94532	-1238.97112	-1239.17446	-1239.18315	-1239.37623	-1239.21123	-1239.43244
9.92	-1237.61123	-1238.05117	-1238.74544	-1238.94520	-1238.97104	-1239.17437	-1239.18308	-1239.37617	-1239.21121	-1239.43240
10.02	-1237.61118	-1238.05106	-1238.74534	-1238.94509	-1238.97097	-1239.17429	-1239.18302	-1239.37611	-1239.21119	-1239.43237
10.11	-1237.61113	-1238.05097	-1238.74524	-1238.94499	-1238.97090	-1239.17422	-1239.18297	-1239.37606	-1239.21117	-1239.43235
10.20	-1237.61108	-1238.05089	-1238.74516	-1238.94490	-1238.97085	-1239.17415	-1239.18292	-1239.37601	-1239.21116	-1239.43232
10.30	-1237.61104	-1238.05081	-1238.74508	-1238.94482	-1238.97080	-1239.17410	-1239.18288	-1239.37597	-1239.21116	-1239.43230
10.39	-1237.61100	-1238.05075	-1238.74501	-1238.94475	-1238.97075	-1239.17404	-1239.18285	-1239.37593	-1239.21115	-1239.43229
10.49	-1237.61097	-1238.05070	-1238.74494	-1238.94468	-1238.97071	-1239.17400	-1239.18282	-1239.37590	-1239.21115	-1239.43228
10.58	-1237.61094	-1238.05065	-1238.74488	-1238.94462	-1238.97068	-1239.17395	-1239.18279	-1239.37587	-1239.21115	-1239.43227
10.68	-1237.61091	-1238.05062	-1238.74483	-1238.94456	-1238.97065	-1239.17392	-1239.18277	-1239.37585	-1239.21115	-1239.43226
10.77	-1237.61088	-1238.05060	-1238.74479	-1238.94451	-1238.97062	-1239.17389	-1239.18276	-1239.37583	-1239.21116	-1239.43225
10.87	-1237.61085	-1238.05059	-1238.74474	-1238.94446	-1238.97060	-1239.17386	-1239.18274	-1239.37581	-1239.21116	-1239.43225
10.96	-1237.61083	-1238.05059	-1238.74471	-1238.94442	-1238.97058	-1239.17383	-1239.18273	-1239.37580	-1239.21116	-1239.43225
11.05	-1237.61081	-1238.05061	-1238.74467	-1238.94439	-1238.97056	-1239.17381	-1239.18272	-1239.37579	-1239.21117	-1239.43225
11.15	-1237.61079	-1238.05063	-1238.74464	-1238.94435	-1238.97055	-1239.17379	-1239.18271	-1239.37578	-1239.21118	-1239.43224
11.24	-1237.61077	-1238.05067	-1238.74461	-1238.94432	-1238.97053	-1239.17377	-1239.18271	-1239.37578	-1239.21118	-1239.43224
11.34	-1237.61075	-1238.05071	-1238.74458	-1238.94429	-1238.97052	-1239.17375	-1239.18270	-1239.37577	-1239.21119	-1239.43224
11.43	-1237.61073	-1238.05073	-1238.74456	-1238.94426	-1238.97051	-1239.17374	-1239.18270	-1239.37576	-1239.21119	-1239.43224
11.53	-1237.61072	-1238.05073	-1238.74453	-1238.94423	-1238.97050	-1239.17372	-1239.18269	-1239.37575	-1239.21119	-1239.43223
11.62	-1237.61071	-1238.05072	-1238.74451	-1238.94421	-1238.97049	-1239.17371	-1239.18270	-1239.37575	-1239.21120	-1239.43223
11.72	-1237.61069	-1238.05067	-1238.74450	-1238.94419	-1238.97048	-1239.17370	-1239.18270	-1239.37576	-1239.21121	-1239.43224
11.81	-1237.61068	-1238.05059	-1238.74448	-1238.94417	-1238.97048	-1239.17369	-1239.18270	-1239.37576	-1239.21122	-1239.43224
11.91	-1237.61067	-1238.05044	-1238.74446	-1238.94415	-1238.97047	-1239.17367	-1239.18270	-1239.37575	-1239.21122	-1239.43223
12.00	-1237.61066	-1238.05025	-1238.74445	-1238.94414	-1238.97046	-1239.17366	-1239.18270	-1239.37575	-1239.21122	-1239.43223
12.09	-1237.61065	-1238.05003	-1238.74443	-1238.94412	-1238.97046	-1239.17366	-1239.18271	-1239.37576	-1239.21123	-1239.43223
12.19	-1237.61064	-1238.04983	-1238.74442	-1238.94411	-1238.97045	-1239.17365	-1239.18271	-1239.37576	-1239.21123	-1239.43223
12.28	-1237.61063	-1238.04967	-1238.74441	-1238.94410	-1238.97045	-1239.17364	-1239.18271	-1239.37576	-1239.21123	-1239.43223
12.38	-1237.61062	-1238.04954	-1238.74440	-1238.94408	-1238.97044	-1239.17364	-1239.18272	-1239.37576	-1239.21124	-1239.43223
12.47	-1237.61061	-1238.04944	-1238.74439	-1238.94407	-1238.97044	-1239.17363	-1239.18272	-1239.37577	-1239.21124	-1239.43223
12.57	-1237.61061	-1238.04937	-1238.74438	-1238.94406	-1238.97044	-1239.17362	-1239.18273	-1239.37577	-1239.21124	-1239.43222

Table S6: The absolute energies in hartree of the potential energy curve of VSi in the $^2\Sigma^+$ state with a CASSCF wave function and nom-CPO active space.

Distance	CASSCF	CASPT2	tpbe	ftpbe	trevpbe	ftrevpbe	tblyp	ftblyp	torelyp	ftorelyp
12.66	-1237.61060	-1238.04930	-1238.74437	-1238.94405	-1238.97043	-1239.17362	-1239.18273	-1239.37577	-1239.21124	-1239.43222
12.76	-1237.61059	-1238.04926	-1238.74436	-1238.94404	-1238.97043	-1239.17361	-1239.18274	-1239.37577	-1239.21124	-1239.43222
12.85	-1237.61059	-1238.04921	-1238.74435	-1238.94403	-1238.97043	-1239.17361	-1239.18274	-1239.37578	-1239.21124	-1239.43222
12.94	-1237.61058	-1238.04918	-1238.74435	-1238.94403	-1238.97042	-1239.17360	-1239.18275	-1239.37578	-1239.21125	-1239.43222
13.04	-1237.61058	-1238.04915	-1238.74434	-1238.94402	-1238.97042	-1239.17360	-1239.18275	-1239.37579	-1239.21125	-1239.43222
13.13	-1237.61057	-1238.04913	-1238.74434	-1238.94402	-1238.97042	-1239.17360	-1239.18276	-1239.37579	-1239.21125	-1239.43222
13.23	-1237.61056	-1238.04911	-1238.74433	-1238.94401	-1238.97042	-1239.17360	-1239.18276	-1239.37580	-1239.21125	-1239.43222
13.32	-1237.61056	-1238.04909	-1238.74433	-1238.94401	-1238.97042	-1239.17359	-1239.18277	-1239.37580	-1239.21125	-1239.43222
13.42	-1237.61056	-1238.04907	-1238.74432	-1238.94400	-1238.97041	-1239.17359	-1239.18277	-1239.37581	-1239.21125	-1239.43222
13.51	-1237.61055	-1238.04847	-1238.74432	-1238.94400	-1238.97041	-1239.17359	-1239.18278	-1239.37581	-1239.21125	-1239.43222
13.61	-1237.61055	-1238.04845	-1238.74431	-1238.94399	-1238.97041	-1239.17358	-1239.18278	-1239.37581	-1239.21125	-1239.43222
13.70	-1237.61054	-1238.04843	-1238.74431	-1238.94399	-1238.97041	-1239.17358	-1239.18278	-1239.37581	-1239.21125	-1239.43221
13.80	-1237.61054	-1238.04842	-1238.74431	-1238.94398	-1238.97041	-1239.17358	-1239.18279	-1239.37582	-1239.21125	-1239.43221
13.89	-1237.61054	-1238.04841	-1238.74430	-1238.94398	-1238.97040	-1239.17358	-1239.18279	-1239.37582	-1239.21125	-1239.43221
13.98	-1237.61053	-1238.04839	-1238.74430	-1238.94397	-1238.97040	-1239.17358	-1239.18279	-1239.37582	-1239.21125	-1239.43221
14.08	-1237.61053	-1238.04838	-1238.74430	-1238.94397	-1238.97040	-1239.17358	-1239.18280	-1239.37582	-1239.21125	-1239.43221
14.17	-1237.61053	-1238.04837	-1238.74429	-1238.94397	-1238.97040	-1239.17358	-1239.18280	-1239.37583	-1239.21125	-1239.43221
14.27	-1237.61052	-1238.04836	-1238.74429	-1238.94397	-1238.97040	-1239.17358	-1239.18280	-1239.37583	-1239.21125	-1239.43221
14.36	-1237.61052	-1238.04835	-1238.74429	-1238.94396	-1238.97040	-1239.17358	-1239.18281	-1239.37583	-1239.21125	-1239.43221
14.46	-1237.61052	-1238.04834	-1238.74429	-1238.94396	-1238.97039	-1239.17358	-1239.18281	-1239.37584	-1239.21125	-1239.43221
14.55	-1237.61051	-1238.04834	-1238.74429	-1238.94396	-1238.97039	-1239.17358	-1239.18281	-1239.37584	-1239.21125	-1239.43221
14.65	-1237.61051	-1238.04833	-1238.74428	-1238.94396	-1238.97039	-1239.17358	-1239.18282	-1239.37584	-1239.21126	-1239.43221
14.74	-1237.61051	-1238.04832	-1238.74428	-1238.94396	-1238.97039	-1239.17358	-1239.18282	-1239.37585	-1239.21126	-1239.43221
14.83	-1237.61051	-1238.04831	-1238.74428	-1238.94396	-1238.97039	-1239.17358	-1239.18282	-1239.37585	-1239.21126	-1239.43221
14.93	-1237.61050	-1238.04831	-1238.74428	-1238.94395	-1238.97039	-1239.17358	-1239.18282	-1239.37585	-1239.21125	-1239.43221
15.02	-1237.61050	-1238.04830	-1238.74428	-1238.94395	-1238.97039	-1239.17358	-1239.18283	-1239.37585	-1239.21125	-1239.43221
15.12	-1237.61050	-1238.04829	-1238.74427	-1238.94395	-1238.97039	-1239.17358	-1239.18283	-1239.37586	-1239.21125	-1239.43221
15.21	-1237.61050	-1238.04829	-1238.74427	-1238.94395	-1238.97038	-1239.17358	-1239.18283	-1239.37586	-1239.21125	-1239.43221
15.31	-1237.61050	-1238.04827	-1238.74427	-1238.94395	-1238.97038	-1239.17358	-1239.18283	-1239.37586	-1239.21125	-1239.43220
15.40	-1237.61049	-1238.04826	-1238.74427	-1238.94395	-1238.97038	-1239.17358	-1239.18283	-1239.37586	-1239.21125	-1239.43220
15.50	-1237.61049	-1238.04826	-1238.74427	-1238.94395	-1238.97038	-1239.17358	-1239.18284	-1239.37587	-1239.21125	-1239.43221
15.59	-1237.61049	-1238.04825	-1238.74427	-1238.94395	-1238.97038	-1239.17358	-1239.18284	-1239.37587	-1239.21126	-1239.43221
15.68	-1237.61049	-1238.04825	-1238.74427	-1238.94395	-1238.97038	-1239.17358	-1239.18284	-1239.37587	-1239.21126	-1239.43221
15.78	-1237.61049	-1238.04824	-1238.74427	-1238.94394	-1238.97038	-1239.17358	-1239.18285	-1239.37587	-1239.21126	-1239.43221
15.87	-1237.61049	-1238.04824	-1238.74427	-1238.94394	-1238.97038	-1239.17358	-1239.18285	-1239.37587	-1239.21126	-1239.43221
15.97	-1237.61048	-1238.04821	-1238.74427	-1238.94394	-1238.97038	-1239.17358	-1239.18285	-1239.37588	-1239.21126	-1239.43221
16.06	-1237.61048	-1238.04821	-1238.74427	-1238.94394	-1238.97038	-1239.17358	-1239.18285	-1239.37588	-1239.21126	-1239.43221
16.16	-1237.61048	-1238.04821	-1238.74427	-1238.94394	-1238.97038	-1239.17358	-1239.18285	-1239.37588	-1239.21126	-1239.43221
16.25	-1237.61048	-1238.04821	-1238.74426	-1238.94394	-1238.97038	-1239.17358	-1239.18285	-1239.37588	-1239.21126	-1239.43221
16.35	-1237.61048	-1238.04821	-1238.74426	-1238.94394	-1238.97038	-1239.17358	-1239.18285	-1239.37588	-1239.21126	-1239.43221
16.44	-1237.61048	-1238.04821	-1238.74426	-1238.94394	-1238.97038	-1239.17358	-1239.18286	-1239.37588	-1239.21126	-1239.43221
16.54	-1237.61048	-1238.04821	-1238.74426	-1238.94394	-1238.97038	-1239.17358	-1239.18286	-1239.37588	-1239.21125	-1239.43221
16.63	-1237.61047	-1238.04821	-1238.74426	-1238.94394	-1238.97038	-1239.17358	-1239.18286	-1239.37588	-1239.21125	-1239.43221
16.72	-1237.61047	-1238.04821	-1238.74426	-1238.94394	-1238.97038	-1239.17358	-1239.18286	-1239.37588	-1239.21125	-1239.43221
16.82	-1237.61047	-1238.04821	-1238.74426	-1238.94394	-1238.97037	-1239.17358	-1239.18286	-1239.37589	-1239.21125	-1239.43221
16.91	-1237.61047	-1238.04821	-1238.74426	-1238.94394	-1238.97037	-1239.17358	-1239.18286	-1239.37589	-1239.21125	-1239.43221
17.01	-1237.61047	-1238.04821	-1238.74426	-1238.94394	-1238.97037	-1239.17358	-1239.18286	-1239.37589	-1239.21125	-1239.43221
17.10	-1237.61047	-1238.04821	-1238.74426	-1238.94394	-1238.97037	-1239.17358	-1239.18286	-1239.37589	-1239.21125	-1239.43221
17.20	-1237.61047	-1238.04821	-1238.74426	-1238.94394	-1238.97037	-1239.17358	-1239.18286	-1239.37589	-1239.21125	-1239.43221
17.29	-1237.61047	-1238.04821	-1238.74426	-1238.94394	-1238.97037	-1239.17358	-1239.18286	-1239.37589	-1239.21125	-1239.43221
17.39	-1237.61047	-1238.04821	-1238.74426	-1238.94394	-1238.97037	-1239.17358	-1239.18286	-1239.37589	-1239.21125	-1239.43221
17.48	-1237.61046	-1238.04821	-1238.74426	-1238.94394	-1238.97037	-1239.17358	-1239.18286	-1239.37589	-1239.21125	-1239.43221

Table S6: The absolute energies in hartree of the potential energy curve of VSi in the $^2\Sigma^+$ state with a CASSCF wave function and nom-CPO active space.

Distance	CASSCF	CASPT2	tpbe	ftpbe	trevpbe	ftrevpbe	tblyp	ftblyp	torelyp	ftorelyp
17.57	-1237.61046	-1238.04821	-1238.74426	-1238.94394	-1238.97037	-1239.17358	-1239.18287	-1239.37589	-1239.21125	-1239.43221
17.67	-1237.61046	-1238.04821	-1238.74426	-1238.94394	-1238.97037	-1239.17358	-1239.18287	-1239.37589	-1239.21125	-1239.43221
17.76	-1237.61046	-1238.04821	-1238.74426	-1238.94394	-1238.97037	-1239.17358	-1239.18287	-1239.37590	-1239.21125	-1239.43221
17.86	-1237.61046	-1238.04821	-1238.74426	-1238.94394	-1238.97037	-1239.17358	-1239.18287	-1239.37590	-1239.21125	-1239.43221
17.95	-1237.61046	-1238.04821	-1238.74426	-1238.94393	-1238.97037	-1239.17358	-1239.18287	-1239.37590	-1239.21125	-1239.43221
18.05	-1237.61046	-1238.04821	-1238.74426	-1238.94393	-1238.97037	-1239.17358	-1239.18287	-1239.37590	-1239.21125	-1239.43221

Table S7: The absolute energies in hartree of the potential energy curve of VSi in the $^2\Delta$ state with a CASSCF wave function and nom-CPO active space.

Distance	CASSCF	CASPT2	tpbe	ftpbe	trevpbe	ftrevpbe	tblyp	ftblyp	torelyp	ftorelyp
Distance	CASSCF	CASPT2	tpbe	ftpbe	trevpbe	ftrevpbe	tblyp	ftblyp	torelyp	ftorelyp
1.98	-1235.49529	-1236.01615	-1236.72563	-1236.93499	-1236.93327	-1237.14354	-1237.14111	-1237.34831	-1237.20454	-1237.43541
2.08	-1235.93159	-1236.43703	-1237.16527	-1237.37080	-1237.37370	-1237.58010	-1237.58032	-1237.78459	-1237.63990	-1237.86694
2.17	-1236.27308	-1236.76397	-1237.50138	-1237.70561	-1237.71080	-1237.91599	-1237.91652	-1238.11988	-1237.97379	-1238.19971
2.27	-1236.54063	-1237.01856	-1237.76407	-1237.96727	-1237.97444	-1238.17874	-1238.17941	-1238.38196	-1238.23483	-1238.45991
2.36	-1236.75126	-1237.21848	-1237.97037	-1238.17265	-1238.18165	-1238.38517	-1238.38605	-1238.58781	-1238.43965	-1238.66405
2.46	-1236.91803	-1237.42319	-1238.13325	-1238.33493	-1238.34539	-1238.54851	-1238.54933	-1238.75061	-1238.60114	-1238.82536
2.55	-1237.04723	-1237.55492	-1238.26352	-1238.46480	-1238.47649	-1238.67934	-1238.67998	-1238.88098	-1238.73039	-1238.95449
2.65	-1237.15901	-1237.66100	-1238.36835	-1238.56947	-1238.58217	-1238.78503	-1238.78508	-1238.98603	-1238.83443	-1239.05874
2.74	-1237.24683	-1237.74759	-1238.45419	-1238.65516	-1238.66888	-1238.87169	-1238.87110	-1239.07192	-1238.91978	-1239.14412
2.83	-1237.31837	-1237.81907	-1238.52463	-1238.72532	-1238.74039	-1238.94296	-1238.94128	-1239.14191	-1238.99051	-1239.21462
2.93	-1237.37935	-1237.87862	-1238.58512	-1238.78587	-1238.80178	-1239.00440	-1239.00171	-1239.20221	-1239.05095	-1239.27492
3.02	-1237.43021	-1237.92868	-1238.63611	-1238.83684	-1238.85363	-1239.05613	-1239.05265	-1239.25285	-1239.10198	-1239.32554
3.12	-1237.47285	-1237.97041	-1238.67887	-1238.87953	-1238.89723	-1239.09954	-1239.09529	-1239.29502	-1239.14477	-1239.36763
3.21	-1237.50870	-1238.00491	-1238.71426	-1238.91483	-1238.93335	-1239.13542	-1239.13065	-1239.32968	-1239.17994	-1239.40191
3.31	-1237.53870	-1238.03336	-1238.74318	-1238.94363	-1238.96279	-1239.16464	-1239.15972	-1239.35800	-1239.20835	-1239.42952
3.40	-1237.56353	-1238.05668	-1238.76651	-1238.96692	-1238.98650	-1239.18830	-1239.18339	-1239.38115	-1239.23103	-1239.45179
3.50	-1237.58387	-1238.07556	-1238.78514	-1238.98552	-1239.00542	-1239.20723	-1239.20247	-1239.39988	-1239.24897	-1239.46951
3.59	-1237.60035	-1238.09064	-1238.79984	-1239.00017	-1239.02035	-1239.22220	-1239.21769	-1239.41480	-1239.26297	-1239.48337
3.68	-1237.61355	-1238.10249	-1238.81123	-1239.01151	-1239.03192	-1239.23382	-1239.22965	-1239.42647	-1239.27367	-1239.49397
3.78	-1237.62399	-1238.11162	-1238.81983	-1239.02005	-1239.04067	-1239.24265	-1239.23884	-1239.43536	-1239.28157	-1239.50181
3.87	-1237.63215	-1238.11847	-1238.82606	-1239.02623	-1239.04702	-1239.24911	-1239.24566	-1239.44189	-1239.28711	-1239.50733
3.97	-1237.63846	-1238.12340	-1238.83027	-1239.03043	-1239.05133	-1239.25358	-1239.25045	-1239.44639	-1239.29063	-1239.51088
4.06	-1237.64327	-1238.12676	-1238.83277	-1239.03296	-1239.05391	-1239.25641	-1239.25352	-1239.44918	-1239.29244	-1239.51280
4.16	-1237.64692	-1238.12880	-1238.83383	-1239.03410	-1239.05503	-1239.25787	-1239.25511	-1239.45051	-1239.29281	-1239.51337
4.35	-1237.65166	-1238.12977	-1238.83257	-1239.03313	-1239.05389	-1239.25759	-1239.25474	-1239.44973	-1239.29030	-1239.51152
4.44	-1237.65312	-1238.12987	-1238.83040	-1239.03116	-1239.05179	-1239.25600	-1239.25292	-1239.44775	-1239.28760	-1239.50927
4.54	-1237.65417	-1238.12924	-1238.82700	-1239.02797	-1239.04847	-1239.25321	-1239.24979	-1239.44448	-1239.28373	-1239.50593
4.63	-1237.65494	-1238.12788	-1238.82301	-1239.02408	-1239.04457	-1239.24971	-1239.24604	-1239.44049	-1239.27933	-1239.50196
4.72	-1237.65543	-1238.12579	-1238.81960	-1239.02064	-1239.04128	-1239.24658	-1239.24286	-1239.43701	-1239.27568	-1239.49844
4.82	-1237.65558	-1238.12358	-1238.81678	-1239.01776	-1239.03860	-1239.24395	-1239.24028	-1239.43415	-1239.27276	-1239.49553
4.91	-1237.65536	-1238.12143	-1238.81418	-1239.01507	-1239.03614	-1239.24149	-1239.23791	-1239.43152	-1239.27012	-1239.49285
5.01	-1237.65482	-1238.11921	-1238.81162	-1239.01242	-1239.03372	-1239.23905	-1239.23561	-1239.42898	-1239.26759	-1239.49024
5.10	-1237.65397	-1238.11685	-1238.80907	-1239.00979	-1239.03131	-1239.23660	-1239.23333	-1239.42648	-1239.26511	-1239.48767
5.20	-1237.65287	-1238.11436	-1238.80651	-1239.00714	-1239.02889	-1239.23413	-1239.23105	-1239.42400	-1239.26265	-1239.48512
5.29	-1237.65156	-1238.11177	-1238.80395	-1239.00449	-1239.02647	-1239.23163	-1239.22878	-1239.42154	-1239.26022	-1239.48257
5.39	-1237.65006	-1238.10909	-1238.80140	-1239.00183	-1239.02404	-1239.22911	-1239.22652	-1239.41909	-1239.25781	-1239.48004
5.48	-1237.64843	-1238.10636	-1238.79886	-1238.99919	-1239.02162	-1239.22659	-1239.22429	-1239.41668	-1239.25542	-1239.47753
5.57	-1237.64669	-1238.10359	-1238.79635	-1238.99658	-1239.01922	-1239.22409	-1239.22210	-1239.41432	-1239.25308	-1239.47506
5.67	-1237.64487	-1238.10081	-1238.79388	-1238.99401	-1239.01686	-1239.22161	-1239.21996	-1239.41201	-1239.25079	-1239.47265

Table S7: The absolute energies in hartree of the potential energy curve of VSi in the $^2\Delta$ state with a CASSCF wave function and nom-CPO active space.

Distance	CASSCF	CASPT2	tpbe	ftpbe	trevpbe	ftrevpbe	tblyp	ftblyp	torelyp	ftorelyp
5.76	-1237.64300	-1238.09803	-1238.79146	-1238.99149	-1239.01454	-1239.21917	-1239.21788	-1239.40978	-1239.24856	-1239.47029
5.86	-1237.64110	-1238.09528	-1238.78910	-1238.98902	-1239.01227	-1239.21678	-1239.21586	-1239.40762	-1239.24639	-1239.46800
5.95	-1237.63919	-1238.09257	-1238.78680	-1238.98663	-1239.01006	-1239.21444	-1239.21392	-1239.40556	-1239.24430	-1239.46579
6.05	-1237.63729	-1238.08993	-1238.78457	-1238.98431	-1239.00792	-1239.21217	-1239.21207	-1239.40359	-1239.24228	-1239.46367
6.14	-1237.63542	-1238.08735	-1238.78241	-1238.98208	-1239.00584	-1239.20998	-1239.21030	-1239.40174	-1239.24033	-1239.46164
6.24	-1237.63360	-1238.08483	-1238.78032	-1238.97993	-1239.00382	-1239.20785	-1239.20862	-1239.39999	-1239.23847	-1239.45970
6.33	-1237.63184	-1238.08241	-1238.77830	-1238.97786	-1239.00187	-1239.20581	-1239.20703	-1239.39836	-1239.23668	-1239.45786
6.43	-1237.63015	-1238.08008	-1238.77635	-1238.97587	-1238.99998	-1239.20384	-1239.20552	-1239.39683	-1239.23496	-1239.45612
6.52	-1237.62855	-1238.07786	-1238.77447	-1238.97397	-1238.99816	-1239.20194	-1239.20408	-1239.39540	-1239.23332	-1239.45446
6.61	-1237.62703	-1238.07575	-1238.77264	-1238.97213	-1238.99639	-1239.20012	-1239.20271	-1239.39407	-1239.23174	-1239.45289
6.71	-1237.62560	-1238.07375	-1238.77087	-1238.97036	-1238.99468	-1239.19835	-1239.20141	-1239.39282	-1239.23022	-1239.45139
6.80	-1237.62428	-1238.07188	-1238.76915	-1238.96865	-1238.99301	-1239.19665	-1239.20015	-1239.39164	-1239.22875	-1239.44995
6.90	-1237.62305	-1238.07012	-1238.76748	-1238.96700	-1238.99140	-1239.19501	-1239.19894	-1239.39052	-1239.22734	-1239.44858
6.99	-1237.62192	-1238.06849	-1238.76587	-1238.96541	-1238.98984	-1239.19344	-1239.19778	-1239.38945	-1239.22598	-1239.44726
7.09	-1237.62088	-1238.06697	-1238.76432	-1238.96389	-1238.98834	-1239.19193	-1239.19666	-1239.38844	-1239.22468	-1239.44601
7.18	-1237.61993	-1238.06556	-1238.76283	-1238.96242	-1238.98690	-1239.19048	-1239.19559	-1239.38748	-1239.22344	-1239.44481
7.28	-1237.61906	-1238.06426	-1238.76140	-1238.96103	-1238.98554	-1239.18911	-1239.19456	-1239.38656	-1239.22227	-1239.44367
7.37	-1237.61828	-1238.06306	-1238.76005	-1238.95970	-1238.98424	-1239.18780	-1239.19358	-1239.38568	-1239.22116	-1239.44260
7.46	-1237.61756	-1238.06195	-1238.75876	-1238.95844	-1238.98301	-1239.18657	-1239.19265	-1239.38485	-1239.22013	-1239.44159
7.56	-1237.61692	-1238.06094	-1238.75756	-1238.95725	-1238.98186	-1239.18542	-1239.19178	-1239.38407	-1239.21916	-1239.44064
7.65	-1237.61633	-1238.06002	-1238.75642	-1238.95614	-1238.98079	-1239.18434	-1239.19095	-1239.38333	-1239.21827	-1239.43976
7.75	-1237.61580	-1238.05917	-1238.75537	-1238.95510	-1238.97980	-1239.18334	-1239.19018	-1239.38265	-1239.21745	-1239.43894
7.84	-1237.61532	-1238.05839	-1238.75439	-1238.95414	-1238.97888	-1239.18242	-1239.18947	-1239.38201	-1239.21670	-1239.43820
7.94	-1237.61489	-1238.05768	-1238.75348	-1238.95325	-1238.97804	-1239.18157	-1239.18880	-1239.38142	-1239.21602	-1239.43751
8.03	-1237.61450	-1238.05703	-1238.75265	-1238.95242	-1238.97726	-1239.18078	-1239.18819	-1239.38087	-1239.21540	-1239.43689
8.13	-1237.61415	-1238.05643	-1238.75188	-1238.95166	-1238.97655	-1239.18007	-1239.18762	-1239.38037	-1239.21484	-1239.43632
8.22	-1237.61383	-1238.05589	-1238.75117	-1238.95096	-1238.97590	-1239.17941	-1239.18711	-1239.37991	-1239.21434	-1239.43581
8.31	-1237.61354	-1238.05539	-1238.75053	-1238.95032	-1238.97532	-1239.17882	-1239.18664	-1239.37949	-1239.21390	-1239.43536
8.41	-1237.61327	-1238.05493	-1238.74994	-1238.94974	-1238.97479	-1239.17828	-1239.18621	-1239.37911	-1239.21351	-1239.43495
8.50	-1237.61304	-1238.05451	-1238.74940	-1238.94920	-1238.97431	-1239.17779	-1239.18582	-1239.37876	-1239.21316	-1239.43459
8.60	-1237.61282	-1238.05413	-1238.74892	-1238.94872	-1238.97388	-1239.17735	-1239.18547	-1239.37844	-1239.21285	-1239.43426
8.69	-1237.61263	-1238.05378	-1238.74847	-1238.94828	-1238.97350	-1239.17695	-1239.18515	-1239.37815	-1239.21258	-1239.43398
8.79	-1237.61245	-1238.05346	-1238.74807	-1238.94787	-1238.97315	-1239.17660	-1239.18486	-1239.37788	-1239.21235	-1239.43372
8.88	-1237.61229	-1238.05317	-1238.74771	-1238.94751	-1238.97284	-1239.17627	-1239.18460	-1239.37764	-1239.21214	-1239.43350
8.98	-1237.61214	-1238.05290	-1238.74738	-1238.94718	-1238.97256	-1239.17599	-1239.18437	-1239.37741	-1239.21197	-1239.43331
9.07	-1237.61201	-1238.05265	-1238.74709	-1238.94688	-1238.97232	-1239.17573	-1239.18416	-1239.37720	-1239.21182	-1239.43315
9.17	-1237.61189	-1238.05243	-1238.74682	-1238.94661	-1238.97210	-1239.17550	-1239.18398	-1239.37702	-1239.21169	-1239.43300
9.26	-1237.61178	-1238.05222	-1238.74658	-1238.94637	-1238.97190	-1239.17530	-1239.18382	-1239.37687	-1239.21159	-1239.43288
9.35	-1237.61168	-1238.05203	-1238.74637	-1238.94615	-1238.97173	-1239.17512	-1239.18367	-1239.37673	-1239.21150	-1239.43278
9.45	-1237.61159	-1238.05185	-1238.74617	-1238.94595	-1238.97158	-1239.17495	-1239.18354	-1239.37661	-1239.21142	-1239.43269
9.54	-1237.61150	-1238.05169	-1238.74599	-1238.94577	-1238.97144	-1239.17481	-1239.18343	-1239.37650	-1239.21136	-1239.43261
9.64	-1237.61142	-1238.05154	-1238.74583	-1238.94561	-1238.97132	-1239.17468	-1239.18332	-1239.37640	-1239.21131	-1239.43254
9.73	-1237.61135	-1238.05141	-1238.74569	-1238.94546	-1238.97121	-1239.17456	-1239.18323	-1239.37631	-1239.21126	-1239.43249
9.83	-1237.61129	-1238.05128	-1238.74556	-1238.94532	-1238.97112	-1239.17446	-1239.18315	-1239.37623	-1239.21123	-1239.43244
9.92	-1237.61123	-1238.05117	-1238.74544	-1238.94520	-1238.97104	-1239.17437	-1239.18308	-1239.37617	-1239.21121	-1239.43240
10.02	-1237.61118	-1238.05106	-1238.74534	-1238.94509	-1238.97097	-1239.17429	-1239.18302	-1239.37611	-1239.21119	-1239.43237
10.11	-1237.61113	-1238.05097	-1238.74524	-1238.94499	-1238.97090	-1239.17422	-1239.18297	-1239.37606	-1239.21117	-1239.43235
10.20	-1237.61108	-1238.05089	-1238.74516	-1238.94490	-1238.97085	-1239.17415	-1239.18292	-1239.37601	-1239.21116	-1239.43232
10.30	-1237.61104	-1238.05081	-1238.74508	-1238.94482	-1238.97080	-1239.17410	-1239.18288	-1239.37597	-1239.21116	-1239.43230
10.39	-1237.61100	-1238.05075	-1238.74501	-1238.94475	-1238.97075	-1239.17404	-1239.18285	-1239.37593	-1239.21115	-1239.43229
10.49	-1237.61097	-1238.05070	-1238.74494	-1238.94468	-1238.97071	-1239.17400	-1239.18282	-1239.37590	-1239.21115	-1239.43228
10.58	-1237.61094	-1238.05065	-1238.74488	-1238.94462	-1238.97068	-1239.17395	-1239.18279	-1239.37587	-1239.21115	-1239.43227

Table S7: The absolute energies in hartree of the potential energy curve of VSi in the $^2\Delta$ state with a CASSCF wave function and nom-CPO active space.

Distance	CASSCF	CASPT2	tpbe	ftpbe	trevpbe	ftrevpbe	tblyp	ftblyp	torelyp	ftorelyp
10.68	-1237.61091	-1238.05062	-1238.74483	-1238.94456	-1238.97065	-1239.17392	-1239.18277	-1239.37585	-1239.21115	-1239.43226
10.77	-1237.61088	-1238.05060	-1238.74479	-1238.94451	-1238.97062	-1239.17389	-1239.18276	-1239.37583	-1239.21116	-1239.43225
10.87	-1237.61085	-1238.05059	-1238.74474	-1238.94446	-1238.97060	-1239.17386	-1239.18274	-1239.37581	-1239.21116	-1239.43225
10.96	-1237.61083	-1238.05059	-1238.74471	-1238.94442	-1238.97058	-1239.17383	-1239.18273	-1239.37580	-1239.21116	-1239.43225
11.05	-1237.61081	-1238.05061	-1238.74467	-1238.94439	-1238.97056	-1239.17381	-1239.18272	-1239.37579	-1239.21117	-1239.43225
11.15	-1237.61079	-1238.05063	-1238.74464	-1238.94435	-1238.97055	-1239.17379	-1239.18271	-1239.37578	-1239.21118	-1239.43224
11.24	-1237.61077	-1238.05067	-1238.74461	-1238.94432	-1238.97053	-1239.17377	-1239.18271	-1239.37578	-1239.21118	-1239.43224
11.34	-1237.61075	-1238.05071	-1238.74458	-1238.94429	-1238.97052	-1239.17375	-1239.18270	-1239.37577	-1239.21119	-1239.43224
11.43	-1237.61073	-1238.05073	-1238.74456	-1238.94426	-1238.97051	-1239.17374	-1239.18270	-1239.37576	-1239.21119	-1239.43224
11.53	-1237.61072	-1238.05073	-1238.74453	-1238.94423	-1238.97050	-1239.17372	-1239.18269	-1239.37575	-1239.21119	-1239.43223
11.62	-1237.61071	-1238.05072	-1238.74451	-1238.94421	-1238.97049	-1239.17371	-1239.18270	-1239.37575	-1239.21120	-1239.43223
11.72	-1237.61069	-1238.05067	-1238.74450	-1238.94419	-1238.97048	-1239.17370	-1239.18270	-1239.37576	-1239.21121	-1239.43224
11.81	-1237.61068	-1238.05059	-1238.74448	-1238.94417	-1238.97048	-1239.17369	-1239.18270	-1239.37576	-1239.21122	-1239.43224
11.91	-1237.61067	-1238.05044	-1238.74446	-1238.94415	-1238.97047	-1239.17367	-1239.18270	-1239.37575	-1239.21122	-1239.43223
12.00	-1237.61066	-1238.05025	-1238.74445	-1238.94414	-1238.97046	-1239.17366	-1239.18270	-1239.37575	-1239.21122	-1239.43223
12.09	-1237.61065	-1238.05003	-1238.74443	-1238.94412	-1238.97046	-1239.17366	-1239.18271	-1239.37576	-1239.21123	-1239.43223
12.19	-1237.61064	-1238.04983	-1238.74442	-1238.94411	-1238.97045	-1239.17365	-1239.18271	-1239.37576	-1239.21123	-1239.43223
12.28	-1237.61063	-1238.04967	-1238.74441	-1238.94410	-1238.97045	-1239.17364	-1239.18271	-1239.37576	-1239.21123	-1239.43223
12.38	-1237.61062	-1238.04954	-1238.74440	-1238.94408	-1238.97044	-1239.17364	-1239.18272	-1239.37576	-1239.21124	-1239.43223
12.47	-1237.61061	-1238.04944	-1238.74439	-1238.94407	-1238.97044	-1239.17363	-1239.18272	-1239.37577	-1239.21124	-1239.43223
12.57	-1237.61061	-1238.04937	-1238.74438	-1238.94406	-1238.97044	-1239.17362	-1239.18273	-1239.37577	-1239.21124	-1239.43222
12.66	-1237.61060	-1238.04930	-1238.74437	-1238.94405	-1238.97043	-1239.17362	-1239.18273	-1239.37577	-1239.21124	-1239.43222
12.76	-1237.61059	-1238.04926	-1238.74436	-1238.94404	-1238.97043	-1239.17361	-1239.18274	-1239.37577	-1239.21124	-1239.43222
12.85	-1237.61059	-1238.04921	-1238.74435	-1238.94403	-1238.97043	-1239.17361	-1239.18274	-1239.37578	-1239.21124	-1239.43222
12.94	-1237.61058	-1238.04918	-1238.74435	-1238.94403	-1238.97042	-1239.17360	-1239.18275	-1239.37578	-1239.21125	-1239.43222
13.04	-1237.61058	-1238.04915	-1238.74434	-1238.94402	-1238.97042	-1239.17360	-1239.18275	-1239.37579	-1239.21125	-1239.43222
13.13	-1237.61057	-1238.04913	-1238.74434	-1238.94402	-1238.97042	-1239.17360	-1239.18276	-1239.37579	-1239.21125	-1239.43222
13.23	-1237.61056	-1238.04911	-1238.74433	-1238.94401	-1238.97042	-1239.17360	-1239.18276	-1239.37580	-1239.21125	-1239.43222
13.32	-1237.61056	-1238.04909	-1238.74433	-1238.94401	-1238.97042	-1239.17359	-1239.18277	-1239.37580	-1239.21125	-1239.43222
13.42	-1237.61056	-1238.04907	-1238.74432	-1238.94400	-1238.97041	-1239.17359	-1239.18277	-1239.37581	-1239.21125	-1239.43222
13.51	-1237.61055	-1238.04847	-1238.74432	-1238.94400	-1238.97041	-1239.17359	-1239.18278	-1239.37581	-1239.21125	-1239.43222
13.61	-1237.61055	-1238.04845	-1238.74431	-1238.94399	-1238.97041	-1239.17358	-1239.18278	-1239.37581	-1239.21125	-1239.43222
13.70	-1237.61054	-1238.04843	-1238.74431	-1238.94399	-1238.97041	-1239.17358	-1239.18278	-1239.37581	-1239.21125	-1239.43221
13.80	-1237.61054	-1238.04842	-1238.74431	-1238.94398	-1238.97041	-1239.17358	-1239.18279	-1239.37582	-1239.21125	-1239.43221
13.89	-1237.61054	-1238.04841	-1238.74430	-1238.94398	-1238.97040	-1239.17358	-1239.18279	-1239.37582	-1239.21125	-1239.43221
13.98	-1237.61053	-1238.04839	-1238.74430	-1238.94397	-1238.97040	-1239.17358	-1239.18279	-1239.37582	-1239.21125	-1239.43221
14.08	-1237.61053	-1238.04838	-1238.74430	-1238.94397	-1238.97040	-1239.17358	-1239.18280	-1239.37582	-1239.21125	-1239.43221
14.17	-1237.61053	-1238.04837	-1238.74429	-1238.94397	-1238.97040	-1239.17358	-1239.18280	-1239.37583	-1239.21125	-1239.43221
14.27	-1237.61052	-1238.04836	-1238.74429	-1238.94397	-1238.97040	-1239.17358	-1239.18280	-1239.37583	-1239.21125	-1239.43221
14.36	-1237.61052	-1238.04835	-1238.74429	-1238.94396	-1238.97040	-1239.17358	-1239.18281	-1239.37583	-1239.21125	-1239.43221
14.46	-1237.61052	-1238.04834	-1238.74429	-1238.94396	-1238.97039	-1239.17358	-1239.18281	-1239.37584	-1239.21125	-1239.43221
14.55	-1237.61051	-1238.04834	-1238.74429	-1238.94396	-1238.97039	-1239.17358	-1239.18281	-1239.37584	-1239.21125	-1239.43221
14.65	-1237.61051	-1238.04833	-1238.74428	-1238.94396	-1238.97039	-1239.17358	-1239.18282	-1239.37584	-1239.21126	-1239.43221
14.74	-1237.61051	-1238.04832	-1238.74428	-1238.94396	-1238.97039	-1239.17358	-1239.18282	-1239.37585	-1239.21126	-1239.43221
14.83	-1237.61051	-1238.04831	-1238.74428	-1238.94396	-1238.97039	-1239.17358	-1239.18282	-1239.37585	-1239.21126	-1239.43221
14.93	-1237.61050	-1238.04831	-1238.74428	-1238.94395	-1238.97039	-1239.17358	-1239.18282	-1239.37585	-1239.21125	-1239.43221
15.02	-1237.61050	-1238.04830	-1238.74428	-1238.94395	-1238.97039	-1239.17358	-1239.18283	-1239.37585	-1239.21125	-1239.43221
15.12	-1237.61050	-1238.04829	-1238.74427	-1238.94395	-1238.97039	-1239.17358	-1239.18283	-1239.37586	-1239.21125	-1239.43221
15.21	-1237.61050	-1238.04829	-1238.74427	-1238.94395	-1238.97038	-1239.17358	-1239.18283	-1239.37586	-1239.21125	-1239.43221
15.31	-1237.61050	-1238.04827	-1238.74427	-1238.94395	-1238.97038	-1239.17358	-1239.18283	-1239.37586	-1239.21125	-1239.43220
15.40	-1237.61049	-1238.04826	-1238.74427	-1238.94395	-1238.97038	-1239.17358	-1239.18283	-1239.37586	-1239.21125	-1239.43220
15.50	-1237.61049	-1238.04826	-1238.74427	-1238.94395	-1238.97038	-1239.17358	-1239.18284	-1239.37587	-1239.21125	-1239.43221

Table S7: The absolute energies in hartree of the potential energy curve of VSi in the $^2\Delta$ state with a CASSCF wave function and nom-CPO active space.

Distance	CASSCF	CASPT2	tpbe	ftpbe	trevpbe	ftrevpbe	tblyp	ftblyp	torelyp	ftorelyp
15.59	-1237.61049	-1238.04825	-1238.74427	-1238.94395	-1238.97038	-1239.17358	-1239.18284	-1239.37587	-1239.21126	-1239.43221
15.68	-1237.61049	-1238.04825	-1238.74427	-1238.94395	-1238.97038	-1239.17358	-1239.18284	-1239.37587	-1239.21126	-1239.43221
15.78	-1237.61049	-1238.04824	-1238.74427	-1238.94394	-1238.97038	-1239.17358	-1239.18285	-1239.37587	-1239.21126	-1239.43221
15.87	-1237.61049	-1238.04824	-1238.74427	-1238.94394	-1238.97038	-1239.17358	-1239.18285	-1239.37587	-1239.21126	-1239.43221
15.97	-1237.61048	-1238.04821	-1238.74427	-1238.94394	-1238.97038	-1239.17358	-1239.18285	-1239.37588	-1239.21126	-1239.43221
16.06	-1237.61048	-1238.04821	-1238.74427	-1238.94394	-1238.97038	-1239.17358	-1239.18285	-1239.37588	-1239.21126	-1239.43221
16.16	-1237.61048	-1238.04821	-1238.74427	-1238.94394	-1238.97038	-1239.17358	-1239.18285	-1239.37588	-1239.21126	-1239.43221
16.25	-1237.61048	-1238.04821	-1238.74426	-1238.94394	-1238.97038	-1239.17358	-1239.18285	-1239.37588	-1239.21126	-1239.43221
16.35	-1237.61048	-1238.04821	-1238.74426	-1238.94394	-1238.97038	-1239.17358	-1239.18285	-1239.37588	-1239.21126	-1239.43221
16.44	-1237.61048	-1238.04821	-1238.74426	-1238.94394	-1238.97038	-1239.17358	-1239.18286	-1239.37588	-1239.21126	-1239.43221
16.54	-1237.61048	-1238.04821	-1238.74426	-1238.94394	-1238.97038	-1239.17358	-1239.18286	-1239.37588	-1239.21125	-1239.43221
16.63	-1237.61047	-1238.04821	-1238.74426	-1238.94394	-1238.97038	-1239.17358	-1239.18286	-1239.37588	-1239.21125	-1239.43221
16.72	-1237.61047	-1238.04821	-1238.74426	-1238.94394	-1238.97038	-1239.17358	-1239.18286	-1239.37588	-1239.21125	-1239.43221
16.82	-1237.61047	-1238.04821	-1238.74426	-1238.94394	-1238.97037	-1239.17358	-1239.18286	-1239.37589	-1239.21125	-1239.43221
16.91	-1237.61047	-1238.04821	-1238.74426	-1238.94394	-1238.97037	-1239.17358	-1239.18286	-1239.37589	-1239.21125	-1239.43221
17.01	-1237.61047	-1238.04821	-1238.74426	-1238.94394	-1238.97037	-1239.17358	-1239.18286	-1239.37589	-1239.21125	-1239.43221
17.10	-1237.61047	-1238.04821	-1238.74426	-1238.94394	-1238.97037	-1239.17358	-1239.18286	-1239.37589	-1239.21125	-1239.43221
17.20	-1237.61047	-1238.04821	-1238.74426	-1238.94394	-1238.97037	-1239.17358	-1239.18286	-1239.37589	-1239.21125	-1239.43221
17.29	-1237.61047	-1238.04821	-1238.74426	-1238.94394	-1238.97037	-1239.17358	-1239.18286	-1239.37589	-1239.21125	-1239.43221
17.39	-1237.61047	-1238.04821	-1238.74426	-1238.94394	-1238.97037	-1239.17358	-1239.18286	-1239.37589	-1239.21125	-1239.43221
17.48	-1237.61046	-1238.04821	-1238.74426	-1238.94394	-1238.97037	-1239.17358	-1239.18286	-1239.37589	-1239.21125	-1239.43221
17.57	-1237.61046	-1238.04821	-1238.74426	-1238.94394	-1238.97037	-1239.17358	-1239.18287	-1239.37589	-1239.21125	-1239.43221
17.67	-1237.61046	-1238.04821	-1238.74426	-1238.94394	-1238.97037	-1239.17358	-1239.18287	-1239.37589	-1239.21125	-1239.43221
17.76	-1237.61046	-1238.04821	-1238.74426	-1238.94394	-1238.97037	-1239.17358	-1239.18287	-1239.37590	-1239.21125	-1239.43221
17.86	-1237.61046	-1238.04821	-1238.74426	-1238.94394	-1238.97037	-1239.17358	-1239.18287	-1239.37590	-1239.21125	-1239.43221
17.95	-1237.61046	-1238.04821	-1238.74426	-1238.94393	-1238.97037	-1239.17358	-1239.18287	-1239.37590	-1239.21125	-1239.43221
18.05	-1237.61046	-1238.04821	-1238.74426	-1238.94393	-1238.97037	-1239.17358	-1239.18287	-1239.37590	-1239.21125	-1239.43221

Table S8: The absolute energies in hartree of the potential energy curve of VSi in the $^4\Pi$ state with a SP-based wave function and nom-CPO active space.

Distance	SP	tpbe	ftpbe	trevpbe	ftrevpbe	tblyp	ftblyp	torelyp	ftorelyp
1.98	-1235.42856	-1236.68402	-1236.68402	-1236.88451		-1237.09693	-1237.29391	-1237.16678	-1237.38708
2.08	-1235.85641	-1237.10455	-1237.10455	-1237.30404		-1237.51803	-1237.71395	-1237.58435	-1237.80332
2.17	-1236.19035	-1237.43279	-1237.43279	-1237.63154		-1237.84687	-1238.04194	-1237.91003	-1238.12806
2.27	-1236.45192	-1237.68999	-1237.68999	-1237.88821		-1238.10477	-1238.29921	-1238.16511	-1238.38248
2.36	-1236.65789	-1237.89289	-1237.89289	-1238.09066		-1238.30841	-1238.50241	-1238.36634	-1238.58327
2.46	-1236.82170	-1238.05283	-1238.05283	-1238.25046		-1238.46915	-1238.66404	-1238.52476	-1238.74562
2.55	-1236.95571	-1238.18059	-1238.18059	-1238.37901		-1238.59740	-1238.79296	-1238.65063	-1238.87334
2.65	-1237.05979	-1238.28362	-1238.28362	-1238.48656		-1238.70453	-1238.90447	-1238.74454	-1238.96804
2.74	-1237.15596	-1238.37941	-1238.37941	-1238.58327		-1238.80041	-1239.00096	-1238.83942	-1239.06395
2.83	-1237.23895	-1238.46063	-1238.46063	-1238.66484	-1238.88183	-1238.88078	-1239.08064	-1238.91984	-1239.14554
2.93	-1237.31252	-1238.53105	-1238.53105	-1238.73388	-1238.95178	-1238.95082	-1239.14885	-1238.98975	-1239.21429
3.02	-1237.37517	-1238.59091	-1238.59091	-1238.79281	-1239.01129	-1239.01073	-1239.20761	-1239.04930	-1239.27286
3.12	-1237.42795	-1238.64138	-1238.64138	-1238.84271	-1239.06165	-1239.06133	-1239.25753	-1239.09940	-1239.32224
3.21	-1237.47219	-1238.68347	-1238.68347	-1238.88433	-1239.10370	-1239.10359	-1239.29925	-1239.14115	-1239.36337
3.31	-1237.50909	-1238.71834	-1238.71834	-1238.91878	-1239.13855	-1239.13866	-1239.33382	-1239.17575	-1239.39741
3.40	-1237.53970	-1238.74702	-1238.74702	-1238.94708	-1239.16723	-1239.16757	-1239.36227	-1239.20421	-1239.42535
3.50	-1237.56489	-1238.77038	-1238.77038	-1238.97010	-1239.19062	-1239.19120	-1239.38547	-1239.22742	-1239.44809
3.59	-1237.58547	-1238.78923	-1238.78923	-1238.98864	-1239.20950	-1239.21033	-1239.40420	-1239.24615	-1239.46640
3.68	-1237.60210	-1238.80423	-1238.80423	-1239.00335	-1239.22455	-1239.22564	-1239.41912	-1239.26108	-1239.48093

Table S8: The absolute energies in hartree of the potential energy curve of VSi in the $^4\Pi$ state with a SP-based wave function and nom-CPO active space.

Distance	SP	tpbe	ftpbe	trevpbe	ftrevpbe	tblyp	ftblyp	torelyp	ftorelyp
3.78	-1237.61540	-1238.81594	-1238.81594	-1239.01480	-1239.23633	-1239.23768	-1239.43078	-1239.27275	-1239.49225
3.87	-1237.62588	-1238.82485	-1238.82485	-1239.02349	-1239.24533	-1239.24692	-1239.43967	-1239.28164	-1239.50083
3.97	-1237.63402	-1238.83136	-1238.83136	-1239.02981	-1239.25196	-1239.25376	-1239.44618	-1239.28813	-1239.50705
4.06	-1237.64023	-1238.83581	-1238.83581	-1239.03412	-1239.25659	-1239.25854	-1239.45066	-1239.29254	-1239.51127
4.16	-1237.64484	-1238.83845	-1238.83845	-1239.03668	-1239.25947	-1239.26150	-1239.45336	-1239.29512	-1239.51372
4.35	-1237.64818	-1238.83949	-1238.83949	-1239.03769	-1239.26082	-1239.26283	-1239.45448	-1239.29605	-1239.51461
4.44	-1237.65051	-1238.83912	-1238.83912	-1239.03737	-1239.26084	-1239.26273	-1239.45423	-1239.29553	-1239.51414
4.54	-1237.65206	-1238.83753	-1238.83753	-1239.03590	-1239.25974	-1239.26139	-1239.45279	-1239.29375	-1239.51251
4.63	-1237.65300	-1238.83495	-1238.83495	-1239.03350	-1239.25771	-1239.25901	-1239.45039	-1239.29097	-1239.50996
4.72	-1237.65347	-1238.83160	-1238.83160	-1239.03035	-1239.25495	-1239.25583	-1239.44722	-1239.28741	-1239.50669
4.82	-1237.65357	-1238.82770	-1238.82770	-1239.02667	-1239.25167	-1239.25207	-1239.44351	-1239.28333	-1239.50295
4.91	-1237.65289	-1238.81929	-1238.81929	-1239.01868	-1239.24808	-1239.24392	-1239.43551	-1239.27474	-1239.49505
5.01	-1237.65219	-1238.81519	-1238.81519	-1239.01474	-1239.24441	-1239.23997	-1239.43162	-1239.27064	-1239.49125
5.10	-1237.65127	-1238.81124	-1238.81124	-1239.01092	-1239.24081	-1239.23620	-1239.42788	-1239.26674	-1239.48760
5.20	-1237.65017	-1238.80736	-1238.80736	-1239.00715	-1239.23728	-1239.23253	-1239.42423	-1239.26298	-1239.48403
5.29	-1237.64890	-1238.80380	-1238.80380	-1239.00364	-1239.23377	-1239.22920	-1239.42091	-1239.25957	-1239.48076
5.39	-1237.64747	-1238.80070	-1238.80070	-1239.00058	-1239.23048	-1239.22636	-1239.41807	-1239.25666	-1239.47796
5.48	-1237.64590	-1238.79788	-1238.79788	-1238.99779	-1239.22761	-1239.22382	-1239.41553	-1239.25405	-1239.47545
5.57	-1237.64421	-1238.79520	-1238.79520	-1238.99514	-1239.22499	-1239.22143	-1239.41315	-1239.25160	-1239.47309
5.67	-1237.64243	-1238.79263	-1238.79263	-1238.99259	-1239.22249	-1239.21917	-1239.41089	-1239.24925	-1239.47083
5.76	-1237.64057	-1238.79015	-1238.79015	-1238.99012	-1239.22008	-1239.21700	-1239.40874	-1239.24700	-1239.46866
5.86	-1237.63867	-1238.78775	-1238.78775	-1238.98774	-1239.21773	-1239.21493	-1239.40668	-1239.24484	-1239.46657
5.95	-1237.63674	-1238.78544	-1238.78544	-1238.98543	-1239.21545	-1239.21295	-1239.40474	-1239.24277	-1239.46458
6.05	-1237.63480	-1238.78321	-1238.78321	-1238.98322	-1239.21324	-1239.21107	-1239.40289	-1239.24078	-1239.46268
6.14	-1237.63288	-1238.78105	-1238.78105	-1238.98108	-1239.21111	-1239.20929	-1239.40115	-1239.23887	-1239.46085
6.24	-1237.63098	-1238.77898	-1238.77898	-1238.97902	-1239.20903	-1239.20760	-1239.39951	-1239.23705	-1239.45910
6.33	-1237.62912	-1238.77699	-1238.77699	-1238.97702	-1239.20702	-1239.20600	-1239.39797	-1239.23530	-1239.45743
6.43	-1237.62732	-1238.77506	-1238.77506	-1238.97511	-1239.20507	-1239.20448	-1239.39653	-1239.23363	-1239.45585
6.52	-1237.62558	-1238.77321	-1238.77321	-1238.97327	-1239.20319	-1239.20306	-1239.39519	-1239.23204	-1239.45435
6.61	-1237.62393	-1238.77141	-1238.77141	-1238.97149	-1239.20138	-1239.20172	-1239.39395	-1239.23052	-1239.45290
6.71	-1237.62237	-1238.76968	-1238.76968	-1238.96976	-1239.19961	-1239.20045	-1239.39278	-1239.22905	-1239.45150
6.80	-1237.62090	-1238.76799	-1238.76799	-1238.96808	-1239.19789	-1239.19924	-1239.39169	-1239.22763	-1239.45015
6.90	-1237.61955	-1238.76633	-1238.76633	-1238.96644	-1239.19620	-1239.19808	-1239.39066	-1239.22626	-1239.44884
6.99	-1237.61831	-1238.76469	-1238.76469	-1238.96482	-1239.19455	-1239.19695	-1239.38967	-1239.22490	-1239.44754
7.09	-1237.61719	-1238.76308	-1238.76308	-1238.96323	-1239.19293	-1239.19584	-1239.38872	-1239.22358	-1239.44625
7.18	-1237.61619	-1238.76151	-1238.76151	-1238.96168	-1239.19132	-1239.19477	-1239.38779	-1239.22230	-1239.44499
7.28	-1237.61531	-1238.76000	-1238.76000	-1238.96019	-1239.18976	-1239.19375	-1239.38691	-1239.22108	-1239.44379
7.37	-1237.61455	-1238.75859	-1238.75859	-1238.95880	-1239.18827	-1239.19279	-1239.38609	-1239.21995	-1239.44267
7.46	-1237.61389	-1238.75729	-1238.75729	-1238.95754	-1239.18689	-1239.19190	-1239.38532	-1239.21894	-1239.44165
7.56	-1237.61331	-1238.75613	-1238.75613	-1238.95639	-1239.18564	-1239.19111	-1239.38462	-1239.21803	-1239.44074
7.65	-1237.61281	-1238.75509	-1238.75509	-1238.95537	-1239.18452	-1239.19039	-1239.38398	-1239.21724	-1239.43994
7.75	-1237.61237	-1238.75416	-1238.75416	-1238.95445	-1239.18352	-1239.18975	-1239.38340	-1239.21655	-1239.43923
7.84	-1237.61198	-1238.75333	-1238.75333	-1238.95363	-1239.18265	-1239.18918	-1239.38288	-1239.21595	-1239.43861
7.94	-1237.61164	-1238.75259	-1238.75259	-1238.95289	-1239.18187	-1239.18866	-1239.38240	-1239.21542	-1239.43807
8.03	-1237.61133	-1238.75192	-1238.75192	-1238.95223	-1239.18118	-1239.18820	-1239.38197	-1239.21496	-1239.43759
8.13	-1237.61105	-1238.75133	-1238.75133	-1238.95163	-1239.18057	-1239.18779	-1239.38158	-1239.21456	-1239.43718
8.22	-1237.61081	-1238.75079	-1238.75079	-1238.95109	-1239.18003	-1239.18742	-1239.38123	-1239.21422	-1239.43681
8.31	-1237.61059	-1238.75031	-1238.75031	-1238.95061	-1239.17954	-1239.18709	-1239.38091	-1239.21392	-1239.43650
8.41	-1237.61039	-1238.74987	-1238.74987	-1238.95017	-1239.17910	-1239.18680	-1239.38062	-1239.21366	-1239.43622
8.50	-1237.61021	-1238.74948	-1238.74948	-1238.94977	-1239.17871	-1239.18653	-1239.38036	-1239.21344	-1239.43598
8.60	-1237.61004	-1238.74912	-1238.74912	-1238.94940	-1239.17837	-1239.18629	-1239.38012	-1239.21324	-1239.43577
8.69	-1237.60989	-1238.74879	-1238.74879	-1238.94907	-1239.17805	-1239.18608	-1239.37990	-1239.21308	-1239.43558

Table S8: The absolute energies in hartree of the potential energy curve of VSi in the $^4\Pi$ state with a SP-based wave function and nom-CPO active space.

Distance	SP	tpbe	ftpbe	trevpbe	ftrevpbe	tblyp	ftblyp	torelyp	ftorelyp
8.79	-1237.60976	-1238.74850	-1238.74850	-1238.94877	-1239.17777	-1239.18588	-1239.37971	-1239.21294	-1239.43542
8.88	-1237.60963	-1238.74823	-1238.74823	-1238.94850	-1239.17752	-1239.18571	-1239.37953	-1239.21282	-1239.43528
8.98	-1237.60952	-1238.74799	-1238.74799	-1238.94825	-1239.17729	-1239.18555	-1239.37937	-1239.21272	-1239.43517
9.07	-1237.60941	-1238.74778	-1238.74778	-1238.94802	-1239.17709	-1239.18542	-1239.37923	-1239.21264	-1239.43507
9.17	-1237.60932	-1238.74758	-1238.74758	-1238.94782	-1239.17691	-1239.18530	-1239.37910	-1239.21258	-1239.43498
9.26	-1237.60923	-1238.74741	-1238.74741	-1238.94764	-1239.17675	-1239.18519	-1239.37899	-1239.21253	-1239.43491
9.35	-1237.60915	-1238.74725	-1238.74725	-1238.94747	-1239.17660	-1239.18510	-1239.37889	-1239.21249	-1239.43486
9.45	-1237.60908	-1238.74711	-1238.74711	-1238.94732	-1239.17648	-1239.18502	-1239.37879	-1239.21245	-1239.43481
9.54	-1237.60901	-1238.74698	-1238.74698	-1238.94718	-1239.17637	-1239.18495	-1239.37871	-1239.21243	-1239.43477
9.64	-1237.60895	-1238.74686	-1238.74686	-1238.94706	-1239.17626	-1239.18488	-1239.37864	-1239.21242	-1239.43474
9.73	-1237.60889	-1238.74676	-1238.74676	-1238.94694	-1239.17618	-1239.18482	-1239.37857	-1239.21240	-1239.43471
9.83	-1237.60884	-1238.74666	-1238.74666	-1238.94684	-1239.17609	-1239.18478	-1239.37852	-1239.21240	-1239.43468
9.92	-1237.60879	-1238.74657	-1238.74657	-1238.94674	-1239.17602	-1239.18473	-1239.37846	-1239.21240	-1239.43466
10.02	-1237.60875	-1238.74649	-1238.74649	-1238.94665	-1239.17596	-1239.18470	-1239.37842	-1239.21240	-1239.43465
10.11	-1237.60870	-1238.74642	-1238.74642	-1238.94657	-1239.17590	-1239.18467	-1239.37838	-1239.21240	-1239.43463
10.20	-1237.60867	-1238.74636	-1238.74636	-1238.94650	-1239.17584	-1239.18464	-1239.37835	-1239.21241	-1239.43462
10.30	-1237.60863	-1238.74630	-1238.74630	-1238.94644	-1239.17580	-1239.18462	-1239.37832	-1239.21242	-1239.43462
10.39	-1237.60860	-1238.74625	-1238.74625	-1238.94638	-1239.17576	-1239.18461	-1239.37829	-1239.21243	-1239.43462
10.49	-1237.60857	-1238.74620	-1238.74620	-1238.94633	-1239.17572	-1239.18459	-1239.37827	-1239.21244	-1239.43462
10.58	-1237.60854	-1238.74616	-1238.74616	-1238.94628	-1239.17569	-1239.18458	-1239.37825	-1239.21246	-1239.43462
10.68	-1237.60851	-1238.74612	-1238.74612	-1238.94624	-1239.17567	-1239.18457	-1239.37824	-1239.21247	-1239.43462
10.77	-1237.60849	-1238.74609	-1238.74609	-1238.94620	-1239.17564	-1239.18457	-1239.37823	-1239.21248	-1239.43462
10.87	-1237.60846	-1238.74606	-1238.74606	-1238.94616	-1239.17562	-1239.18456	-1239.37821	-1239.21249	-1239.43462
10.96	-1237.60844	-1238.74603	-1238.74603	-1238.94612	-1239.17560	-1239.18456	-1239.37820	-1239.21250	-1239.43462
11.05	-1237.60842	-1238.74600	-1238.74600	-1238.94609	-1239.17558	-1239.18455	-1239.37820	-1239.21251	-1239.43462
11.15	-1237.60840	-1238.74597	-1238.74597	-1238.94606	-1239.17556	-1239.18455	-1239.37819	-1239.21252	-1239.43462
11.24	-1237.60839	-1238.74595	-1238.74595	-1238.94603	-1239.17554	-1239.18456	-1239.37819	-1239.21253	-1239.43461
11.34	-1237.60837	-1238.74593	-1238.74593	-1238.94600	-1239.17553	-1239.18456	-1239.37818	-1239.21254	-1239.43461
11.43	-1237.60835	-1238.74591	-1238.74591	-1238.94598	-1239.17551	-1239.18456	-1239.37818	-1239.21256	-1239.43461
11.53	-1237.60834	-1238.74589	-1238.74589	-1238.94596	-1239.17550	-1239.18457	-1239.37818	-1239.21257	-1239.43462
11.62	-1237.60833	-1238.74588	-1238.74588	-1238.94595	-1239.17549	-1239.18457	-1239.37818	-1239.21258	-1239.43462
11.72	-1237.60831	-1238.74587	-1238.74587	-1238.94593	-1239.17548	-1239.18458	-1239.37819	-1239.21258	-1239.43462
11.81	-1237.60830	-1238.74585	-1238.74585	-1238.94591	-1239.17548	-1239.18458	-1239.37819	-1239.21259	-1239.43462
11.91	-1237.60829	-1238.74584	-1238.74584	-1238.94590	-1239.17547	-1239.18459	-1239.37819	-1239.21260	-1239.43462
12.00	-1237.60828	-1238.74583	-1238.74583	-1238.94589	-1239.17546	-1239.18460	-1239.37819	-1239.21260	-1239.43462
12.09	-1237.60827	-1238.74582	-1238.74582	-1238.94587	-1239.17546	-1239.18460	-1239.37819	-1239.21260	-1239.43461
12.19	-1237.60826	-1238.74581	-1238.74581	-1238.94586	-1239.17545	-1239.18460	-1239.37820	-1239.21261	-1239.43461
12.28	-1237.60825	-1238.74580	-1238.74580	-1238.94585	-1239.17544	-1239.18461	-1239.37820	-1239.21261	-1239.43461
12.38	-1237.60824	-1238.74579	-1238.74579	-1238.94584	-1239.17544	-1239.18462	-1239.37820	-1239.21262	-1239.43461
12.47	-1237.60824	-1238.74578	-1238.74578	-1238.94583	-1239.17543	-1239.18462	-1239.37821	-1239.21262	-1239.43461
12.57	-1237.60823	-1238.74578	-1238.74578	-1238.94582	-1239.17542	-1239.18463	-1239.37821	-1239.21262	-1239.43460
12.66	-1237.60822	-1238.74577	-1238.74577	-1238.94581	-1239.17542	-1239.18464	-1239.37822	-1239.21263	-1239.43460
12.76	-1237.60822	-1238.74576	-1238.74576	-1238.94580	-1239.17541	-1239.18464	-1239.37822	-1239.21263	-1239.43460
12.85	-1237.60821	-1238.74576	-1238.74576	-1238.94580	-1239.17541	-1239.18465	-1239.37822	-1239.21263	-1239.43460
12.94	-1237.60820	-1238.74576	-1238.74576	-1238.94579	-1239.17541	-1239.18466	-1239.37823	-1239.21264	-1239.43460
13.04	-1237.60820	-1238.74575	-1238.74575	-1238.94579	-1239.17540	-1239.18466	-1239.37824	-1239.21264	-1239.43460
13.13	-1237.60819	-1238.74575	-1238.74575	-1238.94578	-1239.17540	-1239.18467	-1239.37824	-1239.21264	-1239.43460
13.23	-1237.60819	-1238.74575	-1238.74575	-1238.94578	-1239.17540	-1239.18467	-1239.37824	-1239.21264	-1239.43460
13.32	-1237.60818	-1238.74574	-1238.74574	-1238.94578	-1239.17540	-1239.18468	-1239.37825	-1239.21264	-1239.43459
13.42	-1237.60818	-1238.74574	-1238.74574	-1238.94577	-1239.17539	-1239.18468	-1239.37825	-1239.21264	-1239.43459
13.51	-1237.60817	-1238.74574	-1238.74574	-1238.94577	-1239.17539	-1239.18469	-1239.37826	-1239.21264	-1239.43459
13.61	-1237.60817	-1238.74573	-1238.74573	-1238.94576	-1239.17539	-1239.18469	-1239.37826	-1239.21264	-1239.43459

Table S8: The absolute energies in hartree of the potential energy curve of VSi in the $^4\Pi$ state with a SP-based wave function and nom-CPO active space.

Distance	SP	tpbe	ftpbe	trevpbe	ftrevpbe	tblyp	ftblyp	torelyp	ftorelyp
13.70	-1237.60816	-1238.74573	-1238.74573	-1238.94576	-1239.17539	-1239.18470	-1239.37826	-1239.21264	-1239.43459
13.80	-1237.60816	-1238.74573	-1238.74573	-1238.94576	-1239.17538	-1239.18470	-1239.37827	-1239.21264	-1239.43458
13.89	-1237.60816	-1238.74572	-1238.74572	-1238.94575	-1239.17538	-1239.18471	-1239.37827	-1239.21264	-1239.43458
13.98	-1237.60815	-1238.74572	-1238.74572	-1238.94575	-1239.17538	-1239.18471	-1239.37827	-1239.21264	-1239.43458
14.08	-1237.60815	-1238.74572	-1238.74572	-1238.94575	-1239.17538	-1239.18471	-1239.37828	-1239.21264	-1239.43458
14.17	-1237.60815	-1238.74572	-1238.74572	-1238.94574	-1239.17537	-1239.18472	-1239.37828	-1239.21265	-1239.43458
14.27	-1237.60814	-1238.74571	-1238.74571	-1238.94574	-1239.17537	-1239.18472	-1239.37828	-1239.21265	-1239.43458
14.36	-1237.60814	-1238.74571	-1238.74571	-1238.94574	-1239.17537	-1239.18473	-1239.37829	-1239.21265	-1239.43458
14.46	-1237.60814	-1238.74571	-1238.74571	-1238.94574	-1239.17537	-1239.18473	-1239.37829	-1239.21265	-1239.43458
14.55	-1237.60814	-1238.74571	-1238.74571	-1238.94574	-1239.17537	-1239.18473	-1239.37829	-1239.21265	-1239.43458
14.65	-1237.60813	-1238.74571	-1238.74571	-1238.94574	-1239.17537	-1239.18474	-1239.37830	-1239.21265	-1239.43458
14.74	-1237.60813	-1238.74571	-1238.74571	-1238.94574	-1239.17537	-1239.18474	-1239.37830	-1239.21265	-1239.43458
14.83	-1237.60813	-1238.74571	-1238.74571	-1238.94574	-1239.17537	-1239.18474	-1239.37830	-1239.21265	-1239.43458
14.93	-1237.60813	-1238.74571	-1238.74571	-1238.94574	-1239.17537	-1239.18475	-1239.37830	-1239.21265	-1239.43458
15.02	-1237.60812	-1238.74571	-1238.74571	-1238.94574	-1239.17537	-1239.18475	-1239.37831	-1239.21265	-1239.43458
15.12	-1237.60812	-1238.74571	-1238.74571	-1238.94574	-1239.17536	-1239.18475	-1239.37831	-1239.21265	-1239.43457
15.21	-1237.60812	-1238.74571	-1238.74571	-1238.94573	-1239.17536	-1239.18476	-1239.37831	-1239.21265	-1239.43457
15.31	-1237.60812	-1238.74571	-1238.74571	-1238.94573	-1239.17536	-1239.18476	-1239.37831	-1239.21265	-1239.43457
15.40	-1237.60812	-1238.74571	-1238.74571	-1238.94573	-1239.17536	-1239.18476	-1239.37831	-1239.21265	-1239.43457
15.50	-1237.60811	-1238.74571	-1238.74571	-1238.94573	-1239.17536	-1239.18476	-1239.37832	-1239.21265	-1239.43457
15.59	-1237.60811	-1238.74570	-1238.74570	-1238.94573	-1239.17536	-1239.18476	-1239.37832	-1239.21265	-1239.43457
15.68	-1237.60811	-1238.74570	-1238.74570	-1238.94573	-1239.17536	-1239.18476	-1239.37832	-1239.21265	-1239.43457
15.78	-1237.60811	-1238.74570	-1238.74570	-1238.94573	-1239.17536	-1239.18476	-1239.37832	-1239.21265	-1239.43457
15.87	-1237.60811	-1238.74570	-1238.74570	-1238.94573	-1239.17536	-1239.18477	-1239.37832	-1239.21265	-1239.43457
15.97	-1237.60811	-1238.74570	-1238.74570	-1238.94573	-1239.17536	-1239.18477	-1239.37832	-1239.21265	-1239.43457
16.06	-1237.60810	-1238.74570	-1238.74570	-1238.94573	-1239.17536	-1239.18477	-1239.37832	-1239.21265	-1239.43457
16.16	-1237.60810	-1238.74570	-1238.74570	-1238.94573	-1239.17536	-1239.18477	-1239.37832	-1239.21265	-1239.43457
16.25	-1237.60810	-1238.74570	-1238.74570	-1238.94573	-1239.17536	-1239.18477	-1239.37833	-1239.21265	-1239.43457
16.35	-1237.60810	-1238.74570	-1238.74570	-1238.94573	-1239.17536	-1239.18477	-1239.37833	-1239.21265	-1239.43457
16.44	-1237.60810	-1238.74570	-1238.74570	-1238.94573	-1239.17536	-1239.18478	-1239.37833	-1239.21265	-1239.43457
16.54	-1237.60810	-1238.74570	-1238.74570	-1238.94573	-1239.17536	-1239.18478	-1239.37833	-1239.21265	-1239.43457
16.63	-1237.60810	-1238.74570	-1238.74570	-1238.94573	-1239.17536	-1239.18478	-1239.37833	-1239.21265	-1239.43457
16.72	-1237.60810	-1238.74570	-1238.74570	-1238.94573	-1239.17536	-1239.18478	-1239.37833	-1239.21265	-1239.43457
16.82	-1237.60810	-1238.74570	-1238.74570	-1238.94573	-1239.17536	-1239.18478	-1239.37834	-1239.21265	-1239.43457
16.91	-1237.60809	-1238.74570	-1238.74570	-1238.94573	-1239.17536	-1239.18479	-1239.37834	-1239.21265	-1239.43457
17.01	-1237.60809	-1238.74570	-1238.74570	-1238.94573	-1239.17536	-1239.18479	-1239.37834	-1239.21265	-1239.43457
17.10	-1237.60809	-1238.74570	-1238.74570	-1238.94573	-1239.17536	-1239.18479	-1239.37834	-1239.21265	-1239.43457
17.20	-1237.60809	-1238.74570	-1238.74570	-1238.94573	-1239.17536	-1239.18479	-1239.37834	-1239.21265	-1239.43457
17.29	-1237.60809	-1238.74570	-1238.74570	-1238.94573	-1239.17536	-1239.18479	-1239.37834	-1239.21265	-1239.43457
17.39	-1237.60809	-1238.74570	-1238.74570	-1238.94573	-1239.17536	-1239.18479	-1239.37834	-1239.21265	-1239.43457
17.48	-1237.60809	-1238.74570	-1238.74570	-1238.94573	-1239.17536	-1239.18479	-1239.37834	-1239.21265	-1239.43457
17.57	-1237.60809	-1238.74570	-1238.74570	-1238.94573	-1239.17536	-1239.18479	-1239.37834	-1239.21265	-1239.43457
17.67	-1237.60809	-1238.74570	-1238.74570	-1238.94573	-1239.17536	-1239.18479	-1239.37834	-1239.21265	-1239.43457
17.76	-1237.60809	-1238.74570	-1238.74570	-1238.94573	-1239.17536	-1239.18479	-1239.37834	-1239.21265	-1239.43457
17.86	-1237.60809	-1238.74570	-1238.74570	-1238.94573	-1239.17536	-1239.18479	-1239.37834	-1239.21265	-1239.43457
17.95	-1237.60809	-1238.74570	-1238.74570	-1238.94573	-1239.17536	-1239.18479	-1239.37834	-1239.21265	-1239.43457
18.05	-1237.60809	-1238.74570	-1238.74570	-1238.94573	-1239.17536	-1239.18479	-1239.37834	-1239.21265	-1239.43457

Table S9: The absolute energies in hartree of the potential energy curve of VSi in the $^6\Sigma^+$ state with a SP-based wave function and nom-CPO active space.

Distance	SP	tpbe	ftpbe	trevpbe	ftrevpbe	tblyp	ftblyp	torelyp	ftorelyp
1.98	-1235.30176	-1236.55209	-1236.74918	-1236.75747	-1236.95742	-1236.96545	-1237.15607	-1237.03092	-1237.24771
2.08	-1235.73582	-1236.98177	-1237.17892	-1237.18827	-1237.38833	-1237.39651	-1237.58704	-1237.45703	-1237.67398
2.17	-1236.07476	-1237.31790	-1237.51518	-1237.52544	-1237.72567	-1237.73391	-1237.92447	-1237.79023	-1238.00746
2.27	-1236.34116	-1237.58286	-1237.78030	-1237.79137	-1237.99180	-1238.00004	-1238.19066	-1238.05299	-1238.27048
2.36	-1236.55253	-1237.79367	-1237.99125	-1238.00309	-1238.20368	-1238.21189	-1238.40252	-1238.26232	-1238.48003
2.46	-1236.72260	-1237.96172	-1238.15946	-1238.17203	-1238.37280	-1238.38061	-1238.57104	-1238.42996	-1238.64782
2.55	-1236.86471	-1238.08980	-1238.29009	-1238.30121	-1238.50550	-1238.50699	-1238.69935	-1238.55694	-1238.78030
2.65	-1236.98513	-1238.20588	-1238.40676	-1238.41805	-1238.62332	-1238.62286	-1238.81569	-1238.67121	-1238.89609
2.74	-1237.08659	-1238.30543	-1238.50656	-1238.51827	-1238.72398	-1238.72240	-1238.91531	-1238.76900	-1238.99448
2.83	-1237.17282	-1238.39165	-1238.59274	-1238.60506	-1238.81079	-1238.80877	-1239.00144	-1238.85362	-1239.07897
2.93	-1237.24664	-1238.46684	-1238.66764	-1238.68074	-1238.88612	-1238.88428	-1239.07641	-1238.92741	-1239.15207
3.02	-1237.31020	-1238.53271	-1238.73302	-1238.74704	-1238.95180	-1238.95060	-1239.14199	-1238.99210	-1239.21569
3.12	-1237.36522	-1238.59117	-1238.79077	-1238.80587	-1239.00972	-1239.00971	-1239.20003	-1239.04957	-1239.27161
3.21	-1237.41620	-1238.64784	-1238.84730	-1238.86264	-1239.06688	-1239.06634	-1239.25628	-1239.10299	-1239.32482
3.31	-1237.45875	-1238.68799	-1238.88686	-1238.90321	-1239.10682	-1239.10680	-1239.29608	-1239.14294	-1239.36395
3.40	-1237.49477	-1238.72158	-1238.91999	-1238.93720	-1239.14030	-1239.14071	-1239.32947	-1239.17631	-1239.39665
3.50	-1237.52509	-1238.74952	-1238.94754	-1238.96553	-1239.16820	-1239.16897	-1239.35731	-1239.20405	-1239.42382
3.59	-1237.55043	-1238.77257	-1238.97025	-1238.98897	-1239.19126	-1239.19235	-1239.38034	-1239.22694	-1239.44621
3.68	-1237.57145	-1238.79141	-1238.98879	-1239.00817	-1239.21012	-1239.21154	-1239.39921	-1239.24565	-1239.46446
3.78	-1237.58873	-1238.80659	-1239.00371	-1239.02369	-1239.22536	-1239.22707	-1239.41449	-1239.26075	-1239.47915
3.87	-1237.60280	-1238.81861	-1239.01550	-1239.03604	-1239.23744	-1239.23944	-1239.42664	-1239.27272	-1239.49074
3.97	-1237.61411	-1238.82788	-1239.02458	-1239.04564	-1239.24681	-1239.24908	-1239.43610	-1239.28198	-1239.49967
4.06	-1237.62307	-1238.83480	-1239.03135	-1239.05286	-1239.25385	-1239.25637	-1239.44325	-1239.28891	-1239.50630
4.16	-1237.63004	-1238.83968	-1239.03612	-1239.05804	-1239.25888	-1239.26160	-1239.44840	-1239.29381	-1239.51097
4.35	-1237.63531	-1238.84277	-1239.03914	-1239.06142	-1239.26216	-1239.26505	-1239.45180	-1239.29695	-1239.51391
4.44	-1237.63917	-1238.84431	-1239.04065	-1239.06324	-1239.26392	-1239.26692	-1239.45369	-1239.29853	-1239.51536
4.54	-1237.64185	-1238.84449	-1239.04086	-1239.06370	-1239.26439	-1239.26743	-1239.45426	-1239.29877	-1239.51553
4.63	-1237.64355	-1238.84348	-1239.03994	-1239.06298	-1239.26373	-1239.26674	-1239.45369	-1239.29783	-1239.51459
4.72	-1237.64444	-1238.84144	-1239.03803	-1239.06122	-1239.26208	-1239.26498	-1239.45212	-1239.29585	-1239.51268
4.82	-1237.64468	-1238.83848	-1239.03526	-1239.05854	-1239.25959	-1239.26229	-1239.44966	-1239.29296	-1239.50995
4.91	-1237.64440	-1238.83466	-1239.03170	-1239.05501	-1239.25633	-1239.25872	-1239.44640	-1239.28921	-1239.50646
5.01	-1237.64373	-1238.82916	-1239.02663	-1239.04985	-1239.25158	-1239.25345	-1239.44162	-1239.28379	-1239.50153
5.10	-1237.64286	-1238.82068	-1239.01866	-1239.04172	-1239.24397	-1239.24518	-1239.43397	-1239.27539	-1239.49379
5.20	-1237.64179	-1238.81538	-1239.01355	-1239.03667	-1239.23912	-1239.24010	-1239.42914	-1239.27023	-1239.48890
5.29	-1237.64048	-1238.81094	-1239.00925	-1239.03246	-1239.23505	-1239.23589	-1239.42511	-1239.26597	-1239.48483
5.39	-1237.63895	-1238.80688	-1239.00530	-1239.02861	-1239.23131	-1239.23206	-1239.42144	-1239.26211	-1239.48113
5.48	-1237.63723	-1238.80307	-1239.00159	-1239.02498	-1239.22779	-1239.22848	-1239.41800	-1239.25850	-1239.47768
5.57	-1237.63536	-1238.79943	-1238.99804	-1239.02153	-1239.22442	-1239.22509	-1239.41474	-1239.25508	-1239.47441
5.67	-1237.63338	-1238.79594	-1238.99464	-1239.01820	-1239.22119	-1239.22186	-1239.41164	-1239.25182	-1239.47129
5.76	-1237.63130	-1238.79258	-1238.99138	-1239.01500	-1239.21807	-1239.21877	-1239.40868	-1239.24871	-1239.46833
5.86	-1237.62916	-1238.78935	-1238.98824	-1239.01191	-1239.21507	-1239.21582	-1239.40587	-1239.24572	-1239.46550
5.95	-1237.62699	-1238.78625	-1238.98524	-1239.00895	-1239.21219	-1239.21300	-1239.40321	-1239.24288	-1239.46281
6.05	-1237.62480	-1238.78328	-1238.98236	-1239.00610	-1239.20943	-1239.21034	-1239.40070	-1239.24016	-1239.46028
6.14	-1237.62260	-1238.78045	-1238.97963	-1239.00339	-1239.20680	-1239.20782	-1239.39836	-1239.23759	-1239.45789
6.24	-1237.62044	-1238.77775	-1238.97704	-1239.00080	-1239.20430	-1239.20546	-1239.39618	-1239.23517	-1239.45566
6.33	-1237.61831	-1238.77518	-1238.97457	-1238.99833	-1239.20191	-1239.20324	-1239.39416	-1239.23287	-1239.45357
6.43	-1237.61623	-1238.77274	-1238.97224	-1238.99598	-1239.19964	-1239.20118	-1239.39231	-1239.23070	-1239.45162
6.52	-1237.61423	-1238.77041	-1238.97002	-1238.99374	-1239.19748	-1239.19925	-1239.39061	-1239.22866	-1239.44980
6.61	-1237.61232	-1238.76821	-1238.96793	-1238.99161	-1239.19543	-1239.19748	-1239.38908	-1239.22674	-1239.44812
6.71	-1237.61051	-1238.76611	-1238.96593	-1238.98958	-1239.19346	-1239.19583	-1239.38769	-1239.22493	-1239.44654
6.80	-1237.60883	-1238.76409	-1238.96402	-1238.98763	-1239.19157	-1239.19431	-1239.38644	-1239.22321	-1239.44505
6.90	-1237.60728	-1238.76213	-1238.96217	-1238.98573	-1239.18973	-1239.19288	-1239.38531	-1239.22155	-1239.44364

Table S9: The absolute energies in hartree of the potential energy curve of VSi in the $^6\Sigma^+$ state with a SP-based wave function and nom-CPO active space.

Distance	SP	tpbe	ftpbe	trevpbe	ftrevpbe	tblyp	ftblyp	torelyp	ftorelyp
6.99	-1237.60590	-1238.76022	-1238.96037	-1238.98387	-1239.18792	-1239.19153	-1239.38426	-1239.21994	-1239.44227
7.09	-1237.60468	-1238.75837	-1238.95862	-1238.98207	-1239.18618	-1239.19026	-1239.38328	-1239.21839	-1239.44094
7.18	-1237.60364	-1238.75661	-1238.95696	-1238.98036	-1239.18454	-1239.18906	-1239.38235	-1239.21693	-1239.43969
7.28	-1237.60275	-1238.75499	-1238.95543	-1238.97880	-1239.18304	-1239.18795	-1239.38146	-1239.21560	-1239.43853
7.37	-1237.60198	-1238.75353	-1238.95405	-1238.97739	-1239.18170	-1239.18694	-1239.38062	-1239.21441	-1239.43747
7.46	-1237.60133	-1238.75224	-1238.95281	-1238.97616	-1239.18051	-1239.18603	-1239.37984	-1239.21337	-1239.43653
7.56	-1237.60076	-1238.75108	-1238.95169	-1238.97506	-1239.17946	-1239.18520	-1239.37911	-1239.21244	-1239.43568
7.65	-1237.60027	-1238.75005	-1238.95069	-1238.97409	-1239.17851	-1239.18446	-1239.37844	-1239.21163	-1239.43491
7.75	-1237.59983	-1238.74912	-1238.94978	-1238.97322	-1239.17766	-1239.18379	-1239.37782	-1239.21091	-1239.43423
7.84	-1237.59944	-1238.74828	-1238.94895	-1238.97245	-1239.17690	-1239.18319	-1239.37725	-1239.21027	-1239.43362
7.94	-1237.59909	-1238.74752	-1238.94820	-1238.97175	-1239.17621	-1239.18264	-1239.37674	-1239.20971	-1239.43307
8.03	-1237.59877	-1238.74683	-1238.94752	-1238.97112	-1239.17559	-1239.18215	-1239.37627	-1239.20920	-1239.43257
8.13	-1237.59849	-1238.74620	-1238.94689	-1238.97055	-1239.17502	-1239.18170	-1239.37583	-1239.20876	-1239.43214
8.22	-1237.59823	-1238.74563	-1238.94632	-1238.97004	-1239.17451	-1239.18129	-1239.37543	-1239.20837	-1239.43175
8.31	-1237.59800	-1238.74512	-1238.94580	-1238.96958	-1239.17405	-1239.18092	-1239.37508	-1239.20803	-1239.43140
8.41	-1237.59779	-1238.74465	-1238.94533	-1238.96916	-1239.17363	-1239.18060	-1239.37475	-1239.20773	-1239.43109
8.50	-1237.59760	-1238.74422	-1238.94490	-1238.96879	-1239.17325	-1239.18030	-1239.37445	-1239.20746	-1239.43082
8.60	-1237.59742	-1238.74383	-1238.94450	-1238.96846	-1239.17291	-1239.18004	-1239.37418	-1239.20724	-1239.43059
8.69	-1237.59726	-1238.74348	-1238.94414	-1238.96816	-1239.17260	-1239.17979	-1239.37394	-1239.20704	-1239.43038
8.79	-1237.59712	-1238.74316	-1238.94382	-1238.96789	-1239.17233	-1239.17958	-1239.37372	-1239.20688	-1239.43020
8.88	-1237.59698	-1238.74287	-1238.94351	-1238.96765	-1239.17208	-1239.17938	-1239.37352	-1239.20673	-1239.43004
8.98	-1237.59686	-1238.74260	-1238.94324	-1238.96744	-1239.17185	-1239.17921	-1239.37333	-1239.20661	-1239.42990
9.07	-1237.59675	-1238.74236	-1238.94299	-1238.96724	-1239.17165	-1239.17905	-1239.37317	-1239.20650	-1239.42978
9.17	-1237.59665	-1238.74214	-1238.94276	-1238.96707	-1239.17147	-1239.17891	-1239.37302	-1239.20642	-1239.42968
9.26	-1237.59655	-1238.74194	-1238.94255	-1238.96692	-1239.17130	-1239.17879	-1239.37288	-1239.20635	-1239.42959
9.35	-1237.59647	-1238.74177	-1238.94237	-1238.96678	-1239.17116	-1239.17868	-1239.37276	-1239.20629	-1239.42952
9.45	-1237.59639	-1238.74161	-1238.94220	-1238.96667	-1239.17103	-1239.17858	-1239.37266	-1239.20624	-1239.42946
9.54	-1237.59631	-1238.74146	-1238.94204	-1238.96656	-1239.17091	-1239.17849	-1239.37256	-1239.20621	-1239.42941
9.64	-1237.59624	-1238.74133	-1238.94190	-1238.96647	-1239.17081	-1239.17842	-1239.37247	-1239.20618	-1239.42937
9.73	-1237.59618	-1238.74121	-1238.94178	-1238.96639	-1239.17072	-1239.17835	-1239.37240	-1239.20616	-1239.42933
9.83	-1237.59613	-1238.74110	-1238.94166	-1238.96632	-1239.17064	-1239.17829	-1239.37233	-1239.20615	-1239.42930
9.92	-1237.59607	-1238.74100	-1238.94155	-1238.96626	-1239.17056	-1239.17824	-1239.37227	-1239.20614	-1239.42928
10.02	-1237.59602	-1238.74092	-1238.94146	-1238.96620	-1239.17050	-1239.17820	-1239.37221	-1239.20614	-1239.42926
10.11	-1237.59598	-1238.74083	-1238.94137	-1238.96615	-1239.17044	-1239.17816	-1239.37217	-1239.20614	-1239.42924
10.20	-1237.59594	-1238.74076	-1238.94128	-1238.96611	-1239.17038	-1239.17813	-1239.37212	-1239.20614	-1239.42923
10.30	-1237.59590	-1238.74070	-1238.94121	-1238.96607	-1239.17033	-1239.17811	-1239.37209	-1239.20615	-1239.42922
10.39	-1237.59586	-1238.74064	-1238.94114	-1238.96604	-1239.17029	-1239.17809	-1239.37206	-1239.20616	-1239.42921
10.49	-1237.59583	-1238.74059	-1238.94109	-1238.96601	-1239.17026	-1239.17807	-1239.37203	-1239.20617	-1239.42921
10.58	-1237.59580	-1238.74054	-1238.94103	-1238.96599	-1239.17023	-1239.17805	-1239.37201	-1239.20618	-1239.42921
10.68	-1237.59577	-1238.74050	-1238.94099	-1238.96597	-1239.17020	-1239.17804	-1239.37199	-1239.20619	-1239.42921
10.77	-1237.59574	-1238.74046	-1238.94094	-1238.96596	-1239.17018	-1239.17803	-1239.37198	-1239.20621	-1239.42921
10.87	-1237.59572	-1238.74043	-1238.94090	-1238.96594	-1239.17016	-1239.17803	-1239.37196	-1239.20622	-1239.42921
10.96	-1237.59570	-1238.74039	-1238.94086	-1238.96593	-1239.17014	-1239.17802	-1239.37195	-1239.20623	-1239.42921
11.05	-1237.59567	-1238.74036	-1238.94083	-1238.96592	-1239.17012	-1239.17802	-1239.37194	-1239.20624	-1239.42921
11.15	-1237.59565	-1238.74034	-1238.94080	-1238.96591	-1239.17010	-1239.17802	-1239.37193	-1239.20625	-1239.42921
11.24	-1237.59563	-1238.74031	-1238.94077	-1238.96590	-1239.17008	-1239.17802	-1239.37193	-1239.20626	-1239.42921
11.34	-1237.59562	-1238.74029	-1238.94074	-1238.96589	-1239.17006	-1239.17802	-1239.37193	-1239.20627	-1239.42921
11.43	-1237.59560	-1238.74027	-1238.94071	-1238.96588	-1239.17005	-1239.17803	-1239.37193	-1239.20629	-1239.42921
11.53	-1237.59558	-1238.74025	-1238.94069	-1238.96588	-1239.17004	-1239.17803	-1239.37193	-1239.20630	-1239.42921
11.62	-1237.59557	-1238.74023	-1238.94067	-1238.96587	-1239.17003	-1239.17804	-1239.37193	-1239.20631	-1239.42921
11.72	-1237.59556	-1238.74022	-1238.94065	-1238.96587	-1239.17002	-1239.17804	-1239.37193	-1239.20632	-1239.42921
11.81	-1237.59554	-1238.74021	-1238.94064	-1238.96587	-1239.17002	-1239.17805	-1239.37193	-1239.20633	-1239.42921

Table S9: The absolute energies in hartree of the potential energy curve of VSi in the $^6\Sigma^+$ state with a SP-based wave function and nom-CPO active space.

Distance	SP	tpbe	ftpbe	trevpbe	ftrevpbe	tblyp	ftblyp	torelyp	ftorelyp
11.91	-1237.59553	-1238.74020	-1238.94062	-1238.96587	-1239.17001	-1239.17805	-1239.37193	-1239.20633	-1239.42921
12.00	-1237.59552	-1238.74019	-1238.94061	-1238.96586	-1239.17000	-1239.17806	-1239.37193	-1239.20634	-1239.42921
12.09	-1237.59551	-1238.74017	-1238.94060	-1238.96586	-1239.17000	-1239.17807	-1239.37193	-1239.20635	-1239.42921
12.19	-1237.59550	-1238.74016	-1238.94058	-1238.96586	-1239.16999	-1239.17807	-1239.37193	-1239.20635	-1239.42921
12.28	-1237.59549	-1238.74016	-1238.94057	-1238.96586	-1239.16999	-1239.17808	-1239.37193	-1239.20635	-1239.42921
12.38	-1237.59548	-1238.74015	-1238.94056	-1238.96586	-1239.16998	-1239.17809	-1239.37193	-1239.20636	-1239.42921
12.47	-1237.59547	-1238.74014	-1238.94055	-1238.96585	-1239.16998	-1239.17809	-1239.37193	-1239.20636	-1239.42921
12.57	-1237.59546	-1238.74013	-1238.94054	-1238.96585	-1239.16997	-1239.17810	-1239.37193	-1239.20637	-1239.42921
12.66	-1237.59546	-1238.74012	-1238.94053	-1238.96585	-1239.16996	-1239.17811	-1239.37193	-1239.20637	-1239.42921
12.76	-1237.59545	-1238.74012	-1238.94052	-1238.96585	-1239.16996	-1239.17811	-1239.37193	-1239.20638	-1239.42921
12.85	-1237.59544	-1238.74011	-1238.94052	-1238.96585	-1239.16996	-1239.17812	-1239.37193	-1239.20638	-1239.42921
12.94	-1237.59544	-1238.74011	-1238.94051	-1238.96585	-1239.16995	-1239.17813	-1239.37193	-1239.20638	-1239.42921
13.04	-1237.59543	-1238.74011	-1238.94051	-1238.96585	-1239.16995	-1239.17814	-1239.37193	-1239.20639	-1239.42921
13.13	-1237.59542	-1238.74010	-1238.94050	-1238.96585	-1239.16995	-1239.17814	-1239.37193	-1239.20639	-1239.42920
13.23	-1237.59542	-1238.74010	-1238.94050	-1238.96585	-1239.16995	-1239.17815	-1239.37193	-1239.20639	-1239.42920
13.32	-1237.59541	-1238.74010	-1238.94049	-1238.96585	-1239.16994	-1239.17816	-1239.37193	-1239.20639	-1239.42920
13.42	-1237.59541	-1238.74009	-1238.94049	-1238.96585	-1239.16994	-1239.17816	-1239.37193	-1239.20640	-1239.42920
13.51	-1237.59540	-1238.74009	-1238.94049	-1238.96585	-1239.16994	-1239.17817	-1239.37193	-1239.20640	-1239.42920
13.61	-1237.59540	-1238.74009	-1238.94048	-1238.96585	-1239.16994	-1239.17817	-1239.37193	-1239.20640	-1239.42920
13.70	-1237.59540	-1238.74008	-1238.94048	-1238.96584	-1239.16994	-1239.17818	-1239.37193	-1239.20640	-1239.42920
13.80	-1237.59539	-1238.74008	-1238.94048	-1238.96584	-1239.16993	-1239.17818	-1239.37193	-1239.20640	-1239.42919
13.89	-1237.59539	-1238.74008	-1238.94048	-1238.96584	-1239.16993	-1239.17819	-1239.37193	-1239.20640	-1239.42919
13.98	-1237.59538	-1238.74008	-1238.94047	-1238.96584	-1239.16993	-1239.17819	-1239.37193	-1239.20640	-1239.42919
14.08	-1237.59538	-1238.74007	-1238.94047	-1238.96584	-1239.16993	-1239.17820	-1239.37193	-1239.20640	-1239.42919
14.17	-1237.59538	-1238.74007	-1238.94046	-1238.96584	-1239.16993	-1239.17820	-1239.37193	-1239.20640	-1239.42919
14.27	-1237.59537	-1238.74007	-1238.94046	-1238.96584	-1239.16992	-1239.17820	-1239.37193	-1239.20640	-1239.42919
14.36	-1237.59537	-1238.74007	-1238.94046	-1238.96584	-1239.16992	-1239.17821	-1239.37193	-1239.20640	-1239.42919
14.46	-1237.59537	-1238.74007	-1238.94046	-1238.96584	-1239.16992	-1239.17821	-1239.37193	-1239.20640	-1239.42919
14.55	-1237.59536	-1238.74007	-1238.94046	-1238.96584	-1239.16992	-1239.17822	-1239.37193	-1239.20640	-1239.42919
14.65	-1237.59536	-1238.74007	-1238.94046	-1238.96584	-1239.16992	-1239.17822	-1239.37193	-1239.20640	-1239.42919
14.74	-1237.59536	-1238.74007	-1238.94046	-1238.96584	-1239.16992	-1239.17823	-1239.37193	-1239.20641	-1239.42919
14.83	-1237.59536	-1238.74007	-1238.94046	-1238.96584	-1239.16992	-1239.17823	-1239.37193	-1239.20641	-1239.42919
14.93	-1237.59535	-1238.74007	-1238.94046	-1238.96584	-1239.16992	-1239.17823	-1239.37193	-1239.20641	-1239.42919
15.02	-1237.59535	-1238.74007	-1238.94046	-1238.96584	-1239.16992	-1239.17824	-1239.37193	-1239.20641	-1239.42919
15.12	-1237.59535	-1238.74007	-1238.94046	-1238.96584	-1239.16992	-1239.17824	-1239.37193	-1239.20641	-1239.42919
15.21	-1237.59535	-1238.74007	-1238.94046	-1238.96584	-1239.16992	-1239.17824	-1239.37193	-1239.20641	-1239.42919
15.31	-1237.59535	-1238.74006	-1238.94045	-1238.96584	-1239.16992	-1239.17825	-1239.37193	-1239.20641	-1239.42918
15.40	-1237.59534	-1238.74006	-1238.94045	-1238.96584	-1239.16992	-1239.17825	-1239.37193	-1239.20641	-1239.42918
15.50	-1237.59534	-1238.74006	-1238.94045	-1238.96584	-1239.16992	-1239.17825	-1239.37193	-1239.20641	-1239.42918
15.59	-1237.59534	-1238.74006	-1238.94045	-1238.96584	-1239.16992	-1239.17825	-1239.37193	-1239.20641	-1239.42918
15.68	-1237.59534	-1238.74006	-1238.94045	-1238.96584	-1239.16991	-1239.17825	-1239.37193	-1239.20641	-1239.42918
15.78	-1237.59534	-1238.74006	-1238.94045	-1238.96584	-1239.16991	-1239.17826	-1239.37193	-1239.20641	-1239.42918
15.87	-1237.59534	-1238.74006	-1238.94045	-1238.96583	-1239.16991	-1239.17826	-1239.37193	-1239.20641	-1239.42918
15.97	-1237.59533	-1238.74006	-1238.94045	-1238.96583	-1239.16991	-1239.17826	-1239.37193	-1239.20641	-1239.42918
16.06	-1237.59533	-1238.74006	-1238.94045	-1238.96583	-1239.16991	-1239.17826	-1239.37193	-1239.20641	-1239.42918
16.16	-1237.59533	-1238.74006	-1238.94045	-1238.96583	-1239.16991	-1239.17826	-1239.37193	-1239.20641	-1239.42918
16.25	-1237.59533	-1238.74006	-1238.94045	-1238.96583	-1239.16991	-1239.17826	-1239.37193	-1239.20641	-1239.42918
16.35	-1237.59533	-1238.74006	-1238.94045	-1238.96583	-1239.16991	-1239.17827	-1239.37193	-1239.20641	-1239.42918
16.44	-1237.59533	-1238.74006	-1238.94045	-1238.96583	-1239.16991	-1239.17827	-1239.37193	-1239.20641	-1239.42918
16.54	-1237.59533	-1238.74006	-1238.94045	-1238.96584	-1239.16991	-1239.17827	-1239.37193	-1239.20641	-1239.42918
16.63	-1237.59533	-1238.74006	-1238.94045	-1238.96584	-1239.16991	-1239.17827	-1239.37193	-1239.20641	-1239.42918
16.72	-1237.59532	-1238.74006	-1238.94045	-1238.96584	-1239.16991	-1239.17828	-1239.37193	-1239.20641	-1239.42918

Table S9: The absolute energies in hartree of the potential energy curve of VSi in the $^6\Sigma^+$ state with a SP-based wave function and nom-CPO active space.

Distance	SP	tpbe	ftpbe	trevpbe	ftrevpbe	tblyp	ftblyp	torelyp	ftorelyp
16.82	-1237.59532	-1238.74006	-1238.94045	-1238.96584	-1239.16991	-1239.17828	-1239.37193	-1239.20641	-1239.42918
16.91	-1237.59532	-1238.74006	-1238.94045	-1238.96584	-1239.16992	-1239.17828	-1239.37193	-1239.20641	-1239.42919
17.01	-1237.59532	-1238.74006	-1238.94045	-1238.96584	-1239.16992	-1239.17828	-1239.37193	-1239.20641	-1239.42919
17.10	-1237.59532	-1238.74007	-1238.94045	-1238.96584	-1239.16992	-1239.17828	-1239.37193	-1239.20641	-1239.42919
17.20	-1237.59532	-1238.74007	-1238.94045	-1238.96584	-1239.16992	-1239.17829	-1239.37193	-1239.20642	-1239.42919
17.29	-1237.59532	-1238.74007	-1238.94045	-1238.96584	-1239.16992	-1239.17829	-1239.37193	-1239.20642	-1239.42919
17.39	-1237.59532	-1238.74007	-1238.94045	-1238.96584	-1239.16992	-1239.17829	-1239.37193	-1239.20641	-1239.42919
17.48	-1237.59532	-1238.74006	-1238.94045	-1238.96584	-1239.16992	-1239.17829	-1239.37193	-1239.20641	-1239.42919
17.57	-1237.59532	-1238.74006	-1238.94045	-1238.96584	-1239.16992	-1239.17829	-1239.37193	-1239.20641	-1239.42919
17.67	-1237.59532	-1238.74006	-1238.94045	-1238.96584	-1239.16991	-1239.17829	-1239.37193	-1239.20641	-1239.42918
17.76	-1237.59532	-1238.74006	-1238.94045	-1238.96584	-1239.16991	-1239.17829	-1239.37193	-1239.20641	-1239.42918
17.86	-1237.59531	-1238.74006	-1238.94045	-1238.96584	-1239.16991	-1239.17829	-1239.37193	-1239.20641	-1239.42918
17.95	-1237.59531	-1238.74006	-1238.94045	-1238.96584	-1239.16991	-1239.17829	-1239.37193	-1239.20641	-1239.42918
18.05	-1237.59531	-1238.74006	-1238.94045	-1238.96584	-1239.16991	-1239.17829	-1239.37193	-1239.20641	-1239.42918

Table S10: The absolute energies in hartree of the potential energy curve of VSi in the $^2\Sigma^+$ state with a SP-based wave function and nom-CPO active space.

Distance	SP	tpbe	ftpbe	trevpbe	ftrevpbe	tblyp	ftblyp	torelyp	ftorelyp
1.98	-1235.49398	-1236.75560	-1236.96452	-1236.96443	-1237.17374	-1237.17019	-1237.17019	-1237.23379	-1237.46445
2.08	-1235.91926	-1237.17342	-1237.38147	-1237.38341	-1237.59184	-1237.58887	-1237.58887	-1237.64839	-1237.87807
2.17	-1236.24875	-1237.49740	-1237.70459	-1237.70847	-1237.91605	-1237.91973	-1237.91973	-1237.96984	-1238.19856
2.27	-1236.52305	-1237.76754	-1237.97170	-1237.97826	-1238.18269	-1238.18307	-1238.18307	-1238.23924	-1238.46418
2.36	-1236.73328	-1237.97435	-1238.17764	-1238.18601	-1238.38962	-1238.39012	-1238.39012	-1238.44436	-1238.66830
2.46	-1236.89913	-1238.13782	-1238.34034	-1238.35034	-1238.55327	-1238.55394	-1238.55394	-1238.60642	-1238.82953
2.55	-1237.03076	-1238.26814	-1238.46991	-1238.48147	-1238.68375	-1238.68471	-1238.68471	-1238.73563	-1238.95800
2.65	-1237.13715	-1238.37417	-1238.57497	-1238.58810	-1238.79056	-1238.79113	-1238.79113	-1238.84073	-1239.06492
2.74	-1237.22502	-1238.45951	-1238.66034	-1238.67433	-1238.87696	-1238.87662	-1238.87662	-1238.92545	-1239.14990
2.83	-1237.29692	-1238.53102	-1238.73188	-1238.74668	-1238.94944	-1238.94832	-1238.94832	-1238.99663	-1239.22131
2.93	-1237.35593	-1238.59121	-1238.79195	-1238.80763	-1239.01028	-1239.00867	-1239.00867	-1239.05669	-1239.28121
3.02	-1237.40450	-1238.64232	-1238.84283	-1238.85947	-1239.06181	-1239.05993	-1239.05993	-1239.10796	-1239.33200
3.12	-1237.45009	-1238.68333	-1238.88398	-1238.90151	-1239.10383	-1239.10056	-1239.10056	-1239.14956	-1239.37328
3.21	-1237.48635	-1238.71842	-1238.91897	-1238.93745	-1239.13948	-1239.13549	-1239.13549	-1239.18471	-1239.40742
3.31	-1237.51710	-1238.74667	-1238.94708	-1238.96633	-1239.16808	-1239.16382	-1239.16382	-1239.21259	-1239.43440
3.40	-1237.54290	-1238.76931	-1238.96969	-1238.98938	-1239.19110	-1239.18677	-1239.18677	-1239.23458	-1239.45591
3.50	-1237.56436	-1238.78730	-1238.98768	-1239.00767	-1239.20944	-1239.20523	-1239.20523	-1239.25179	-1239.47285
3.59	-1237.58204	-1238.80138	-1239.00175	-1239.02196	-1239.22384	-1239.21985	-1239.21985	-1239.26504	-1239.48595
3.68	-1237.59651	-1238.81214	-1239.01251	-1239.03288	-1239.23492	-1239.23119	-1239.23119	-1239.27494	-1239.49579
3.78	-1237.60828	-1238.82009	-1239.02048	-1239.04094	-1239.24319	-1239.23973	-1239.23973	-1239.28204	-1239.50288
3.87	-1237.61781	-1238.82564	-1239.02607	-1239.04659	-1239.24911	-1239.24586	-1239.24586	-1239.28674	-1239.50764
3.97	-1237.62550	-1238.82920	-1239.02971	-1239.05021	-1239.25307	-1239.24995	-1239.24995	-1239.28945	-1239.51050
4.06	-1237.63170	-1238.83113	-1239.03176	-1239.05220	-1239.25546	-1239.25238	-1239.25238	-1239.29057	-1239.51184
4.16	-1237.63667	-1238.83180	-1239.03257	-1239.05292	-1239.25662	-1239.25347	-1239.25347	-1239.29048	-1239.51204
4.35	-1237.64065	-1238.83151	-1239.03243	-1239.05270	-1239.25684	-1239.25355	-1239.25355	-1239.28952	-1239.51140
4.44	-1237.64377	-1238.83055	-1239.03159	-1239.05182	-1239.25635	-1239.25290	-1239.25290	-1239.28799	-1239.51016
4.54	-1237.64618	-1238.82907	-1239.03019	-1239.05043	-1239.25530	-1239.25172	-1239.25172	-1239.28604	-1239.50848
4.63	-1237.64798	-1238.82716	-1239.02831	-1239.04862	-1239.25374	-1239.25007	-1239.25007	-1239.28376	-1239.50639
4.72	-1237.64923	-1238.82504	-1239.02618	-1239.04663	-1239.25190	-1239.24820	-1239.24820	-1239.28138	-1239.50412
4.82	-1237.64998	-1238.82285	-1239.02395	-1239.04457	-1239.24994	-1239.24626	-1239.24626	-1239.27904	-1239.50181
4.91	-1237.65026	-1238.82059	-1239.02163	-1239.04246	-1239.24787	-1239.24425	-1239.24425	-1239.27670	-1239.49949
5.01	-1237.65012	-1238.81826	-1239.01925	-1239.04028	-1239.24572	-1239.24216	-1239.24216	-1239.27436	-1239.49711

Table S10: The absolute energies in hartree of the potential energy curve of VSi in the $^2\Sigma^+$ state with a SP-based wave function and nom-CPO active space.

Distance	SP	tpbe	ftpbe	trevpbe	ftrevpbe	tblyp	ftblyp	torelyp	ftorelyp
5.10	-1237.64963	-1238.81589	-1239.01680	-1239.03805	-1239.24347	-1239.24004	-1239.24004	-1239.27200	-1239.49469
5.20	-1237.64881	-1238.81345	-1239.01427	-1239.03574	-1239.24112	-1239.23785	-1239.23785	-1239.26962	-1239.49223
5.29	-1237.64773	-1238.81095	-1239.01168	-1239.03339	-1239.23871	-1239.23564	-1239.23564	-1239.26721	-1239.48974
5.39	-1237.64643	-1238.80844	-1239.00907	-1239.03100	-1239.23626	-1239.23341	-1239.23341	-1239.26480	-1239.48724
5.48	-1237.64495	-1238.80591	-1239.00645	-1239.02860	-1239.23377	-1239.23117	-1239.23117	-1239.26240	-1239.48472
5.57	-1237.64332	-1238.80339	-1239.00382	-1239.02620	-1239.23127	-1239.22894	-1239.22894	-1239.26002	-1239.48220
5.67	-1237.64158	-1238.80090	-1239.00121	-1239.02382	-1239.22877	-1239.22675	-1239.22675	-1239.25768	-1239.47974
5.76	-1237.63975	-1238.79844	-1238.99865	-1239.02147	-1239.22631	-1239.22461	-1239.22461	-1239.25538	-1239.47733
5.86	-1237.63787	-1238.79602	-1238.99613	-1239.01915	-1239.22388	-1239.22252	-1239.22252	-1239.25313	-1239.47497
5.95	-1237.63596	-1238.79366	-1238.99366	-1239.01689	-1239.22148	-1239.22050	-1239.22050	-1239.25096	-1239.47266
6.05	-1237.63404	-1238.79138	-1238.99127	-1239.01469	-1239.21914	-1239.21857	-1239.21857	-1239.24886	-1239.47043
6.14	-1237.63213	-1238.78916	-1238.98895	-1239.01256	-1239.21687	-1239.21672	-1239.21672	-1239.24683	-1239.46830
6.24	-1237.63024	-1238.78702	-1238.98672	-1239.01049	-1239.21468	-1239.21495	-1239.21495	-1239.24489	-1239.46628
6.33	-1237.62841	-1238.78495	-1238.98458	-1239.00849	-1239.21257	-1239.21329	-1239.21329	-1239.24303	-1239.46434
6.43	-1237.62662	-1238.78296	-1238.98253	-1239.00657	-1239.21054	-1239.21172	-1239.21172	-1239.24126	-1239.46251
6.52	-1237.62491	-1238.78104	-1238.98056	-1239.00471	-1239.20859	-1239.21022	-1239.21022	-1239.23956	-1239.46077
6.61	-1237.62329	-1238.77919	-1238.97867	-1239.00292	-1239.20671	-1239.20882	-1239.20882	-1239.23795	-1239.45913
6.71	-1237.62174	-1238.77740	-1238.97687	-1239.00118	-1239.20492	-1239.20748	-1239.20748	-1239.23640	-1239.45759
6.80	-1237.62030	-1238.77567	-1238.97514	-1238.99951	-1239.20319	-1239.20621	-1239.20621	-1239.23492	-1239.45613
6.90	-1237.61895	-1238.77399	-1238.97346	-1238.99788	-1239.20153	-1239.20501	-1239.20501	-1239.23349	-1239.45473
6.99	-1237.61770	-1238.77236	-1238.97185	-1238.99631	-1239.19992	-1239.20384	-1239.20384	-1239.23212	-1239.45339
7.09	-1237.61655	-1238.77079	-1238.97029	-1238.99478	-1239.19837	-1239.20272	-1239.20272	-1239.23080	-1239.45210
7.18	-1237.61549	-1238.76926	-1238.96879	-1238.99331	-1239.19688	-1239.20164	-1239.20164	-1239.22954	-1239.45087
7.28	-1237.61452	-1238.76780	-1238.96735	-1238.99190	-1239.19546	-1239.20060	-1239.20060	-1239.22833	-1239.44970
7.37	-1237.61364	-1238.76640	-1238.96598	-1238.99056	-1239.19410	-1239.19961	-1239.19961	-1239.22719	-1239.44859
7.46	-1237.61284	-1238.76507	-1238.96467	-1238.98929	-1239.19282	-1239.19866	-1239.19866	-1239.22611	-1239.44754
7.56	-1237.61211	-1238.76381	-1238.96344	-1238.98809	-1239.19161	-1239.19776	-1239.19776	-1239.22510	-1239.44655
7.65	-1237.61145	-1238.76262	-1238.96227	-1238.98696	-1239.19048	-1239.19691	-1239.19691	-1239.22416	-1239.44563
7.75	-1237.61085	-1238.76150	-1238.96117	-1238.98590	-1239.18941	-1239.19610	-1239.19610	-1239.22329	-1239.44476
7.84	-1237.61031	-1238.76046	-1238.96014	-1238.98492	-1239.18842	-1239.19534	-1239.19534	-1239.22248	-1239.44395
7.94	-1237.60983	-1238.75948	-1238.95918	-1238.98400	-1239.18750	-1239.19464	-1239.19464	-1239.22174	-1239.44321
8.03	-1237.60938	-1238.75858	-1238.95829	-1238.98316	-1239.18665	-1239.19398	-1239.19398	-1239.22106	-1239.44253
8.13	-1237.60898	-1238.75775	-1238.95747	-1238.98239	-1239.18587	-1239.19337	-1239.19337	-1239.22045	-1239.44191
8.22	-1237.60862	-1238.75699	-1238.95672	-1238.98169	-1239.18516	-1239.19282	-1239.19282	-1239.21990	-1239.44135
8.31	-1237.60830	-1238.75629	-1238.95602	-1238.98105	-1239.18451	-1239.19231	-1239.19231	-1239.21941	-1239.44085
8.41	-1237.60800	-1238.75565	-1238.95539	-1238.98047	-1239.18392	-1239.19184	-1239.19184	-1239.21897	-1239.44040
8.50	-1237.60773	-1238.75506	-1238.95480	-1238.97994	-1239.18338	-1239.19142	-1239.19142	-1239.21858	-1239.43999
8.60	-1237.60749	-1238.75453	-1238.95428	-1238.97946	-1239.18290	-1239.19103	-1239.19103	-1239.21824	-1239.43963
8.69	-1237.60727	-1238.75404	-1238.95379	-1238.97903	-1239.18246	-1239.19068	-1239.19068	-1239.21793	-1239.43931
8.79	-1237.60707	-1238.75360	-1238.95335	-1238.97865	-1239.18206	-1239.19036	-1239.19036	-1239.21767	-1239.43902
8.88	-1237.60689	-1238.75319	-1238.95295	-1238.97830	-1239.18170	-1239.19008	-1239.19008	-1239.21743	-1239.43877
8.98	-1237.60673	-1238.75283	-1238.95258	-1238.97799	-1239.18138	-1239.18982	-1239.18982	-1239.21723	-1239.43855
9.07	-1237.60658	-1238.75250	-1238.95225	-1238.97771	-1239.18109	-1239.18959	-1239.18959	-1239.21706	-1239.43836
9.17	-1237.60645	-1238.75221	-1238.95196	-1238.97746	-1239.18083	-1239.18938	-1239.18938	-1239.21691	-1239.43820
9.26	-1237.60632	-1238.75194	-1238.95169	-1238.97725	-1239.18061	-1239.18920	-1239.18920	-1239.21678	-1239.43806
9.35	-1237.60621	-1238.75171	-1238.95145	-1238.97706	-1239.18041	-1239.18904	-1239.18904	-1239.21668	-1239.43794
9.45	-1237.60611	-1238.75149	-1238.95123	-1238.97689	-1239.18023	-1239.18890	-1239.18890	-1239.21659	-1239.43784
9.54	-1237.60602	-1238.75130	-1238.95104	-1238.97674	-1239.18007	-1239.18877	-1239.18877	-1239.21652	-1239.43776
9.64	-1237.60593	-1238.75112	-1238.95086	-1238.97660	-1239.17993	-1239.18865	-1239.18865	-1239.21646	-1239.43768
9.73	-1237.60585	-1238.75096	-1238.95070	-1238.97648	-1239.17980	-1239.18856	-1239.18856	-1239.21641	-1239.43762
9.83	-1237.60578	-1238.75082	-1238.95055	-1238.97637	-1239.17968	-1239.18846	-1239.18846	-1239.21637	-1239.43757
9.92	-1237.60572	-1238.75069	-1238.95041	-1238.97628	-1239.17958	-1239.18838	-1239.18838	-1239.21634	-1239.43752

Table S10: The absolute energies in hartree of the potential energy curve of VSi in the $^2\Sigma^+$ state with a SP-based wave function and nom-CPO active space.

Distance	SP	tpbe	ftpbe	trevpbe	ftrevpbe	tblyp	ftblyp	torelyp	ftorelyp
10.02	-1237.60566	-1238.75057	-1238.95029	-1238.97620	-1239.17949	-1239.18831	-1239.18831	-1239.21631	-1239.43748
10.11	-1237.60560	-1238.75046	-1238.95018	-1238.97612	-1239.17940	-1239.18825	-1239.18825	-1239.21629	-1239.43744
10.20	-1237.60556	-1238.75037	-1238.95008	-1238.97606	-1239.17933	-1239.18820	-1239.18820	-1239.21628	-1239.43742
10.30	-1237.60551	-1238.75028	-1238.94999	-1238.97600	-1239.17927	-1239.18815	-1239.18815	-1239.21627	-1239.43740
10.39	-1237.60547	-1238.75021	-1238.94991	-1238.97596	-1239.17922	-1239.18812	-1239.18812	-1239.21627	-1239.43739
10.49	-1237.60543	-1238.75014	-1238.94984	-1238.97591	-1239.17917	-1239.18809	-1239.18809	-1239.21626	-1239.43737
10.58	-1237.60539	-1238.75008	-1238.94977	-1238.97588	-1239.17912	-1239.18806	-1239.18806	-1239.21626	-1239.43737
10.68	-1237.60536	-1238.75002	-1238.94972	-1238.97585	-1239.17909	-1239.18803	-1239.18803	-1239.21627	-1239.43736
10.77	-1237.60533	-1238.74997	-1238.94966	-1238.97582	-1239.17905	-1239.18801	-1239.18801	-1239.21627	-1239.43736
10.87	-1237.60530	-1238.74992	-1238.94961	-1238.97579	-1239.17902	-1239.18799	-1239.18799	-1239.21628	-1239.43735
10.96	-1237.60528	-1238.74988	-1238.94957	-1238.97577	-1239.17899	-1239.18798	-1239.18798	-1239.21628	-1239.43735
11.05	-1237.60525	-1238.74984	-1238.94952	-1238.97575	-1239.17897	-1239.18796	-1239.18796	-1239.21628	-1239.43734
11.15	-1237.60523	-1238.74980	-1238.94948	-1238.97573	-1239.17894	-1239.18795	-1239.18795	-1239.21629	-1239.43734
11.24	-1237.60521	-1238.74977	-1238.94944	-1238.97571	-1239.17892	-1239.18795	-1239.18795	-1239.21629	-1239.43733
11.34	-1237.60519	-1238.74974	-1238.94941	-1238.97569	-1239.17890	-1239.18794	-1239.18794	-1239.21630	-1239.43733
11.43	-1237.60517	-1238.74971	-1238.94938	-1238.97568	-1239.17888	-1239.18794	-1239.18794	-1239.21630	-1239.43733
11.53	-1237.60516	-1238.74969	-1238.94935	-1238.97567	-1239.17886	-1239.18794	-1239.18794	-1239.21631	-1239.43733
11.62	-1237.60514	-1238.74967	-1238.94933	-1238.97566	-1239.17885	-1239.18794	-1239.18794	-1239.21632	-1239.43733
11.72	-1237.60513	-1238.74965	-1238.94931	-1238.97566	-1239.17884	-1239.18794	-1239.18794	-1239.21633	-1239.43733
11.81	-1237.60512	-1238.74963	-1238.94929	-1238.97565	-1239.17883	-1239.18794	-1239.18794	-1239.21634	-1239.43734
11.91	-1237.60510	-1238.74962	-1238.94927	-1238.97565	-1239.17882	-1239.18794	-1239.18794	-1239.21634	-1239.43734
12.00	-1237.60509	-1238.74960	-1238.94926	-1238.97564	-1239.17881	-1239.18794	-1239.18794	-1239.21635	-1239.43734
12.09	-1237.60508	-1238.74959	-1238.94924	-1238.97564	-1239.17881	-1239.18795	-1239.18795	-1239.21635	-1239.43734
12.19	-1237.60507	-1238.74957	-1238.94923	-1238.97563	-1239.17880	-1239.18795	-1239.18795	-1239.21635	-1239.43734
12.28	-1237.60506	-1238.74956	-1238.94921	-1238.97563	-1239.17879	-1239.18795	-1239.18795	-1239.21636	-1239.43734
12.38	-1237.60505	-1238.74955	-1238.94920	-1238.97562	-1239.17878	-1239.18796	-1239.18796	-1239.21636	-1239.43733
12.47	-1237.60504	-1238.74954	-1238.94919	-1238.97561	-1239.17877	-1239.18796	-1239.18796	-1239.21636	-1239.43733
12.57	-1237.60504	-1238.74952	-1238.94917	-1238.97561	-1239.17877	-1239.18796	-1239.18796	-1239.21636	-1239.43733
12.66	-1237.60503	-1238.74951	-1238.94916	-1238.97560	-1239.17875	-1239.18797	-1239.18797	-1239.21636	-1239.43732
12.76	-1237.60502	-1238.74950	-1238.94915	-1238.97560	-1239.17875	-1239.18797	-1239.18797	-1239.21636	-1239.43732
12.85	-1237.60502	-1238.74949	-1238.94914	-1238.97559	-1239.17874	-1239.18797	-1239.18797	-1239.21637	-1239.43732
12.94	-1237.60501	-1238.74949	-1238.94913	-1238.97559	-1239.17874	-1239.18798	-1239.18798	-1239.21637	-1239.43733
13.04	-1237.60500	-1238.74948	-1238.94912	-1238.97559	-1239.17874	-1239.18798	-1239.18798	-1239.21637	-1239.43732
13.13	-1237.60500	-1238.74947	-1238.94912	-1238.97559	-1239.17873	-1239.18799	-1239.18799	-1239.21637	-1239.43732
13.23	-1237.60499	-1238.74947	-1238.94911	-1238.97559	-1239.17873	-1239.18799	-1239.18799	-1239.21638	-1239.43732
13.32	-1237.60499	-1238.74947	-1238.94911	-1238.97558	-1239.17873	-1239.18800	-1239.18800	-1239.21638	-1239.43732
13.42	-1237.60498	-1238.74946	-1238.94910	-1238.97558	-1239.17872	-1239.18800	-1239.18800	-1239.21638	-1239.43732
13.51	-1237.60498	-1238.74946	-1238.94910	-1238.97558	-1239.17872	-1239.18801	-1239.18801	-1239.21638	-1239.43732
13.61	-1237.60497	-1238.74945	-1238.94909	-1238.97558	-1239.17872	-1239.18801	-1239.18801	-1239.21638	-1239.43732
13.70	-1237.60497	-1238.74945	-1238.94909	-1238.97558	-1239.17872	-1239.18802	-1239.18802	-1239.21638	-1239.43732
13.80	-1237.60497	-1238.74944	-1238.94909	-1238.97557	-1239.17871	-1239.18802	-1239.18802	-1239.21638	-1239.43732
13.89	-1237.60496	-1238.74944	-1238.94908	-1238.97557	-1239.17871	-1239.18802	-1239.18802	-1239.21638	-1239.43731
13.98	-1237.60496	-1238.74944	-1238.94908	-1238.97557	-1239.17871	-1239.18803	-1239.18803	-1239.21638	-1239.43731
14.08	-1237.60495	-1238.74943	-1238.94907	-1238.97557	-1239.17870	-1239.18803	-1239.18803	-1239.21638	-1239.43731
14.17	-1237.60495	-1238.74943	-1238.94907	-1238.97557	-1239.17870	-1239.18803	-1239.18803	-1239.21638	-1239.43731
14.27	-1237.60495	-1238.74942	-1238.94906	-1238.97556	-1239.17870	-1239.18804	-1239.18804	-1239.21638	-1239.43731
14.36	-1237.60495	-1238.74942	-1238.94906	-1238.97556	-1239.17870	-1239.18804	-1239.18804	-1239.21638	-1239.43731
14.46	-1237.60494	-1238.74942	-1238.94906	-1238.97556	-1239.17869	-1239.18804	-1239.18804	-1239.21638	-1239.43731
14.55	-1237.60494	-1238.74942	-1238.94906	-1238.97556	-1239.17869	-1239.18805	-1239.18805	-1239.21638	-1239.43731
14.65	-1237.60494	-1238.74941	-1238.94905	-1238.97556	-1239.17869	-1239.18805	-1239.18805	-1239.21638	-1239.43731
14.74	-1237.60493	-1238.74941	-1238.94905	-1238.97556	-1239.17869	-1239.18805	-1239.18805	-1239.21638	-1239.43731
14.83	-1237.60493	-1238.74941	-1238.94905	-1238.97556	-1239.17869	-1239.18806	-1239.18806	-1239.21638	-1239.43731

Table S10: The absolute energies in hartree of the potential energy curve of VSi in the $^2\Sigma^+$ state with a SP-based wave function and nom-CPO active space.

Distance	SP	tpbe	ftpbe	trevpbe	ftrevpbe	tblyp	ftblyp	torelyp	ftorelyp
14.93	-1237.60493	-1238.74941	-1238.94905	-1238.97556	-1239.17869	-1239.18806	-1239.18806	-1239.21638	-1239.43731
15.02	-1237.60493	-1238.74941	-1238.94905	-1238.97555	-1239.17869	-1239.18806	-1239.18806	-1239.21638	-1239.43730
15.12	-1237.60492	-1238.74941	-1238.94905	-1238.97555	-1239.17869	-1239.18807	-1239.18807	-1239.21638	-1239.43731
15.21	-1237.60492	-1238.74941	-1238.94904	-1238.97555	-1239.17868	-1239.18807	-1239.18807	-1239.21638	-1239.43730
15.31	-1237.60492	-1238.74941	-1238.94905	-1238.97555	-1239.17869	-1239.18807	-1239.18807	-1239.21638	-1239.43731
15.40	-1237.60492	-1238.74940	-1238.94904	-1238.97555	-1239.17868	-1239.18807	-1239.18807	-1239.21638	-1239.43730
15.50	-1237.60492	-1238.74940	-1238.94904	-1238.97555	-1239.17868	-1239.18807	-1239.18807	-1239.21638	-1239.43731
15.59	-1237.60491	-1238.74940	-1238.94904	-1238.97555	-1239.17868	-1239.18808	-1239.18808	-1239.21638	-1239.43730
15.68	-1237.60491	-1238.74940	-1238.94904	-1238.97555	-1239.17868	-1239.18808	-1239.18808	-1239.21638	-1239.43731
15.78	-1237.60491	-1238.74940	-1238.94904	-1238.97554	-1239.17868	-1239.18808	-1239.18808	-1239.21638	-1239.43730
15.87	-1237.60491	-1238.74940	-1238.94904	-1238.97554	-1239.17868	-1239.18808	-1239.18808	-1239.21638	-1239.43731
15.97	-1237.60491	-1238.74939	-1238.94903	-1238.97554	-1239.17868	-1239.18808	-1239.18808	-1239.21637	-1239.43730
16.06	-1237.60491	-1238.74939	-1238.94904	-1238.97554	-1239.17868	-1239.18808	-1239.18808	-1239.21638	-1239.43731
16.16	-1237.60490	-1238.74939	-1238.94903	-1238.97554	-1239.17867	-1239.18808	-1239.18808	-1239.21637	-1239.43730
16.25	-1237.60490	-1238.74939	-1238.94904	-1238.97554	-1239.17868	-1239.18809	-1239.18809	-1239.21638	-1239.43731
16.35	-1237.60490	-1238.74939	-1238.94903	-1238.97554	-1239.17867	-1239.18809	-1239.18809	-1239.21638	-1239.43730
16.44	-1237.60490	-1238.74939	-1238.94903	-1238.97554	-1239.17867	-1239.18809	-1239.18809	-1239.21637	-1239.43730
16.54	-1237.60490	-1238.74939	-1238.94903	-1238.97554	-1239.17867	-1239.18809	-1239.18809	-1239.21637	-1239.43730
16.63	-1237.60490	-1238.74939	-1238.94903	-1238.97554	-1239.17867	-1239.18809	-1239.18809	-1239.21638	-1239.43730
16.72	-1237.60490	-1238.74939	-1238.94903	-1238.97554	-1239.17867	-1239.18809	-1239.18809	-1239.21637	-1239.43730
16.82	-1237.60490	-1238.74939	-1238.94903	-1238.97554	-1239.17867	-1239.18809	-1239.18809	-1239.21638	-1239.43731
16.91	-1237.60489	-1238.74939	-1238.94903	-1238.97554	-1239.17867	-1239.18810	-1239.18810	-1239.21637	-1239.43730
17.01	-1237.60489	-1238.74939	-1238.94903	-1238.97554	-1239.17867	-1239.18810	-1239.18810	-1239.21638	-1239.43731
17.10	-1237.60489	-1238.74939	-1238.94903	-1238.97554	-1239.17867	-1239.18810	-1239.18810	-1239.21638	-1239.43730
17.20	-1237.60489	-1238.74939	-1238.94903	-1238.97554	-1239.17867	-1239.18810	-1239.18810	-1239.21638	-1239.43731
17.29	-1237.60489	-1238.74939	-1238.94903	-1238.97554	-1239.17867	-1239.18810	-1239.18810	-1239.21637	-1239.43730
17.39	-1237.60489	-1238.74939	-1238.94903	-1238.97554	-1239.17867	-1239.18810	-1239.18810	-1239.21638	-1239.43731
17.48	-1237.60489	-1238.74939	-1238.94903	-1238.97553	-1239.17867	-1239.18810	-1239.18810	-1239.21637	-1239.43730
17.57	-1237.60489	-1238.74938	-1238.94903	-1238.97553	-1239.17867	-1239.18810	-1239.18810	-1239.21637	-1239.43730
17.67	-1237.60489	-1238.74939	-1238.94903	-1238.97554	-1239.17867	-1239.18810	-1239.18810	-1239.21637	-1239.43731
17.76	-1237.60489	-1238.74938	-1238.94903	-1238.97553	-1239.17867	-1239.18810	-1239.18810	-1239.21637	-1239.43730
17.86	-1237.60488	-1238.74938	-1238.94903	-1238.97553	-1239.17867	-1239.18810	-1239.18810	-1239.21637	-1239.43730
17.95	-1237.60488	-1238.74938	-1238.94903	-1238.97553	-1239.17867	-1239.18810	-1239.18810	-1239.21637	-1239.43730
18.05	-1237.60488	-1238.74938	-1238.94902	-1238.97553	-1239.17867	-1239.18810	-1239.18810	-1239.21637	-1239.43730

Table S11: The absolute energies in hartree of the potential energy curve of VSi in the $^2\Delta$ state with a SP-based wave function and nom-CPO active space.

Distance	SP	tpbe	ftpbe	trevpbe	ftrevpbe	tblyp	ftblyp	torelyp	ftorelyp
1.98	-1235.24350	-1236.52863	-1236.73815	-1236.73590	-1236.94637	-1236.94108	-1237.14876	-1237.00355	-1237.23554
2.08	-1235.66382	-1236.93763	-1237.14752	-1237.14616	-1237.35707	-1237.35136	-1237.55939	-1237.41128	-1237.64213
2.17	-1235.99217	-1237.25827	-1237.46751	-1237.46792	-1237.67822	-1237.67328	-1237.88062	-1237.72906	-1237.95910
2.27	-1236.25011	-1237.50938	-1237.71827	-1237.72009	-1237.93008	-1237.92586	-1238.13283	-1237.97768	-1238.20727
2.36	-1236.47798	-1237.69846	-1237.90823	-1237.91016	-1238.12169	-1238.12081	-1238.32943	-1238.15888	-1238.39290
2.46	-1236.64869	-1237.86858	-1238.07865	-1238.08114	-1238.29290	-1238.29212	-1238.50107	-1238.32745	-1238.56172
2.55	-1236.78809	-1238.00946	-1238.21983	-1238.22283	-1238.43478	-1238.43414	-1238.64354	-1238.46743	-1238.70187
2.65	-1236.90417	-1238.12762	-1238.33858	-1238.34178	-1238.55430	-1238.55322	-1238.76333	-1238.58520	-1238.82007
2.74	-1237.00245	-1238.22763	-1238.43894	-1238.44252	-1238.65541	-1238.65394	-1238.86443	-1238.68501	-1238.92014
2.83	-1237.09016	-1238.31365	-1238.52561	-1238.52923	-1238.74300	-1238.73961	-1238.95068	-1238.77144	-1239.00761
2.93	-1237.16925	-1238.38888	-1238.60135	-1238.60503	-1238.81963	-1238.81410	-1239.02558	-1238.84600	-1239.08311
3.02	-1237.23841	-1238.45373	-1238.66663	-1238.67045	-1238.88571	-1238.87844	-1239.09021	-1238.91014	-1239.14790

Table S11: The absolute energies in hartree of the potential energy curve of VSi in the $^2\Delta$ state with a SP-based wave function and nom-CPO active space.

Distance	SP	tpbe	ftpbe	trevpbe	ftrevpbe	tblyp	ftblyp	torelyp	ftorelyp
3.12	-1237.29802	-1238.50944	-1238.72257	-1238.72670	-1238.94234	-1238.93400	-1239.14590	-1238.96529	-1239.20338
3.21	-1237.34900	-1238.55702	-1238.77016	-1238.77479	-1238.99053	-1238.98158	-1239.19342	-1239.01239	-1239.25053
3.31	-1237.39234	-1238.59738	-1238.81036	-1238.81563	-1239.03123	-1239.02204	-1239.23360	-1239.05234	-1239.29024
3.40	-1237.42900	-1238.63138	-1238.84423	-1238.85010	-1239.06560	-1239.05620	-1239.26753	-1239.08597	-1239.32373
3.50	-1237.45984	-1238.65981	-1238.87251	-1238.87896	-1239.09435	-1239.08482	-1239.29593	-1239.11406	-1239.35168
3.59	-1237.48560	-1238.68338	-1238.89583	-1238.90294	-1239.11808	-1239.10861	-1239.31937	-1239.13732	-1239.37463
3.68	-1237.50698	-1238.70273	-1238.91491	-1238.92268	-1239.13755	-1239.12820	-1239.33858	-1239.15638	-1239.39335
3.78	-1237.52459	-1238.71841	-1238.93038	-1238.93872	-1239.15340	-1239.14412	-1239.35420	-1239.17180	-1239.40853
3.87	-1237.53895	-1238.73092	-1238.94267	-1238.95159	-1239.16607	-1239.15688	-1239.36664	-1239.18408	-1239.42056
3.97	-1237.55055	-1238.74071	-1238.95218	-1238.96171	-1239.17591	-1239.16692	-1239.37629	-1239.19364	-1239.42977
4.06	-1237.55981	-1238.74814	-1238.95933	-1238.96945	-1239.18337	-1239.17460	-1239.38356	-1239.20087	-1239.43662
4.16	-1237.56709	-1238.75355	-1238.96451	-1238.97517	-1239.18886	-1239.18026	-1239.38886	-1239.20610	-1239.44155
4.35	-1237.57270	-1238.75723	-1238.96798	-1238.97914	-1239.19265	-1239.18417	-1239.39245	-1239.20962	-1239.44481
4.44	-1237.57691	-1238.75942	-1238.96998	-1238.98161	-1239.19495	-1239.18660	-1239.39455	-1239.21166	-1239.44661
4.54	-1237.57996	-1238.76037	-1238.97071	-1238.98284	-1239.19598	-1239.18778	-1239.39538	-1239.21249	-1239.44717
4.63	-1237.58204	-1238.76027	-1238.97040	-1238.98301	-1239.19596	-1239.18790	-1239.39514	-1239.21230	-1239.44670
4.72	-1237.58331	-1238.75931	-1238.96925	-1238.98230	-1239.19510	-1239.18714	-1239.39406	-1239.21126	-1239.44544
4.82	-1237.58392	-1238.75761	-1238.96739	-1238.98086	-1239.19353	-1239.18566	-1239.39229	-1239.20952	-1239.44351
4.91	-1237.58398	-1238.75530	-1238.96493	-1238.97879	-1239.19134	-1239.18356	-1239.38990	-1239.20720	-1239.44101
5.01	-1237.58360	-1238.75226	-1238.96173	-1238.97599	-1239.18840	-1239.18073	-1239.38677	-1239.20416	-1239.43779
5.10	-1237.58294	-1238.74619	-1238.95542	-1238.97019	-1239.18239	-1239.17491	-1239.38047	-1239.19809	-1239.43143
5.20	-1237.58209	-1238.74200	-1238.95105	-1238.96621	-1239.17824	-1239.17094	-1239.37620	-1239.19397	-1239.42711
5.29	-1237.58098	-1238.73871	-1238.94763	-1238.96311	-1239.17502	-1239.16789	-1239.37295	-1239.19080	-1239.42381
5.39	-1237.57963	-1238.73568	-1238.94450	-1238.96025	-1239.17207	-1239.16511	-1239.37000	-1239.18790	-1239.42082
5.48	-1237.57807	-1238.73277	-1238.94152	-1238.95749	-1239.16924	-1239.16245	-1239.36722	-1239.18513	-1239.41799
5.57	-1237.57635	-1238.72995	-1238.93862	-1238.95481	-1239.16649	-1239.15989	-1239.36455	-1239.18245	-1239.41526
5.67	-1237.57450	-1238.72718	-1238.93579	-1238.95218	-1239.16379	-1239.15740	-1239.36196	-1239.17983	-1239.41260
5.76	-1237.57254	-1238.72448	-1238.93303	-1238.94960	-1239.16114	-1239.15499	-1239.35945	-1239.17728	-1239.41001
5.86	-1237.57051	-1238.72183	-1238.93034	-1238.94707	-1239.15856	-1239.15264	-1239.35703	-1239.17479	-1239.40750
5.95	-1237.56844	-1238.71927	-1238.92773	-1238.94460	-1239.15605	-1239.15038	-1239.35472	-1239.17238	-1239.40509
6.05	-1237.56634	-1238.71678	-1238.92521	-1238.94221	-1239.15362	-1239.14822	-1239.35251	-1239.17006	-1239.40277
6.14	-1237.56425	-1238.71438	-1238.92278	-1238.93990	-1239.15126	-1239.14615	-1239.35040	-1239.16781	-1239.40054
6.24	-1237.56217	-1238.71207	-1238.92045	-1238.93767	-1239.14900	-1239.14419	-1239.34842	-1239.16566	-1239.39841
6.33	-1237.56014	-1238.70986	-1238.91822	-1238.93552	-1239.14682	-1239.14233	-1239.34655	-1239.16359	-1239.39638
6.43	-1237.55815	-1238.70773	-1238.91607	-1238.93346	-1239.14471	-1239.14059	-1239.34479	-1239.16162	-1239.39443
6.52	-1237.55624	-1238.70569	-1238.91401	-1238.93148	-1239.14270	-1239.13894	-1239.34314	-1239.15973	-1239.39259
6.61	-1237.55441	-1238.70373	-1238.91205	-1238.92957	-1239.14077	-1239.13739	-1239.34162	-1239.15792	-1239.39084
6.71	-1237.55268	-1238.70185	-1238.91017	-1238.92773	-1239.13891	-1239.13594	-1239.34020	-1239.15620	-1239.38917
6.80	-1237.55106	-1238.70004	-1238.90835	-1238.92595	-1239.13710	-1239.13458	-1239.33888	-1239.15454	-1239.38755
6.90	-1237.54957	-1238.69828	-1238.90658	-1238.92423	-1239.13535	-1239.13330	-1239.33764	-1239.15294	-1239.38599
6.99	-1237.54821	-1238.69658	-1238.90488	-1238.92256	-1239.13364	-1239.13208	-1239.33648	-1239.15141	-1239.38449
7.09	-1237.54699	-1238.69491	-1238.90322	-1238.92093	-1239.13199	-1239.13091	-1239.33538	-1239.14993	-1239.38303
7.18	-1237.54591	-1238.69330	-1238.90162	-1238.91935	-1239.13039	-1239.12980	-1239.33434	-1239.14852	-1239.38163
7.28	-1237.54496	-1238.69177	-1238.90011	-1238.91785	-1239.12889	-1239.12874	-1239.33336	-1239.14720	-1239.38033
7.37	-1237.54415	-1238.69034	-1238.89870	-1238.91647	-1239.12751	-1239.12775	-1239.33245	-1239.14600	-1239.37914
7.46	-1237.54344	-1238.68903	-1238.89742	-1238.91520	-1239.12626	-1239.12684	-1239.33161	-1239.14492	-1239.37808
7.56	-1237.54283	-1238.68784	-1238.89626	-1238.91407	-1239.12515	-1239.12601	-1239.33084	-1239.14396	-1239.37713
7.65	-1237.54230	-1238.68678	-1238.89523	-1238.91307	-1239.12415	-1239.12527	-1239.33015	-1239.14312	-1239.37629
7.75	-1237.54183	-1238.68583	-1238.89430	-1238.91218	-1239.12328	-1239.12460	-1239.32952	-1239.14238	-1239.37557
7.84	-1237.54142	-1238.68499	-1238.89346	-1238.91139	-1239.12249	-1239.12400	-1239.32896	-1239.14174	-1239.37492
7.94	-1237.54106	-1238.68422	-1238.89270	-1238.91068	-1239.12179	-1239.12346	-1239.32844	-1239.14117	-1239.37436
8.03	-1237.54073	-1238.68353	-1238.89202	-1238.91005	-1239.12117	-1239.12298	-1239.32798	-1239.14067	-1239.37386

Table S11: The absolute energies in hartree of the potential energy curve of VSi in the $^2\Delta$ state with a SP-based wave function and nom-CPO active space.

Distance	SP	tpbe	ftpbe	trevpbe	ftrevpbe	tblyp	ftblyp	torelyp	ftorelyp
8.13	-1237.54044	-1238.68291	-1238.89141	-1238.90949	-1239.12061	-1239.12254	-1239.32756	-1239.14024	-1239.37343
8.22	-1237.54018	-1238.68235	-1238.89085	-1238.90898	-1239.12011	-1239.12215	-1239.32718	-1239.13986	-1239.37304
8.31	-1237.53995	-1238.68185	-1238.89034	-1238.90853	-1239.11966	-1239.12180	-1239.32683	-1239.13953	-1239.37271
8.41	-1237.53973	-1238.68139	-1238.88988	-1238.90813	-1239.11926	-1239.12149	-1239.32652	-1239.13925	-1239.37242
8.50	-1237.53954	-1238.68098	-1238.88947	-1238.90778	-1239.11889	-1239.12121	-1239.32624	-1239.13901	-1239.37216
8.60	-1237.53937	-1238.68060	-1238.88909	-1238.90746	-1239.11857	-1239.12096	-1239.32598	-1239.13879	-1239.37194
8.69	-1237.53921	-1238.68026	-1238.88875	-1238.90717	-1239.11828	-1239.12073	-1239.32575	-1239.13861	-1239.37174
8.79	-1237.53906	-1238.67996	-1238.88843	-1238.90691	-1239.11802	-1239.12053	-1239.32555	-1239.13846	-1239.37158
8.88	-1237.53893	-1238.67968	-1238.88815	-1238.90669	-1239.11778	-1239.12034	-1239.32536	-1239.13833	-1239.37143
8.98	-1237.53881	-1238.67942	-1238.88789	-1238.90648	-1239.11757	-1239.12018	-1239.32518	-1239.13821	-1239.37130
9.07	-1237.53870	-1238.67919	-1238.88765	-1238.90630	-1239.11738	-1239.12003	-1239.32503	-1239.13812	-1239.37119
9.17	-1237.53860	-1238.67899	-1238.88744	-1238.90614	-1239.11721	-1239.11990	-1239.32489	-1239.13805	-1239.37110
9.26	-1237.53851	-1238.67880	-1238.88724	-1238.90600	-1239.11706	-1239.11979	-1239.32477	-1239.13799	-1239.37103
9.35	-1237.53843	-1238.67864	-1238.88707	-1238.90588	-1239.11693	-1239.11969	-1239.32466	-1239.13794	-1239.37097
9.45	-1237.53835	-1238.67849	-1238.88691	-1238.90577	-1239.11681	-1239.11961	-1239.32457	-1239.13791	-1239.37092
9.54	-1237.53828	-1238.67835	-1238.88677	-1238.90567	-1239.11671	-1239.11953	-1239.32448	-1239.13788	-1239.37088
9.64	-1237.53821	-1238.67823	-1238.88664	-1238.90559	-1239.11661	-1239.11946	-1239.32440	-1239.13786	-1239.37085
9.73	-1237.53815	-1238.67812	-1238.88652	-1238.90552	-1239.11653	-1239.11940	-1239.32433	-1239.13785	-1239.37082
9.83	-1237.53810	-1238.67802	-1238.88642	-1238.90545	-1239.11646	-1239.11935	-1239.32427	-1239.13784	-1239.37080
9.92	-1237.53804	-1238.67793	-1238.88632	-1238.90540	-1239.11639	-1239.11931	-1239.32422	-1239.13784	-1239.37078
10.02	-1237.53800	-1238.67785	-1238.88623	-1238.90535	-1239.11633	-1239.11927	-1239.32417	-1239.13784	-1239.37076
10.11	-1237.53795	-1238.67777	-1238.88614	-1238.90530	-1239.11628	-1239.11924	-1239.32413	-1239.13785	-1239.37075
10.20	-1237.53791	-1238.67770	-1238.88607	-1238.90526	-1239.11623	-1239.11921	-1239.32409	-1239.13785	-1239.37074
10.30	-1237.53788	-1238.67764	-1238.88600	-1238.90523	-1239.11619	-1239.11919	-1239.32406	-1239.13786	-1239.37074
10.39	-1237.53784	-1238.67759	-1238.88594	-1238.90520	-1239.11615	-1239.11917	-1239.32403	-1239.13787	-1239.37074
10.49	-1237.53781	-1238.67754	-1238.88588	-1238.90518	-1239.11612	-1239.11916	-1239.32401	-1239.13789	-1239.37074
10.58	-1237.53778	-1238.67750	-1238.88584	-1238.90516	-1239.11609	-1239.11915	-1239.32399	-1239.13790	-1239.37074
10.68	-1237.53775	-1238.67746	-1238.88579	-1238.90515	-1239.11607	-1239.11914	-1239.32398	-1239.13792	-1239.37074
10.77	-1237.53772	-1238.67742	-1238.88575	-1238.90513	-1239.11605	-1239.11913	-1239.32396	-1239.13793	-1239.37075
10.87	-1237.53770	-1238.67739	-1238.88571	-1238.90512	-1239.11603	-1239.11913	-1239.32395	-1239.13794	-1239.37075
10.96	-1237.53768	-1238.67736	-1238.88568	-1238.90511	-1239.11601	-1239.11912	-1239.32394	-1239.13795	-1239.37075
11.05	-1237.53766	-1238.67733	-1238.88565	-1238.90510	-1239.11600	-1239.11912	-1239.32393	-1239.13797	-1239.37075
11.15	-1237.53764	-1238.67731	-1238.88562	-1238.90508	-1239.11598	-1239.11912	-1239.32393	-1239.13798	-1239.37075
11.24	-1237.53762	-1238.67728	-1238.88559	-1238.90508	-1239.11596	-1239.11912	-1239.32392	-1239.13799	-1239.37075
11.34	-1237.53760	-1238.67726	-1238.88556	-1238.90507	-1239.11595	-1239.11913	-1239.32392	-1239.13800	-1239.37075
11.43	-1237.53759	-1238.67724	-1238.88554	-1238.90506	-1239.11594	-1239.11913	-1239.32392	-1239.13801	-1239.37075
11.53	-1237.53757	-1238.67723	-1238.88552	-1238.90506	-1239.11593	-1239.11914	-1239.32392	-1239.13802	-1239.37075
11.62	-1237.53756	-1238.67721	-1238.88550	-1238.90506	-1239.11592	-1239.11915	-1239.32392	-1239.13804	-1239.37076
11.72	-1237.53754	-1238.67720	-1238.88548	-1238.90506	-1239.11592	-1239.11915	-1239.32393	-1239.13805	-1239.37076
11.81	-1237.53753	-1238.67719	-1238.88547	-1238.90505	-1239.11591	-1239.11916	-1239.32393	-1239.13805	-1239.37076
11.91	-1237.53752	-1238.67718	-1238.88546	-1238.90505	-1239.11590	-1239.11917	-1239.32393	-1239.13806	-1239.37076
12.00	-1237.53751	-1238.67717	-1238.88544	-1238.90505	-1239.11590	-1239.11917	-1239.32393	-1239.13807	-1239.37076
12.09	-1237.53750	-1238.67716	-1238.88543	-1238.90505	-1239.11589	-1239.11918	-1239.32394	-1239.13807	-1239.37076
12.19	-1237.53749	-1238.67715	-1238.88542	-1238.90504	-1239.11589	-1239.11919	-1239.32394	-1239.13807	-1239.37076
12.28	-1237.53748	-1238.67714	-1238.88541	-1238.90504	-1239.11588	-1239.11919	-1239.32394	-1239.13808	-1239.37076
12.38	-1237.53747	-1238.67713	-1238.88540	-1238.90504	-1239.11588	-1239.11920	-1239.32395	-1239.13808	-1239.37076
12.47	-1237.53746	-1238.67712	-1238.88539	-1238.90504	-1239.11587	-1239.11921	-1239.32395	-1239.13809	-1239.37076
12.57	-1237.53746	-1238.67711	-1238.88538	-1238.90504	-1239.11587	-1239.11921	-1239.32396	-1239.13809	-1239.37075
12.66	-1237.53745	-1238.67711	-1238.88537	-1238.90503	-1239.11586	-1239.11922	-1239.32396	-1239.13810	-1239.37075
12.76	-1237.53744	-1238.67710	-1238.88536	-1238.90503	-1239.11586	-1239.11923	-1239.32397	-1239.13810	-1239.37075
12.85	-1237.53743	-1238.67710	-1238.88536	-1238.90503	-1239.11585	-1239.11924	-1239.32397	-1239.13810	-1239.37075
12.94	-1237.53743	-1238.67709	-1238.88535	-1238.90503	-1239.11585	-1239.11924	-1239.32398	-1239.13811	-1239.37075

Table S11: The absolute energies in hartree of the potential energy curve of VSi in the $^2\Delta$ state with a SP-based wave function and nom-CPO active space.

Distance	SP	tpbe	ftpbe	trevpbe	ftrevpbe	tblyp	ftblyp	torelyp	ftorelyp
13.04	-1237.53742	-1238.67709	-1238.88535	-1238.90503	-1239.11585	-1239.11925	-1239.32398	-1239.13811	-1239.37075
13.13	-1237.53742	-1238.67709	-1238.88535	-1238.90503	-1239.11585	-1239.11926	-1239.32399	-1239.13811	-1239.37075
13.23	-1237.53741	-1238.67709	-1238.88534	-1238.90503	-1239.11585	-1239.11927	-1239.32400	-1239.13812	-1239.37075
13.32	-1237.53741	-1238.67708	-1238.88534	-1238.90503	-1239.11584	-1239.11927	-1239.32400	-1239.13811	-1239.37074
13.42	-1237.53740	-1238.67708	-1238.88533	-1238.90503	-1239.11584	-1239.11928	-1239.32400	-1239.13811	-1239.37074
13.51	-1237.53740	-1238.67708	-1238.88533	-1238.90503	-1239.11584	-1239.11928	-1239.32401	-1239.13811	-1239.37074
13.61	-1237.53739	-1238.67707	-1238.88533	-1238.90503	-1239.11584	-1239.11929	-1239.32401	-1239.13811	-1239.37074
13.70	-1237.53739	-1238.67707	-1238.88532	-1238.90503	-1239.11583	-1239.11929	-1239.32402	-1239.13811	-1239.37074
13.80	-1237.53738	-1238.67707	-1238.88532	-1238.90503	-1239.11583	-1239.11930	-1239.32402	-1239.13811	-1239.37074
13.89	-1237.53738	-1238.67706	-1238.88532	-1238.90503	-1239.11583	-1239.11930	-1239.32402	-1239.13812	-1239.37074
13.98	-1237.53738	-1238.67706	-1238.88531	-1238.90502	-1239.11583	-1239.11931	-1239.32403	-1239.13812	-1239.37074
14.08	-1237.53737	-1238.67706	-1238.88531	-1238.90502	-1239.11583	-1239.11931	-1239.32403	-1239.13812	-1239.37074
14.17	-1237.53737	-1238.67706	-1238.88531	-1238.90502	-1239.11583	-1239.11931	-1239.32404	-1239.13812	-1239.37073
14.27	-1237.53737	-1238.67706	-1238.88531	-1238.90502	-1239.11582	-1239.11932	-1239.32404	-1239.13812	-1239.37073
14.36	-1237.53736	-1238.67706	-1238.88531	-1238.90502	-1239.11582	-1239.11932	-1239.32404	-1239.13812	-1239.37073
14.46	-1237.53736	-1238.67706	-1238.88531	-1238.90502	-1239.11582	-1239.11933	-1239.32405	-1239.13812	-1239.37073
14.55	-1237.53736	-1238.67706	-1238.88530	-1238.90502	-1239.11582	-1239.11933	-1239.32405	-1239.13812	-1239.37073
14.65	-1237.53736	-1238.67706	-1238.88530	-1238.90502	-1239.11582	-1239.11934	-1239.32405	-1239.13812	-1239.37073
14.74	-1237.53735	-1238.67706	-1238.88530	-1238.90502	-1239.11582	-1239.11934	-1239.32406	-1239.13812	-1239.37073
14.83	-1237.53735	-1238.67706	-1238.88530	-1238.90503	-1239.11582	-1239.11935	-1239.32406	-1239.13813	-1239.37073
14.93	-1237.53735	-1238.67706	-1238.88530	-1238.90503	-1239.11582	-1239.11935	-1239.32407	-1239.13813	-1239.37073
15.02	-1237.53735	-1238.67706	-1238.88530	-1238.90503	-1239.11582	-1239.11935	-1239.32407	-1239.13813	-1239.37073
15.12	-1237.53734	-1238.67706	-1238.88530	-1238.90503	-1239.11582	-1239.11936	-1239.32407	-1239.13813	-1239.37073
15.21	-1237.53734	-1238.67706	-1238.88530	-1238.90503	-1239.11582	-1239.11936	-1239.32407	-1239.13813	-1239.37073
15.31	-1237.53734	-1238.67705	-1238.88530	-1238.90503	-1239.11582	-1239.11936	-1239.32408	-1239.13813	-1239.37073
15.40	-1237.53734	-1238.67705	-1238.88530	-1238.90502	-1239.11582	-1239.11936	-1239.32408	-1239.13813	-1239.37073
15.50	-1237.53734	-1238.67705	-1238.88530	-1238.90502	-1239.11582	-1239.11937	-1239.32408	-1239.13813	-1239.37073
15.59	-1237.53734	-1238.67705	-1238.88530	-1238.90502	-1239.11582	-1239.11937	-1239.32408	-1239.13813	-1239.37073
15.68	-1237.53733	-1238.67705	-1238.88530	-1238.90502	-1239.11582	-1239.11937	-1239.32408	-1239.13813	-1239.37073
15.78	-1237.53733	-1238.67705	-1238.88530	-1238.90502	-1239.11582	-1239.11937	-1239.32408	-1239.13813	-1239.37073
15.87	-1237.53733	-1238.67705	-1238.88530	-1238.90502	-1239.11582	-1239.11937	-1239.32408	-1239.13813	-1239.37073
15.97	-1237.53733	-1238.67705	-1238.88530	-1238.90502	-1239.11582	-1239.11937	-1239.32409	-1239.13813	-1239.37073
16.06	-1237.53733	-1238.67705	-1238.88530	-1238.90502	-1239.11582	-1239.11937	-1239.32409	-1239.13813	-1239.37073
16.16	-1237.53733	-1238.67705	-1238.88530	-1238.90502	-1239.11582	-1239.11938	-1239.32409	-1239.13813	-1239.37073
16.25	-1237.53733	-1238.67705	-1238.88530	-1238.90502	-1239.11582	-1239.11938	-1239.32409	-1239.13813	-1239.37073
16.35	-1237.53732	-1238.67705	-1238.88530	-1238.90502	-1239.11582	-1239.11938	-1239.32409	-1239.13813	-1239.37073
16.44	-1237.53732	-1238.67705	-1238.88530	-1238.90502	-1239.11582	-1239.11938	-1239.32409	-1239.13813	-1239.37073
16.54	-1237.53732	-1238.67705	-1238.88530	-1238.90502	-1239.11582	-1239.11938	-1239.32410	-1239.13813	-1239.37073
16.63	-1237.53732	-1238.67705	-1238.88530	-1238.90502	-1239.11582	-1239.11939	-1239.32410	-1239.13813	-1239.37073
16.72	-1237.53732	-1238.67705	-1238.88530	-1238.90502	-1239.11582	-1239.11939	-1239.32410	-1239.13813	-1239.37073
16.82	-1237.53732	-1238.67705	-1238.88530	-1238.90503	-1239.11582	-1239.11939	-1239.32410	-1239.13813	-1239.37073
16.91	-1237.53732	-1238.67705	-1238.88530	-1238.90503	-1239.11582	-1239.11939	-1239.32411	-1239.13813	-1239.37073
17.01	-1237.53732	-1238.67706	-1238.88530	-1238.90503	-1239.11582	-1239.11940	-1239.32411	-1239.13813	-1239.37073
17.10	-1237.53732	-1238.67706	-1238.88530	-1238.90503	-1239.11582	-1239.11940	-1239.32411	-1239.13814	-1239.37073
17.20	-1237.53732	-1238.67706	-1238.88530	-1238.90503	-1239.11582	-1239.11940	-1239.32411	-1239.13814	-1239.37073
17.29	-1237.53731	-1238.67706	-1238.88530	-1238.90503	-1239.11582	-1239.11940	-1239.32411	-1239.13814	-1239.37074
17.39	-1237.53731	-1238.67706	-1238.88530	-1238.90503	-1239.11582	-1239.11940	-1239.32411	-1239.13814	-1239.37073
17.48	-1237.53731	-1238.67706	-1238.88530	-1238.90503	-1239.11582	-1239.11940	-1239.32411	-1239.13813	-1239.37073
17.57	-1237.53731	-1238.67706	-1238.88530	-1238.90503	-1239.11582	-1239.11940	-1239.32411	-1239.13813	-1239.37073
17.67	-1237.53731	-1238.67705	-1238.88530	-1238.90503	-1239.11582	-1239.11940	-1239.32411	-1239.13813	-1239.37073
17.76	-1237.53731	-1238.67705	-1238.88530	-1238.90503	-1239.11582	-1239.11940	-1239.32411	-1239.13813	-1239.37073
17.86	-1237.53731	-1238.67705	-1238.88530	-1238.90503	-1239.11582	-1239.11940	-1239.32411	-1239.13813	-1239.37073

Table S11: The absolute energies in hartree of the potential energy curve of VSi in the $^2\Delta$ state with a SP-based wave function and nom-CPO active space.

Distance	SP	tpbe	ftpbe	trevpbe	ftrevpbe	tblyp	ftblyp	torelyp	ftorelyp
17.95	-1237.53731	-1238.67705	-1238.88530	-1238.90503	-1239.11582	-1239.11940	-1239.32411	-1239.13813	-1239.37073
18.05	-1237.53731	-1238.67705	-1238.88530	-1238.90502	-1239.11582	-1239.11940	-1239.32411	-1239.13813	-1239.37073

Table S12: The absolute energies in hartree of the potential energy curve of NbSi in the $^4\Pi$ state with a CASSCF wave function and nom-CPO active space.

Distance	CASSCF	CASPT2	tpbe	ftpbe	trevpbe	ftrevpbe	tblyp	ftblyp	torelyp	ftorelyp
1.98	-4102.00026	-4102.48735	-4104.18979	-4104.66309	-4104.46862	-4104.94503	-4104.82668	-4105.29417	-4105.76608	-4106.28632
2.08	-4102.80637	-4103.29636	-4104.99127	-4105.46440	-4105.27125	-4105.74747	-4105.63012	-4106.09740	-4106.56359	-4107.08362
2.17	-4103.45707	-4103.94981	-4105.63685	-4106.10984	-4105.91804	-4106.39409	-4106.27730	-4106.74440	-4107.20580	-4107.72561
2.27	-4103.97998	-4104.47601	-4106.15515	-4106.62804	-4106.43760	-4106.91351	-4106.79686	-4107.26377	-4107.72150	-4108.24113
2.36	-4104.40138	-4104.90756	-4106.57199	-4107.04427	-4106.85569	-4107.33082	-4107.21512	-4107.68119	-4108.13843	-4108.65703
2.46	-4104.74707	-4105.25372	-4106.90825	-4107.37841	-4107.19290	-4107.66501	-4107.55337	-4108.01807	-4108.47427	-4108.98914
2.55	-4105.02867	-4105.52914	-4107.17813	-4107.64579	-4107.46377	-4107.93261	-4107.82468	-4108.28793	-4108.74337	-4109.25435
2.65	-4105.25654	-4105.75365	-4107.40005	-4107.86759	-4107.68658	-4108.15533	-4108.04726	-4108.51028	-4108.96333	-4109.47423
2.74	-4105.42599	-4105.90016	-4107.55651	-4108.02787	-4107.84255	-4108.31666	-4108.20722	-4108.67374	-4109.10815	-4109.62653
2.83	-4105.58936	-4106.05930	-4107.71501	-4108.18661	-4108.00187	-4108.47627	-4108.36601	-4108.83254	-4109.26552	-4109.78412
2.93	-4105.72479	-4106.19133	-4107.84699	-4108.31863	-4108.13461	-4108.60908	-4108.49840	-4108.96477	-4109.39647	-4109.91505
3.02	-4105.83711	-4106.30154	-4107.95701	-4108.42858	-4108.24531	-4108.71975	-4108.60885	-4109.07498	-4109.50547	-4110.02400
3.12	-4105.93026	-4106.39360	-4108.04885	-4108.52030	-4108.33775	-4108.81212	-4108.70115	-4109.16700	-4109.59629	-4110.11477
3.21	-4106.00748	-4106.47047	-4108.12558	-4108.59684	-4108.41501	-4108.88926	-4108.77834	-4109.24385	-4109.67202	-4110.19043
3.31	-4106.05987	-4106.53073	-4108.18751	-4108.65682	-4108.47758	-4108.94942	-4108.84181	-4109.30616	-4109.73635	-4110.25136
3.40	-4106.11529	-4106.58602	-4108.24172	-4108.71019	-4108.53215	-4109.00338	-4108.89658	-4109.35977	-4109.78926	-4110.30376
3.50	-4106.16210	-4106.63176	-4108.28696	-4108.75498	-4108.57775	-4109.04872	-4108.94219	-4109.40475	-4109.83342	-4110.34765
3.59	-4106.20103	-4106.66959	-4108.32441	-4108.79194	-4108.61552	-4109.08612	-4108.98011	-4109.44207	-4109.86991	-4110.38376
3.68	-4106.23312	-4106.70053	-4108.35502	-4108.82213	-4108.64644	-4109.11670	-4109.01124	-4109.47269	-4109.89967	-4110.41318
3.78	-4106.25931	-4106.72555	-4108.37977	-4108.84654	-4108.67148	-4109.14147	-4109.03652	-4109.49754	-4109.92372	-4110.43694
3.87	-4106.28043	-4106.74553	-4108.39957	-4108.86607	-4108.69155	-4109.16133	-4109.05685	-4109.51751	-4109.94292	-4110.45591
3.97	-4106.29722	-4106.76124	-4108.41513	-4108.88141	-4108.70738	-4109.17698	-4109.07296	-4109.53330	-4109.95798	-4110.47077
4.06	-4106.31031	-4106.77333	-4108.42711	-4108.89319	-4108.71961	-4109.18906	-4109.08548	-4109.54553	-4109.96954	-4110.48216
4.16	-4106.32026	-4106.78238	-4108.43608	-4108.90199	-4108.72882	-4109.19815	-4109.09500	-4109.55478	-4109.97817	-4110.49063
4.35	-4106.32737	-4106.78778	-4108.44127	-4108.90734	-4108.73434	-4109.20385	-4109.10059	-4109.56043	-4109.98386	-4110.49636
4.44	-4106.33252	-4106.79208	-4108.44559	-4108.91155	-4108.73888	-4109.20833	-4109.10545	-4109.56509	-4109.98803	-4110.50047
4.54	-4106.33582	-4106.79472	-4108.44822	-4108.91404	-4108.74171	-4109.21107	-4109.10861	-4109.56800	-4109.99047	-4110.50282
4.63	-4106.33759	-4106.79594	-4108.44942	-4108.91509	-4108.74309	-4109.21235	-4109.11035	-4109.56946	-4109.99150	-4110.50373
4.72	-4106.33809	-4106.79598	-4108.44945	-4108.91498	-4108.74329	-4109.21246	-4109.11092	-4109.56974	-4109.99138	-4110.50350
4.82	-4106.33757	-4106.79507	-4108.44855	-4108.91395	-4108.74255	-4109.21164	-4109.11054	-4109.56907	-4109.99034	-4110.50236
4.91	-4106.33623	-4106.79340	-4108.44688	-4108.91217	-4108.74102	-4109.21007	-4109.10938	-4109.56761	-4109.98853	-4110.50047
5.01	-4106.33425	-4106.79114	-4108.44459	-4108.90979	-4108.73886	-4109.20788	-4109.10759	-4109.56552	-4109.98612	-4110.49798
5.10	-4106.33178	-4106.78841	-4108.44183	-4108.90694	-4108.73622	-4109.20524	-4109.10530	-4109.56292	-4109.98322	-4110.49503
5.20	-4106.32895	-4106.78533	-4108.43871	-4108.90375	-4108.73321	-4109.20224	-4109.10263	-4109.55995	-4109.97995	-4110.49172
5.29	-4106.32587	-4106.78198	-4108.43533	-4108.90030	-4108.72993	-4109.19899	-4109.09966	-4109.55668	-4109.97640	-4110.48814
5.39	-4106.32262	-4106.77844	-4108.43173	-4108.89666	-4108.72643	-4109.19554	-4109.09646	-4109.55318	-4109.97262	-4110.48435
5.48	-4106.31927	-4106.77475	-4108.42799	-4108.89287	-4108.72277	-4109.19194	-4109.09308	-4109.54951	-4109.96870	-4110.48042
5.57	-4106.31588	-4106.77096	-4108.42417	-4108.88901	-4108.71903	-4109.18826	-4109.08961	-4109.54574	-4109.96470	-4110.47641
5.67	-4106.31248	-4106.76712	-4108.42035	-4108.88515	-4108.71530	-4109.18458	-4109.08612	-4109.54197	-4109.96073	-4110.47243
5.76	-4106.30911	-4106.76328	-4108.41661	-4108.88136	-4108.71163	-4109.18095	-4109.08268	-4109.53826	-4109.95686	-4110.46855
5.86	-4106.30579	-4106.75946	-4108.41304	-4108.87773	-4108.70814	-4109.17750	-4109.07941	-4109.53472	-4109.95325	-4110.46491
5.95	-4106.30254	-4106.75568	-4108.40976	-4108.87440	-4108.70494	-4109.17434	-4109.07642	-4109.53147	-4109.95010	-4110.46170
6.05	-4106.29950	-4106.74856	-4108.40493	-4108.87000	-4108.70032	-4109.17044	-4109.07172	-4109.52676	-4109.94667	-4110.45852
6.14	-4106.29673	-4106.74455	-4108.40132	-4108.86641	-4108.69678	-4109.16707	-4109.06829	-4109.52310	-4109.94304	-4110.45493

Table S12: The absolute energies in hartree of the potential energy curve of NbSi in the $^4\Pi$ state with a CASSCF wave function and nom-CPO active space.

Distance	CASSCF	CASPT2	tpbe	ftpbe	trevpbe	ftrevpbe	tblyp	ftblyp	torelyp	ftorelyp
6.24	-4106.29412	-4106.74106	-4108.39813	-4108.86322	-4108.69365	-4109.16406	-4109.06528	-4109.51987	-4109.93976	-4110.45167
6.33	-4106.28991	-4106.72610	-4108.37182	-4108.83837	-4108.66778	-4109.13938	-4109.03868	-4109.49517	-4109.90846	-4110.42255
6.43	-4106.28752	-4106.72231	-4108.36803	-4108.83459	-4108.66409	-4109.13572	-4109.03519	-4109.49162	-4109.90475	-4110.41890
6.52	-4106.28518	-4106.71883	-4108.36470	-4108.83128	-4108.66086	-4109.13251	-4109.03220	-4109.48858	-4109.90155	-4110.41575
6.61	-4106.28290	-4106.71554	-4108.36162	-4108.82821	-4108.65787	-4109.12953	-4109.02946	-4109.48581	-4109.89860	-4110.41286
6.71	-4106.28069	-4106.71239	-4108.35873	-4108.82534	-4108.65506	-4109.12673	-4109.02694	-4109.48327	-4109.89587	-4110.41019
6.80	-4106.27857	-4106.70937	-4108.35601	-4108.82265	-4108.65242	-4109.12410	-4109.02462	-4109.48095	-4109.89332	-4110.40771
6.90	-4106.27655	-4106.70644	-4108.35344	-4108.82011	-4108.64992	-4109.12162	-4109.02247	-4109.47883	-4109.89094	-4110.40541
6.99	-4106.27465	-4106.70357	-4108.35099	-4108.81771	-4108.64755	-4109.11926	-4109.02049	-4109.47692	-4109.88871	-4110.40327
7.09	-4106.27289	-4106.70076	-4108.34864	-4108.81543	-4108.64527	-4109.11700	-4109.01867	-4109.47522	-4109.88661	-4110.40127
7.18	-4106.27129	-4106.69814	-4108.34639	-4108.81326	-4108.64309	-4109.11485	-4109.01699	-4109.47367	-4109.88464	-4110.39939
7.28	-4106.26986	-4106.69577	-4108.34425	-4108.81119	-4108.64101	-4109.11278	-4109.01540	-4109.47223	-4109.88278	-4110.39761
7.37	-4106.26858	-4106.69363	-4108.34220	-4108.80920	-4108.63902	-4109.11081	-4109.01390	-4109.47088	-4109.88102	-4110.39592
7.46	-4106.26744	-4106.69171	-4108.34025	-4108.80731	-4108.63713	-4109.10892	-4109.01246	-4109.46959	-4109.87936	-4110.39431
7.56	-4106.26642	-4106.68998	-4108.33840	-4108.80551	-4108.63535	-4109.10714	-4109.01110	-4109.46836	-4109.87781	-4110.39279
7.65	-4106.26552	-4106.68842	-4108.33666	-4108.80382	-4108.63367	-4109.10547	-4109.00981	-4109.46720	-4109.87636	-4110.39137
7.75	-4106.26472	-4106.68701	-4108.33503	-4108.80224	-4108.63211	-4109.10391	-4109.00860	-4109.46611	-4109.87501	-4110.39004
7.84	-4106.26402	-4106.68574	-4108.33351	-4108.80076	-4108.63066	-4109.10247	-4109.00746	-4109.46508	-4109.87378	-4110.38882
7.94	-4106.26339	-4106.68459	-4108.33211	-4108.79938	-4108.62933	-4109.10113	-4109.00640	-4109.46413	-4109.87264	-4110.38769
8.03	-4106.26284	-4106.68355	-4108.33081	-4108.79812	-4108.62810	-4109.09991	-4109.00542	-4109.46324	-4109.87161	-4110.38666
8.13	-4106.26235	-4106.68262	-4108.32962	-4108.79696	-4108.62698	-4109.09879	-4109.00451	-4109.46241	-4109.87068	-4110.38573
8.22	-4106.26192	-4106.68178	-4108.32853	-4108.79589	-4108.62596	-4109.09777	-4109.00368	-4109.46165	-4109.86984	-4110.38489
8.31	-4106.26154	-4106.68102	-4108.32754	-4108.79492	-4108.62504	-4109.09685	-4109.00291	-4109.46096	-4109.86910	-4110.38414
8.41	-4106.26120	-4106.68033	-4108.32664	-4108.79404	-4108.62422	-4109.09603	-4109.00222	-4109.46032	-4109.86844	-4110.38347
8.50	-4106.26090	-4106.67971	-4108.32584	-4108.79324	-4108.62348	-4109.09529	-4109.00160	-4109.45975	-4109.86786	-4110.38288
8.60	-4106.26063	-4106.67915	-4108.32511	-4108.79253	-4108.62283	-4109.09463	-4109.00105	-4109.45924	-4109.86736	-4110.38236
8.69	-4106.26040	-4106.67864	-4108.32446	-4108.79188	-4108.62224	-4109.09404	-4109.00054	-4109.45877	-4109.86692	-4110.38191
8.79	-4106.26018	-4106.67819	-4108.32388	-4108.79131	-4108.62173	-4109.09352	-4109.00010	-4109.45836	-4109.86654	-4110.38151
8.88	-4106.26000	-4106.67778	-4108.32337	-4108.79080	-4108.62128	-4109.09306	-4108.99972	-4109.45799	-4109.86621	-4110.38116
8.98	-4106.25983	-4106.67740	-4108.32290	-4108.79032	-4108.62087	-4109.09264	-4108.99936	-4109.45765	-4109.86593	-4110.38086
9.07	-4106.25968	-4106.67706	-4108.32250	-4108.78991	-4108.62052	-4109.09228	-4108.99906	-4109.45736	-4109.86569	-4110.38060
9.17	-4106.25954	-4106.67675	-4108.32213	-4108.78954	-4108.62021	-4109.09195	-4108.99879	-4109.45709	-4109.86548	-4110.38037
9.26	-4106.25942	-4106.67647	-4108.32180	-4108.78920	-4108.61993	-4109.09166	-4108.99855	-4109.45685	-4109.86531	-4110.38017
9.35	-4106.25931	-4106.67621	-4108.32150	-4108.78890	-4108.61969	-4109.09140	-4108.99833	-4109.45663	-4109.86516	-4110.38000
9.45	-4106.25921	-4106.67597	-4108.32124	-4108.78862	-4108.61948	-4109.09118	-4108.99814	-4109.45644	-4109.86504	-4110.37985
9.54	-4106.25912	-4106.67575	-4108.32101	-4108.78838	-4108.61930	-4109.09098	-4108.99798	-4109.45628	-4109.86494	-4110.37973
9.64	-4106.25904	-4106.67555	-4108.32080	-4108.78817	-4108.61914	-4109.09080	-4108.99784	-4109.45613	-4109.86486	-4110.37962
9.73	-4106.25896	-4106.67536	-4108.32061	-4108.78797	-4108.61899	-4109.09065	-4108.99771	-4109.45600	-4109.86479	-4110.37953
9.83	-4106.25890	-4106.67518	-4108.32044	-4108.78779	-4108.61887	-4109.09051	-4108.99760	-4109.45588	-4109.86473	-4110.37945
9.92	-4106.25883	-4106.67502	-4108.32029	-4108.78762	-4108.61875	-4109.09038	-4108.99750	-4109.45577	-4109.86468	-4110.37938
10.02	-4106.25878	-4106.67488	-4108.32015	-4108.78748	-4108.61866	-4109.09027	-4108.99742	-4109.45568	-4109.86465	-4110.37933
10.11	-4106.25873	-4106.67474	-4108.32002	-4108.78734	-4108.61857	-4109.09017	-4108.99734	-4109.45559	-4109.86461	-4110.37928
10.20	-4106.25868	-4106.67461	-4108.31991	-4108.78722	-4108.61848	-4109.09007	-4108.99727	-4109.45551	-4109.86459	-4110.37924
10.30	-4106.25864	-4106.67449	-4108.31981	-4108.78711	-4108.61842	-4109.09000	-4108.99721	-4109.45545	-4109.86457	-4110.37920
10.39	-4106.25860	-4106.67438	-4108.31972	-4108.78701	-4108.61836	-4109.08992	-4108.99716	-4109.45539	-4109.86456	-4110.37917
10.49	-4106.25856	-4106.67427	-4108.31963	-4108.78692	-4108.61831	-4109.08986	-4108.99711	-4109.45534	-4109.86455	-4110.37914
10.58	-4106.25853	-4106.67418	-4108.31956	-4108.78684	-4108.61826	-4109.08981	-4108.99708	-4109.45529	-4109.86455	-4110.37912
10.68	-4106.25850	-4106.67409	-4108.31950	-4108.78677	-4108.61823	-4109.08976	-4108.99705	-4109.45526	-4109.86455	-4110.37911
10.77	-4106.25847	-4106.67400	-4108.31944	-4108.78670	-4108.61819	-4109.08972	-4108.99702	-4109.45522	-4109.86455	-4110.37909
10.87	-4106.25845	-4106.67392	-4108.31938	-4108.78664	-4108.61816	-4109.08968	-4108.99700	-4109.45519	-4109.86455	-4110.37908
10.96	-4106.25842	-4106.67384	-4108.31933	-4108.78658	-4108.61813	-4109.08964	-4108.99698	-4109.45517	-4109.86455	-4110.37907
11.05	-4106.25840	-4106.67377	-4108.31928	-4108.78653	-4108.61811	-4109.08961	-4108.99696	-4109.45514	-4109.86455	-4110.37906

Table S12: The absolute energies in hartree of the potential energy curve of NbSi in the $^4\Pi$ state with a CASSCF wave function and nom-CPO active space.

Distance	CASSCF	CASPT2	tpbe	ftpbe	trevpbe	ftrevpbe	tblyp	ftblyp	torelyp	ftorelyp
11.15	-4106.25838	-4106.67370	-4108.31924	-4108.78648	-4108.61808	-4109.08958	-4108.99695	-4109.45512	-4109.86455	-4110.37905
11.24	-4106.25836	-4106.67364	-4108.31920	-4108.78643	-4108.61806	-4109.08955	-4108.99693	-4109.45510	-4109.86455	-4110.37904
11.34	-4106.25835	-4106.67358	-4108.31916	-4108.78639	-4108.61804	-4109.08952	-4108.99692	-4109.45508	-4109.86456	-4110.37903
11.43	-4106.25833	-4106.67352	-4108.31913	-4108.78635	-4108.61803	-4109.08949	-4108.99691	-4109.45507	-4109.86456	-4110.37902
11.53	-4106.25831	-4106.67347	-4108.31910	-4108.78632	-4108.61801	-4109.08947	-4108.99691	-4109.45506	-4109.86456	-4110.37901
11.62	-4106.25830	-4106.67342	-4108.31907	-4108.78629	-4108.61800	-4109.08946	-4108.99690	-4109.45505	-4109.86456	-4110.37901
11.72	-4106.25829	-4106.67337	-4108.31905	-4108.78626	-4108.61799	-4109.08944	-4108.99690	-4109.45504	-4109.86457	-4110.37900
11.81	-4106.25828	-4106.67332	-4108.31903	-4108.78624	-4108.61798	-4109.08943	-4108.99690	-4109.45504	-4109.86457	-4110.37900
11.91	-4106.25826	-4106.67328	-4108.31901	-4108.78621	-4108.61797	-4109.08942	-4108.99690	-4109.45504	-4109.86458	-4110.37900
12.00	-4106.25825	-4106.67324	-4108.31899	-4108.78619	-4108.61797	-4109.08941	-4108.99690	-4109.45503	-4109.86458	-4110.37900
12.09	-4106.25824	-4106.67320	-4108.31897	-4108.78618	-4108.61796	-4109.08940	-4108.99690	-4109.45503	-4109.86459	-4110.37899
12.19	-4106.25824	-4106.67316	-4108.31895	-4108.78615	-4108.61795	-4109.08938	-4108.99690	-4109.45503	-4109.86459	-4110.37899
12.28	-4106.25823	-4106.67312	-4108.31894	-4108.78613	-4108.61794	-4109.08937	-4108.99690	-4109.45503	-4109.86459	-4110.37899
12.38	-4106.25821	-4106.67301	-4108.31890	-4108.78611	-4108.61791	-4109.08936	-4108.99688	-4109.45503	-4109.86457	-4110.37898
12.47	-4106.25821	-4106.67306	-4108.31892	-4108.78611	-4108.61794	-4109.08936	-4108.99691	-4109.45503	-4109.86460	-4110.37898
12.57	-4106.25821	-4106.67303	-4108.31890	-4108.78609	-4108.61793	-4109.08934	-4108.99691	-4109.45503	-4109.86460	-4110.37897
12.66	-4106.25820	-4106.67300	-4108.31889	-4108.78607	-4108.61793	-4109.08933	-4108.99692	-4109.45503	-4109.86460	-4110.37896
12.76	-4106.25819	-4106.67297	-4108.31888	-4108.78606	-4108.61792	-4109.08933	-4108.99692	-4109.45503	-4109.86460	-4110.37896
12.85	-4106.25819	-4106.67294	-4108.31887	-4108.78605	-4108.61792	-4109.08932	-4108.99692	-4109.45503	-4109.86460	-4110.37896
12.94	-4106.25818	-4106.67291	-4108.31886	-4108.78604	-4108.61791	-4109.08931	-4108.99693	-4109.45503	-4109.86460	-4110.37895
13.04	-4106.25818	-4106.67289	-4108.31885	-4108.78603	-4108.61791	-4109.08931	-4108.99693	-4109.45504	-4109.86460	-4110.37895
13.13	-4106.25817	-4106.67286	-4108.31884	-4108.78602	-4108.61790	-4109.08930	-4108.99693	-4109.45503	-4109.86459	-4110.37894
13.23	-4106.25817	-4106.67284	-4108.31883	-4108.78601	-4108.61790	-4109.08930	-4108.99693	-4109.45504	-4109.86459	-4110.37894
13.32	-4106.25816	-4106.67282	-4108.31883	-4108.78601	-4108.61790	-4109.08929	-4108.99694	-4109.45504	-4109.86459	-4110.37894
13.42	-4106.25816	-4106.67280	-4108.31883	-4108.78600	-4108.61790	-4109.08929	-4108.99695	-4109.45505	-4109.86460	-4110.37894
13.51	-4106.25815	-4106.67278	-4108.31882	-4108.78600	-4108.61790	-4109.08929	-4108.99695	-4109.45505	-4109.86460	-4110.37894
13.61	-4106.25815	-4106.67276	-4108.31882	-4108.78599	-4108.61790	-4109.08929	-4108.99696	-4109.45506	-4109.86460	-4110.37894
13.70	-4106.25815	-4106.67274	-4108.31881	-4108.78599	-4108.61789	-4109.08929	-4108.99696	-4109.45506	-4109.86460	-4110.37894
13.80	-4106.25814	-4106.67272	-4108.31881	-4108.78598	-4108.61789	-4109.08928	-4108.99696	-4109.45506	-4109.86460	-4110.37893
13.89	-4106.25814	-4106.67270	-4108.31881	-4108.78598	-4108.61789	-4109.08928	-4108.99697	-4109.45507	-4109.86460	-4110.37893
13.98	-4106.25814	-4106.67269	-4108.31880	-4108.78597	-4108.61789	-4109.08927	-4108.99697	-4109.45507	-4109.86460	-4110.37893
14.08	-4106.25813	-4106.67267	-4108.31880	-4108.78597	-4108.61789	-4109.08928	-4108.99698	-4109.45507	-4109.86461	-4110.37893
14.17	-4106.25813	-4106.67266	-4108.31880	-4108.78597	-4108.61789	-4109.08927	-4108.99698	-4109.45507	-4109.86460	-4110.37892
14.27	-4106.25813	-4106.67264	-4108.31879	-4108.78596	-4108.61788	-4109.08926	-4108.99698	-4109.45507	-4109.86460	-4110.37892
14.36	-4106.25813	-4106.67262	-4108.31879	-4108.78595	-4108.61788	-4109.08926	-4108.99698	-4109.45507	-4109.86460	-4110.37891
14.46	-4106.25812	-4106.67261	-4108.31878	-4108.78595	-4108.61788	-4109.08926	-4108.99699	-4109.45508	-4109.86459	-4110.37891
14.55	-4106.25812	-4106.67259	-4108.31878	-4108.78595	-4108.61787	-4109.08925	-4108.99699	-4109.45508	-4109.86459	-4110.37891
14.65	-4106.25812	-4106.67258	-4108.31878	-4108.78594	-4108.61787	-4109.08925	-4108.99699	-4109.45508	-4109.86459	-4110.37891
14.74	-4106.25812	-4106.67257	-4108.31877	-4108.78594	-4108.61787	-4109.08925	-4108.99699	-4109.45508	-4109.86459	-4110.37891
14.83	-4106.25812	-4106.67256	-4108.31877	-4108.78594	-4108.61787	-4109.08925	-4108.99700	-4109.45509	-4109.86459	-4110.37891
14.93	-4106.25811	-4106.67254	-4108.31877	-4108.78594	-4108.61787	-4109.08925	-4108.99700	-4109.45509	-4109.86459	-4110.37891
15.02	-4106.25811	-4106.67253	-4108.31877	-4108.78594	-4108.61787	-4109.08925	-4108.99700	-4109.45510	-4109.86459	-4110.37891
15.12	-4106.25811	-4106.67252	-4108.31876	-4108.78593	-4108.61786	-4109.08924	-4108.99700	-4109.45509	-4109.86458	-4110.37890
15.21	-4106.25811	-4106.67251	-4108.31876	-4108.78593	-4108.61786	-4109.08924	-4108.99700	-4109.45509	-4109.86458	-4110.37890
15.31	-4106.25811	-4106.67250	-4108.31876	-4108.78593	-4108.61786	-4109.08924	-4108.99701	-4109.45510	-4109.86458	-4110.37890
15.40	-4106.25811	-4106.67249	-4108.31876	-4108.78593	-4108.61786	-4109.08924	-4108.99701	-4109.45510	-4109.86458	-4110.37890
15.50	-4106.25810	-4106.67248	-4108.31876	-4108.78593	-4108.61786	-4109.08924	-4108.99701	-4109.45510	-4109.86458	-4110.37890
15.59	-4106.25810	-4106.67247	-4108.31876	-4108.78593	-4108.61786	-4109.08924	-4108.99701	-4109.45510	-4109.86458	-4110.37890
15.68	-4106.25810	-4106.67246	-4108.31876	-4108.78593	-4108.61786	-4109.08924	-4108.99701	-4109.45510	-4109.86458	-4110.37890
15.78	-4106.25810	-4106.67245	-4108.31876	-4108.78592	-4108.61786	-4109.08923	-4108.99701	-4109.45510	-4109.86458	-4110.37889
15.87	-4106.25810	-4106.67245	-4108.31875	-4108.78592	-4108.61786	-4109.08923	-4108.99702	-4109.45510	-4109.86458	-4110.37889
15.97	-4106.25810	-4106.67244	-4108.31875	-4108.78592	-4108.61786	-4109.08923	-4108.99702	-4109.45510	-4109.86458	-4110.37889

Table S12: The absolute energies in hartree of the potential energy curve of NbSi in the $^4\Pi$ state with a CASSCF wave function and nom-CPO active space.

Distance	CASSCF	CASPT2	tpbe	ftpbe	trevpbe	ftrevpbe	tblyp	ftblyp	torelyp	ftorelyp
16.06	-4106.25810	-4106.67243	-4108.31875	-4108.78592	-4108.61786	-4109.08923	-4108.99702	-4109.45511	-4109.86458	-4110.37889
16.16	-4106.25810	-4106.67243	-4108.31875	-4108.78592	-4108.61785	-4109.08923	-4108.99702	-4109.45511	-4109.86458	-4110.37889
16.25	-4106.25810	-4106.67242	-4108.31875	-4108.78592	-4108.61785	-4109.08923	-4108.99702	-4109.45511	-4109.86458	-4110.37889
16.35	-4106.25809	-4106.67242	-4108.31875	-4108.78592	-4108.61785	-4109.08923	-4108.99702	-4109.45511	-4109.86458	-4110.37889
16.44	-4106.25809	-4106.67241	-4108.31875	-4108.78592	-4108.61785	-4109.08923	-4108.99702	-4109.45511	-4109.86458	-4110.37889
16.54	-4106.25809	-4106.67240	-4108.31875	-4108.78591	-4108.61785	-4109.08923	-4108.99702	-4109.45511	-4109.86458	-4110.37889
16.63	-4106.25809	-4106.67240	-4108.31875	-4108.78591	-4108.61785	-4109.08923	-4108.99703	-4109.45511	-4109.86457	-4110.37889
16.72	-4106.25809	-4106.67239	-4108.31875	-4108.78591	-4108.61785	-4109.08923	-4108.99703	-4109.45511	-4109.86457	-4110.37889
16.82	-4106.25809	-4106.67239	-4108.31875	-4108.78591	-4108.61785	-4109.08923	-4108.99703	-4109.45511	-4109.86457	-4110.37889
16.91	-4106.25809	-4106.67239	-4108.31875	-4108.78591	-4108.61785	-4109.08923	-4108.99703	-4109.45511	-4109.86458	-4110.37889
17.01	-4106.25809	-4106.67238	-4108.31875	-4108.78591	-4108.61785	-4109.08923	-4108.99703	-4109.45512	-4109.86457	-4110.37888
17.10	-4106.25809	-4106.67238	-4108.31875	-4108.78591	-4108.61785	-4109.08923	-4108.99703	-4109.45512	-4109.86457	-4110.37888
17.20	-4106.25809	-4106.67237	-4108.31875	-4108.78592	-4108.61785	-4109.08923	-4108.99703	-4109.45512	-4109.86458	-4110.37889
17.29	-4106.25809	-4106.67237	-4108.31875	-4108.78592	-4108.61785	-4109.08923	-4108.99704	-4109.45512	-4109.86458	-4110.37889
17.39	-4106.25809	-4106.67237	-4108.31875	-4108.78592	-4108.61786	-4109.08923	-4108.99704	-4109.45512	-4109.86458	-4110.37889
17.48	-4106.25809	-4106.67236	-4108.31875	-4108.78592	-4108.61786	-4109.08923	-4108.99704	-4109.45512	-4109.86458	-4110.37889
17.57	-4106.25809	-4106.67236	-4108.31875	-4108.78592	-4108.61786	-4109.08923	-4108.99704	-4109.45512	-4109.86458	-4110.37889
17.67	-4106.25808	-4106.67236	-4108.31875	-4108.78592	-4108.61786	-4109.08923	-4108.99704	-4109.45512	-4109.86458	-4110.37889
17.76	-4106.25808	-4106.67235	-4108.31875	-4108.78592	-4108.61785	-4109.08923	-4108.99704	-4109.45512	-4109.86458	-4110.37889
17.86	-4106.25808	-4106.67235	-4108.31875	-4108.78592	-4108.61785	-4109.08923	-4108.99704	-4109.45512	-4109.86458	-4110.37889
17.95	-4106.25808	-4106.67235	-4108.31875	-4108.78591	-4108.61785	-4109.08923	-4108.99704	-4109.45512	-4109.86458	-4110.37888
18.05	-4106.25808	-4106.67234	-4108.31875	-4108.78591	-4108.61785	-4109.08923	-4108.99704	-4109.45512	-4109.86458	-4110.37888

Table S13: The absolute energies in hartree of the potential energy curve of NbSi in the $^6\Sigma^+$ state with a CASSCF wave function and nom-CPO active space.

Distance	CASSCF	CASPT2	tpbe	ftpbe	trevpbe	ftrevpbe	tblyp	ftblyp	torelyp	ftorelyp
1.98	-4100.79633	-4102.21485	-4103.88577	-4104.35732	-4104.16275	-4104.63842	-4104.52495	-4104.98834	-4105.45846	-4105.97683
2.08	-4101.69595	-4103.06915	-4104.73184	-4105.20292	-4105.00997	-4105.48522	-4105.37244	-4105.83510	-4106.30103	-4106.81895
2.17	-4102.54888	-4103.76125	-4105.41586	-4105.88649	-4105.69512	-4106.16996	-4106.05784	-4106.51982	-4106.98178	-4107.49928
2.27	-4103.24033	-4104.31920	-4105.96671	-4106.43698	-4106.24709	-4106.72160	-4106.61005	-4107.07147	-4107.52963	-4108.04684
2.36	-4103.79813	-4104.76784	-4106.40953	-4106.87955	-4106.69096	-4107.16527	-4107.05417	-4107.51519	-4107.96970	-4108.48679
2.46	-4104.24700	-4105.12851	-4106.76593	-4107.23583	-4107.04837	-4107.52262	-4107.41177	-4107.87254	-4108.32362	-4108.84078
2.55	-4104.60824	-4105.41893	-4107.05382	-4107.52374	-4107.33721	-4107.81156	-4107.70067	-4108.16135	-4108.60915	-4109.12659
2.65	-4104.89973	-4105.65221	-4107.28788	-4107.75805	-4107.57220	-4108.04694	-4107.93526	-4108.39606	-4108.84064	-4109.35875
2.74	-4105.13646	-4105.83649	-4107.47721	-4107.94820	-4107.76252	-4108.23828	-4108.12456	-4108.58563	-4109.02735	-4109.54681
2.83	-4105.33205	-4105.99178	-4107.63494	-4108.10657	-4107.92111	-4108.39770	-4108.28248	-4108.74359	-4109.18317	-4109.70370
2.93	-4105.49658	-4106.12527	-4107.76899	-4108.24092	-4108.05584	-4108.53294	-4108.41684	-4108.87782	-4109.31536	-4109.83667
3.02	-4105.63510	-4106.23913	-4107.88283	-4108.35481	-4108.17024	-4108.64762	-4108.53104	-4108.99177	-4109.42735	-4109.94924
3.12	-4105.75175	-4106.33143	-4107.97714	-4108.44829	-4108.26516	-4108.74151	-4108.62709	-4109.08784	-4109.52331	-4110.04316
3.21	-4105.83513	-4106.41466	-4108.05978	-4108.53058	-4108.34809	-4108.82440	-4108.71007	-4109.17024	-4109.60402	-4110.12393
3.31	-4105.92100	-4106.48552	-4108.13015	-4108.60047	-4108.41874	-4108.89477	-4108.78085	-4109.24046	-4109.67276	-4110.19238
3.40	-4105.99411	-4106.54556	-4108.18978	-4108.65959	-4108.47866	-4108.95427	-4108.84102	-4109.30006	-4109.73107	-4110.25019
3.50	-4106.05627	-4106.59616	-4108.24016	-4108.70945	-4108.52934	-4109.00447	-4108.89199	-4109.35050	-4109.78040	-4110.29895
3.59	-4106.10897	-4106.63859	-4108.28252	-4108.75132	-4108.57202	-4109.04664	-4108.93499	-4109.39300	-4109.82191	-4110.33988
3.68	-4106.15345	-4106.67395	-4108.31795	-4108.78625	-4108.60775	-4109.08186	-4108.97109	-4109.42862	-4109.85661	-4110.37402
3.78	-4106.19081	-4106.70322	-4108.34744	-4108.81526	-4108.63755	-4109.11113	-4109.00129	-4109.45835	-4109.88552	-4110.40234
3.87	-4106.22199	-4106.72728	-4108.37192	-4108.83922	-4108.66232	-4109.13533	-4109.02651	-4109.48308	-4109.90948	-4110.42571
3.97	-4106.24782	-4106.74692	-4108.39213	-4108.85887	-4108.68281	-4109.15518	-4109.04751	-4109.50356	-4109.92923	-4110.44480
4.06	-4106.26905	-4106.76276	-4108.40859	-4108.87479	-4108.69953	-4109.17130	-4109.06475	-4109.52030	-4109.94526	-4110.46023
4.16	-4106.28632	-4106.77524	-4108.42165	-4108.88742	-4108.71284	-4109.18414	-4109.07853	-4109.53366	-4109.95797	-4110.47244

Table S13: The absolute energies in hartree of the potential energy curve of NbSi in the $^6\Sigma^+$ state with a CASSCF wave function and nom-CPO active space.

Distance	CASSCF	CASPT2	tpbe	ftpbe	trevpbe	ftrevpbe	tblyp	ftblyp	torelyp	ftorelyp
4.35	-4106.30018	-4106.78483	-4108.43178	-4108.89721	-4108.72321	-4109.19413	-4109.08932	-4109.54412	-4109.96780	-4110.48187
4.44	-4106.31111	-4106.79194	-4108.43940	-4108.90455	-4108.73106	-4109.20168	-4109.09757	-4109.55209	-4109.97519	-4110.48892
4.54	-4106.31952	-4106.79698	-4108.44489	-4108.90981	-4108.73678	-4109.20715	-4109.10366	-4109.55796	-4109.98051	-4110.49395
4.63	-4106.32577	-4106.80026	-4108.44860	-4108.91333	-4108.74071	-4109.21086	-4109.10795	-4109.56205	-4109.98409	-4110.49728
4.72	-4106.33018	-4106.80207	-4108.45084	-4108.91540	-4108.74314	-4109.21312	-4109.11074	-4109.56468	-4109.98624	-4110.49921
4.82	-4106.33304	-4106.80265	-4108.45183	-4108.91626	-4108.74433	-4109.21416	-4109.11228	-4109.56608	-4109.98718	-4110.49996
4.91	-4106.33459	-4106.80220	-4108.45178	-4108.91610	-4108.74447	-4109.21417	-4109.11276	-4109.56645	-4109.98713	-4110.49973
5.01	-4106.33505	-4106.80092	-4108.45086	-4108.91509	-4108.74373	-4109.21333	-4109.11237	-4109.56595	-4109.98623	-4110.49869
5.10	-4106.33459	-4106.79895	-4108.44925	-4108.91340	-4108.74229	-4109.21180	-4109.11126	-4109.56476	-4109.98466	-4110.49699
5.20	-4106.33337	-4106.79643	-4108.44708	-4108.91118	-4108.74028	-4109.20973	-4109.10959	-4109.56302	-4109.98257	-4110.49478
5.29	-4106.33154	-4106.79347	-4108.44448	-4108.90853	-4108.73784	-4109.20724	-4109.10747	-4109.56085	-4109.98007	-4110.49218
5.39	-4106.32922	-4106.79017	-4108.44153	-4108.90555	-4108.73503	-4109.20440	-4109.10499	-4109.55832	-4109.97723	-4110.48926
5.48	-4106.32649	-4106.78662	-4108.43830	-4108.90230	-4108.73195	-4109.20129	-4109.10222	-4109.55552	-4109.97414	-4110.48610
5.57	-4106.32346	-4106.78290	-4108.43489	-4108.89889	-4108.72868	-4109.19801	-4109.09926	-4109.55253	-4109.97088	-4110.48280
5.67	-4106.32020	-4106.75452	-4108.40063	-4108.86502	-4108.69531	-4109.16533	-4109.06315	-4109.51545	-4109.93588	-4110.44690
5.76	-4106.29507	-4106.75089	-4108.39763	-4108.86204	-4108.69246	-4109.16256	-4109.06046	-4109.51271	-4109.93288	-4110.44397
5.86	-4106.29317	-4106.74730	-4108.39468	-4108.85911	-4108.68965	-4109.15982	-4109.05782	-4109.51002	-4109.92997	-4110.44111
5.95	-4106.29117	-4106.74381	-4108.39184	-4108.85630	-4108.68696	-4109.15718	-4109.05529	-4109.50744	-4109.92719	-4110.43839
6.05	-4106.28910	-4106.74045	-4108.38916	-4108.85362	-4108.68440	-4109.15466	-4109.05292	-4109.50500	-4109.92460	-4110.43584
6.14	-4106.28698	-4106.73726	-4108.38670	-4108.85115	-4108.68205	-4109.15234	-4109.05076	-4109.50276	-4109.92224	-4110.43351
6.24	-4106.28482	-4106.73428	-4108.38455	-4108.84898	-4108.68000	-4109.15031	-4109.04891	-4109.50080	-4109.92023	-4110.43151
6.33	-4106.28265	-4106.73172	-4108.38289	-4108.84726	-4108.67842	-4109.14873	-4109.04754	-4109.49926	-4109.91874	-4110.43000
6.43	-4106.28049	-4106.72952	-4108.38191	-4108.84616	-4108.67749	-4109.14775	-4109.04684	-4109.49828	-4109.91797	-4110.42912
6.52	-4106.27836	-4106.72824	-4108.38227	-4108.84629	-4108.67786	-4109.14797	-4109.04746	-4109.49838	-4109.91864	-4110.42951
6.61	-4106.27630	-4106.72763	-4108.38357	-4108.84723	-4108.67909	-4109.14898	-4109.04901	-4109.49917	-4109.92034	-4110.43069
6.71	-4106.27438	-4106.72634	-4108.38345	-4108.84689	-4108.67892	-4109.14870	-4109.04918	-4109.49886	-4109.92048	-4110.43045
6.80	-4106.27267	-4106.72463	-4108.38224	-4108.84556	-4108.67769	-4109.14744	-4109.04827	-4109.49770	-4109.91942	-4110.42921
6.90	-4106.27117	-4106.72278	-4108.38070	-4108.84395	-4108.67615	-4109.14591	-4109.04704	-4109.49634	-4109.91795	-4110.42764
6.99	-4106.26987	-4106.72095	-4108.37903	-4108.84224	-4108.67449	-4109.14427	-4109.04567	-4109.49489	-4109.91633	-4110.42596
7.09	-4106.26874	-4106.71920	-4108.37736	-4108.84053	-4108.67284	-4109.14263	-4109.04428	-4109.49345	-4109.91469	-4110.42429
7.18	-4106.26774	-4106.71756	-4108.37574	-4108.83888	-4108.67126	-4109.14105	-4109.04292	-4109.49206	-4109.91312	-4110.42268
7.28	-4106.26687	-4106.71603	-4108.37421	-4108.83731	-4108.66976	-4109.13956	-4109.04163	-4109.49075	-4109.91164	-4110.42117
7.37	-4106.26611	-4106.71463	-4108.37277	-4108.83585	-4108.66838	-4109.13817	-4109.04042	-4109.48952	-4109.91028	-4110.41978
7.46	-4106.26544	-4106.71335	-4108.37145	-4108.83449	-4108.66711	-4109.13688	-4109.03931	-4109.48838	-4109.90904	-4110.41849
7.56	-4106.26485	-4106.71217	-4108.37024	-4108.83324	-4108.66594	-4109.13570	-4109.03829	-4109.48733	-4109.90791	-4110.41732
7.65	-4106.26433	-4106.71109	-4108.36913	-4108.83210	-4108.66490	-4109.13462	-4109.03736	-4109.48638	-4109.90691	-4110.41627
7.75	-4106.26387	-4106.71011	-4108.36813	-4108.83106	-4108.66395	-4109.13365	-4109.03652	-4109.48551	-4109.90602	-4110.41533
7.84	-4106.26346	-4106.68584	-4108.33291	-4108.80013	-4108.63040	-4109.10175	-4109.00717	-4109.46531	-4109.87582	-4110.39012
7.94	-4106.25270	-4106.68504	-4108.33194	-4108.79918	-4108.62949	-4109.10083	-4109.00661	-4109.46490	-4109.87519	-4110.38948
8.03	-4106.25219	-4106.68432	-4108.33108	-4108.79834	-4108.62869	-4109.10002	-4109.00610	-4109.46448	-4109.87460	-4110.38888
8.13	-4106.25175	-4106.68367	-4108.33030	-4108.79758	-4108.62797	-4109.09930	-4109.00563	-4109.46408	-4109.87407	-4110.38834
8.22	-4106.25137	-4106.68307	-4108.32959	-4108.79688	-4108.62733	-4109.09865	-4109.00519	-4109.46369	-4109.87358	-4110.38785
8.31	-4106.25103	-4106.68253	-4108.32895	-4108.79625	-4108.62675	-4109.09807	-4109.00478	-4109.46332	-4109.87315	-4110.38741
8.41	-4106.25073	-4106.68203	-4108.32837	-4108.79567	-4108.62622	-4109.09754	-4109.00442	-4109.46298	-4109.87277	-4110.38701
8.50	-4106.25046	-4106.68156	-4108.32784	-4108.79514	-4108.62575	-4109.09706	-4109.00408	-4109.46266	-4109.87243	-4110.38666
8.60	-4106.25022	-4106.68112	-4108.32736	-4108.79466	-4108.62533	-4109.09663	-4109.00378	-4109.46237	-4109.87213	-4110.38635
8.69	-4106.25000	-4106.68072	-4108.32693	-4108.79421	-4108.62495	-4109.09624	-4109.00350	-4109.46210	-4109.87187	-4110.38607
8.79	-4106.24981	-4106.68035	-4108.32652	-4108.79380	-4108.62460	-4109.09588	-4109.00324	-4109.46185	-4109.87163	-4110.38582
8.88	-4106.24963	-4106.68001	-4108.32615	-4108.79343	-4108.62429	-4109.09556	-4109.00301	-4109.46162	-4109.87143	-4110.38560
8.98	-4106.24947	-4106.67969	-4108.32582	-4108.79308	-4108.62400	-4109.09526	-4109.00280	-4109.46141	-4109.87124	-4110.38540
9.07	-4106.24933	-4106.67940	-4108.32551	-4108.79276	-4108.62374	-4109.09499	-4109.00260	-4109.46121	-4109.87108	-4110.38523
9.17	-4106.24919	-4106.67912	-4108.32522	-4108.79247	-4108.62351	-4109.09475	-4109.00242	-4109.46103	-4109.87095	-4110.38507

Table S13: The absolute energies in hartree of the potential energy curve of NbSi in the $^6\Sigma^+$ state with a CASSCF wave function and nom-CPO active space.

Distance	CASSCF	CASPT2	tpbe	ftpbe	trevpbe	ftrevpbe	tblyp	ftblyp	torelyp	ftorelyp
9.26	-4106.24907	-4106.67887	-4108.32496	-4108.79220	-4108.62330	-4109.09453	-4109.00226	-4109.46087	-4109.87083	-4110.38493
9.35	-4106.24896	-4106.67863	-4108.32473	-4108.79196	-4108.62312	-4109.09433	-4109.00212	-4109.46072	-4109.87073	-4110.38482
9.45	-4106.24886	-4106.67841	-4108.32453	-4108.79174	-4108.62296	-4109.09416	-4109.00200	-4109.46059	-4109.87065	-4110.38472
9.54	-4106.24877	-4106.67820	-4108.32434	-4108.79155	-4108.62282	-4109.09401	-4109.00189	-4109.46047	-4109.87059	-4110.38463
9.64	-4106.24869	-4106.67801	-4108.32417	-4108.79137	-4108.62269	-4109.09387	-4109.00180	-4109.46037	-4109.87054	-4110.38456
9.73	-4106.24861	-4106.67783	-4108.32402	-4108.79120	-4108.62258	-4109.09374	-4109.00171	-4109.46027	-4109.87050	-4110.38450
9.83	-4106.24854	-4106.67767	-4108.32387	-4108.79105	-4108.62247	-4109.09363	-4109.00163	-4109.46018	-4109.87046	-4110.38444
9.92	-4106.24848	-4106.67751	-4108.32374	-4108.79090	-4108.62238	-4109.09352	-4109.00156	-4109.46010	-4109.87043	-4110.38439
10.02	-4106.24842	-4106.67737	-4108.32362	-4108.79077	-4108.62230	-4109.09342	-4109.00150	-4109.46003	-4109.87040	-4110.38435
10.11	-4106.24836	-4106.67723	-4108.32351	-4108.79065	-4108.62222	-4109.09333	-4109.00144	-4109.45996	-4109.87038	-4110.38431
10.20	-4106.24831	-4106.67711	-4108.32341	-4108.79054	-4108.62215	-4109.09325	-4109.00139	-4109.45990	-4109.87037	-4110.38428
10.30	-4106.24827	-4106.67698	-4108.32331	-4108.79044	-4108.62209	-4109.09318	-4109.00135	-4109.45985	-4109.87036	-4110.38426
10.39	-4106.24822	-4106.67687	-4108.32323	-4108.79035	-4108.62204	-4109.09312	-4109.00131	-4109.45980	-4109.87035	-4110.38423
10.49	-4106.24818	-4106.67677	-4108.32316	-4108.79027	-4108.62200	-4109.09307	-4109.00128	-4109.45977	-4109.87035	-4110.38421
10.58	-4106.24815	-4106.67667	-4108.32310	-4108.79020	-4108.62197	-4109.09303	-4109.00125	-4109.45973	-4109.87036	-4110.38420
10.68	-4106.24811	-4106.67658	-4108.32304	-4108.79013	-4108.62194	-4109.09299	-4109.00123	-4109.45971	-4109.87037	-4110.38419
10.77	-4106.24808	-4106.67649	-4108.32299	-4108.79008	-4108.62191	-4109.09295	-4109.00122	-4109.45969	-4109.87038	-4110.38418
10.87	-4106.24805	-4106.67641	-4108.32294	-4108.79002	-4108.62189	-4109.09292	-4109.00121	-4109.45966	-4109.87038	-4110.38417
10.96	-4106.24802	-4106.67633	-4108.32290	-4108.78997	-4108.62187	-4109.09288	-4109.00120	-4109.45965	-4109.87039	-4110.38416
11.05	-4106.24800	-4106.67626	-4108.32286	-4108.78992	-4108.62184	-4109.09285	-4109.00119	-4109.45963	-4109.87040	-4110.38415
11.15	-4106.24798	-4106.67619	-4108.32282	-4108.78987	-4108.62183	-4109.09282	-4109.00118	-4109.45961	-4109.87040	-4110.38415
11.24	-4106.24795	-4106.67612	-4108.32278	-4108.78982	-4108.62181	-4109.09279	-4109.00117	-4109.45959	-4109.87041	-4110.38414
11.34	-4106.24793	-4106.67606	-4108.32275	-4108.78978	-4108.62179	-4109.09277	-4109.00116	-4109.45958	-4109.87041	-4110.38413
11.43	-4106.24791	-4106.67600	-4108.32272	-4108.78974	-4108.62178	-4109.09274	-4109.00116	-4109.45957	-4109.87042	-4110.38412
11.53	-4106.24790	-4106.67595	-4108.32269	-4108.78971	-4108.62177	-4109.09273	-4109.00116	-4109.45956	-4109.87042	-4110.38412
11.62	-4106.24788	-4106.67590	-4108.32266	-4108.78968	-4108.62175	-4109.09271	-4109.00115	-4109.45955	-4109.87043	-4110.38411
11.72	-4106.24786	-4106.67585	-4108.32264	-4108.78966	-4108.62175	-4109.09270	-4109.00116	-4109.45955	-4109.87043	-4110.38410
11.81	-4106.24785	-4106.67580	-4108.32263	-4108.78964	-4108.62174	-4109.09269	-4109.00116	-4109.45955	-4109.87044	-4110.38410
11.91	-4106.24784	-4106.67575	-4108.32261	-4108.78962	-4108.62174	-4109.09268	-4109.00117	-4109.45955	-4109.87046	-4110.38410
12.00	-4106.24782	-4106.67571	-4108.32260	-4108.78960	-4108.62174	-4109.09267	-4109.00118	-4109.45956	-4109.87047	-4110.38410
12.09	-4106.24781	-4106.67567	-4108.32259	-4108.78959	-4108.62174	-4109.09267	-4109.00118	-4109.45956	-4109.87047	-4110.38410
12.19	-4106.24780	-4106.67563	-4108.32258	-4108.78957	-4108.62174	-4109.09266	-4109.00119	-4109.45956	-4109.87048	-4110.38410
12.28	-4106.24779	-4106.67560	-4108.32256	-4108.78956	-4108.62173	-4109.09265	-4109.00120	-4109.45957	-4109.87049	-4110.38410
12.38	-4106.24778	-4106.67556	-4108.32255	-4108.78954	-4108.62173	-4109.09264	-4109.00120	-4109.45957	-4109.87049	-4110.38410
12.47	-4106.24777	-4106.67553	-4108.32254	-4108.78953	-4108.62173	-4109.09263	-4109.00121	-4109.45957	-4109.87050	-4110.38409
12.57	-4106.24776	-4106.67550	-4108.32253	-4108.78951	-4108.62173	-4109.09262	-4109.00121	-4109.45957	-4109.87050	-4110.38409
12.66	-4106.24775	-4106.67547	-4108.32252	-4108.78950	-4108.62172	-4109.09261	-4109.00122	-4109.45957	-4109.87050	-4110.38408
12.76	-4106.24775	-4106.67544	-4108.32251	-4108.78949	-4108.62172	-4109.09260	-4109.00122	-4109.45957	-4109.87050	-4110.38407
12.85	-4106.24774	-4106.67541	-4108.32250	-4108.78947	-4108.62171	-4109.09260	-4109.00123	-4109.45957	-4109.87050	-4110.38407
12.94	-4106.24773	-4106.67538	-4108.32250	-4108.78946	-4108.62171	-4109.09259	-4109.00123	-4109.45958	-4109.87050	-4110.38406
13.04	-4106.24772	-4106.67536	-4108.32249	-4108.78946	-4108.62171	-4109.09259	-4109.00124	-4109.45958	-4109.87050	-4110.38406
13.13	-4106.24772	-4106.67533	-4108.32248	-4108.78945	-4108.62171	-4109.09258	-4109.00125	-4109.45958	-4109.87051	-4110.38406
13.23	-4106.24771	-4106.67531	-4108.32248	-4108.78944	-4108.62171	-4109.09258	-4109.00125	-4109.45959	-4109.87051	-4110.38406
13.32	-4106.24771	-4106.67529	-4108.32248	-4108.78944	-4108.62171	-4109.09258	-4109.00126	-4109.45960	-4109.87051	-4110.38406
13.42	-4106.24770	-4106.67527	-4108.32247	-4108.78944	-4108.62171	-4109.09258	-4109.00127	-4109.45960	-4109.87052	-4110.38406
13.51	-4106.24770	-4106.67525	-4108.32247	-4108.78944	-4108.62171	-4109.09258	-4109.00128	-4109.45961	-4109.87052	-4110.38406
13.61	-4106.24769	-4106.67523	-4108.32247	-4108.78943	-4108.62171	-4109.09258	-4109.00129	-4109.45962	-4109.87053	-4110.38406
13.70	-4106.24769	-4106.67521	-4108.32247	-4108.78943	-4108.62172	-4109.09258	-4109.00130	-4109.45962	-4109.87053	-4110.38406
13.80	-4106.24768	-4106.67519	-4108.32247	-4108.78943	-4108.62172	-4109.09258	-4109.00130	-4109.45963	-4109.87053	-4110.38406
13.89	-4106.24768	-4106.67517	-4108.32247	-4108.78942	-4108.62172	-4109.09258	-4109.00131	-4109.45963	-4109.87053	-4110.38406
13.98	-4106.24767	-4106.67516	-4108.32246	-4108.78942	-4108.62172	-4109.09257	-4109.00131	-4109.45964	-4109.87053	-4110.38405
14.08	-4106.24767	-4106.67514	-4108.32246	-4108.78942	-4108.62172	-4109.09257	-4109.00132	-4109.45964	-4109.87053	-4110.38405

Table S13: The absolute energies in hartree of the potential energy curve of NbSi in the $^6\Sigma^+$ state with a CASSCF wave function and nom-CPO active space.

Distance	CASSCF	CASPT2	tpbe	ftpbe	trevpbe	ftrevpbe	tblyp	ftblyp	torelyp	ftorelyp
14.17	-4106.24767	-4106.67513	-4108.32246	-4108.78941	-4108.62171	-4109.09257	-4109.00132	-4109.45964	-4109.87053	-4110.38405
14.27	-4106.24766	-4106.67511	-4108.32246	-4108.78941	-4108.62171	-4109.09256	-4109.00133	-4109.45964	-4109.87053	-4110.38404
14.36	-4106.24766	-4106.67510	-4108.32245	-4108.78940	-4108.62171	-4109.09256	-4109.00133	-4109.45965	-4109.87053	-4110.38404
14.46	-4106.24765	-4106.67509	-4108.32245	-4108.78940	-4108.62171	-4109.09256	-4109.00133	-4109.45965	-4109.87053	-4110.38404
14.55	-4106.24765	-4106.67507	-4108.32245	-4108.78940	-4108.62171	-4109.09256	-4109.00134	-4109.45965	-4109.87053	-4110.38404
14.65	-4106.24765	-4106.67506	-4108.32245	-4108.78940	-4108.62171	-4109.09256	-4109.00134	-4109.45966	-4109.87053	-4110.38404
14.74	-4106.24765	-4106.67505	-4108.32244	-4108.78940	-4108.62171	-4109.09256	-4109.00135	-4109.45966	-4109.87053	-4110.38403
14.83	-4106.24764	-4106.67504	-4108.32244	-4108.78939	-4108.62171	-4109.09255	-4109.00135	-4109.45966	-4109.87053	-4110.38403
14.93	-4106.24764	-4106.67503	-4108.32244	-4108.78939	-4108.62171	-4109.09255	-4109.00135	-4109.45967	-4109.87053	-4110.38403
15.02	-4106.24764	-4106.67502	-4108.32244	-4108.78939	-4108.62171	-4109.09255	-4109.00136	-4109.45967	-4109.87053	-4110.38404
15.12	-4106.24764	-4106.67501	-4108.32244	-4108.78939	-4108.62171	-4109.09256	-4109.00136	-4109.45967	-4109.87053	-4110.38404
15.21	-4106.24763	-4106.67500	-4108.32244	-4108.78939	-4108.62171	-4109.09256	-4109.00137	-4109.45968	-4109.87053	-4110.38404
15.31	-4106.24763	-4106.67499	-4108.32244	-4108.78939	-4108.62171	-4109.09256	-4109.00137	-4109.45968	-4109.87053	-4110.38404
15.40	-4106.24763	-4106.67498	-4108.32245	-4108.78939	-4108.62171	-4109.09256	-4109.00138	-4109.45969	-4109.87053	-4110.38404
15.50	-4106.24763	-4106.67497	-4108.32245	-4108.78939	-4108.62171	-4109.09256	-4109.00138	-4109.45969	-4109.87054	-4110.38404
15.59	-4106.24762	-4106.67496	-4108.32245	-4108.78939	-4108.62171	-4109.09256	-4109.00138	-4109.45969	-4109.87054	-4110.38403
15.68	-4106.24762	-4106.67496	-4108.32245	-4108.78939	-4108.62171	-4109.09256	-4109.00139	-4109.45969	-4109.87054	-4110.38403
15.78	-4106.24762	-4106.67495	-4108.32244	-4108.78939	-4108.62171	-4109.09255	-4109.00139	-4109.45969	-4109.87054	-4110.38403
15.87	-4106.24762	-4106.67494	-4108.32244	-4108.78939	-4108.62171	-4109.09255	-4109.00139	-4109.45970	-4109.87054	-4110.38403
15.97	-4106.24762	-4106.67493	-4108.32244	-4108.78939	-4108.62171	-4109.09255	-4109.00139	-4109.45970	-4109.87053	-4110.38403
16.06	-4106.24762	-4106.67492	-4108.32244	-4108.78939	-4108.62171	-4109.09255	-4109.00139	-4109.45970	-4109.87053	-4110.38403
16.16	-4106.24761	-4106.67492	-4108.32244	-4108.78939	-4108.62171	-4109.09255	-4109.00140	-4109.45970	-4109.87053	-4110.38403
16.25	-4106.24761	-4106.67491	-4108.32244	-4108.78939	-4108.62171	-4109.09255	-4109.00140	-4109.45970	-4109.87053	-4110.38403
16.35	-4106.24761	-4106.67491	-4108.32244	-4108.78939	-4108.62171	-4109.09255	-4109.00140	-4109.45970	-4109.87053	-4110.38403
16.44	-4106.24761	-4106.67490	-4108.32244	-4108.78939	-4108.62171	-4109.09255	-4109.00140	-4109.45970	-4109.87053	-4110.38403
16.54	-4106.24761	-4106.67490	-4108.32244	-4108.78938	-4108.62171	-4109.09255	-4109.00140	-4109.45970	-4109.87053	-4110.38402
16.63	-4106.24761	-4106.67489	-4108.32244	-4108.78938	-4108.62171	-4109.09255	-4109.00140	-4109.45970	-4109.87053	-4110.38402
16.72	-4106.24761	-4106.67489	-4108.32244	-4108.78938	-4108.62171	-4109.09254	-4109.00140	-4109.45970	-4109.87053	-4110.38402
16.82	-4106.24760	-4106.67488	-4108.32244	-4108.78938	-4108.62171	-4109.09254	-4109.00140	-4109.45971	-4109.87053	-4110.38402
16.91	-4106.24760	-4106.67488	-4108.32244	-4108.78938	-4108.62171	-4109.09254	-4109.00140	-4109.45971	-4109.87053	-4110.38402
17.01	-4106.24760	-4106.67487	-4108.32244	-4108.78938	-4108.62171	-4109.09254	-4109.00141	-4109.45971	-4109.87053	-4110.38402
17.10	-4106.24760	-4106.67487	-4108.32244	-4108.78938	-4108.62171	-4109.09255	-4109.00141	-4109.45971	-4109.87053	-4110.38402
17.20	-4106.24760	-4106.67487	-4108.32244	-4108.78938	-4108.62171	-4109.09255	-4109.00141	-4109.45971	-4109.87053	-4110.38402
17.29	-4106.24760	-4106.67486	-4108.32244	-4108.78938	-4108.62171	-4109.09255	-4109.00141	-4109.45971	-4109.87053	-4110.38402
17.39	-4106.24760	-4106.67486	-4108.32244	-4108.78938	-4108.62171	-4109.09255	-4109.00141	-4109.45971	-4109.87053	-4110.38402
17.48	-4106.24760	-4106.67486	-4108.32244	-4108.78938	-4108.62171	-4109.09255	-4109.00141	-4109.45972	-4109.87053	-4110.38402
17.57	-4106.24760	-4106.67485	-4108.32244	-4108.78938	-4108.62171	-4109.09255	-4109.00142	-4109.45972	-4109.87053	-4110.38402
17.67	-4106.24760	-4106.67485	-4108.32244	-4108.78938	-4108.62171	-4109.09255	-4109.00142	-4109.45972	-4109.87053	-4110.38402
17.76	-4106.24760	-4106.67485	-4108.32244	-4108.78938	-4108.62171	-4109.09255	-4109.00142	-4109.45972	-4109.87053	-4110.38402
17.86	-4106.24759	-4106.67484	-4108.32244	-4108.78938	-4108.62171	-4109.09255	-4109.00142	-4109.45972	-4109.87053	-4110.38402
17.95	-4106.24759	-4106.67484	-4108.32244	-4108.78938	-4108.62171	-4109.09255	-4109.00142	-4109.45972	-4109.87053	-4110.38402
18.05	-4106.24759	-4106.67484	-4108.32244	-4108.78938	-4108.62171	-4109.09255	-4109.00142	-4109.45972	-4109.87053	-4110.38402

Table S14: The absolute energies in hartree of the potential energy curve of NbSi in the $^2\Sigma^+$ state with a CASSCF wave function and nom-CPO active space.

Distance	CASSCF	CASPT2	tpbe	ftpbe	trevpbe	ftrevpbe	tblyp	ftblyp	torelyp	ftorelyp
1.98	-4102.90780	-4103.38580	-4105.09589	-4105.56728	-4105.37836	-4105.85046	-4105.73339	-4106.20238	-4106.68025	-4107.19645
2.08	-4103.56272	-4104.03926	-4105.74164	-4106.21259	-4106.02503	-4106.49678	-4106.38109	-4106.84941	-4107.32149	-4107.83733
2.17	-4104.08849	-4104.56391	-4106.25899	-4106.72960	-4106.54321	-4107.01476	-4106.90020	-4107.36787	-4107.83452	-4108.35020
2.27	-4104.50978	-4104.98577	-4106.67342	-4107.14391	-4106.95823	-4107.42993	-4107.31626	-4107.78360	-4108.24453	-4108.76060

Table S14: The absolute energies in hartree of the potential energy curve of NbSi in the $^2\Sigma^+$ state with a CASSCF wave function and nom-CPO active space.

Distance	CASSCF	CASPT2	tpbe	ftpbe	trevpbe	ftrevpbe	tblyp	ftblyp	torelyp	ftorelyp
2.36	-4104.84989	-4105.33076	-4107.00927	-4107.48010	-4107.29414	-4107.76667	-4107.65413	-4108.12216	-4108.57511	-4109.09278
2.46	-4105.12878	-4105.60975	-4107.28300	-4107.75289	-4107.56852	-4108.03979	-4107.93009	-4108.39728	-4108.84613	-4109.36230
2.55	-4105.36279	-4105.82707	-4107.49393	-4107.96193	-4107.78062	-4108.24870	-4108.14292	-4108.60982	-4109.05720	-4109.56924
2.65	-4105.54784	-4106.01061	-4107.67528	-4108.14334	-4107.96286	-4108.43108	-4108.32443	-4108.79121	-4109.23711	-4109.74929
2.74	-4105.69758	-4106.15839	-4107.82170	-4108.28999	-4108.11016	-4108.57870	-4108.47109	-4108.93793	-4109.38239	-4109.89490
2.83	-4105.82033	-4106.27886	-4107.94194	-4108.41227	-4108.23144	-4108.70244	-4108.59060	-4109.05869	-4109.50169	-4110.01661
2.93	-4105.92181	-4106.37643	-4108.03902	-4108.50955	-4108.32940	-4108.80073	-4108.68792	-4109.15590	-4109.59810	-4110.11326
3.02	-4106.00495	-4106.45700	-4108.11922	-4108.58970	-4108.41037	-4108.88173	-4108.76852	-4109.23622	-4109.67758	-4110.19273
3.12	-4106.07282	-4106.52344	-4108.18546	-4108.65578	-4108.47728	-4108.94858	-4108.83519	-4109.30253	-4109.74311	-4110.25817
3.21	-4106.12801	-4106.57807	-4108.24002	-4108.71014	-4108.53245	-4109.00362	-4108.89019	-4109.35712	-4109.79699	-4110.31192
3.31	-4106.17267	-4106.62283	-4108.28481	-4108.75467	-4108.57781	-4109.04879	-4108.93541	-4109.40189	-4109.84119	-4110.35590
3.40	-4106.20863	-4106.65938	-4108.32151	-4108.79100	-4108.61502	-4109.08571	-4108.97253	-4109.43848	-4109.87738	-4110.39174
3.50	-4106.23745	-4106.68910	-4108.35150	-4108.82047	-4108.64551	-4109.11573	-4109.00298	-4109.46828	-4109.90703	-4110.42081
3.59	-4106.26046	-4106.71301	-4108.37589	-4108.84416	-4108.67036	-4109.13990	-4109.02788	-4109.49241	-4109.93126	-4110.44417
3.68	-4106.27230	-4106.73181	-4108.39529	-4108.86274	-4108.69020	-4109.15890	-4109.04792	-4109.51162	-4109.95070	-4110.46253
3.78	-4106.28871	-4106.74598	-4108.41043	-4108.87695	-4108.70576	-4109.17348	-4109.06386	-4109.52680	-4109.96623	-4110.47678
3.87	-4106.30155	-4106.75653	-4108.42180	-4108.88804	-4108.71754	-4109.18500	-4109.07594	-4109.53855	-4109.97757	-4110.48771
3.97	-4106.30953	-4106.76428	-4108.43045	-4108.89675	-4108.72654	-4109.19416	-4109.08536	-4109.54794	-4109.98594	-4110.49614
4.06	-4106.31862	-4106.76968	-4108.43663	-4108.90324	-4108.73296	-4109.20104	-4109.09237	-4109.55511	-4109.99148	-4110.50216
4.16	-4106.31782	-4106.77316	-4108.44070	-4108.90758	-4108.73718	-4109.20568	-4109.09728	-4109.56011	-4109.99476	-4110.50594
4.35	-4106.32877	-4106.77506	-4108.44315	-4108.91018	-4108.73971	-4109.20850	-4109.10055	-4109.56334	-4109.99634	-4110.50793
4.44	-4106.32934	-4106.77562	-4108.44423	-4108.91133	-4108.74082	-4109.20984	-4109.10247	-4109.56512	-4109.99647	-4110.50840
4.54	-4106.32894	-4106.77504	-4108.44414	-4108.91127	-4108.74069	-4109.20991	-4109.10325	-4109.56568	-4109.99520	-4110.50745
4.63	-4106.32778	-4106.77322	-4108.44257	-4108.90973	-4108.73889	-4109.20838	-4109.10273	-4109.56485	-4109.99167	-4110.50435
4.72	-4106.32606	-4106.76951	-4108.43827	-4108.90554	-4108.73417	-4109.20412	-4109.09954	-4109.56132	-4109.98459	-4110.49774
4.82	-4106.32392	-4106.76552	-4108.43362	-4108.90106	-4108.72929	-4109.19973	-4109.09561	-4109.55714	-4109.97808	-4110.49161
4.91	-4106.32149	-4106.76140	-4108.42874	-4108.89642	-4108.72426	-4109.19528	-4109.09126	-4109.55262	-4109.97175	-4110.48575
5.01	-4106.31888	-4106.75699	-4108.42330	-4108.89128	-4108.71872	-4109.19041	-4109.08624	-4109.54746	-4109.96506	-4110.47963
5.10	-4106.31615	-4106.75236	-4108.41771	-4108.88596	-4108.71309	-4109.18537	-4109.08098	-4109.54205	-4109.95844	-4110.47356
5.20	-4106.31338	-4106.74765	-4108.41223	-4108.88068	-4108.70760	-4109.18038	-4109.07579	-4109.53671	-4109.95215	-4110.46772
5.29	-4106.31058	-4106.74294	-4108.40692	-4108.87553	-4108.70231	-4109.17550	-4109.07075	-4109.53152	-4109.94621	-4110.46214
5.39	-4106.30778	-4106.73830	-4108.40182	-4108.87056	-4108.69727	-4109.17078	-4109.06592	-4109.52656	-4109.94064	-4110.45687
5.48	-4106.30498	-4106.73376	-4108.39695	-4108.86578	-4108.69247	-4109.16624	-4109.06132	-4109.52183	-4109.93542	-4110.45189
5.57	-4106.30218	-4106.72933	-4108.39230	-4108.86120	-4108.68791	-4109.16187	-4109.05695	-4109.51733	-4109.93053	-4110.44718
5.67	-4106.29938	-4106.72502	-4108.38786	-4108.85680	-4108.68356	-4109.15768	-4109.05280	-4109.51306	-4109.92594	-4110.44273
5.76	-4106.29660	-4106.72083	-4108.38362	-4108.85260	-4108.67942	-4109.15365	-4109.04886	-4109.50900	-4109.92161	-4110.43852
5.86	-4106.29384	-4106.71676	-4108.37958	-4108.84856	-4108.67547	-4109.14979	-4109.04511	-4109.50514	-4109.91754	-4110.43453
5.95	-4106.29112	-4106.71282	-4108.37572	-4108.84471	-4108.67171	-4109.14609	-4109.04156	-4109.50147	-4109.91369	-4110.43074
6.05	-4106.28843	-4106.70900	-4108.37204	-4108.84102	-4108.66813	-4109.14255	-4109.03819	-4109.49800	-4109.91006	-4110.42716
6.14	-4106.28578	-4106.70531	-4108.36852	-4108.83749	-4108.66470	-4109.13914	-4109.03499	-4109.49470	-4109.90662	-4110.42376
6.24	-4106.28320	-4106.70175	-4108.36517	-4108.83411	-4108.66144	-4109.13589	-4109.03197	-4109.49157	-4109.90338	-4110.42055
6.33	-4106.28068	-4106.69832	-4108.36198	-4108.83090	-4108.65834	-4109.13278	-4109.02912	-4109.48863	-4109.90032	-4110.41751
6.43	-4106.27823	-4106.69504	-4108.35895	-4108.82785	-4108.65540	-4109.12983	-4109.02644	-4109.48587	-4109.89744	-4110.41466
6.52	-4106.27586	-4106.69190	-4108.35608	-4108.82493	-4108.65261	-4109.12699	-4109.02392	-4109.48327	-4109.89473	-4110.41197
6.61	-4106.27359	-4106.68892	-4108.35335	-4108.82217	-4108.64996	-4109.12429	-4109.02156	-4109.48084	-4109.89220	-4110.40944
6.71	-4106.27141	-4106.68609	-4108.35079	-4108.81957	-4108.64746	-4109.12175	-4109.01938	-4109.47859	-4109.88983	-4110.40710
6.80	-4106.26934	-4106.68341	-4108.34836	-4108.81711	-4108.64511	-4109.11933	-4109.01735	-4109.47652	-4109.88763	-4110.40491
6.90	-4106.26739	-4106.68091	-4108.34608	-4108.81477	-4108.64290	-4109.11703	-4109.01549	-4109.47460	-4109.88559	-4110.40287
6.99	-4106.26556	-4106.67857	-4108.34393	-4108.81256	-4108.64081	-4109.11484	-4109.01377	-4109.47284	-4109.88369	-4110.40097
7.09	-4106.26386	-4106.67639	-4108.34189	-4108.81048	-4108.63883	-4109.11277	-4109.01219	-4109.47124	-4109.88193	-4110.39921
7.18	-4106.26228	-4106.67439	-4108.33997	-4108.80849	-4108.63697	-4109.11078	-4109.01075	-4109.46977	-4109.88031	-4110.39754
7.28	-4106.26084	-4106.67255	-4108.33815	-4108.80658	-4108.63520	-4109.10886	-4109.00941	-4109.46841	-4109.87880	-4110.39597

Table S14: The absolute energies in hartree of the potential energy curve of NbSi in the $^2\Sigma^+$ state with a CASSCF wave function and nom-CPO active space.

Distance	CASSCF	CASPT2	tpbe	ftpbe	trevpbe	ftrevpbe	tblyp	ftblyp	torelyp	ftorelyp
7.37	-4106.25951	-4106.67088	-4108.33641	-4108.80477	-4108.63353	-4109.10702	-4109.00819	-4109.46718	-4109.87739	-4110.39449
7.46	-4106.25831	-4106.66937	-4108.33475	-4108.80303	-4108.63192	-4109.10525	-4109.00704	-4109.46603	-4109.87608	-4110.39307
7.56	-4106.25723	-4106.66800	-4108.33314	-4108.80133	-4108.63037	-4109.10352	-4109.00596	-4109.46494	-4109.87483	-4110.39168
7.65	-4106.25625	-4106.66678	-4108.33159	-4108.79969	-4108.62888	-4109.10183	-4109.00493	-4109.46392	-4109.87365	-4110.39033
7.75	-4106.25537	-4106.66569	-4108.33009	-4108.79810	-4108.62744	-4109.10020	-4109.00395	-4109.46295	-4109.87252	-4110.38902
7.84	-4106.25459	-4106.66473	-4108.32865	-4108.79658	-4108.62606	-4109.09864	-4109.00300	-4109.46203	-4109.87146	-4110.38776
7.94	-4106.25388	-4106.66387	-4108.32727	-4108.79513	-4108.62475	-4109.09715	-4109.00211	-4109.46116	-4109.87046	-4110.38655
8.03	-4106.25325	-4106.66312	-4108.32597	-4108.79376	-4108.62351	-4109.09575	-4109.00126	-4109.46034	-4109.86954	-4110.38541
8.13	-4106.25269	-4106.66246	-4108.32475	-4108.79248	-4108.62236	-4109.09444	-4109.00047	-4109.45958	-4109.86869	-4110.38435
8.22	-4106.25219	-4106.66187	-4108.32363	-4108.79132	-4108.62131	-4109.09326	-4108.99974	-4109.45889	-4109.86793	-4110.38340
8.31	-4106.25174	-4106.66136	-4108.32261	-4108.79028	-4108.62037	-4109.09221	-4108.99908	-4109.45828	-4109.86727	-4110.38255
8.41	-4106.25133	-4106.66089	-4108.32171	-4108.78935	-4108.61954	-4109.09128	-4108.99850	-4109.45773	-4109.86670	-4110.38182
8.50	-4106.25097	-4106.66047	-4108.32091	-4108.78853	-4108.61881	-4109.09047	-4108.99799	-4109.45725	-4109.86621	-4110.38119
8.60	-4106.25065	-4106.66008	-4108.32021	-4108.78781	-4108.61818	-4109.08977	-4108.99754	-4109.45683	-4109.86580	-4110.38066
8.69	-4106.25037	-4106.65973	-4108.31960	-4108.78719	-4108.61763	-4109.08918	-4108.99715	-4109.45647	-4109.86546	-4110.38022
8.79	-4106.25011	-4106.65940	-4108.31907	-4108.78665	-4108.61717	-4109.08867	-4108.99682	-4109.45615	-4109.86517	-4110.37984
8.88	-4106.24989	-4106.65909	-4108.31860	-4108.78617	-4108.61676	-4109.08822	-4108.99652	-4109.45587	-4109.86493	-4110.37953
8.98	-4106.24968	-4106.65880	-4108.31818	-4108.78575	-4108.61640	-4109.08784	-4108.99626	-4109.45562	-4109.86473	-4110.37927
9.07	-4106.24950	-4106.65853	-4108.31782	-4108.78538	-4108.61610	-4109.08751	-4108.99603	-4109.45539	-4109.86456	-4110.37905
9.17	-4106.24934	-4106.65828	-4108.31749	-4108.78504	-4108.61583	-4109.08721	-4108.99582	-4109.45519	-4109.86442	-4110.37886
9.26	-4106.24919	-4106.65805	-4108.31721	-4108.78475	-4108.61560	-4109.08696	-4108.99566	-4109.45503	-4109.86431	-4110.37871
9.35	-4106.24906	-4106.65783	-4108.31696	-4108.78450	-4108.61541	-4109.08675	-4108.99551	-4109.45488	-4109.86423	-4110.37859
9.45	-4106.24895	-4106.65763	-4108.31674	-4108.78427	-4108.61523	-4109.08656	-4108.99538	-4109.45475	-4109.86415	-4110.37848
9.54	-4106.24884	-4106.65744	-4108.31655	-4108.78406	-4108.61508	-4109.08639	-4108.99527	-4109.45463	-4109.86410	-4110.37839
9.64	-4106.24874	-4106.65726	-4108.31637	-4108.78388	-4108.61495	-4109.08625	-4108.99517	-4109.45453	-4109.86405	-4110.37832
9.73	-4106.24865	-4106.65709	-4108.31621	-4108.78371	-4108.61484	-4109.08612	-4108.99508	-4109.45443	-4109.86402	-4110.37826
9.83	-4106.24857	-4106.65692	-4108.31606	-4108.78355	-4108.61473	-4109.08599	-4108.99500	-4109.45434	-4109.86398	-4110.37820
9.92	-4106.24850	-4106.65678	-4108.31594	-4108.78342	-4108.61465	-4109.08590	-4108.99494	-4109.45428	-4109.86397	-4110.37817
10.02	-4106.24844	-4106.65664	-4108.31583	-4108.78330	-4108.61457	-4109.08581	-4108.99489	-4109.45421	-4109.86396	-4110.37813
10.11	-4106.24837	-4106.65651	-4108.31573	-4108.78319	-4108.61451	-4109.08573	-4108.99484	-4109.45416	-4109.86395	-4110.37811
10.20	-4106.24832	-4106.65639	-4108.31564	-4108.78309	-4108.61445	-4109.08566	-4108.99480	-4109.45411	-4109.86395	-4110.37809
10.30	-4106.24827	-4106.65628	-4108.31556	-4108.78300	-4108.61441	-4109.08560	-4108.99477	-4109.45407	-4109.86396	-4110.37807
10.39	-4106.24822	-4106.65617	-4108.31549	-4108.78292	-4108.61437	-4109.08555	-4108.99474	-4109.45403	-4109.86397	-4110.37806
10.49	-4106.24818	-4106.65606	-4108.31542	-4108.78285	-4108.61433	-4109.08550	-4108.99471	-4109.45400	-4109.86397	-4110.37804
10.58	-4106.24814	-4106.65597	-4108.31536	-4108.78278	-4108.61429	-4109.08545	-4108.99469	-4109.45397	-4109.86398	-4110.37804
10.68	-4106.24810	-4106.65587	-4108.31531	-4108.78272	-4108.61427	-4109.08542	-4108.99468	-4109.45395	-4109.86399	-4110.37803
10.77	-4106.24807	-4106.65579	-4108.31526	-4108.78266	-4108.61424	-4109.08538	-4108.99467	-4109.45393	-4109.86400	-4110.37803
10.87	-4106.24804	-4106.65571	-4108.31522	-4108.78261	-4108.61422	-4109.08535	-4108.99466	-4109.45391	-4109.86401	-4110.37802
10.96	-4106.24801	-4106.65563	-4108.31518	-4108.78257	-4108.61421	-4109.08533	-4108.99465	-4109.45390	-4109.86403	-4110.37802
11.05	-4106.24799	-4106.65556	-4108.31515	-4108.78253	-4108.61420	-4109.08531	-4108.99465	-4109.45389	-4109.86404	-4110.37802
11.15	-4106.24796	-4106.65549	-4108.31511	-4108.78249	-4108.61418	-4109.08528	-4108.99464	-4109.45388	-4109.86406	-4110.37802
11.24	-4106.24794	-4106.65542	-4108.31509	-4108.78246	-4108.61417	-4109.08527	-4108.99465	-4109.45387	-4109.86407	-4110.37802
11.34	-4106.24792	-4106.65536	-4108.31506	-4108.78243	-4108.61416	-4109.08525	-4108.99465	-4109.45387	-4109.86408	-4110.37802
11.43	-4106.24790	-4106.65530	-4108.31504	-4108.78239	-4108.61416	-4109.08523	-4108.99465	-4109.45386	-4109.86409	-4110.37802
11.53	-4106.24788	-4106.65525	-4108.31502	-4108.78237	-4108.61415	-4109.08522	-4108.99465	-4109.45386	-4109.86411	-4110.37802
11.62	-4106.24787	-4106.65520	-4108.31500	-4108.78235	-4108.61415	-4109.08521	-4108.99466	-4109.45386	-4109.86412	-4110.37802
11.72	-4106.24785	-4106.65515	-4108.31498	-4108.78232	-4108.61414	-4109.08519	-4108.99466	-4109.45386	-4109.86412	-4110.37802
11.81	-4106.24784	-4106.65510	-4108.31496	-4108.78230	-4108.61413	-4109.08519	-4108.99467	-4109.45386	-4109.86413	-4110.37802
11.91	-4106.24782	-4106.65506	-4108.31495	-4108.78229	-4108.61414	-4109.08518	-4108.99468	-4109.45387	-4109.86415	-4110.37803
12.00	-4106.24781	-4106.65502	-4108.31494	-4108.78227	-4108.61413	-4109.08517	-4108.99468	-4109.45387	-4109.86415	-4110.37802
12.09	-4106.24780	-4106.65498	-4108.31493	-4108.78226	-4108.61413	-4109.08517	-4108.99469	-4109.45387	-4109.86416	-4110.37802
12.19	-4106.24779	-4106.65494	-4108.31492	-4108.78224	-4108.61413	-4109.08516	-4108.99470	-4109.45388	-4109.86418	-4110.37803

Table S14: The absolute energies in hartree of the potential energy curve of NbSi in the $^2\Sigma^+$ state with a CASSCF wave function and nom-CPO active space.

Distance	CASSCF	CASPT2	tpbe	ftpbe	trevpbe	ftrevpbe	tblyp	ftblyp	torelyp	ftorelyp
12.28	-4106.24778	-4106.65491	-4108.31491	-4108.78223	-4108.61413	-4109.08515	-4108.99471	-4109.45388	-4109.86418	-4110.37803
12.38	-4106.24777	-4106.65487	-4108.31489	-4108.78221	-4108.61412	-4109.08514	-4108.99471	-4109.45388	-4109.86418	-4110.37801
12.47	-4106.24776	-4106.65485	-4108.31490	-4108.78221	-4108.61413	-4109.08515	-4108.99473	-4109.45390	-4109.86420	-4110.37803
12.57	-4106.24775	-4106.65481	-4108.31488	-4108.78220	-4108.61413	-4109.08514	-4108.99473	-4109.45390	-4109.86420	-4110.37803
12.66	-4106.24774	-4106.65479	-4108.31488	-4108.78219	-4108.61413	-4109.08513	-4108.99474	-4109.45390	-4109.86421	-4110.37802
12.76	-4106.24773	-4106.65476	-4108.31487	-4108.78218	-4108.61413	-4109.08513	-4108.99475	-4109.45391	-4109.86421	-4110.37802
12.85	-4106.24773	-4106.65473	-4108.31486	-4108.78217	-4108.61412	-4109.08512	-4108.99475	-4109.45391	-4109.86421	-4110.37802
12.94	-4106.24772	-4106.65471	-4108.31486	-4108.78217	-4108.61412	-4109.08512	-4108.99476	-4109.45392	-4109.86422	-4110.37802
13.04	-4106.24771	-4106.65468	-4108.31485	-4108.78216	-4108.61412	-4109.08512	-4108.99477	-4109.45392	-4109.86422	-4110.37802
13.13	-4106.24771	-4106.65466	-4108.31485	-4108.78215	-4108.61412	-4109.08511	-4108.99477	-4109.45392	-4109.86422	-4110.37801
13.23	-4106.24770	-4106.65463	-4108.31485	-4108.78215	-4108.61412	-4109.08511	-4108.99479	-4109.45394	-4109.86423	-4110.37802
13.32	-4106.24770	-4106.65461	-4108.31484	-4108.78215	-4108.61412	-4109.08511	-4108.99479	-4109.45394	-4109.86423	-4110.37802
13.42	-4106.24769	-4106.65459	-4108.31484	-4108.78214	-4108.61413	-4109.08511	-4108.99480	-4109.45395	-4109.86424	-4110.37802
13.51	-4106.24769	-4106.65457	-4108.31484	-4108.78214	-4108.61413	-4109.08511	-4108.99481	-4109.45396	-4109.86424	-4110.37802
13.61	-4106.24768	-4106.65455	-4108.31484	-4108.78214	-4108.61413	-4109.08511	-4108.99482	-4109.45396	-4109.86424	-4110.37802
13.70	-4106.24768	-4106.65453	-4108.31483	-4108.78213	-4108.61413	-4109.08511	-4108.99482	-4109.45396	-4109.86424	-4110.37802
13.80	-4106.24767	-4106.65452	-4108.31483	-4108.78213	-4108.61413	-4109.08510	-4108.99483	-4109.45397	-4109.86424	-4110.37801
13.89	-4106.24767	-4106.65450	-4108.31483	-4108.78212	-4108.61412	-4109.08510	-4108.99483	-4109.45397	-4109.86425	-4110.37801
13.98	-4106.24766	-4106.65449	-4108.31483	-4108.78212	-4108.61412	-4109.08510	-4108.99484	-4109.45398	-4109.86425	-4110.37801
14.08	-4106.24766	-4106.65447	-4108.31483	-4108.78212	-4108.61412	-4109.08510	-4108.99484	-4109.45398	-4109.86425	-4110.37801
14.17	-4106.24766	-4106.65446	-4108.31482	-4108.78212	-4108.61412	-4109.08510	-4108.99485	-4109.45399	-4109.86425	-4110.37801
14.27	-4106.24765	-4106.65444	-4108.31482	-4108.78212	-4108.61412	-4109.08510	-4108.99485	-4109.45399	-4109.86425	-4110.37801
14.36	-4106.24765	-4106.65443	-4108.31482	-4108.78211	-4108.61412	-4109.08510	-4108.99486	-4109.45400	-4109.86425	-4110.37801
14.46	-4106.24765	-4106.65442	-4108.31482	-4108.78211	-4108.61412	-4109.08509	-4108.99486	-4109.45400	-4109.86425	-4110.37801
14.55	-4106.24764	-4106.65441	-4108.31482	-4108.78211	-4108.61412	-4109.08509	-4108.99487	-4109.45400	-4109.86425	-4110.37801
14.65	-4106.24764	-4106.65440	-4108.31482	-4108.78211	-4108.61412	-4109.08509	-4108.99487	-4109.45401	-4109.86425	-4110.37801
14.74	-4106.24764	-4106.65439	-4108.31482	-4108.78211	-4108.61412	-4109.08509	-4108.99488	-4109.45401	-4109.86425	-4110.37801
14.83	-4106.24764	-4106.65438	-4108.31482	-4108.78211	-4108.61413	-4109.08509	-4108.99488	-4109.45402	-4109.86426	-4110.37801
14.93	-4106.24763	-4106.65436	-4108.31482	-4108.78211	-4108.61413	-4109.08509	-4108.99489	-4109.45402	-4109.86426	-4110.37801
15.02	-4106.24763	-4106.65435	-4108.31482	-4108.78211	-4108.61413	-4109.08509	-4108.99489	-4109.45402	-4109.86426	-4110.37801
15.12	-4106.24763	-4106.65434	-4108.31482	-4108.78211	-4108.61413	-4109.08509	-4108.99489	-4109.45403	-4109.86426	-4110.37801
15.21	-4106.24763	-4106.65433	-4108.31482	-4108.78211	-4108.61413	-4109.08509	-4108.99490	-4109.45403	-4109.86426	-4110.37801
15.31	-4106.24763	-4106.65432	-4108.31482	-4108.78211	-4108.61413	-4109.08509	-4108.99490	-4109.45403	-4109.86426	-4110.37801
15.40	-4106.24762	-4106.65432	-4108.31482	-4108.78211	-4108.61413	-4109.08509	-4108.99490	-4109.45403	-4109.86426	-4110.37801
15.50	-4106.24762	-4106.65431	-4108.31482	-4108.78211	-4108.61413	-4109.08509	-4108.99491	-4109.45404	-4109.86426	-4110.37801
15.59	-4106.24762	-4106.65430	-4108.31482	-4108.78211	-4108.61413	-4109.08509	-4108.99491	-4109.45404	-4109.86426	-4110.37801
15.68	-4106.24762	-4106.65429	-4108.31482	-4108.78211	-4108.61413	-4109.08509	-4108.99492	-4109.45405	-4109.86426	-4110.37801
15.78	-4106.24762	-4106.65429	-4108.31482	-4108.78211	-4108.61413	-4109.08509	-4108.99492	-4109.45405	-4109.86426	-4110.37801
15.87	-4106.24761	-4106.65345	-4108.31482	-4108.78211	-4108.61413	-4109.08509	-4108.99492	-4109.45405	-4109.86426	-4110.37801
15.97	-4106.24761	-4106.65344	-4108.31482	-4108.78211	-4108.61413	-4109.08509	-4108.99492	-4109.45405	-4109.86426	-4110.37801
16.06	-4106.24761	-4106.65343	-4108.31482	-4108.78211	-4108.61413	-4109.08509	-4108.99493	-4109.45405	-4109.86426	-4110.37801
16.16	-4106.24761	-4106.65343	-4108.31482	-4108.78211	-4108.61413	-4109.08509	-4108.99493	-4109.45406	-4109.86427	-4110.37801
16.25	-4106.24761	-4106.65342	-4108.31482	-4108.78211	-4108.61413	-4109.08509	-4108.99493	-4109.45406	-4109.86427	-4110.37801
16.35	-4106.24761	-4106.65341	-4108.31482	-4108.78211	-4108.61413	-4109.08510	-4108.99494	-4109.45406	-4109.86427	-4110.37801
16.44	-4106.24761	-4106.65341	-4108.31482	-4108.78211	-4108.61413	-4109.08510	-4108.99494	-4109.45407	-4109.86427	-4110.37801
16.54	-4106.24761	-4106.65340	-4108.31482	-4108.78211	-4108.61414	-4109.08510	-4108.99494	-4109.45407	-4109.86427	-4110.37801
16.63	-4106.24760	-4106.65339	-4108.31482	-4108.78211	-4108.61414	-4109.08510	-4108.99494	-4109.45407	-4109.86427	-4110.37801
16.72	-4106.24760	-4106.65337	-4108.31482	-4108.78211	-4108.61414	-4109.08510	-4108.99494	-4109.45407	-4109.86427	-4110.37801
16.82	-4106.24760	-4106.65337	-4108.31482	-4108.78211	-4108.61413	-4109.08510	-4108.99495	-4109.45407	-4109.86427	-4110.37801
16.91	-4106.24760	-4106.65336	-4108.31482	-4108.78211	-4108.61413	-4109.08509	-4108.99495	-4109.45407	-4109.86427	-4110.37801
17.01	-4106.24760	-4106.65332	-4108.31482	-4108.78211	-4108.61413	-4109.08509	-4108.99495	-4109.45407	-4109.86427	-4110.37801
17.10	-4106.24760	-4106.65332	-4108.31482	-4108.78211	-4108.61413	-4109.08509	-4108.99495	-4109.45408	-4109.86427	-4110.37801

Table S14: The absolute energies in hartree of the potential energy curve of NbSi in the $^2\Sigma^+$ state with a CASSCF wave function and nom-CPO active space.

Distance	CASSCF	CASPT2	tpbe	ftpbe	trevpbe	ftrevpbe	tblyp	ftblyp	torelyp	ftorelyp
17.20	-4106.24760	-4106.65332	-4108.31482	-4108.78211	-4108.61413	-4109.08509	-4108.99495	-4109.45408	-4109.86427	-4110.37801
17.29	-4106.24760	-4106.65328	-4108.31482	-4108.78211	-4108.61413	-4109.08509	-4108.99495	-4109.45408	-4109.86427	-4110.37801
17.39	-4106.24760	-4106.65327	-4108.31482	-4108.78211	-4108.61413	-4109.08509	-4108.99495	-4109.45408	-4109.86427	-4110.37801
17.48	-4106.24760	-4106.65326	-4108.31482	-4108.78211	-4108.61413	-4109.08509	-4108.99495	-4109.45408	-4109.86427	-4110.37801
17.57	-4106.24759	-4106.65326	-4108.31482	-4108.78211	-4108.61414	-4109.08509	-4108.99496	-4109.45408	-4109.86427	-4110.37801
17.67	-4106.24759	-4106.65326	-4108.31482	-4108.78211	-4108.61414	-4109.08509	-4108.99496	-4109.45408	-4109.86427	-4110.37801
17.76	-4106.24759	-4106.65326	-4108.31483	-4108.78211	-4108.61414	-4109.08510	-4108.99496	-4109.45408	-4109.86427	-4110.37801
17.86	-4106.24759	-4106.65325	-4108.31483	-4108.78211	-4108.61414	-4109.08510	-4108.99496	-4109.45409	-4109.86427	-4110.37801
17.95	-4106.24759	-4106.65325	-4108.31483	-4108.78211	-4108.61414	-4109.08510	-4108.99496	-4109.45409	-4109.86427	-4110.37801
18.05	-4106.24759	-4106.65325	-4108.31483	-4108.78211	-4108.61414	-4109.08510	-4108.99496	-4109.45409	-4109.86427	-4110.37801

Table S15: The absolute energies in hartree of the potential energy curve of NbSi in the $^2\Delta$ state with a CASSCF wave function and nom-CPO active space.

Distance	CASSCF	CASPT2	tpbe	ftpbe	trevpbe	ftrevpbe	tblyp	ftblyp	torelyp	ftorelyp
1.98	-4102.92306	-4103.38518	-4105.09591	-4105.57091	-4105.37828	-4105.85467	-4105.72527	-4106.20429	-4106.67886	-4107.19991
2.08	-4103.57585	-4104.03820	-4105.74073	-4106.21532	-4106.02412	-4106.50011	-4106.37270	-4106.85073	-4107.31951	-4107.84014
2.17	-4104.08849	-4104.56087	-4106.25625	-4106.73044	-4106.54067	-4107.01625	-4106.89044	-4107.36768	-4107.83127	-4108.35144
2.27	-4104.50978	-4104.97732	-4106.66670	-4107.14050	-4106.95210	-4107.42730	-4107.30290	-4107.77935	-4108.23836	-4108.75802
2.36	-4104.84989	-4105.30829	-4106.99274	-4107.46619	-4107.27908	-4107.75391	-4107.63081	-4108.10646	-4108.56148	-4109.08058
2.46	-4105.12878	-4105.57121	-4107.25200	-4107.72504	-4107.53922	-4108.01365	-4107.89146	-4108.36661	-4108.81823	-4109.33675
2.55	-4105.36566	-4105.78030	-4107.45868	-4107.93129	-4107.74671	-4108.22072	-4108.09903	-4108.57407	-4109.02277	-4109.54074
2.65	-4105.55183	-4105.95160	-4107.62405	-4108.09625	-4107.91282	-4108.38644	-4108.27067	-4108.74014	-4109.18632	-4109.70378
2.74	-4105.70265	-4106.08652	-4107.75711	-4108.22892	-4108.04654	-4108.51979	-4108.40476	-4108.87381	-4109.31779	-4109.83480
2.83	-4105.82557	-4106.19575	-4107.86480	-4108.33627	-4108.15483	-4108.62777	-4108.51340	-4108.98205	-4109.42408	-4109.94071
2.93	-4105.92619	-4106.28470	-4107.95248	-4108.42364	-4108.24304	-4108.71571	-4108.60195	-4109.07023	-4109.51049	-4110.02682
3.02	-4106.00857	-4106.35746	-4108.02418	-4108.49507	-4108.31521	-4108.78766	-4108.67446	-4109.14238	-4109.58101	-4110.09712
3.12	-4106.07588	-4106.52435	-4108.08299	-4108.55365	-4108.37446	-4108.84673	-4108.83550	-4109.20163	-4109.63875	-4110.15468
3.21	-4106.13069	-4106.57866	-4108.24005	-4108.71064	-4108.53238	-4109.00410	-4108.89016	-4109.35768	-4109.79675	-4110.31219
3.31	-4106.17511	-4106.62322	-4108.28463	-4108.75495	-4108.57753	-4109.04908	-4108.93511	-4109.40208	-4109.84067	-4110.35589
3.40	-4106.21095	-4106.65968	-4108.32117	-4108.79107	-4108.61462	-4109.08581	-4108.97204	-4109.43836	-4109.87668	-4110.39150
3.50	-4106.23977	-4106.68932	-4108.35105	-4108.82029	-4108.64502	-4109.11558	-4109.00236	-4109.46790	-4109.90623	-4110.42031
3.59	-4106.26299	-4106.71257	-4108.37529	-4108.84337	-4108.66974	-4109.13907	-4109.02724	-4109.49164	-4109.93052	-4110.44313
3.68	-4106.28184	-4106.73017	-4108.39476	-4108.86172	-4108.68964	-4109.15784	-4109.04748	-4109.51063	-4109.95030	-4110.46147
3.78	-4106.29826	-4106.74235	-4108.41130	-4108.87772	-4108.70679	-4109.17471	-4109.06503	-4109.52779	-4109.96609	-4110.47675
3.87	-4106.31068	-4106.75335	-4108.42236	-4108.88887	-4108.71809	-4109.18616	-4109.07684	-4109.53958	-4109.97628	-4110.48712
3.97	-4106.32004	-4106.76157	-4108.43058	-4108.89713	-4108.72645	-4109.19465	-4109.08582	-4109.54850	-4109.98358	-4110.49457
4.06	-4106.32681	-4106.76740	-4108.43639	-4108.90295	-4108.73236	-4109.20063	-4109.09243	-4109.55497	-4109.98845	-4110.49952
4.16	-4106.33139	-4106.77117	-4108.44014	-4108.90668	-4108.73617	-4109.20450	-4109.09699	-4109.55936	-4109.99125	-4110.50237
4.35	-4106.33541	-4106.77318	-4108.44213	-4108.90864	-4108.73819	-4109.20658	-4109.09979	-4109.56196	-4109.99227	-4110.50343
4.44	-4106.33546	-4106.77372	-4108.44265	-4108.90913	-4108.73870	-4109.20718	-4109.10110	-4109.56306	-4109.99179	-4110.50299
4.54	-4106.33455	-4106.77305	-4108.44192	-4108.90840	-4108.73795	-4109.20656	-4109.10116	-4109.56290	-4109.99003	-4110.50132
4.63	-4106.33291	-4106.77139	-4108.44016	-4108.90669	-4108.73615	-4109.20497	-4109.10017	-4109.56170	-4109.98723	-4110.49866
4.72	-4106.33073	-4106.76892	-4108.43757	-4108.90419	-4108.73349	-4109.20261	-4109.09829	-4109.55965	-4109.98357	-4110.49524
4.82	-4106.32817	-4106.76582	-4108.43427	-4108.90105	-4108.73012	-4109.19963	-4109.09567	-4109.55688	-4109.97923	-4110.49121
4.91	-4106.32536	-4106.76222	-4108.43039	-4108.89740	-4108.72618	-4109.19616	-4109.09241	-4109.55351	-4109.97433	-4110.48674
5.01	-4106.32244	-4106.75824	-4108.42599	-4108.89327	-4108.72172	-4109.19226	-4109.08856	-4109.54959	-4109.96896	-4110.48189
5.10	-4106.31950	-4106.75394	-4108.42105	-4108.88866	-4108.71673	-4109.18791	-4109.08410	-4109.54509	-4109.96307	-4110.47665
5.20	-4106.31661	-4106.74934	-4108.41563	-4108.88358	-4108.71127	-4109.18312	-4109.07910	-4109.54006	-4109.95676	-4110.47106
5.29	-4106.31378	-4106.74457	-4108.41006	-4108.87828	-4108.70569	-4109.17811	-4109.07388	-4109.53476	-4109.95042	-4110.46535
5.39	-4106.31101	-4106.73977	-4108.40459	-4108.87299	-4108.70024	-4109.17309	-4109.06874	-4109.52949	-4109.94434	-4110.45976

Table S15: The absolute energies in hartree of the potential energy curve of NbSi in the $^2\Delta$ state with a CASSCF wave function and nom-CPO active space.

Distance	CASSCF	CASPT2	tpbe	ftpbe	trevpbe	ftrevpbe	tblyp	ftblyp	torelyp	ftorelyp
5.48	-4106.30826	-4106.73506	-4108.39937	-4108.86787	-4108.69506	-4109.16821	-4109.06382	-4109.52440	-4109.93865	-4110.45441
5.57	-4106.30554	-4106.73050	-4108.39444	-4108.86300	-4108.69019	-4109.16356	-4109.05919	-4109.51960	-4109.93338	-4110.44939
5.67	-4106.30282	-4106.72611	-4108.38981	-4108.85840	-4108.68563	-4109.15915	-4109.05486	-4109.51510	-4109.92852	-4110.44470
5.76	-4106.30012	-4106.72186	-4108.38543	-4108.85403	-4108.68134	-4109.15496	-4109.05078	-4109.51085	-4109.92401	-4110.44030
5.86	-4106.29743	-4106.71774	-4108.38128	-4108.84986	-4108.67727	-4109.15096	-4109.04693	-4109.50683	-4109.91979	-4110.43615
5.95	-4106.29477	-4106.71376	-4108.37732	-4108.84588	-4108.67341	-4109.14713	-4109.04328	-4109.50303	-4109.91581	-4110.43223
6.05	-4106.29215	-4106.70990	-4108.37355	-4108.84207	-4108.66973	-4109.14345	-4109.03982	-4109.49940	-4109.91207	-4110.42851
6.14	-4106.28956	-4106.70618	-4108.36994	-4108.83841	-4108.66621	-4109.13991	-4109.03653	-4109.49595	-4109.90853	-4110.42497
6.24	-4106.28703	-4106.70259	-4108.36650	-4108.83491	-4108.66286	-4109.13652	-4109.03341	-4109.49268	-4109.90518	-4110.42162
6.33	-4106.28455	-4106.69914	-4108.36322	-4108.83156	-4108.65967	-4109.13327	-4109.03047	-4109.48959	-4109.90203	-4110.41844
6.43	-4106.28215	-4106.69584	-4108.36009	-4108.82835	-4108.65663	-4109.13015	-4109.02769	-4109.48665	-4109.89905	-4110.41542
6.52	-4106.27982	-4106.69268	-4108.35712	-4108.82530	-4108.65373	-4109.12717	-4109.02507	-4109.48389	-4109.89625	-4110.41257
6.61	-4106.27757	-4106.68967	-4108.35429	-4108.82239	-4108.65098	-4109.12432	-4109.02261	-4109.48130	-4109.89361	-4110.40988
6.71	-4106.27541	-4106.68682	-4108.35161	-4108.81962	-4108.64838	-4109.12160	-4109.02031	-4109.47888	-4109.89115	-4110.40734
6.80	-4106.27335	-4106.68412	-4108.34907	-4108.81701	-4108.64591	-4109.11902	-4109.01817	-4109.47663	-4109.88885	-4110.40498
6.90	-4106.27140	-4106.68158	-4108.34667	-4108.81455	-4108.64359	-4109.11658	-4109.01620	-4109.47457	-4109.88671	-4110.40278
6.99	-4106.26955	-4106.67920	-4108.34441	-4108.81222	-4108.64139	-4109.11427	-4109.01437	-4109.47268	-4109.88472	-4110.40073
7.09	-4106.26782	-4106.67698	-4108.34226	-4108.81002	-4108.63931	-4109.11207	-4109.01268	-4109.47096	-4109.88288	-4110.39882
7.18	-4106.26620	-4106.67492	-4108.34022	-4108.80794	-4108.63734	-4109.10999	-4109.01112	-4109.46939	-4109.88117	-4110.39705
7.28	-4106.26471	-4106.67303	-4108.33829	-4108.80599	-4108.63547	-4109.10803	-4109.00968	-4109.46796	-4109.87959	-4110.39540
7.37	-4106.26333	-4106.67129	-4108.33646	-4108.80414	-4108.63371	-4109.10616	-4109.00835	-4109.46667	-4109.87811	-4110.39387
7.46	-4106.26207	-4106.66970	-4108.33472	-4108.80238	-4108.63202	-4109.10438	-4109.00710	-4109.46548	-4109.87673	-4110.39243
7.56	-4106.26092	-4106.66825	-4108.33305	-4108.80070	-4108.63042	-4109.10269	-4109.00593	-4109.46437	-4109.87543	-4110.39108
7.65	-4106.25988	-4106.66693	-4108.33145	-4108.79910	-4108.62889	-4109.10108	-4109.00481	-4109.46335	-4109.87422	-4110.38980
7.75	-4106.25893	-4106.66573	-4108.32993	-4108.79758	-4108.62743	-4109.09955	-4109.00376	-4109.46239	-4109.87307	-4110.38859
7.84	-4106.25808	-4106.66464	-4108.32848	-4108.79614	-4108.62605	-4109.09809	-4109.00275	-4109.46149	-4109.87200	-4110.38745
7.94	-4106.25732	-4106.66365	-4108.32710	-4108.79476	-4108.62475	-4109.09672	-4109.00180	-4109.46063	-4109.87099	-4110.38637
8.03	-4106.25663	-4106.66275	-4108.32581	-4108.79347	-4108.62351	-4109.09543	-4109.00088	-4109.45982	-4109.87005	-4110.38537
8.13	-4106.25601	-4106.66193	-4108.32458	-4108.79225	-4108.62236	-4109.09422	-4109.00002	-4109.45906	-4109.86918	-4110.38443
8.22	-4106.25546	-4106.66118	-4108.32343	-4108.79111	-4108.62128	-4109.09309	-4108.99920	-4109.45835	-4109.86837	-4110.38356
8.31	-4106.25497	-4106.66050	-4108.32235	-4108.79004	-4108.62027	-4109.09204	-4108.99842	-4109.45767	-4109.86762	-4110.38275
8.41	-4106.25453	-4106.65989	-4108.32135	-4108.78904	-4108.61934	-4109.09107	-4108.99769	-4109.45703	-4109.86694	-4110.38202
8.50	-4106.25414	-4106.65934	-4108.32042	-4108.78812	-4108.61848	-4109.09018	-4108.99700	-4109.45644	-4109.86633	-4110.38136
8.60	-4106.25379	-4106.65885	-4108.31957	-4108.78729	-4108.61771	-4109.08938	-4108.99637	-4109.45590	-4109.86580	-4110.38078
8.69	-4106.25348	-4106.65842	-4108.31883	-4108.78655	-4108.61704	-4109.08868	-4108.99582	-4109.45543	-4109.86534	-4110.38028
8.79	-4106.25321	-4106.65804	-4108.31818	-4108.78590	-4108.61646	-4109.08807	-4108.99534	-4109.45502	-4109.86495	-4110.37985
8.88	-4106.25297	-4106.65770	-4108.31761	-4108.78533	-4108.61596	-4109.08754	-4108.99493	-4109.45466	-4109.86462	-4110.37948
8.98	-4106.25275	-4106.65739	-4108.31711	-4108.78482	-4108.61552	-4109.08708	-4108.99457	-4109.45434	-4109.86434	-4110.37916
9.07	-4106.25255	-4106.65711	-4108.31667	-4108.78438	-4108.61514	-4109.08667	-4108.99426	-4109.45406	-4109.86411	-4110.37888
9.17	-4106.25237	-4106.65685	-4108.31628	-4108.78398	-4108.61481	-4109.08632	-4108.99399	-4109.45381	-4109.86391	-4110.37865
9.26	-4106.25221	-4106.65661	-4108.31593	-4108.78363	-4108.61452	-4109.08601	-4108.99376	-4109.45360	-4109.86375	-4110.37845
9.35	-4106.25207	-4106.65639	-4108.31563	-4108.78332	-4108.61426	-4109.08573	-4108.99356	-4109.45340	-4109.86361	-4110.37827
9.45	-4106.25194	-4106.65619	-4108.31536	-4108.78304	-4108.61405	-4109.08549	-4108.99338	-4109.45324	-4109.86349	-4110.37813
9.54	-4106.25182	-4106.65600	-4108.31512	-4108.78279	-4108.61385	-4109.08528	-4108.99323	-4109.45309	-4109.86340	-4110.37800
9.64	-4106.25171	-4106.65583	-4108.31490	-4108.78257	-4108.61368	-4109.08510	-4108.99310	-4109.45296	-4109.86332	-4110.37790
9.73	-4106.25162	-4106.65567	-4108.31471	-4108.78237	-4108.61354	-4109.08493	-4108.99298	-4109.45285	-4109.86326	-4110.37781
9.83	-4106.25153	-4106.65552	-4108.31454	-4108.78219	-4108.61341	-4109.08479	-4108.99288	-4109.45275	-4109.86321	-4110.37773
9.92	-4106.25145	-4106.65537	-4108.31438	-4108.78202	-4108.61329	-4109.08465	-4108.99278	-4109.45265	-4109.86316	-4110.37766
10.02	-4106.25137	-4106.65524	-4108.31425	-4108.78188	-4108.61319	-4109.08455	-4108.99271	-4109.45257	-4109.86314	-4110.37761
10.11	-4106.25130	-4106.65511	-4108.31412	-4108.78175	-4108.61310	-4109.08444	-4108.99264	-4109.45250	-4109.86311	-4110.37756
10.20	-4106.25124	-4106.65500	-4108.31402	-4108.78164	-4108.61304	-4109.08436	-4108.99259	-4109.45245	-4109.86310	-4110.37753
10.30	-4106.25119	-4106.65489	-4108.31391	-4108.78152	-4108.61296	-4109.08428	-4108.99253	-4109.45239	-4109.86308	-4110.37749

Table S15: The absolute energies in hartree of the potential energy curve of NbSi in the $^2\Delta$ state with a CASSCF wave function and nom-CPO active space.

Distance	CASSCF	CASPT2	tpbe	ftpbe	trevpbe	ftrevpbe	tblyp	ftblyp	torelyp	ftorelyp
10.39	-4106.25113	-4106.65479	-4108.31384	-4108.78144	-4108.61292	-4109.08422	-4108.99251	-4109.45236	-4109.86309	-4110.37748
10.49	-4106.25108	-4106.65469	-4108.31375	-4108.78134	-4108.61286	-4109.08415	-4108.99246	-4109.45231	-4109.86308	-4110.37745
10.58	-4106.25104	-4106.65460	-4108.31368	-4108.78127	-4108.61282	-4109.08409	-4108.99243	-4109.45228	-4109.86308	-4110.37744
10.68	-4106.25100	-4106.65452	-4108.31361	-4108.78120	-4108.61278	-4109.08405	-4108.99241	-4109.45225	-4109.86308	-4110.37743
10.77	-4106.25096	-4106.65443	-4108.31355	-4108.78113	-4108.61274	-4109.08400	-4108.99239	-4109.45222	-4109.86308	-4110.37742
10.87	-4106.25093	-4106.65436	-4108.31350	-4108.78107	-4108.61271	-4109.08397	-4108.99237	-4109.45220	-4109.86309	-4110.37741
10.96	-4106.25089	-4106.65429	-4108.31345	-4108.78102	-4108.61269	-4109.08393	-4108.99236	-4109.45218	-4109.86310	-4110.37740
11.05	-4106.25086	-4106.65422	-4108.31341	-4108.78097	-4108.61267	-4109.08390	-4108.99235	-4109.45216	-4109.86311	-4110.37740
11.15	-4106.25084	-4106.65416	-4108.31337	-4108.78093	-4108.61265	-4109.08388	-4108.99234	-4109.45215	-4109.86312	-4110.37740
11.24	-4106.25081	-4106.65410	-4108.31334	-4108.78089	-4108.61264	-4109.08386	-4108.99234	-4109.45215	-4109.86313	-4110.37739
11.34	-4106.25079	-4106.65404	-4108.31331	-4108.78085	-4108.61262	-4109.08383	-4108.99233	-4109.45214	-4109.86314	-4110.37739
11.43	-4106.25076	-4106.65399	-4108.31328	-4108.78082	-4108.61261	-4109.08382	-4108.99234	-4109.45214	-4109.86315	-4110.37739
11.53	-4106.25074	-4106.65394	-4108.31325	-4108.78079	-4108.61260	-4109.08380	-4108.99234	-4109.45213	-4109.86316	-4110.37740
11.62	-4106.25072	-4106.65389	-4108.31324	-4108.78077	-4108.61260	-4109.08379	-4108.99234	-4109.45213	-4109.86317	-4110.37740
11.72	-4106.25071	-4106.65385	-4108.31321	-4108.78075	-4108.61259	-4109.08378	-4108.99234	-4109.45213	-4109.86318	-4110.37740
11.81	-4106.25069	-4106.65381	-4108.31319	-4108.78072	-4108.61258	-4109.08376	-4108.99234	-4109.45213	-4109.86319	-4110.37740
11.91	-4106.25067	-4106.65376	-4108.31317	-4108.78070	-4108.61257	-4109.08375	-4108.99235	-4109.45213	-4109.86319	-4110.37739
12.00	-4106.25066	-4106.65373	-4108.31316	-4108.78068	-4108.61257	-4109.08374	-4108.99235	-4109.45214	-4109.86320	-4110.37740
12.09	-4106.25065	-4106.65369	-4108.31314	-4108.78066	-4108.61256	-4109.08373	-4108.99236	-4109.45214	-4109.86321	-4110.37740
12.19	-4106.25063	-4106.65365	-4108.31313	-4108.78065	-4108.61256	-4109.08372	-4108.99236	-4109.45214	-4109.86321	-4110.37739
12.28	-4106.25062	-4106.65362	-4108.31311	-4108.78063	-4108.61255	-4109.08371	-4108.99237	-4109.45214	-4109.86322	-4110.37739
12.38	-4106.25061	-4106.65358	-4108.31310	-4108.78061	-4108.61255	-4109.08371	-4108.99238	-4109.45215	-4109.86322	-4110.37739
12.47	-4106.25060	-4106.65355	-4108.31309	-4108.78060	-4108.61255	-4109.08370	-4108.99238	-4109.45215	-4109.86323	-4110.37739
12.57	-4106.25059	-4106.65352	-4108.31308	-4108.78059	-4108.61254	-4109.08369	-4108.99239	-4109.45216	-4109.86323	-4110.37739
12.66	-4106.25058	-4106.65349	-4108.31307	-4108.78058	-4108.61254	-4109.08369	-4108.99239	-4109.45216	-4109.86323	-4110.37739
12.76	-4106.25057	-4106.65346	-4108.31306	-4108.78057	-4108.61254	-4109.08368	-4108.99240	-4109.45217	-4109.86324	-4110.37739
12.85	-4106.25056	-4106.65344	-4108.31305	-4108.78056	-4108.61253	-4109.08367	-4108.99240	-4109.45217	-4109.86324	-4110.37739
12.94	-4106.25055	-4106.65342	-4108.31304	-4108.78055	-4108.61253	-4109.08367	-4108.99241	-4109.45218	-4109.86325	-4110.37739
13.04	-4106.25054	-4106.65340	-4108.31304	-4108.78055	-4108.61253	-4109.08367	-4108.99242	-4109.45219	-4109.86325	-4110.37739
13.13	-4106.25054	-4106.65338	-4108.31303	-4108.78054	-4108.61253	-4109.08367	-4108.99243	-4109.45219	-4109.86325	-4110.37739
13.23	-4106.25053	-4106.65335	-4108.31302	-4108.78053	-4108.61252	-4109.08366	-4108.99243	-4109.45219	-4109.86325	-4110.37738
13.32	-4106.25052	-4106.65333	-4108.31302	-4108.78052	-4108.61252	-4109.08365	-4108.99244	-4109.45220	-4109.86325	-4110.37738
13.42	-4106.25052	-4106.65331	-4108.31301	-4108.78051	-4108.61252	-4109.08365	-4108.99244	-4109.45220	-4109.86325	-4110.37738
13.51	-4106.25051	-4106.65329	-4108.31301	-4108.78051	-4108.61252	-4109.08365	-4108.99245	-4109.45221	-4109.86325	-4110.37738
13.61	-4106.25051	-4106.65327	-4108.31300	-4108.78050	-4108.61252	-4109.08364	-4108.99246	-4109.45222	-4109.86325	-4110.37738
13.70	-4106.25050	-4106.65325	-4108.31300	-4108.78050	-4108.61251	-4109.08364	-4108.99246	-4109.45222	-4109.86325	-4110.37737
13.80	-4106.25050	-4106.65323	-4108.31299	-4108.78049	-4108.61251	-4109.08363	-4108.99246	-4109.45222	-4109.86325	-4110.37737
13.89	-4106.25049	-4106.65322	-4108.31299	-4108.78049	-4108.61251	-4109.08363	-4108.99247	-4109.45223	-4109.86325	-4110.37737
13.98	-4106.25049	-4106.65276	-4108.31298	-4108.78048	-4108.61251	-4109.08363	-4108.99247	-4109.45223	-4109.86325	-4110.37737
14.08	-4106.25048	-4106.65274	-4108.31298	-4108.78048	-4108.61251	-4109.08363	-4108.99248	-4109.45223	-4109.86325	-4110.37737
14.17	-4106.25048	-4106.65273	-4108.31298	-4108.78048	-4108.61250	-4109.08363	-4108.99248	-4109.45224	-4109.86325	-4110.37737
14.27	-4106.25047	-4106.65271	-4108.31298	-4108.78047	-4108.61250	-4109.08363	-4108.99249	-4109.45224	-4109.86325	-4110.37736
14.36	-4106.25047	-4106.65270	-4108.31297	-4108.78047	-4108.61250	-4109.08362	-4108.99249	-4109.45225	-4109.86325	-4110.37736
14.46	-4106.25047	-4106.65268	-4108.31297	-4108.78047	-4108.61250	-4109.08362	-4108.99250	-4109.45225	-4109.86325	-4110.37736
14.55	-4106.25046	-4106.65267	-4108.31297	-4108.78046	-4108.61250	-4109.08362	-4108.99250	-4109.45225	-4109.86325	-4110.37736
14.65	-4106.25046	-4106.65265	-4108.31296	-4108.78046	-4108.61250	-4109.08362	-4108.99250	-4109.45226	-4109.86325	-4110.37736
14.74	-4106.25046	-4106.65264	-4108.31296	-4108.78046	-4108.61250	-4109.08361	-4108.99251	-4109.45226	-4109.86325	-4110.37736
14.83	-4106.25045	-4106.65263	-4108.31296	-4108.78046	-4108.61250	-4109.08361	-4108.99251	-4109.45226	-4109.86325	-4110.37736
14.93	-4106.25045	-4106.65261	-4108.31296	-4108.78046	-4108.61250	-4109.08361	-4108.99251	-4109.45227	-4109.86325	-4110.37736
15.02	-4106.25045	-4106.65260	-4108.31296	-4108.78045	-4108.61250	-4109.08361	-4108.99252	-4109.45227	-4109.86325	-4110.37736
15.12	-4106.25044	-4106.65258	-4108.31296	-4108.78045	-4108.61249	-4109.08361	-4108.99252	-4109.45227	-4109.86325	-4110.37736
15.21	-4106.25044	-4106.65257	-4108.31296	-4108.78045	-4108.61249	-4109.08361	-4108.99252	-4109.45228	-4109.86325	-4110.37735

Table S15: The absolute energies in hartree of the potential energy curve of NbSi in the $^2\Delta$ state with a CASSCF wave function and nom-CPO active space.

Distance	CASSCF	CASPT2	tpbe	ftpbe	trevpbe	ftrevpbe	tblyp	ftblyp	torelyp	ftorelyp
15.31	-4106.25044	-4106.65255	-4108.31296	-4108.78045	-4108.61249	-4109.08361	-4108.99253	-4109.45228	-4109.86325	-4110.37735
15.40	-4106.25044	-4106.65254	-4108.31295	-4108.78045	-4108.61249	-4109.08361	-4108.99253	-4109.45228	-4109.86325	-4110.37735
15.50	-4106.25044	-4106.65252	-4108.31295	-4108.78045	-4108.61249	-4109.08361	-4108.99253	-4109.45228	-4109.86325	-4110.37735
15.59	-4106.25043	-4106.65250	-4108.31295	-4108.78045	-4108.61249	-4109.08361	-4108.99253	-4109.45229	-4109.86325	-4110.37735
15.68	-4106.25043	-4106.65248	-4108.31295	-4108.78045	-4108.61249	-4109.08360	-4108.99254	-4109.45229	-4109.86325	-4110.37735
15.78	-4106.25043	-4106.65246	-4108.31295	-4108.78044	-4108.61249	-4109.08360	-4108.99254	-4109.45229	-4109.86325	-4110.37735
15.87	-4106.25043	-4106.65244	-4108.31295	-4108.78044	-4108.61249	-4109.08360	-4108.99254	-4109.45229	-4109.86325	-4110.37735
15.97	-4106.25042	-4106.65242	-4108.31295	-4108.78044	-4108.61248	-4109.08360	-4108.99254	-4109.45230	-4109.86325	-4110.37735
16.06	-4106.25042	-4106.65240	-4108.31295	-4108.78044	-4108.61248	-4109.08360	-4108.99254	-4109.45230	-4109.86325	-4110.37735
16.16	-4106.25042	-4106.65239	-4108.31295	-4108.78044	-4108.61248	-4109.08360	-4108.99255	-4109.45230	-4109.86325	-4110.37735
16.25	-4106.25042	-4106.65238	-4108.31295	-4108.78044	-4108.61248	-4109.08360	-4108.99255	-4109.45230	-4109.86325	-4110.37734
16.35	-4106.25042	-4106.65238	-4108.31295	-4108.78044	-4108.61248	-4109.08360	-4108.99255	-4109.45230	-4109.86325	-4110.37734
16.44	-4106.25042	-4106.65237	-4108.31295	-4108.78044	-4108.61248	-4109.08360	-4108.99255	-4109.45231	-4109.86325	-4110.37734
16.54	-4106.25042	-4106.65237	-4108.31294	-4108.78044	-4108.61248	-4109.08360	-4108.99255	-4109.45231	-4109.86324	-4110.37734
16.63	-4106.25041	-4106.65237	-4108.31294	-4108.78044	-4108.61248	-4109.08360	-4108.99256	-4109.45231	-4109.86324	-4110.37734
16.72	-4106.25041	-4106.65237	-4108.31294	-4108.78044	-4108.61248	-4109.08360	-4108.99256	-4109.45231	-4109.86324	-4110.37734
16.82	-4106.25041	-4106.65236	-4108.31294	-4108.78044	-4108.61248	-4109.08360	-4108.99256	-4109.45231	-4109.86324	-4110.37734
16.91	-4106.25041	-4106.65235	-4108.31294	-4108.78044	-4108.61248	-4109.08359	-4108.99256	-4109.45231	-4109.86324	-4110.37734
17.01	-4106.25041	-4106.65234	-4108.31294	-4108.78043	-4108.61248	-4109.08359	-4108.99256	-4109.45231	-4109.86324	-4110.37734
17.10	-4106.25041	-4106.65234	-4108.31294	-4108.78043	-4108.61248	-4109.08359	-4108.99256	-4109.45231	-4109.86324	-4110.37734
17.20	-4106.25041	-4106.65234	-4108.31294	-4108.78043	-4108.61248	-4109.08359	-4108.99256	-4109.45231	-4109.86324	-4110.37734
17.29	-4106.25041	-4106.65231	-4108.31294	-4108.78043	-4108.61248	-4109.08359	-4108.99256	-4109.45231	-4109.86324	-4110.37734
17.39	-4106.25040	-4106.65230	-4108.31294	-4108.78043	-4108.61248	-4109.08359	-4108.99256	-4109.45232	-4109.86324	-4110.37734
17.48	-4106.25040	-4106.65229	-4108.31294	-4108.78043	-4108.61248	-4109.08359	-4108.99256	-4109.45232	-4109.86324	-4110.37734
17.57	-4106.25040	-4106.65228	-4108.31294	-4108.78043	-4108.61248	-4109.08359	-4108.99257	-4109.45232	-4109.86324	-4110.37734
17.67	-4106.25040	-4106.65228	-4108.31294	-4108.78043	-4108.61248	-4109.08359	-4108.99257	-4109.45232	-4109.86324	-4110.37734
17.76	-4106.25040	-4106.65228	-4108.31294	-4108.78043	-4108.61248	-4109.08359	-4108.99257	-4109.45232	-4109.86324	-4110.37734
17.86	-4106.25040	-4106.65228	-4108.31294	-4108.78043	-4108.61248	-4109.08359	-4108.99257	-4109.45232	-4109.86324	-4110.37734
17.95	-4106.25040	-4106.65228	-4108.31294	-4108.78043	-4108.61248	-4109.08359	-4108.99257	-4109.45232	-4109.86324	-4110.37734
18.05	-4106.25040	-4106.65228	-4108.31294	-4108.78044	-4108.61248	-4109.08359	-4108.99257	-4109.45233	-4109.86325	-4110.37734

Table S16: The absolute energies in hartree of the potential energy curve of NbSi in the $^4\Pi$ state with a SP-based wave function and nom-CPO active space.

Distance	SP	tpbe	ftpbe	trevpbe	ftrevpbe	tblyp	ftblyp	torelyp	ftorelyp
1.98	-4101.97596	-4104.18838	-4104.65830	-4104.46747	-4104.93924	-4104.82742	-4105.29378	-4105.76675	-4106.28192
2.08	-4102.78409	-4104.98985	-4105.45953	-4105.27014	-4105.74163	-4105.63072	-4106.09677	-4106.56450	-4107.07937
2.17	-4103.43704	-4105.63527	-4106.10479	-4105.91684	-4106.38815	-4106.27753	-4106.74331	-4107.20692	-4107.72156
2.27	-4103.96285	-4106.15264	-4106.62217	-4106.43553	-4106.90685	-4106.79590	-4107.26152	-4107.72208	-4108.23678
2.36	-4104.38917	-4106.56681	-4107.03611	-4106.85071	-4107.32177	-4107.21148	-4107.67658	-4108.13532	-4108.64950
2.46	-4104.73767	-4106.90563	-4107.37400	-4107.19042	-4107.66004	-4107.55173	-4108.01612	-4108.47283	-4108.98493
2.55	-4105.01983	-4107.18076	-4107.64847	-4107.46644	-4107.93521	-4107.82772	-4108.29152	-4108.74601	-4109.25691
2.65	-4105.24765	-4107.40256	-4107.86996	-4107.68911	-4108.15754	-4108.05034	-4108.51380	-4108.96589	-4109.47635
2.74	-4105.43200	-4107.58175	-4108.04905	-4107.86913	-4108.33747	-4108.23038	-4108.69371	-4109.14333	-4109.65374
2.83	-4105.57146	-4107.71336	-4108.18146	-4108.00030	-4108.46993	-4108.36685	-4108.83195	-4109.26592	-4109.77895
2.93	-4105.70422	-4107.84493	-4108.31304	-4108.13259	-4108.60227	-4108.49909	-4108.96401	-4109.39647	-4109.90950
3.02	-4105.81476	-4107.95531	-4108.42351	-4108.24368	-4108.71353	-4108.60989	-4109.07461	-4109.50615	-4110.01935
3.12	-4105.90758	-4108.04814	-4108.51647	-4108.33717	-4108.80740	-4108.70283	-4109.16720	-4109.59831	-4110.11200
3.21	-4105.98683	-4108.12584	-4108.59416	-4108.41543	-4108.88610	-4108.78043	-4109.24420	-4109.67495	-4110.18928
3.31	-4106.05431	-4108.19045	-4108.65846	-4108.48048	-4108.95110	-4108.84527	-4109.30848	-4109.73838	-4110.25272
3.40	-4106.11104	-4108.24437	-4108.71204	-4108.53478	-4109.00523	-4108.89957	-4109.36228	-4109.79124	-4110.30541

Table S16: The absolute energies in hartree of the potential energy curve of NbSi in the $^4\Pi$ state with a SP-based wave function and nom-CPO active space.

Distance	SP	tpbe	ftpbe	trevpbe	ftrevpbe	tblyp	ftblyp	torelyp	ftorelyp
3.50	-4106.15839	-4108.28918	-4108.75653 -4108.57994	-4109.05020	-4108.94480	-4109.40707	-4109.83511	-4110.34906	
3.59	-4106.19765	-4108.32615	-4108.79319 -4108.61724	-4109.08730	-4108.98222	-4109.44407	-4109.87124	-4110.38494	
3.68	-4106.22997	-4108.35635	-4108.82312 -4108.64775	-4109.11762	-4109.01291	-4109.47438	-4109.90071	-4110.41417	
3.78	-4106.25634	-4108.38081	-4108.84733 -4108.67250	-4109.14220	-4109.03787	-4109.49900	-4109.92454	-4110.43777	
3.87	-4106.27762	-4108.40039	-4108.86670 -4108.69235	-4109.16191	-4109.05795	-4109.51877	-4109.94358	-4110.45661	
3.97	-4106.29454	-4108.41577	-4108.88190 -4108.70800	-4109.17743	-4109.07387	-4109.53439	-4109.95850	-4110.47136	
4.06	-4106.30775	-4108.42759	-4108.89356 -4108.72008	-4109.18940	-4109.08622	-4109.54648	-4109.96993	-4110.48263	
4.16	-4106.31688	-4108.43554	-4108.90147 -4108.72839	-4109.19765	-4109.09461	-4109.55485	-4109.97859	-4110.49112	
4.35	-4106.32449	-4108.44191	-4108.90771 -4108.73500	-4109.20417	-4109.10153	-4109.56150	-4109.98468	-4110.49710	
4.44	-4106.32985	-4108.44619	-4108.91186 -4108.73948	-4109.20858	-4109.10635	-4109.56604	-4109.98871	-4110.50103	
4.54	-4106.33334	-4108.44871	-4108.91427 -4108.74219	-4109.21124	-4109.10940	-4109.56882	-4109.99102	-4110.50325	
4.63	-4106.33528	-4108.44979	-4108.91525 -4108.74346	-4109.21246	-4109.11100	-4109.57015	-4109.99192	-4110.50406	
4.72	-4106.33594	-4108.44971	-4108.91507 -4108.74354	-4109.21251	-4109.11143	-4109.57030	-4109.99167	-4110.50375	
4.82	-4106.33556	-4108.44868	-4108.91396 -4108.74267	-4109.21162	-4109.11091	-4109.56949	-4109.99050	-4110.50251	
4.91	-4106.33435	-4108.44689	-4108.91209	-4108.73875	-4109.20997	-4109.10960	-4109.56790	-4109.98857	-4110.50052
5.01	-4106.33249	-4108.44448	-4108.90962	-4108.73599	-4109.20770	-4109.10766	-4109.56567	-4109.98602	-4110.49793
5.10	-4106.33012	-4108.44160	-4108.90667	-4108.73286	-4109.20496	-4109.10523	-4109.56294	-4109.98299	-4110.49486
5.20	-4106.32738	-4108.43836	-4108.90339	-4108.72947	-4109.20188	-4109.10242	-4109.55983	-4109.97961	-4110.49145
5.29	-4106.32438	-4108.43486	-4108.89984	-4108.72586	-4109.19853	-4109.09933	-4109.55643	-4109.97594	-4110.48776
5.39	-4106.32121	-4108.43116	-4108.89610	-4108.72212	-4109.19498	-4109.09601	-4109.55282	-4109.97206	-4110.48387
5.48	-4106.31793	-4108.42733	-4108.89223	-4108.71830	-4109.19130	-4109.09252	-4109.54904	-4109.96803	-4110.47984
5.57	-4106.31460	-4108.42342	-4108.88828	-4108.71448	-4109.18754	-4109.08895	-4109.54518	-4109.96394	-4110.47574
5.67	-4106.31126	-4108.41952	-4108.88434	-4108.71072	-4109.18377	-4109.08536	-4109.54131	-4109.95986	-4110.47165
5.76	-4106.30794	-4108.41568	-4108.88046	-4108.70706	-4109.18006	-4109.08183	-4109.53750	-4109.95587	-4110.46764
5.86	-4106.30466	-4108.41195	-4108.87668	-4108.70368	-4109.17644	-4109.07838	-4109.53378	-4109.95199	-4110.46377
5.95	-4106.30145	-4108.40849	-4108.87316	-4108.70058	-4109.17307	-4109.07522	-4109.53035	-4109.94850	-4110.46020
6.05	-4106.29831	-4108.40533	-4108.86993	-4108.69826	-4109.17000	-4109.07234	-4109.52720	-4109.94538	-4110.45701
6.14	-4106.29527	-4108.40293	-4108.86749	-4108.69338	-4109.16775	-4109.07023	-4109.52486	-4109.94342	-4110.45499
6.24	-4106.29261	-4108.39786	-4108.86335	-4108.68990	-4109.16425	-4109.06530	-4109.52057	-4109.93986	-4110.45227
6.33	-4106.29032	-4108.39432	-4108.85996	-4108.68688	-4109.16107	-4109.06191	-4109.51710	-4109.93624	-4110.44881
6.43	-4106.28820	-4108.39126	-4108.85701	-4108.66026	-4109.15830	-4109.05900	-4109.51409	-4109.93305	-4110.44574
6.52	-4106.28358	-4108.36402	-4108.83113	-4108.65659	-4109.13253	-4109.03146	-4109.48880	-4109.90127	-4110.44290
6.61	-4106.28121	-4108.36024	-4108.82743	-4108.65356	-4109.12896	-4109.02798	-4109.48538	-4109.89767	-4110.44021
6.71	-4106.27891	-4108.35711	-4108.82434	-4108.65085	-4109.12595	-4109.02518	-4109.48259	-4109.89475	-4110.43765
6.80	-4106.27667	-4108.35431	-4108.82156	-4108.64837	-4109.12325	-4109.02273	-4109.48016	-4109.89217	-4110.43520
6.90	-4106.27451	-4108.35175	-4108.81902	-4108.64610	-4109.12078	-4109.02055	-4109.47800	-4109.88987	-4110.43288
6.99	-4106.27241	-4108.34940	-4108.81669	-4108.64401	-4109.11850	-4109.01859	-4109.47607	-4109.88778	-4110.43068
7.09	-4106.27040	-4108.34723	-4108.81454	-4108.64205	-4109.11640	-4109.01684	-4109.47437	-4109.88590	-4110.42859
7.18	-4106.26848	-4108.34520	-4108.81254	-4108.64025	-4109.11443	-4109.01525	-4109.47284	-4109.88419	-4110.42661
7.28	-4106.26665	-4108.34332	-4108.81069	-4108.63857	-4109.11260	-4109.01382	-4109.47148	-4109.88264	-4110.42470
7.37	-4106.26491	-4108.34157	-4108.80897	-4108.63701	-4109.11089	-4109.01254	-4109.47029	-4109.88124	-4110.42290
7.46	-4106.26328	-4108.33994	-4108.80737	-4108.63556	-4109.10930	-4109.01139	-4109.46924	-4109.87998	-4110.42010
7.56	-4106.26176	-4108.33842	-4108.80588	-4108.63421	-4109.10782	-4109.01035	-4109.46833	-4109.87884	-4110.41804
7.65	-4106.26034	-4108.33700	-4108.80450	-4108.63294	-4109.10644	-4109.00943	-4109.46752	-4109.87781	-4110.41645
7.75	-4106.25903	-4108.33567	-4108.80320	-4108.63175	-4109.10514	-4109.00858	-4109.46681	-4109.87688	-4110.41522
7.84	-4106.25784	-4108.33441	-4108.80197	-4108.63062	-4109.10392	-4109.00780	-4109.46616	-4109.87602	-4110.41419
7.94	-4106.25675	-4108.33320	-4108.80080	-4108.62953	-4109.10275	-4109.00706	-4109.46555	-4109.87522	-4110.41324
8.03	-4106.25577	-4108.33204	-4108.79967	-4108.62848	-4109.10163	-4109.00635	-4109.46497	-4109.87445	-4110.41238
8.13	-4106.25489	-4108.33092	-4108.79858	-4108.62746	-4109.10054	-4109.00564	-4109.46440	-4109.87372	-4110.41152
8.22	-4106.25410	-4108.32983	-4108.79751	-4108.62650	-4109.09949	-4109.00494	-4109.46382	-4109.87300	-4110.41070
8.31	-4106.25340	-4108.32880	-4108.79650	-4108.62558	-4109.09850	-4109.00427	-4109.46326	-4109.87233	-4110.40995
8.41	-4106.25278	-4108.32780	-4108.79552	-4108.62471	-4109.09755	-4109.00360	-4109.46270	-4109.87168	-4110.40930

Table S16: The absolute energies in hartree of the potential energy curve of NbSi in the $^4\Pi$ state with a SP-based wave function and nom-CPO active space.

Distance	SP	tpbe	ftpbe	trevpbe	ftrevpbe	tblyp	ftblyp	torelyp	ftorelyp
8.50	-4106.25223	-4108.32687	-4108.79460	-4108.62391	-4109.09665	-4109.00296	-4109.46216	-4109.87108	-4110.40872
8.60	-4106.25175	-4108.32599	-4108.79374	-4108.62317	-4109.09582	-4109.00235	-4109.46164	-4109.87052	-4110.40822
8.69	-4106.25132	-4108.32518	-4108.79294	-4108.62248	-4109.09506	-4109.00178	-4109.46115	-4109.87000	-4110.40780
8.79	-4106.25094	-4108.32443	-4108.79220	-4108.62186	-4109.09435	-4109.00125	-4109.46068	-4109.86953	-4110.40741
8.88	-4106.25061	-4108.32374	-4108.79151	-4108.62124	-4109.09371	-4109.00075	-4109.46025	-4109.86911	-4110.40702
8.98	-4106.25032	-4108.32305	-4108.79083	-4108.62072	-4109.09307	-4109.00024	-4109.45981	-4109.86869	-4110.40661
9.07	-4106.25007	-4108.32246	-4108.79024	-4108.62032	-4109.09251	-4108.99981	-4109.45941	-4109.86834	-4110.40622
9.17	-4106.24984	-4108.32200	-4108.78977	-4108.61999	-4109.09209	-4108.99947	-4109.45911	-4109.86809	-4110.40586
9.26	-4106.24964	-4108.32161	-4108.78937	-4108.61971	-4109.09173	-4108.99920	-4109.45885	-4109.86790	-4110.40555
9.35	-4106.24946	-4108.32128	-4108.78902	-4108.61948	-4109.09142	-4108.99897	-4109.45863	-4109.86775	-4110.40529
9.45	-4106.24930	-4108.32099	-4108.78873	-4108.61929	-4109.09116	-4108.99878	-4109.45845	-4109.86764	-4110.40506
9.54	-4106.24915	-4108.32075	-4108.78847	-4108.61914	-4109.09094	-4108.99862	-4109.45829	-4109.86756	-4110.40486
9.64	-4106.24902	-4108.32055	-4108.78825	-4108.61899	-4109.09076	-4108.99850	-4109.45816	-4109.86752	-4110.40466
9.73	-4106.24890	-4108.32036	-4108.78804	-4108.61886	-4109.09059	-4108.99838	-4109.45803	-4109.86747	-4110.40444
9.83	-4106.24879	-4108.32018	-4108.78784	-4108.61874	-4109.09043	-4108.99827	-4109.45791	-4109.86742	-4110.40418
9.92	-4106.24869	-4108.32002	-4108.78767	-4108.61866	-4109.09029	-4108.99817	-4109.45781	-4109.86738	-4110.40399
10.02	-4106.24860	-4108.31989	-4108.78753	-4108.61855	-4109.09018	-4108.99811	-4109.45773	-4109.86737	-4110.40385
10.11	-4106.24852	-4108.31975	-4108.78737	-4108.61846	-4109.09006	-4108.99802	-4109.45764	-4109.86734	-4110.40370
10.20	-4106.24844	-4108.31961	-4108.78723	-4108.61838	-4109.08994	-4108.99794	-4109.45755	-4109.86731	-4110.40357
10.30	-4106.24837	-4108.31951	-4108.78711	-4108.61833	-4109.08985	-4108.99788	-4109.45749	-4109.86729	-4110.40344
10.39	-4106.24831	-4108.31942	-4108.78701	-4108.61828	-4109.08979	-4108.99785	-4109.45744	-4109.86730	-4110.40332
10.49	-4106.24825	-4108.31933	-4108.78692	-4108.61823	-4109.08971	-4108.99780	-4109.45739	-4109.86729	-4110.40322
10.58	-4106.24820	-4108.31925	-4108.78683	-4108.61818	-4109.08965	-4108.99776	-4109.45734	-4109.86729	-4110.40312
10.68	-4106.24815	-4108.31918	-4108.78674	-4108.61814	-4109.08959	-4108.99773	-4109.45730	-4109.86729	-4110.40301
10.77	-4106.24810	-4108.31911	-4108.78667	-4108.61811	-4109.08954	-4108.99770	-4109.45727	-4109.86730	-4110.40291
10.87	-4106.24806	-4108.31906	-4108.78661	-4108.61809	-4109.08950	-4108.99768	-4109.45724	-4109.86731	-4110.40284
10.96	-4106.24802	-4108.31900	-4108.78655	-4108.61806	-4109.08946	-4108.99767	-4109.45722	-4109.86732	-4110.40280
11.05	-4106.24798	-4108.31895	-4108.78649	-4108.61803	-4109.08942	-4108.99765	-4109.45720	-4109.86732	-4110.40276
11.15	-4106.24795	-4108.31891	-4108.78643	-4108.61801	-4109.08939	-4108.99763	-4109.45718	-4109.86733	-4110.40270
11.24	-4106.24792	-4108.31887	-4108.78639	-4108.61799	-4109.08936	-4108.99762	-4109.45716	-4109.86734	-4110.40263
11.34	-4106.24789	-4108.31883	-4108.78634	-4108.61798	-4109.08933	-4108.99761	-4109.45714	-4109.86734	-4110.40255
11.43	-4106.24786	-4108.31879	-4108.78630	-4108.61796	-4109.08931	-4108.99760	-4109.45713	-4109.86735	-4110.40250
11.53	-4106.24783	-4108.31876	-4108.78626	-4108.61795	-4109.08928	-4108.99760	-4109.45712	-4109.86736	-4110.40245
11.62	-4106.24781	-4108.31873	-4108.78623	-4108.61794	-4109.08926	-4108.99759	-4109.45711	-4109.86736	-4110.40237
11.72	-4106.24779	-4108.31871	-4108.78620	-4108.61793	-4109.08925	-4108.99759	-4109.45710	-4109.86738	-4110.40231
11.81	-4106.24777	-4108.31869	-4108.78618	-4108.61793	-4109.08923	-4108.99759	-4109.45710	-4109.86739	-4110.40226
11.91	-4106.24775	-4108.31867	-4108.78615	-4108.61792	-4109.08922	-4108.99760	-4109.45710	-4109.86740	-4110.40224
12.00	-4106.24773	-4108.31865	-4108.78613	-4108.61792	-4109.08921	-4108.99760	-4109.45710	-4109.86741	-4110.40223
12.09	-4106.24771	-4108.31863	-4108.78611	-4108.61791	-4109.08920	-4108.99761	-4109.45710	-4109.86742	-4110.40221
12.19	-4106.24770	-4108.31862	-4108.78610	-4108.61791	-4109.08919	-4108.99761	-4109.45711	-4109.86742	-4110.40218
12.28	-4106.24768	-4108.31860	-4108.78608	-4108.61790	-4109.08918	-4108.99762	-4109.45711	-4109.86743	-4110.40216
12.38	-4106.24767	-4108.31859	-4108.78606	-4108.61790	-4109.08917	-4108.99762	-4109.45711	-4109.86744	-4110.40214
12.47	-4106.24765	-4108.31858	-4108.78604	-4108.61789	-4109.08916	-4108.99762	-4109.45711	-4109.86744	-4110.40213
12.57	-4106.24764	-4108.31856	-4108.78603	-4108.61789	-4109.08915	-4108.99763	-4109.45711	-4109.86744	-4110.40212
12.66	-4106.24763	-4108.31855	-4108.78601	-4108.61789	-4109.08914	-4108.99763	-4109.45711	-4109.86745	-4110.40211
12.76	-4106.24762	-4108.31854	-4108.78600	-4108.61788	-4109.08914	-4108.99764	-4109.45711	-4109.86745	-4110.40208
12.85	-4106.24761	-4108.31853	-4108.78599	-4108.61788	-4109.08913	-4108.99764	-4109.45711	-4109.86745	-4110.40204
12.94	-4106.24760	-4108.31852	-4108.78598	-4108.61788	-4109.08912	-4108.99765	-4109.45712	-4109.86746	-4110.40201
13.04	-4106.24759	-4108.31852	-4108.78597	-4108.61788	-4109.08912	-4108.99765	-4109.45712	-4109.86746	-4110.40199
13.13	-4106.24758	-4108.31851	-4108.78597	-4108.61788	-4109.08912	-4108.99766	-4109.45713	-4109.86747	-4110.40197
13.23	-4106.24757	-4108.31851	-4108.78596	-4108.61788	-4109.08911	-4108.99767	-4109.45713	-4109.86747	-4110.40196
13.32	-4106.24756	-4108.31851	-4108.78595	-4108.61788	-4109.08911	-4108.99768	-4109.45714	-4109.86748	-4110.40195

Table S16: The absolute energies in hartree of the potential energy curve of NbSi in the $^4\Pi$ state with a SP-based wave function and nom-CPO active space.

Distance	SP	tpbe	ftpbe	trevpbe	ftrevpbe	tblyp	ftblyp	torelyp	ftorelyp
13.42	-4106.24755	-4108.31850	-4108.78595	-4108.61788	-4109.08911	-4108.99769	-4109.45715	-4109.86748	-4110.40195
13.51	-4106.24755	-4108.31850	-4108.78595	-4108.61788	-4109.08911	-4108.99770	-4109.45716	-4109.86749	-4110.40194
13.61	-4106.24754	-4108.31850	-4108.78594	-4108.61788	-4109.08911	-4108.99770	-4109.45716	-4109.86749	-4110.40193
13.70	-4106.24753	-4108.31849	-4108.78594	-4108.61788	-4109.08911	-4108.99771	-4109.45717	-4109.86749	-4110.40193
13.80	-4106.24753	-4108.31849	-4108.78593	-4108.61788	-4109.08910	-4108.99772	-4109.45717	-4109.86750	-4110.40192
13.89	-4106.24752	-4108.31849	-4108.78593	-4108.61788	-4109.08910	-4108.99772	-4109.45718	-4109.86750	-4110.40192
13.98	-4106.24751	-4108.31848	-4108.78592	-4108.61788	-4109.08910	-4108.99773	-4109.45718	-4109.86750	-4110.40191
14.08	-4106.24751	-4108.31848	-4108.78592	-4108.61788	-4109.08909	-4108.99773	-4109.45718	-4109.86750	-4110.40190
14.17	-4106.24750	-4108.31848	-4108.78591	-4108.61788	-4109.08909	-4108.99773	-4109.45719	-4109.86750	-4110.40190
14.27	-4106.24750	-4108.31847	-4108.78591	-4108.61787	-4109.08909	-4108.99774	-4109.45719	-4109.86750	-4110.40189
14.36	-4106.24749	-4108.31847	-4108.78591	-4108.61787	-4109.08909	-4108.99774	-4109.45719	-4109.86750	-4110.40188
14.46	-4106.24749	-4108.31847	-4108.78590	-4108.61787	-4109.08908	-4108.99775	-4109.45720	-4109.86750	-4110.40187
14.55	-4106.24748	-4108.31847	-4108.78590	-4108.61787	-4109.08908	-4108.99775	-4109.45720	-4109.86750	-4110.40187
14.65	-4106.24748	-4108.31846	-4108.78590	-4108.61787	-4109.08908	-4108.99776	-4109.45721	-4109.86750	-4110.40186
14.74	-4106.24748	-4108.31846	-4108.78590	-4108.61787	-4109.08908	-4108.99776	-4109.45721	-4109.86750	-4110.40185
14.83	-4106.24747	-4108.31846	-4108.78590	-4108.61787	-4109.08908	-4108.99777	-4109.45721	-4109.86750	-4110.40185
14.93	-4106.24747	-4108.31846	-4108.78590	-4108.61787	-4109.08908	-4108.99777	-4109.45722	-4109.86751	-4110.40185
15.02	-4106.24746	-4108.31846	-4108.78590	-4108.61787	-4109.08908	-4108.99778	-4109.45722	-4109.86751	-4110.40184
15.12	-4106.24746	-4108.31846	-4108.78589	-4108.61788	-4109.08908	-4108.99778	-4109.45723	-4109.86751	-4110.40184
15.21	-4106.24746	-4108.31846	-4108.78589	-4108.61788	-4109.08908	-4108.99778	-4109.45723	-4109.86751	-4110.40184
15.31	-4106.24746	-4108.31846	-4108.78589	-4108.61788	-4109.08908	-4108.99779	-4109.45723	-4109.86751	-4110.40184
15.40	-4106.24745	-4108.31846	-4108.78589	-4108.61788	-4109.08908	-4108.99779	-4109.45724	-4109.86751	-4110.40184
15.50	-4106.24745	-4108.31846	-4108.78589	-4108.61788	-4109.08908	-4108.99779	-4109.45724	-4109.86751	-4110.40183
15.59	-4106.24745	-4108.31846	-4108.78589	-4108.61787	-4109.08908	-4108.99780	-4109.45724	-4109.86751	-4110.40183
15.68	-4106.24744	-4108.31846	-4108.78589	-4108.61787	-4109.08908	-4108.99780	-4109.45724	-4109.86751	-4110.40183
15.78	-4106.24744	-4108.31846	-4108.78589	-4108.61787	-4109.08907	-4108.99780	-4109.45725	-4109.86751	-4110.40183
15.87	-4106.24744	-4108.31846	-4108.78589	-4108.61787	-4109.08907	-4108.99780	-4109.45725	-4109.86751	-4110.40183
15.97	-4106.24744	-4108.31845	-4108.78589	-4108.61787	-4109.08907	-4108.99781	-4109.45725	-4109.86751	-4110.40182
16.06	-4106.24743	-4108.31845	-4108.78588	-4108.61787	-4109.08907	-4108.99781	-4109.45725	-4109.86751	-4110.40182
16.16	-4106.24743	-4108.31845	-4108.78588	-4108.61787	-4109.08907	-4108.99781	-4109.45725	-4109.86751	-4110.40182
16.25	-4106.24743	-4108.31845	-4108.78588	-4108.61787	-4109.08907	-4108.99781	-4109.45725	-4109.86751	-4110.40182
16.35	-4106.24743	-4108.31845	-4108.78588	-4108.61787	-4109.08907	-4108.99781	-4109.45726	-4109.86751	-4110.40182
16.44	-4106.24743	-4108.31845	-4108.78588	-4108.61787	-4109.08907	-4108.99782	-4109.45726	-4109.86751	-4110.40181
16.54	-4106.24742	-4108.31845	-4108.78588	-4108.61787	-4109.08907	-4108.99782	-4109.45726	-4109.86751	-4110.40181
16.63	-4106.24742	-4108.31845	-4108.78588	-4108.61787	-4109.08907	-4108.99782	-4109.45726	-4109.86751	-4110.40181
16.72	-4106.24742	-4108.31845	-4108.78588	-4108.61787	-4109.08907	-4108.99782	-4109.45726	-4109.86751	-4110.40180
16.82	-4106.24742	-4108.31845	-4108.78588	-4108.61787	-4109.08907	-4108.99782	-4109.45726	-4109.86751	-4110.40181
16.91	-4106.24742	-4108.31845	-4108.78588	-4108.61787	-4109.08907	-4108.99783	-4109.45727	-4109.86751	-4110.40181
17.01	-4106.24742	-4108.31845	-4108.78588	-4108.61787	-4109.08907	-4108.99783	-4109.45727	-4109.86751	-4110.40181
17.10	-4106.24742	-4108.31845	-4108.78588	-4108.61787	-4109.08907	-4108.99783	-4109.45727	-4109.86752	-4110.40180
17.20	-4106.24741	-4108.31845	-4108.78588	-4108.61787	-4109.08907	-4108.99783	-4109.45727	-4109.86752	-4110.40180
17.29	-4106.24741	-4108.31846	-4108.78588	-4108.61788	-4109.08907	-4108.99784	-4109.45727	-4109.86752	-4110.40180
17.39	-4106.24741	-4108.31846	-4108.78588	-4108.61788	-4109.08907	-4108.99784	-4109.45728	-4109.86752	-4110.40180
17.48	-4106.24741	-4108.31846	-4108.78588	-4108.61788	-4109.08907	-4108.99784	-4109.45728	-4109.86752	-4110.40180
17.57	-4106.24741	-4108.31846	-4108.78588	-4108.61788	-4109.08907	-4108.99784	-4109.45728	-4109.86752	-4110.40180
17.67	-4106.24741	-4108.31846	-4108.78588	-4108.61788	-4109.08907	-4108.99784	-4109.45728	-4109.86752	-4110.40180
17.76	-4106.24741	-4108.31846	-4108.78588	-4108.61787	-4109.08907	-4108.99784	-4109.45728	-4109.86752	-4110.40180
17.86	-4106.24741	-4108.31846	-4108.78588	-4108.61787	-4109.08907	-4108.99784	-4109.45728	-4109.86752	-4110.40180
17.95	-4106.24740	-4108.31845	-4108.78588	-4108.61787	-4109.08907	-4108.99784	-4109.45728	-4109.86752	-4110.40180
18.05	-4106.24740	-4108.31845	-4108.78588	-4108.61787	-4109.08907	-4108.99784	-4109.45728	-4109.86752	-4110.40180

Table S17: The absolute energies in hartree of the potential energy curve of NbSi in the $^2\Delta$ state with a SP-based wave function and nom-CPO active space.

Distance	SP	tpbe	ftpbe	trevpbe	ftrevpbe	tblyp	ftblyp	torelyp	ftorelyp
1.98	-4102.60236	-4104.80299	-4105.27431	-4105.08228	-4105.55746	-4105.44185	-4105.90594	-4106.37105	-4106.88759
2.08	-4103.26773	-4105.46058	-4105.93187	-4105.74088	-4106.21619	-4106.10097	-4106.56478	-4107.02520	-4107.54194
2.17	-4103.80451	-4105.99007	-4106.46150	-4106.27136	-4106.74699	-4106.63191	-4107.09562	-4107.55153	-4108.06865
2.27	-4104.23660	-4106.41587	-4106.88759	-4106.69811	-4107.17421	-4107.05906	-4107.52286	-4107.97445	-4108.49211
2.36	-4104.58448	-4106.75872	-4107.23082	-4107.04186	-4107.51852	-4107.40320	-4107.86721	-4108.31473	-4108.83303
2.46	-4104.86521	-4107.03563	-4107.50813	-4107.31960	-4107.79682	-4107.68131	-4108.14560	-4108.58934	-4109.10828
2.55	-4105.09276	-4107.26057	-4107.73341	-4107.54533	-4108.02300	-4107.90739	-4108.37193	-4108.81229	-4109.33179
2.65	-4105.31671	-4107.44457	-4107.91763	-4107.73005	-4108.20802	-4108.09246	-4108.55718	-4108.99458	-4109.51446
2.74	-4105.47744	-4107.59634	-4108.06948	-4107.88250	-4108.36057	-4108.24523	-4108.71001	-4109.14493	-4109.66497
2.83	-4105.61381	-4107.72260	-4108.19566	-4108.00939	-4108.48734	-4108.37242	-4108.83710	-4109.27000	-4109.79001
2.93	-4105.73037	-4107.82827	-4108.30113	-4108.11567	-4108.59334	-4108.47889	-4108.94339	-4109.37472	-4109.89450
3.02	-4105.83126	-4107.91706	-4108.38966	-4108.20506	-4108.68236	-4108.56837	-4109.03261	-4109.46270	-4109.98218
3.12	-4105.91740	-4107.99181	-4108.46415	-4108.28038	-4108.75732	-4108.64370	-4109.10771	-4109.53667	-4110.05593
3.21	-4105.99082	-4108.05478	-4108.52693	-4108.34390	-4108.82056	-4108.70717	-4109.17099	-4109.59882	-4110.11799
3.31	-4106.05326	-4108.19089	-4108.65954	-4108.47975	-4108.95382	-4108.84310	-4109.30142	-4109.73213	-4110.24972
3.40	-4106.10620	-4108.24169	-4108.70978	-4108.53084	-4109.00440	-4108.89451	-4109.35223	-4109.78182	-4110.29888
3.50	-4106.15091	-4108.28437	-4108.75196	-4108.57382	-4109.04691	-4108.93782	-4109.39499	-4109.82358	-4110.34014
3.59	-4106.18848	-4108.32001	-4108.78715	-4108.60976	-4109.08241	-4108.97409	-4109.43079	-4109.85845	-4110.37454
3.68	-4106.21986	-4108.34960	-4108.81634	-4108.63964	-4109.11189	-4109.00431	-4109.46059	-4109.88740	-4110.40306
3.78	-4106.24588	-4108.37399	-4108.84038	-4108.66433	-4109.13622	-4109.02932	-4109.48524	-4109.91126	-4110.42653
3.87	-4106.26725	-4108.39387	-4108.85994	-4108.68449	-4109.15605	-4109.04982	-4109.50541	-4109.93069	-4110.44559
3.97	-4106.28462	-4108.40985	-4108.87563	-4108.70075	-4109.17200	-4109.06641	-4109.52171	-4109.94630	-4110.46088
4.06	-4106.29854	-4108.42252	-4108.88804	-4108.71368	-4109.18466	-4109.07967	-4109.53471	-4109.95868	-4110.47294
4.16	-4106.30948	-4108.43236	-4108.89765	-4108.72378	-4109.19452	-4109.09010	-4109.54492	-4109.96828	-4110.48226
4.35	-4106.31789	-4108.43977	-4108.90486	-4108.73143	-4109.20195	-4109.09809	-4109.55271	-4109.97551	-4110.48923
4.44	-4106.32412	-4108.44511	-4108.91002	-4108.73700	-4109.20732	-4109.10399	-4109.55843	-4109.98072	-4110.49420
4.54	-4106.32850	-4108.44871	-4108.91345	-4108.74082	-4109.21096	-4109.10814	-4109.56242	-4109.98423	-4110.49748
4.63	-4106.33132	-4108.45085	-4108.91545	-4108.74318	-4109.21316	-4109.11083	-4109.56497	-4109.98632	-4110.49937
4.72	-4106.33282	-4108.45178	-4108.91626	-4108.74431	-4109.21415	-4109.11230	-4109.56631	-4109.98724	-4110.50010
4.82	-4106.33322	-4108.45169	-4108.91606	-4108.74441	-4109.21413	-4109.11272	-4109.56663	-4109.98717	-4110.49986
4.91	-4106.33271	-4108.45075	-4108.91503	-4108.74365	-4109.21326	-4109.11229	-4109.56611	-4109.98627	-4110.49881
5.01	-4106.33144	-4108.44911	-4108.91332	-4108.74219	-4109.21171	-4109.11116	-4109.56490	-4109.98471	-4110.49711
5.10	-4106.32955	-4108.44694	-4108.91108	-4108.74018	-4109.20963	-4109.10948	-4109.56315	-4109.98264	-4110.49490
5.20	-4106.32717	-4108.44434	-4108.90843	-4108.73774	-4109.20713	-4109.10736	-4109.56097	-4109.98016	-4110.49231
5.29	-4106.32439	-4108.44140	-4108.90545	-4108.73496	-4109.20430	-4109.10489	-4109.55845	-4109.97736	-4110.48941
5.39	-4106.32131	-4108.43820	-4108.90222	-4108.73189	-4109.20120	-4109.10215	-4109.55566	-4109.97431	-4110.48628
5.48	-4106.31800	-4108.43480	-4108.89881	-4108.72864	-4109.19793	-4109.09921	-4109.55269	-4109.97108	-4110.48299
5.57	-4106.31452	-4108.43130	-4108.89530	-4108.72526	-4109.19454	-4109.09614	-4109.54959	-4109.96777	-4110.47961
5.67	-4106.31094	-4108.42774	-4108.89175	-4108.72183	-4109.19111	-4109.09302	-4109.54643	-4109.96441	-4110.47621
5.76	-4106.30729	-4108.42417	-4108.88818	-4108.71839	-4109.18767	-4109.08987	-4109.54325	-4109.96106	-4110.47282
5.86	-4106.30362	-4108.42065	-4108.88466	-4108.71497	-4109.18427	-4109.08676	-4109.54010	-4109.95777	-4110.46949
5.95	-4106.29996	-4108.41716	-4108.88118	-4108.71159	-4109.18090	-4109.08368	-4109.53697	-4109.95453	-4110.46622
6.05	-4106.29636	-4108.41375	-4108.87778	-4108.70827	-4109.17761	-4109.08066	-4109.53389	-4109.95138	-4110.46304
6.14	-4106.29283	-4108.41043	-4108.87446	-4108.70504	-4109.17440	-4109.07772	-4109.53086	-4109.94833	-4110.45996
6.24	-4106.28941	-4108.40721	-4108.87122	-4108.70189	-4109.17127	-4109.07486	-4109.52788	-4109.94538	-4110.45697
6.33	-4106.28613	-4108.40404	-4108.86803	-4108.69879	-4109.16819	-4109.07205	-4109.52492	-4109.94248	-4110.45402
6.43	-4106.28301	-4108.40091	-4108.86487	-4108.69571	-4109.16514	-4109.06924	-4109.52193	-4109.93958	-4110.45107
6.52	-4106.28009	-4108.39780	-4108.86171	-4108.69264	-4109.16211	-4109.06641	-4109.51889	-4109.93666	-4110.44809
6.61	-4106.27737	-4108.39469	-4108.85856	-4108.68957	-4109.15908	-4109.06355	-4109.51580	-4109.93370	-4110.44506
6.71	-4106.27488	-4108.39160	-4108.85542	-4108.68651	-4109.15607	-4109.06067	-4109.51266	-4109.93069	-4110.44199
6.80	-4106.27263	-4108.38856	-4108.85230	-4108.68349	-4109.15308	-4109.05780	-4109.50949	-4109.92767	-4110.43888
6.90	-4106.27062	-4108.38559	-4108.84926	-4108.68054	-4109.15017	-4109.05497	-4109.50636	-4109.92467	-4110.43578

Table S17: The absolute energies in hartree of the potential energy curve of NbSi in the $^2\Delta$ state with a SP-based wave function and nom-CPO active space.

Distance	SP	tpbe	ftpbe	trevpbe	ftrevpbe	tblyp	ftblyp	torelyp	ftorelyp
6.99	-4106.26884	-4108.38274	-4108.84631	-4108.67770	-4109.14736	-4109.05221	-4109.50329	-4109.92172	-4110.43273
7.09	-4106.26730	-4108.38002	-4108.84348	-4108.67500	-4109.14467	-4109.04956	-4109.50032	-4109.91889	-4110.42978
7.18	-4106.26597	-4108.37750	-4108.84081	-4108.67249	-4109.14211	-4109.04709	-4109.49749	-4109.91623	-4110.42697
7.28	-4106.26483	-4108.37519	-4108.83833	-4108.67021	-4109.13975	-4109.04481	-4109.49487	-4109.91378	-4110.42435
7.37	-4106.26665	-4108.37311	-4108.83610	-4108.66816	-4109.13762	-4109.04277	-4109.49250	-4109.91159	-4110.42196
7.46	-4106.26604	-4108.37128	-4108.83411	-4108.66637	-4109.13573	-4109.04096	-4109.49040	-4109.90965	-4110.41984
7.56	-4106.26551	-4108.36968	-4108.83235	-4108.66481	-4109.13406	-4109.03940	-4109.48853	-4109.90509	-4110.41653
7.65	-4106.26506	-4108.36417	-4108.82850	-4108.66022	-4109.13115	-4109.03335	-4109.48476	-4109.90393	-4110.41518
7.75	-4106.26467	-4108.36323	-4108.82741	-4108.65933	-4109.13013	-4109.03247	-4109.48363	-4109.90293	-4110.41400
7.84	-4106.26434	-4108.36241	-4108.82646	-4108.65855	-4109.12924	-4109.03170	-4109.48263	-4109.90207	-4110.41296
7.94	-4106.26405	-4108.36170	-4108.82562	-4108.65788	-4109.12846	-4109.03103	-4109.48176	-4109.90132	-4110.41205
8.03	-4106.26381	-4108.36108	-4108.82487	-4108.65730	-4109.12777	-4109.03045	-4109.48098	-4109.90068	-4110.41124
8.13	-4106.26360	-4108.36053	-4108.82420	-4108.65679	-4109.12715	-4109.02995	-4109.48029	-4109.90013	-4110.41052
8.22	-4106.26342	-4108.36005	-4108.82360	-4108.65635	-4109.12660	-4109.02952	-4109.47968	-4109.89966	-4110.40989
8.31	-4106.26326	-4108.35964	-4108.82307	-4108.65598	-4109.12613	-4109.02915	-4109.47914	-4109.89925	-4110.40934
8.41	-4106.26312	-4108.35928	-4108.82262	-4108.65566	-4109.12571	-4109.02883	-4109.47868	-4109.89891	-4110.40887
8.50	-4106.26301	-4108.35897	-4108.82222	-4108.65539	-4109.12536	-4109.02856	-4109.47828	-4109.89863	-4110.40846
8.60	-4106.26291	-4108.35870	-4108.82187	-4108.65516	-4109.12505	-4109.02834	-4109.47793	-4109.89839	-4110.40811
8.69	-4106.26282	-4108.35846	-4108.82157	-4108.65496	-4109.12479	-4109.02814	-4109.47762	-4109.89819	-4110.40780
8.79	-4106.26275	-4108.35825	-4108.82130	-4108.65479	-4109.12456	-4109.02797	-4109.47736	-4109.89802	-4110.40753
8.88	-4106.26268	-4108.35807	-4108.82105	-4108.65464	-4109.12435	-4109.02783	-4109.47712	-4109.89787	-4110.40730
8.98	-4106.26262	-4108.35791	-4108.82084	-4108.65452	-4109.12417	-4109.02771	-4109.47691	-4109.89776	-4110.40709
9.07	-4106.26257	-4108.35777	-4108.82064	-4108.65441	-4109.12400	-4109.02760	-4109.47672	-4109.89766	-4110.40690
9.17	-4106.26253	-4108.35765	-4108.82046	-4108.65432	-4109.12385	-4109.02751	-4109.47655	-4109.89758	-4110.40674
9.26	-4106.26249	-4108.35754	-4108.82030	-4108.65424	-4109.12372	-4109.02744	-4109.47639	-4109.89751	-4110.40659
9.35	-4106.26246	-4108.35745	-4108.82015	-4108.65418	-4109.12361	-4109.02738	-4109.47626	-4109.89747	-4110.40647
9.45	-4106.26243	-4108.35737	-4108.82003	-4108.65413	-4109.12351	-4109.02734	-4109.47615	-4109.89743	-4110.40637
9.54	-4106.26240	-4108.35730	-4108.81992	-4108.65409	-4109.12343	-4109.02730	-4109.47605	-4109.89741	-4110.40628
9.64	-4106.26238	-4108.35724	-4108.81983	-4108.65406	-4109.12336	-4109.02727	-4109.47596	-4109.89739	-4110.40620
9.73	-4106.26236	-4108.35719	-4108.81974	-4108.65404	-4109.12330	-4109.02724	-4109.47589	-4109.89738	-4110.40614
9.83	-4106.26234	-4108.35714	-4108.81967	-4108.65402	-4109.12325	-4109.02722	-4109.47583	-4109.89738	-4110.40608
9.92	-4106.26233	-4108.35710	-4108.81960	-4108.65400	-4109.12321	-4109.02721	-4109.47577	-4109.89738	-4110.40603
10.02	-4106.26231	-4108.35707	-4108.81953	-4108.65399	-4109.12316	-4109.02720	-4109.47571	-4109.89738	-4110.40598
10.11	-4106.26230	-4108.35704	-4108.81947	-4108.65398	-4109.12312	-4109.02719	-4109.47566	-4109.89739	-4110.40594
10.20	-4106.26229	-4108.35701	-4108.81941	-4108.65398	-4109.12308	-4109.02718	-4109.47562	-4109.89740	-4110.40590
10.30	-4106.26228	-4108.35698	-4108.81936	-4108.65398	-4109.12305	-4109.02718	-4109.47557	-4109.89742	-4110.40587
10.39	-4106.26227	-4108.35697	-4108.81931	-4108.65398	-4109.12302	-4109.02718	-4109.47554	-4109.89744	-4110.40584
10.49	-4106.26226	-4108.35695	-4108.81927	-4108.65399	-4109.12300	-4109.02719	-4109.47550	-4109.89746	-4110.40582
10.58	-4106.26225	-4108.35694	-4108.81924	-4108.65399	-4109.12298	-4109.02720	-4109.47548	-4109.89748	-4110.40580
10.68	-4106.26225	-4108.35693	-4108.81921	-4108.65400	-4109.12296	-4109.02720	-4109.47546	-4109.89750	-4110.40579
10.77	-4106.26224	-4108.35692	-4108.81918	-4108.65401	-4109.12295	-4109.02721	-4109.47544	-4109.89753	-4110.40578
10.87	-4106.26223	-4108.35691	-4108.81916	-4108.65402	-4109.12295	-4109.02722	-4109.47543	-4109.89755	-4110.40577
10.96	-4106.26223	-4108.35690	-4108.81914	-4108.65403	-4109.12294	-4109.02723	-4109.47542	-4109.89757	-4110.40577
11.05	-4106.26222	-4108.35690	-4108.81912	-4108.65404	-4109.12293	-4109.02724	-4109.47541	-4109.89759	-4110.40576
11.15	-4106.26222	-4108.35689	-4108.81910	-4108.65405	-4109.12292	-4109.02725	-4109.47540	-4109.89761	-4110.40575
11.24	-4106.26221	-4108.35689	-4108.81908	-4108.65406	-4109.12291	-4109.02726	-4109.47539	-4109.89763	-4110.40574
11.34	-4106.26221	-4108.35688	-4108.81906	-4108.65407	-4109.12291	-4109.02727	-4109.47538	-4109.89765	-4110.40574
11.43	-4106.26220	-4108.35688	-4108.81904	-4108.65408	-4109.12290	-4109.02728	-4109.47536	-4109.89767	-4110.40573
11.53	-4106.26220	-4108.35687	-4108.81902	-4108.65409	-4109.12289	-4109.02729	-4109.47535	-4109.89769	-4110.40572
11.62	-4106.26220	-4108.35687	-4108.81901	-4108.65410	-4109.12288	-4109.02730	-4109.47535	-4109.89771	-4110.40571
11.72	-4106.26219	-4108.35687	-4108.81900	-4108.65411	-4109.12288	-4109.02732	-4109.47534	-4109.89773	-4110.40571
11.81	-4106.26219	-4108.35687	-4108.81899	-4108.65412	-4109.12288	-4109.02733	-4109.47534	-4109.89775	-4110.40570

Table S17: The absolute energies in hartree of the potential energy curve of NbSi in the $^2\Delta$ state with a SP-based wave function and nom-CPO active space.

Distance	SP	tpbe	ftpbe	trevpbe	ftrevpbe	tblyp	ftblyp	torelyp	ftorelyp
11.91	-4106.26218	-4108.35687	-4108.81898	-4108.65413	-4109.12288	-4109.02734	-4109.47534	-4109.89776	-4110.40570
12.00	-4106.26218	-4108.35687	-4108.81897	-4108.65414	-4109.12288	-4109.02735	-4109.47534	-4109.89778	-4110.40571
12.09	-4106.26218	-4108.35687	-4108.81896	-4108.65415	-4109.12288	-4109.02736	-4109.47534	-4109.89780	-4110.40571
12.19	-4106.26218	-4108.35687	-4108.81896	-4108.65416	-4109.12288	-4109.02737	-4109.47534	-4109.89781	-4110.40571
12.28	-4106.26217	-4108.35687	-4108.81895	-4108.65417	-4109.12288	-4109.02738	-4109.47534	-4109.89783	-4110.40571
12.38	-4106.26217	-4108.35687	-4108.81894	-4108.65417	-4109.12287	-4109.02739	-4109.47534	-4109.89784	-4110.40570
12.47	-4106.26217	-4108.35687	-4108.81893	-4108.65418	-4109.12287	-4109.02740	-4109.47534	-4109.89785	-4110.40570
12.57	-4106.26217	-4108.35686	-4108.81892	-4108.65418	-4109.12287	-4109.02741	-4109.47534	-4109.89786	-4110.40570
12.66	-4106.26216	-4108.35686	-4108.81892	-4108.65419	-4109.12287	-4109.02742	-4109.47533	-4109.89787	-4110.40569
12.76	-4106.26216	-4108.35686	-4108.81891	-4108.65419	-4109.12286	-4109.02742	-4109.47533	-4109.89787	-4110.40569
12.85	-4106.26216	-4108.35686	-4108.81891	-4108.65420	-4109.12286	-4109.02743	-4109.47533	-4109.89788	-4110.40569
12.94	-4106.26216	-4108.35686	-4108.81890	-4108.65420	-4109.12286	-4109.02744	-4109.47533	-4109.89789	-4110.40568
13.04	-4106.26216	-4108.35686	-4108.81889	-4108.65421	-4109.12286	-4109.02744	-4109.47533	-4109.89790	-4110.40568
13.13	-4106.26215	-4108.35686	-4108.81889	-4108.65421	-4109.12286	-4109.02745	-4109.47533	-4109.89790	-4110.40567
13.23	-4106.26215	-4108.35686	-4108.81889	-4108.65422	-4109.12286	-4109.02746	-4109.47533	-4109.89791	-4110.40567
13.32	-4106.26215	-4108.35686	-4108.81888	-4108.65422	-4109.12286	-4109.02747	-4109.47533	-4109.89792	-4110.40567
13.42	-4106.26215	-4108.35686	-4108.81888	-4108.65423	-4109.12286	-4109.02747	-4109.47534	-4109.89792	-4110.40567
13.51	-4106.26215	-4108.35686	-4108.81888	-4108.65423	-4109.12286	-4109.02748	-4109.47534	-4109.89793	-4110.40567
13.61	-4106.26214	-4108.35686	-4108.81888	-4108.65424	-4109.12286	-4109.02749	-4109.47534	-4109.89793	-4110.40567
13.70	-4106.26214	-4108.35686	-4108.81887	-4108.65424	-4109.12286	-4109.02749	-4109.47534	-4109.89794	-4110.40567
13.80	-4106.26214	-4108.35686	-4108.81887	-4108.65424	-4109.12285	-4109.02750	-4109.47534	-4109.89794	-4110.40567
13.89	-4106.26214	-4108.35686	-4108.81886	-4108.65424	-4109.12285	-4109.02750	-4109.47534	-4109.89795	-4110.40567
13.98	-4106.26214	-4108.35685	-4108.81886	-4108.65424	-4109.12285	-4109.02751	-4109.47534	-4109.89795	-4110.40566
14.08	-4106.26214	-4108.35685	-4108.81886	-4108.65424	-4109.12285	-4109.02751	-4109.47534	-4109.89795	-4110.40566
14.17	-4106.26214	-4108.35685	-4108.81885	-4108.65424	-4109.12285	-4109.02751	-4109.47534	-4109.89795	-4110.40566
14.27	-4106.26214	-4108.35685	-4108.81885	-4108.65424	-4109.12284	-4109.02752	-4109.47534	-4109.89795	-4110.40566
14.36	-4106.26213	-4108.35685	-4108.81885	-4108.65425	-4109.12284	-4109.02752	-4109.47535	-4109.89795	-4110.40565
14.46	-4106.26213	-4108.35685	-4108.81885	-4108.65425	-4109.12284	-4109.02752	-4109.47535	-4109.89795	-4110.40565
14.55	-4106.26213	-4108.35685	-4108.81884	-4108.65425	-4109.12284	-4109.02753	-4109.47535	-4109.89795	-4110.40565
14.65	-4106.26213	-4108.35685	-4108.81884	-4108.65425	-4109.12284	-4109.02753	-4109.47535	-4109.89795	-4110.40565
14.74	-4106.26213	-4108.35684	-4108.81884	-4108.65425	-4109.12284	-4109.02753	-4109.47535	-4109.89795	-4110.40564
14.83	-4106.26213	-4108.35684	-4108.81884	-4108.65425	-4109.12284	-4109.02754	-4109.47535	-4109.89796	-4110.40564
14.93	-4106.26213	-4108.35684	-4108.81884	-4108.65425	-4109.12284	-4109.02754	-4109.47535	-4109.89796	-4110.40564
15.02	-4106.26213	-4108.35684	-4108.81884	-4108.65425	-4109.12284	-4109.02754	-4109.47535	-4109.89796	-4110.40564
15.12	-4106.26213	-4108.35684	-4108.81884	-4108.65425	-4109.12284	-4109.02755	-4109.47535	-4109.89796	-4110.40564
15.21	-4106.26213	-4108.35684	-4108.81883	-4108.65425	-4109.12284	-4109.02755	-4109.47535	-4109.89796	-4110.40564
15.31	-4106.26213	-4108.35684	-4108.81883	-4108.65425	-4109.12284	-4109.02755	-4109.47536	-4109.89796	-4110.40564
15.40	-4106.26212	-4108.35684	-4108.81883	-4108.65425	-4109.12283	-4109.02755	-4109.47536	-4109.89796	-4110.40564
15.50	-4106.26212	-4108.35684	-4108.81883	-4108.65425	-4109.12283	-4109.02755	-4109.47536	-4109.89796	-4110.40563
15.59	-4106.26212	-4108.35684	-4108.81883	-4108.65425	-4109.12283	-4109.02756	-4109.47536	-4109.89796	-4110.40563
15.68	-4106.26212	-4108.35684	-4108.81882	-4108.65425	-4109.12283	-4109.02756	-4109.47536	-4109.89796	-4110.40563
15.78	-4106.26212	-4108.35683	-4108.81882	-4108.65425	-4109.12283	-4109.02756	-4109.47536	-4109.89796	-4110.40563
15.87	-4106.26212	-4108.35683	-4108.81882	-4108.65425	-4109.12283	-4109.02756	-4109.47536	-4109.89796	-4110.40563
15.97	-4106.26212	-4108.35683	-4108.81882	-4108.65425	-4109.12283	-4109.02756	-4109.47536	-4109.89796	-4110.40563
16.06	-4106.26212	-4108.35683	-4108.81882	-4108.65425	-4109.12283	-4109.02756	-4109.47536	-4109.89796	-4110.40562
16.16	-4106.26212	-4108.35683	-4108.81882	-4108.65425	-4109.12282	-4109.02756	-4109.47536	-4109.89796	-4110.40562
16.25	-4106.26212	-4108.35683	-4108.81882	-4108.65425	-4109.12282	-4109.02756	-4109.47536	-4109.89795	-4110.40562
16.35	-4106.26212	-4108.35683	-4108.81882	-4108.65425	-4109.12282	-4109.02757	-4109.47536	-4109.89795	-4110.40562
16.44	-4106.26212	-4108.35683	-4108.81882	-4108.65425	-4109.12282	-4109.02757	-4109.47536	-4109.89795	-4110.40562
16.54	-4106.26212	-4108.35683	-4108.81882	-4108.65425	-4109.12282	-4109.02757	-4109.47536	-4109.89795	-4110.40562
16.63	-4106.26212	-4108.35683	-4108.81882	-4108.65425	-4109.12282	-4109.02757	-4109.47536	-4109.89795	-4110.40562
16.72	-4106.26212	-4108.35683	-4108.81881	-4108.65425	-4109.12282	-4109.02757	-4109.47536	-4109.89795	-4110.40562

Table S17: The absolute energies in hartree of the potential energy curve of NbSi in the $^2\Delta$ state with a SP-based wave function and nom-CPO active space.

Distance	SP	tpbe	ftpbe	trevpbe	ftrevpbe	tblyp	ftblyp	torelyp	ftorelyp
16.82	-4106.26212	-4108.35683	-4108.81881	-4108.65425	-4109.12282	-4109.02757	-4109.47536	-4109.89795	-4110.40562
16.91	-4106.26212	-4108.35683	-4108.81881	-4108.65425	-4109.12282	-4109.02757	-4109.47536	-4109.89795	-4110.40562
17.01	-4106.26212	-4108.35683	-4108.81881	-4108.65425	-4109.12282	-4109.02757	-4109.47536	-4109.89795	-4110.40562
17.10	-4106.26212	-4108.35683	-4108.81881	-4108.65425	-4109.12282	-4109.02758	-4109.47536	-4109.89795	-4110.40562
17.20	-4106.26211	-4108.35683	-4108.81881	-4108.65425	-4109.12282	-4109.02758	-4109.47536	-4109.89795	-4110.40562
17.29	-4106.26211	-4108.35683	-4108.81881	-4108.65425	-4109.12282	-4109.02758	-4109.47536	-4109.89795	-4110.40562
17.39	-4106.26211	-4108.35683	-4108.81881	-4108.65425	-4109.12282	-4109.02758	-4109.47536	-4109.89795	-4110.40561
17.48	-4106.26211	-4108.35683	-4108.81881	-4108.65425	-4109.12282	-4109.02758	-4109.47536	-4109.89795	-4110.40561
17.57	-4106.26211	-4108.35683	-4108.81881	-4108.65425	-4109.12282	-4109.02758	-4109.47536	-4109.89795	-4110.40561
17.67	-4106.26211	-4108.35683	-4108.81881	-4108.65425	-4109.12282	-4109.02758	-4109.47536	-4109.89795	-4110.40561
17.76	-4106.26211	-4108.35683	-4108.81881	-4108.65425	-4109.12282	-4109.02758	-4109.47537	-4109.89795	-4110.40561
17.86	-4106.26211	-4108.35683	-4108.81881	-4108.65425	-4109.12282	-4109.02758	-4109.47537	-4109.89795	-4110.40561
17.95	-4106.26211	-4108.35683	-4108.81881	-4108.65425	-4109.12282	-4109.02758	-4109.47537	-4109.89795	-4110.40561
18.05	-4106.26211	-4108.35682	-4108.81881	-4108.65425	-4109.12282	-4109.02758	-4109.47537	-4109.89795	-4110.40561

Table S18: The absolute energies in hartree of the potential energy curve of NbSi in the $^2\Sigma^+$ state with a SP-based wave function and nom-CPO active space.

Distance	SP	tpbe	ftpbe	trevpbe	ftrevpbe	tblyp	ftblyp	torelyp	ftorelyp
1.98	-4102.85155	-4105.05240	-4105.52423	-4105.33477	-4105.80698	-4105.69499	-4106.16714	-4106.62993	-4107.15085
2.08	-4103.51960	-4105.71319	-4106.18451	-4105.99663	-4106.46831	-4106.35744	-4106.82907	-4107.28650	-4107.80689
2.17	-4104.05516	-4106.24210	-4106.71296	-4106.52662	-4106.99780	-4106.88778	-4107.35890	-4107.81157	-4108.33137
2.27	-4104.48288	-4106.66428	-4107.13475	-4106.94985	-4107.42060	-4107.31111	-4107.78176	-4108.23046	-4108.74953
2.36	-4104.82401	-4107.00132	-4107.47155	-4107.28792	-4107.75840	-4107.64887	-4108.11913	-4108.56998	-4109.08528
2.46	-4105.09693	-4107.27061	-4107.74092	-4107.55815	-4108.02871	-4107.91830	-4108.38834	-4108.83914	-4109.35359
2.55	-4105.31838	-4107.49030	-4107.96192	-4107.77865	-4108.25091	-4108.13773	-4108.60832	-4109.06291	-4109.57695
2.65	-4105.49792	-4107.66413	-4108.13529	-4107.95348	-4108.42526	-4108.31204	-4108.78207	-4109.24225	-4109.75597
2.74	-4105.64377	-4107.80536	-4108.27618	-4108.09564	-4108.56705	-4108.45387	-4108.92356	-4109.38673	-4109.90024
2.83	-4105.79800	-4107.94505	-4108.41390	-4108.23435	-4108.70322	-4108.59523	-4109.06350	-4109.50393	-4110.01737
2.93	-4105.89671	-4108.04143	-4108.51030	-4108.33152	-4108.80044	-4108.69213	-4109.16041	-4109.59976	-4110.11325
3.02	-4105.97725	-4108.12078	-4108.58970	-4108.41161	-4108.88063	-4108.77200	-4109.24030	-4109.67870	-4110.19224
3.12	-4106.04300	-4108.18639	-4108.65526	-4108.47792	-4108.94692	-4108.83812	-4109.30632	-4109.74404	-4110.25753
3.21	-4106.09699	-4108.24182	-4108.71042	-4108.53410	-4109.00290	-4108.89377	-4109.36161	-4109.79971	-4110.31291
3.31	-4106.14610	-4108.29025	-4108.75956	-4108.58347	-4109.05348	-4108.94146	-4109.40892	-4109.84765	-4110.36184
3.40	-4106.18489	-4108.32714	-4108.79562	-4108.62097	-4109.09014	-4108.97875	-4109.44545	-4109.88470	-4110.39793
3.50	-4106.21662	-4108.35683	-4108.82476	-4108.65116	-4109.11983	-4109.00883	-4109.47489	-4109.91422	-4110.42676
3.59	-4106.24225	-4108.38040	-4108.84786	-4108.67519	-4109.14344	-4109.03285	-4109.49830	-4109.93756	-4110.44947
3.68	-4106.26272	-4108.39892	-4108.86599	-4108.69413	-4109.16206	-4109.05186	-4109.51678	-4109.95583	-4110.46722
3.78	-4106.27886	-4108.41335	-4108.88015	-4108.70896	-4109.17669	-4109.06683	-4109.53132	-4109.96995	-4110.48098
3.87	-4106.29141	-4108.42446	-4108.89112	-4108.72043	-4109.18810	-4109.07856	-4109.54273	-4109.98067	-4110.49150
3.97	-4106.30100	-4108.43288	-4108.89953	-4108.72914	-4109.19692	-4109.08769	-4109.55163	-4109.98855	-4110.49942
4.06	-4106.30811	-4108.43907	-4108.90580	-4108.73555	-4109.20354	-4109.09466	-4109.55842	-4109.99405	-4110.50513
4.16	-4106.31315	-4108.44333	-4108.91018	-4108.73994	-4109.20821	-4109.09976	-4109.56336	-4109.99747	-4110.50888
4.35	-4106.31645	-4108.44588	-4108.91286	-4108.74255	-4109.21112	-4109.10321	-4109.56662	-4109.99899	-4110.51078
4.44	-4106.31835	-4108.44688	-4108.91400	-4108.74347	-4109.21240	-4109.10519	-4109.56839	-4109.99854	-4110.51080
4.54	-4106.32393	-4108.44480	-4108.91147	-4108.74070	-4109.20958	-4109.10418	-4109.56640	-4109.99217	-4110.50395
4.63	-4106.32392	-4108.44275	-4108.90954	-4108.73859	-4109.20781	-4109.10285	-4109.56484	-4109.98897	-4110.50098
4.72	-4106.32315	-4108.43987	-4108.90684	-4108.73564	-4109.20528	-4109.10062	-4109.56243	-4109.98495	-4110.49729
4.82	-4106.32181	-4108.43629	-4108.90348	-4108.73200	-4109.20213	-4109.09765	-4109.55928	-4109.98029	-4110.49303
4.91	-4106.32004	-4108.43220	-4108.89964	-4108.72786	-4109.19851	-4109.09408	-4109.55558	-4109.97516	-4110.48836
5.01	-4106.31797	-4108.42771	-4108.89541	-4108.72333	-4109.19453	-4109.09006	-4109.55144	-4109.96972	-4110.48342

Table S18: The absolute energies in hartree of the potential energy curve of NbSi in the $^2\Sigma^+$ state with a SP-based wave function and nom-CPO active space.

Distance	SP	tpbe	ftpbe	trevpbe	ftrevpbe	tblyp	ftblyp	torelyp	ftorelyp
5.10	-4106.31570	-4108.42299	-4108.89093	-4108.71859	-4109.19030	-4109.08575	-4109.54702	-4109.96413	-4110.47833
5.20	-4106.31329	-4108.41812	-4108.88629	-4108.71372	-4109.18592	-4109.08126	-4109.54241	-4109.95850	-4110.47318
5.29	-4106.31079	-4108.41320	-4108.88156	-4108.70882	-4109.18144	-4109.07667	-4109.53771	-4109.95293	-4110.46805
5.39	-4106.30824	-4108.40831	-4108.87681	-4108.70396	-4109.17694	-4109.07209	-4109.53300	-4109.94750	-4110.46299
5.48	-4106.30565	-4108.40352	-4108.87212	-4108.69923	-4109.17247	-4109.06760	-4109.52837	-4109.94228	-4110.45807
5.57	-4106.30303	-4108.39891	-4108.86757	-4108.69468	-4109.16813	-4109.06328	-4109.52389	-4109.93735	-4110.45338
5.67	-4106.30039	-4108.39451	-4108.86321	-4108.69036	-4109.16397	-4109.05917	-4109.51963	-4109.93273	-4110.44894
5.76	-4106.29773	-4108.39030	-4108.85902	-4108.68623	-4109.15996	-4109.05525	-4109.51557	-4109.92836	-4110.44471
5.86	-4106.29507	-4108.38625	-4108.85497	-4108.68227	-4109.15607	-4109.05150	-4109.51165	-4109.92424	-4110.44068
5.95	-4106.29241	-4108.38237	-4108.85105	-4108.67848	-4109.15231	-4109.04792	-4109.50790	-4109.92032	-4110.43683
6.05	-4106.28977	-4108.37866	-4108.84730	-4108.67486	-4109.14871	-4109.04451	-4109.50433	-4109.91662	-4110.43318
6.14	-4106.28716	-4108.37510	-4108.84371	-4108.67139	-4109.14524	-4109.04126	-4109.50094	-4109.91310	-4110.42970
6.24	-4106.28458	-4108.37171	-4108.84026	-4108.66808	-4109.14190	-4109.03818	-4109.49770	-4109.90979	-4110.42637
6.33	-4106.28204	-4108.36846	-4108.83692	-4108.66492	-4109.13866	-4109.03526	-4109.49459	-4109.90665	-4110.42319
6.43	-4106.27956	-4108.36537	-4108.83373	-4108.66191	-4109.13555	-4109.03249	-4109.49165	-4109.90368	-4110.42018
6.52	-4106.27714	-4108.36240	-4108.83068	-4108.65903	-4109.13258	-4109.02987	-4109.48887	-4109.90087	-4110.41733
6.61	-4106.27479	-4108.35960	-4108.82778	-4108.65630	-4109.12974	-4109.02742	-4109.48626	-4109.89824	-4110.41463
6.71	-4106.27253	-4108.35693	-4108.82501	-4108.65371	-4109.12702	-4109.02513	-4109.48382	-4109.89578	-4110.41209
6.80	-4106.27035	-4108.35442	-4108.82240	-4108.65127	-4109.12445	-4109.02301	-4109.48157	-4109.89348	-4110.40971
6.90	-4106.26826	-4108.35206	-4108.81996	-4108.64897	-4109.12203	-4109.02106	-4109.47950	-4109.89136	-4110.40752
6.99	-4106.26628	-4108.34983	-4108.81765	-4108.64681	-4109.11974	-4109.01925	-4109.47762	-4109.88939	-4110.40548
7.09	-4106.26441	-4108.34771	-4108.81547	-4108.64476	-4109.11757	-4109.01759	-4109.47590	-4109.88758	-4110.40360
7.18	-4106.26264	-4108.34572	-4108.81343	-4108.64283	-4109.11551	-4109.01607	-4109.47434	-4109.88589	-4110.40184
7.28	-4106.26100	-4108.34382	-4108.81149	-4108.64099	-4109.11356	-4109.01466	-4109.47292	-4109.88433	-4110.40020
7.37	-4106.25947	-4108.34202	-4108.80966	-4108.63925	-4109.11171	-4109.01336	-4109.47164	-4109.88288	-4110.39868
7.46	-4106.25807	-4108.34031	-4108.80792	-4108.63760	-4109.10996	-4109.01215	-4109.47047	-4109.88154	-4110.39726
7.56	-4106.25678	-4108.33868	-4108.80629	-4108.63604	-4109.10830	-4109.01102	-4109.46941	-4109.88029	-4110.39594
7.65	-4106.25561	-4108.33712	-4108.80472	-4108.63454	-4109.10672	-4109.00994	-4109.46842	-4109.87911	-4110.39471
7.75	-4106.25454	-4108.33564	-4108.80324	-4108.63313	-4109.10522	-4109.00894	-4109.46750	-4109.87801	-4110.39353
7.84	-4106.25358	-4108.33422	-4108.80182	-4108.63178	-4109.10380	-4109.00797	-4109.46664	-4109.87697	-4110.39242
7.94	-4106.25271	-4108.33287	-4108.80047	-4108.63050	-4109.10244	-4109.00705	-4109.46581	-4109.87599	-4110.39137
8.03	-4106.25193	-4108.33159	-4108.79919	-4108.62928	-4109.10116	-4109.00616	-4109.46503	-4109.87507	-4110.39038
8.13	-4106.25122	-4108.33037	-4108.79798	-4108.62814	-4109.09996	-4109.00532	-4109.46429	-4109.87422	-4110.38945
8.22	-4106.25060	-4108.32923	-4108.79685	-4108.62707	-4109.09883	-4109.00451	-4109.46358	-4109.87343	-4110.38859
8.31	-4106.25003	-4108.32817	-4108.79579	-4108.62607	-4109.09780	-4109.00376	-4109.46293	-4109.87270	-4110.38780
8.41	-4106.24953	-4108.32718	-4108.79481	-4108.62515	-4109.09684	-4109.00304	-4109.46232	-4109.87204	-4110.38708
8.50	-4106.24908	-4108.32626	-4108.79390	-4108.62431	-4109.09596	-4109.00237	-4109.46175	-4109.87144	-4110.38644
8.60	-4106.24869	-4108.32542	-4108.79308	-4108.62355	-4109.09517	-4109.00176	-4109.46123	-4109.87092	-4110.38587
8.69	-4106.24833	-4108.32468	-4108.79234	-4108.62288	-4109.09447	-4109.00121	-4109.46076	-4109.87047	-4110.38537
8.79	-4106.24802	-4108.32403	-4108.79170	-4108.62230	-4109.09386	-4109.00074	-4109.46035	-4109.87009	-4110.38494
8.88	-4106.24774	-4108.32346	-4108.79112	-4108.62179	-4109.09333	-4109.00033	-4109.45999	-4109.86976	-4110.38458
8.98	-4106.24749	-4108.32296	-4108.79062	-4108.62135	-4109.09286	-4108.99997	-4109.45968	-4109.86948	-4110.38426
9.07	-4106.24727	-4108.32252	-4108.79018	-4108.62097	-4109.09246	-4108.99966	-4109.45940	-4109.86925	-4110.38399
9.17	-4106.24707	-4108.32213	-4108.78978	-4108.62064	-4109.09210	-4108.99939	-4109.45916	-4109.86905	-4110.38375
9.26	-4106.24689	-4108.32178	-4108.78943	-4108.62035	-4109.09179	-4108.99916	-4109.45894	-4109.86889	-4110.38355
9.35	-4106.24673	-4108.32148	-4108.78912	-4108.62010	-4109.09152	-4108.99896	-4109.45876	-4109.86875	-4110.38338
9.45	-4106.24658	-4108.32121	-4108.78885	-4108.61988	-4109.09129	-4108.99879	-4109.45860	-4109.86865	-4110.38324
9.54	-4106.24645	-4108.32098	-4108.78861	-4108.61970	-4109.09109	-4108.99864	-4109.45846	-4109.86856	-4110.38312
9.64	-4106.24633	-4108.32076	-4108.78839	-4108.61953	-4109.09090	-4108.99851	-4109.45834	-4109.86848	-4110.38302
9.73	-4106.24622	-4108.32057	-4108.78819	-4108.61938	-4109.09074	-4108.99840	-4109.45822	-4109.86843	-4110.38293
9.83	-4106.24612	-4108.32040	-4108.78801	-4108.61925	-4109.09059	-4108.99829	-4109.45812	-4109.86837	-4110.38286
9.92	-4106.24603	-4108.32024	-4108.78783	-4108.61913	-4109.09046	-4108.99820	-4109.45802	-4109.86832	-4110.38279

Table S18: The absolute energies in hartree of the potential energy curve of NbSi in the $^2\Sigma^+$ state with a SP-based wave function and nom-CPO active space.

Distance	SP	tpbe	ftpbe	trevpbe	ftrevpbe	tblyp	ftblyp	torelyp	ftorelyp
10.02	-4106.24595	-4108.32010	-4108.78769	-4108.61903	-4109.09034	-4108.99812	-4109.45794	-4109.86829	-4110.38273
10.11	-4106.24588	-4108.31997	-4108.78756	-4108.61894	-4109.09024	-4108.99805	-4109.45787	-4109.86827	-4110.38269
10.20	-4106.24581	-4108.31985	-4108.78743	-4108.61886	-4109.09015	-4108.99799	-4109.45780	-4109.86825	-4110.38265
10.30	-4106.24575	-4108.31975	-4108.78732	-4108.61879	-4109.09007	-4108.99793	-4109.45775	-4109.86823	-4110.38262
10.39	-4106.24569	-4108.31967	-4108.78723	-4108.61874	-4109.09000	-4108.99789	-4109.45771	-4109.86823	-4110.38260
10.49	-4106.24564	-4108.31959	-4108.78715	-4108.61869	-4109.08995	-4108.99786	-4109.45767	-4109.86823	-4110.38258
10.58	-4106.24559	-4108.31952	-4108.78708	-4108.61866	-4109.08990	-4108.99784	-4109.45764	-4109.86824	-4110.38257
10.68	-4106.24554	-4108.31946	-4108.78701	-4108.61862	-4109.08986	-4108.99782	-4109.45762	-4109.86825	-4110.38257
10.77	-4106.24550	-4108.31941	-4108.78695	-4108.61859	-4109.08982	-4108.99780	-4109.45760	-4109.86826	-4110.38256
10.87	-4106.24546	-4108.31936	-4108.78689	-4108.61857	-4109.08978	-4108.99779	-4109.45758	-4109.86827	-4110.38255
10.96	-4106.24543	-4108.31931	-4108.78684	-4108.61854	-4109.08975	-4108.99777	-4109.45756	-4109.86828	-4110.38255
11.05	-4106.24540	-4108.31926	-4108.78679	-4108.61852	-4109.08971	-4108.99776	-4109.45754	-4109.86828	-4110.38254
11.15	-4106.24537	-4108.31922	-4108.78674	-4108.61850	-4109.08969	-4108.99775	-4109.45753	-4109.86829	-4110.38254
11.24	-4106.24534	-4108.31919	-4108.78670	-4108.61848	-4109.08966	-4108.99775	-4109.45752	-4109.86830	-4110.38254
11.34	-4106.24531	-4108.31915	-4108.78666	-4108.61846	-4109.08964	-4108.99774	-4109.45751	-4109.86830	-4110.38253
11.43	-4106.24529	-4108.31912	-4108.78663	-4108.61845	-4109.08962	-4108.99774	-4109.45750	-4109.86831	-4110.38253
11.53	-4106.24527	-4108.31909	-4108.78660	-4108.61844	-4109.08960	-4108.99774	-4109.45750	-4109.86832	-4110.38253
11.62	-4106.24525	-4108.31907	-4108.78657	-4108.61843	-4109.08959	-4108.99774	-4109.45750	-4109.86833	-4110.38253
11.72	-4106.24523	-4108.31905	-4108.78655	-4108.61842	-4109.08957	-4108.99774	-4109.45750	-4109.86834	-4110.38253
11.81	-4106.24521	-4108.31903	-4108.78653	-4108.61842	-4109.08957	-4108.99775	-4109.45750	-4109.86835	-4110.38254
11.91	-4106.24519	-4108.31902	-4108.78651	-4108.61841	-4109.08956	-4108.99776	-4109.45751	-4109.86836	-4110.38254
12.00	-4106.24518	-4108.31900	-4108.78649	-4108.61841	-4109.08955	-4108.99776	-4109.45751	-4109.86837	-4110.38254
12.09	-4106.24516	-4108.31899	-4108.78648	-4108.61841	-4109.08954	-4108.99777	-4109.45752	-4109.86838	-4110.38255
12.19	-4106.24515	-4108.31898	-4108.78646	-4108.61840	-4109.08954	-4108.99778	-4109.45752	-4109.86839	-4110.38255
12.28	-4106.24513	-4108.31896	-4108.78645	-4108.61840	-4109.08953	-4108.99778	-4109.45753	-4109.86840	-4110.38255
12.38	-4106.24512	-4108.31895	-4108.78643	-4108.61840	-4109.08952	-4108.99779	-4109.45753	-4109.86840	-4110.38254
12.47	-4106.24511	-4108.31894	-4108.78641	-4108.61839	-4109.08951	-4108.99779	-4109.45753	-4109.86840	-4110.38254
12.57	-4106.24510	-4108.31893	-4108.78640	-4108.61839	-4109.08950	-4108.99780	-4109.45753	-4109.86841	-4110.38254
12.66	-4106.24509	-4108.31892	-4108.78639	-4108.61838	-4109.08949	-4108.99780	-4109.45753	-4109.86841	-4110.38253
12.76	-4106.24508	-4108.31891	-4108.78637	-4108.61838	-4109.08948	-4108.99781	-4109.45754	-4109.86841	-4110.38253
12.85	-4106.24507	-4108.31890	-4108.78636	-4108.61837	-4109.08948	-4108.99781	-4109.45754	-4109.86841	-4110.38253
12.94	-4106.24506	-4108.31889	-4108.78636	-4108.61837	-4109.08948	-4108.99782	-4109.45755	-4109.86841	-4110.38253
13.04	-4106.24506	-4108.31888	-4108.78635	-4108.61837	-4109.08947	-4108.99783	-4109.45755	-4109.86842	-4110.38253
13.13	-4106.24505	-4108.31888	-4108.78634	-4108.61837	-4109.08947	-4108.99783	-4109.45756	-4109.86842	-4110.38253
13.23	-4106.24504	-4108.31887	-4108.78634	-4108.61837	-4109.08947	-4108.99784	-4109.45757	-4109.86842	-4110.38253
13.32	-4106.24503	-4108.31887	-4108.78634	-4108.61837	-4109.08947	-4108.99785	-4109.45758	-4109.86843	-4110.38253
13.42	-4106.24503	-4108.31887	-4108.78633	-4108.61837	-4109.08947	-4108.99786	-4109.45758	-4109.86843	-4110.38254
13.51	-4106.24502	-4108.31886	-4108.78633	-4108.61837	-4109.08947	-4108.99787	-4109.45759	-4109.86844	-4110.38254
13.61	-4106.24502	-4108.31886	-4108.78632	-4108.61837	-4109.08947	-4108.99788	-4109.45760	-4109.86844	-4110.38254
13.70	-4106.24501	-4108.31886	-4108.78632	-4108.61837	-4109.08946	-4108.99788	-4109.45760	-4109.86844	-4110.38254
13.80	-4106.24500	-4108.31886	-4108.78632	-4108.61837	-4109.08946	-4108.99789	-4109.45761	-4109.86844	-4110.38253
13.89	-4106.24500	-4108.31885	-4108.78631	-4108.61837	-4109.08946	-4108.99789	-4109.45761	-4109.86844	-4110.38253
13.98	-4106.24499	-4108.31885	-4108.78631	-4108.61836	-4109.08945	-4108.99790	-4109.45761	-4109.86844	-4110.38253
14.08	-4106.24499	-4108.31884	-4108.78630	-4108.61836	-4109.08945	-4108.99790	-4109.45762	-4109.86844	-4110.38253
14.17	-4106.24499	-4108.31884	-4108.78630	-4108.61836	-4109.08945	-4108.99790	-4109.45762	-4109.86844	-4110.38253
14.27	-4106.24498	-4108.31884	-4108.78629	-4108.61836	-4109.08944	-4108.99791	-4109.45762	-4109.86844	-4110.38252
14.36	-4106.24498	-4108.31883	-4108.78629	-4108.61836	-4109.08944	-4108.99791	-4109.45763	-4109.86844	-4110.38252
14.46	-4106.24497	-4108.31883	-4108.78629	-4108.61836	-4109.08944	-4108.99792	-4109.45763	-4109.86844	-4110.38252
14.55	-4106.24497	-4108.31883	-4108.78629	-4108.61835	-4109.08944	-4108.99792	-4109.45763	-4109.86844	-4110.38252
14.65	-4106.24497	-4108.31883	-4108.78628	-4108.61835	-4109.08944	-4108.99792	-4109.45764	-4109.86844	-4110.38252
14.74	-4106.24496	-4108.31882	-4108.78628	-4108.61835	-4109.08944	-4108.99793	-4109.45764	-4109.86844	-4110.38252
14.83	-4106.24496	-4108.31882	-4108.78628	-4108.61835	-4109.08944	-4108.99793	-4109.45765	-4109.86844	-4110.38252

Table S18: The absolute energies in hartree of the potential energy curve of NbSi in the $^2\Sigma^+$ state with a SP-based wave function and nom-CPO active space.

Distance	SP	tpbe	ftpbe	trevpbe	ftrevpbe	tblyp	ftblyp	torelyp	ftorelyp
14.93	-4106.24496	-4108.31882	-4108.78628	-4108.61835	-4109.08944	-4108.99794	-4109.45765	-4109.86844	-4110.38252
15.02	-4106.24495	-4108.31882	-4108.78628	-4108.61835	-4109.08944	-4108.99794	-4109.45766	-4109.86844	-4110.38252
15.12	-4106.24495	-4108.31882	-4108.78628	-4108.61835	-4109.08944	-4108.99795	-4109.45766	-4109.86844	-4110.38252
15.21	-4106.24495	-4108.31882	-4108.78628	-4108.61835	-4109.08944	-4108.99795	-4109.45766	-4109.86844	-4110.38252
15.31	-4106.24495	-4108.31882	-4108.78628	-4108.61836	-4109.08944	-4108.99796	-4109.45767	-4109.86845	-4110.38252
15.40	-4106.24494	-4108.31882	-4108.78628	-4108.61836	-4109.08944	-4108.99796	-4109.45767	-4109.86845	-4110.38252
15.50	-4106.24494	-4108.31882	-4108.78628	-4108.61836	-4109.08944	-4108.99796	-4109.45767	-4109.86845	-4110.38252
15.59	-4106.24494	-4108.31882	-4108.78628	-4108.61836	-4109.08943	-4108.99797	-4109.45768	-4109.86845	-4110.38252
15.68	-4106.24494	-4108.31882	-4108.78628	-4108.61835	-4109.08943	-4108.99797	-4109.45768	-4109.86845	-4110.38252
15.78	-4106.24494	-4108.31882	-4108.78628	-4108.61835	-4109.08943	-4108.99797	-4109.45768	-4109.86845	-4110.38252
15.87	-4106.24493	-4108.31882	-4108.78627	-4108.61835	-4109.08943	-4108.99797	-4109.45768	-4109.86844	-4110.38252
15.97	-4106.24493	-4108.31882	-4108.78627	-4108.61835	-4109.08943	-4108.99797	-4109.45769	-4109.86844	-4110.38252
16.06	-4106.24493	-4108.31882	-4108.78627	-4108.61835	-4109.08943	-4108.99798	-4109.45769	-4109.86844	-4110.38251
16.16	-4106.24493	-4108.31882	-4108.78627	-4108.61835	-4109.08943	-4108.99798	-4109.45769	-4109.86844	-4110.38251
16.25	-4106.24493	-4108.31881	-4108.78627	-4108.61835	-4109.08943	-4108.99798	-4109.45769	-4109.86844	-4110.38251
16.35	-4106.24492	-4108.31881	-4108.78627	-4108.61835	-4109.08943	-4108.99798	-4109.45769	-4109.86844	-4110.38251
16.44	-4106.24492	-4108.31881	-4108.78627	-4108.61835	-4109.08943	-4108.99798	-4109.45769	-4109.86844	-4110.38251
16.54	-4106.24492	-4108.31881	-4108.78627	-4108.61835	-4109.08942	-4108.99798	-4109.45769	-4109.86844	-4110.38251
16.63	-4106.24492	-4108.31881	-4108.78627	-4108.61835	-4109.08942	-4108.99798	-4109.45769	-4109.86844	-4110.38251
16.72	-4106.24492	-4108.31881	-4108.78627	-4108.61835	-4109.08942	-4108.99799	-4109.45770	-4109.86844	-4110.38251
16.82	-4106.24492	-4108.31881	-4108.78627	-4108.61834	-4109.08942	-4108.99799	-4109.45770	-4109.86844	-4110.38251
16.91	-4106.24492	-4108.31881	-4108.78627	-4108.61835	-4109.08942	-4108.99799	-4109.45770	-4109.86844	-4110.38251
17.01	-4106.24491	-4108.31881	-4108.78627	-4108.61835	-4109.08942	-4108.99799	-4109.45770	-4109.86844	-4110.38251
17.10	-4106.24491	-4108.31881	-4108.78627	-4108.61835	-4109.08942	-4108.99799	-4109.45770	-4109.86844	-4110.38251
17.20	-4106.24491	-4108.31881	-4108.78627	-4108.61835	-4109.08942	-4108.99799	-4109.45770	-4109.86844	-4110.38251
17.29	-4106.24491	-4108.31881	-4108.78627	-4108.61835	-4109.08942	-4108.99800	-4109.45771	-4109.86844	-4110.38251
17.39	-4106.24491	-4108.31881	-4108.78627	-4108.61835	-4109.08942	-4108.99800	-4109.45771	-4109.86844	-4110.38251
17.48	-4106.24491	-4108.31881	-4108.78627	-4108.61835	-4109.08942	-4108.99800	-4109.45771	-4109.86844	-4110.38251
17.57	-4106.24491	-4108.31881	-4108.78627	-4108.61835	-4109.08942	-4108.99800	-4109.45771	-4109.86844	-4110.38251
17.67	-4106.24491	-4108.31881	-4108.78627	-4108.61835	-4109.08942	-4108.99800	-4109.45771	-4109.86844	-4110.38251
17.76	-4106.24491	-4108.31881	-4108.78627	-4108.61835	-4109.08942	-4108.99800	-4109.45771	-4109.86844	-4110.38251
17.86	-4106.24491	-4108.31881	-4108.78627	-4108.61835	-4109.08942	-4108.99800	-4109.45771	-4109.86844	-4110.38251
17.95	-4106.24490	-4108.31881	-4108.78627	-4108.61834	-4109.08942	-4108.99800	-4109.45771	-4109.86844	-4110.38251
18.05	-4106.24490	-4108.31881	-4108.78627	-4108.61834	-4109.08942	-4108.99800	-4109.45771	-4109.86844	-4110.38251

Table S19: The absolute energies in hartree of the potential energy curve of NbSi in the $^6\Sigma^+$ state with a SP-based wave function and nom-CPO active space.

Distance	SP	tpbe	ftpbe	trevpbe	ftrevpbe	tblyp	ftblyp	torelyp	ftorelyp
1.98	-4102.63312	-4104.86329	-4105.33759	-4105.14365	-4105.61946	-4105.50086	-4105.97655	-4106.43674	-4106.95773
2.08	-4103.26727	-4105.49175	-4105.96620	-4105.77329	-4106.24922	-4106.13232	-4106.60679	-4107.06170	-4107.58243
2.17	-4103.77686	-4105.99625	-4106.47088	-4106.27907	-4106.75505	-4106.63878	-4107.11278	-4107.56356	-4108.08381
2.27	-4104.18958	-4106.39972	-4106.87013	-4106.68398	-4107.15487	-4107.04409	-4107.51362	-4107.96616	-4108.48113
2.36	-4104.53227	-4106.72900	-4107.20211	-4107.01438	-4107.48842	-4107.37561	-4107.84689	-4108.29331	-4108.81116
2.46	-4104.81427	-4107.00192	-4107.47469	-4107.28827	-4107.76190	-4107.64997	-4108.12109	-4108.56415	-4109.08142
2.55	-4105.04368	-4107.22310	-4107.69561	-4107.51031	-4107.98370	-4107.87276	-4108.34374	-4108.78330	-4109.30033
2.65	-4105.24865	-4107.39537	-4107.87195	-4107.68276	-4108.16163	-4108.04901	-4108.52435	-4108.94801	-4109.47288
2.74	-4105.41314	-4107.55493	-4108.03285	-4107.84309	-4108.32354	-4108.20914	-4108.68573	-4109.10634	-4109.63287
2.83	-4105.55044	-4107.68873	-4108.16721	-4107.97759	-4108.45869	-4108.34362	-4108.82079	-4109.23910	-4109.76626
2.93	-4105.66983	-4107.80245	-4108.28222	-4108.09195	-4108.57478	-4108.45730	-4108.93526	-4109.35112	-4109.88037
3.02	-4105.76696	-4107.89855	-4108.37805	-4108.18863	-4108.67111	-4108.55422	-4109.03200	-4109.44646	-4109.97527

Table S19: The absolute energies in hartree of the potential energy curve of NbSi in the $^6\Sigma^+$ state with a SP-based wave function and nom-CPO active space.

Distance	SP	tpbe	ftpbe	trevpbe	ftrevpbe	tblyp	ftblyp	torelyp	ftorelyp
3.12	-4105.84873	-4107.98025	-4108.45935	-4108.27088	-4108.75285	-4108.63675	-4109.11422	-4109.52751	-4110.05573
3.21	-4105.91771	-4108.04988	-4108.52845	-4108.34102	-4108.82236	-4108.70720	-4109.18422	-4109.59655	-4110.12413
3.31	-4105.97600	-4108.10935	-4108.58724	-4108.40097	-4108.88153	-4108.76749	-4109.24393	-4109.65553	-4110.18234
3.40	-4106.02535	-4108.16027	-4108.63725	-4108.45235	-4108.93188	-4108.81923	-4109.29488	-4109.70610	-4110.23187
3.50	-4106.06727	-4108.20412	-4108.67960	-4108.49665	-4108.97439	-4108.86398	-4109.33827	-4109.74990	-4110.27381
3.59	-4106.11388	-4108.24236	-4108.71467	-4108.53515	-4109.00940	-4108.90319	-4109.37428	-4109.78862	-4110.30836
3.68	-4106.14613	-4108.27215	-4108.74437	-4108.56530	-4109.03951	-4108.93347	-4109.40438	-4109.81785	-4110.33755
3.78	-4106.17298	-4108.29679	-4108.76894	-4108.59030	-4109.06448	-4108.95860	-4109.42937	-4109.84198	-4110.36170
3.87	-4106.19515	-4108.31696	-4108.78896	-4108.61079	-4109.08487	-4108.97925	-4109.44979	-4109.86167	-4110.38130
3.97	-4106.21325	-4108.33324	-4108.80507	-4108.62739	-4109.10129	-4108.99602	-4109.46628	-4109.87751	-4110.39697
4.06	-4106.22784	-4108.34622	-4108.81794	-4108.64065	-4109.11450	-4109.00944	-4109.47953	-4109.89007	-4110.40951
4.16	-4106.23943	-4108.35636	-4108.82802	-4108.65107	-4109.12491	-4109.02005	-4109.49001	-4109.89982	-4110.41933
4.35	-4106.24843	-4108.36407	-4108.83564	-4108.65903	-4109.13283	-4109.02817	-4109.49800	-4109.90714	-4110.42667
4.44	-4106.25523	-4108.36962	-4108.84107	-4108.66482	-4109.13853	-4109.03414	-4109.50374	-4109.91231	-4110.43177
4.54	-4106.26017	-4108.37336	-4108.84466	-4108.66878	-4109.14238	-4109.03826	-4109.50762	-4109.91567	-4110.43504
4.63	-4106.26357	-4108.37560	-4108.84678	-4108.67124	-4109.14475	-4109.04088	-4109.51002	-4109.91761	-4110.43694
4.72	-4106.26568	-4108.37671	-4108.84772	-4108.67256	-4109.14592	-4109.04237	-4109.51129	-4109.91855	-4110.43775
4.82	-4106.26670	-4108.37682	-4108.84765	-4108.67288	-4109.14606	-4109.04288	-4109.51155	-4109.91854	-4110.43756
4.91	-4106.26682	-4108.37605	-4108.84673	-4108.67230	-4109.14534	-4109.04248	-4109.51091	-4109.91762	-4110.43649
5.01	-4106.26619	-4108.37455	-4108.84510	-4108.67097	-4109.14391	-4109.04133	-4109.50955	-4109.91594	-4110.43469
5.10	-4106.26494	-4108.37245	-4108.84291	-4108.66904	-4109.14191	-4109.03957	-4109.50760	-4109.91366	-4110.43233
5.20	-4106.26319	-4108.36987	-4108.84025	-4108.66662	-4109.13946	-4109.03732	-4109.50518	-4109.91091	-4110.42953
5.29	-4106.26106	-4108.36686	-4108.83720	-4108.66377	-4109.13661	-4109.03462	-4109.50232	-4109.90772	-4110.42633
5.39	-4106.25864	-4108.36349	-4108.83378	-4108.66056	-4109.13340	-4109.03154	-4109.49909	-4109.90418	-4110.42277
5.48	-4106.25599	-4108.35981	-4108.83005	-4108.65703	-4109.12986	-4109.02815	-4109.49552	-4109.90033	-4110.41888
5.57	-4106.25319	-4108.35583	-4108.82603	-4108.65319	-4109.12603	-4109.02443	-4109.49163	-4109.89617	-4110.41469
5.67	-4106.25030	-4108.35153	-4108.82171	-4108.64905	-4109.12190	-4109.02038	-4109.48741	-4109.89170	-4110.41020
5.76	-4106.24737	-4108.34682	-4108.81698	-4108.64448	-4109.11738	-4109.01589	-4109.48276	-4109.88679	-4110.40531
5.86	-4106.24445	-4108.34158	-4108.81174	-4108.63940	-4109.11236	-4109.01085	-4109.47755	-4109.88136	-4110.39990
5.95	-4106.24158	-4108.33608	-4108.80622	-4108.63407	-4109.10705	-4109.00554	-4109.47207	-4109.87570	-4110.39427
6.05	-4106.23876	-4108.33092	-4108.80102	-4108.62904	-4109.10204	-4109.00058	-4109.46695	-4109.87045	-4110.38902
6.14	-4106.23599	-4108.32626	-4108.79632	-4108.62452	-4109.09750	-4108.99615	-4109.46237	-4109.86577	-4110.38433
6.24	-4106.23326	-4108.32204	-4108.79205	-4108.62042	-4109.09337	-4108.99218	-4109.45827	-4109.86156	-4110.38011
6.33	-4106.23059	-4108.31817	-4108.78813	-4108.61666	-4109.08958	-4108.98858	-4109.45455	-4109.85773	-4110.37628
6.43	-4106.22796	-4108.31459	-4108.78450	-4108.61318	-4109.08606	-4108.98529	-4109.45115	-4109.85422	-4110.37275
6.52	-4106.22540	-4108.31126	-4108.78111	-4108.60994	-4109.08278	-4108.98226	-4109.44801	-4109.85096	-4110.36947
6.61	-4106.22290	-4108.30815	-4108.77796	-4108.60691	-4109.07970	-4108.97946	-4109.44512	-4109.84794	-4110.36642
6.71	-4106.22047	-4108.30524	-4108.77500	-4108.60408	-4109.07682	-4108.97689	-4109.44245	-4109.84514	-4110.36359
6.80	-4106.21812	-4108.30255	-4108.77226	-4108.60145	-4109.07415	-4108.97454	-4109.44002	-4109.84255	-4110.36099
6.90	-4106.21586	-4108.30004	-4108.76972	-4108.59901	-4109.07166	-4108.97238	-4109.43780	-4109.84016	-4110.35859
6.99	-4106.21369	-4108.29771	-4108.76737	-4108.59674	-4109.06936	-4108.97042	-4109.43580	-4109.83797	-4110.35639
7.09	-4106.21161	-4108.29555	-4108.76518	-4108.59464	-4109.06722	-4108.96864	-4109.43398	-4109.83595	-4110.35436
7.18	-4106.20964	-4108.29355	-4108.76316	-4108.59269	-4109.06524	-4108.96703	-4109.43234	-4109.83410	-4110.35250
7.28	-4106.20778	-4108.29169	-4108.76129	-4108.59087	-4109.06340	-4108.96557	-4109.43087	-4109.83240	-4110.35080
7.37	-4106.20603	-4108.28997	-4108.75956	-4108.58920	-4109.06170	-4108.96427	-4109.42956	-4109.83086	-4110.34924
7.46	-4106.20440	-4108.28838	-4108.75798	-4108.58766	-4109.06014	-4108.96311	-4109.42841	-4109.82947	-4110.34785
7.56	-4106.20290	-4108.28690	-4108.75650	-4108.58623	-4109.05869	-4108.96207	-4109.42739	-4109.82820	-4110.34657
7.65	-4106.20153	-4108.28550	-4108.75511	-4108.58487	-4109.05732	-4108.96113	-4109.42647	-4109.82702	-4110.34539
7.75	-4106.20030	-4108.28411	-4108.75374	-4108.58353	-4109.05598	-4108.96020	-4109.42561	-4109.82588	-4110.34426
7.84	-4106.19922	-4108.28267	-4108.75232	-4108.58214	-4109.05460	-4108.95924	-4109.42471	-4109.82472	-4110.34311
7.94	-4106.19829	-4108.28116	-4108.75085	-4108.58069	-4109.05315	-4108.95820	-4109.42375	-4109.82350	-4110.34192
8.03	-4106.19749	-4108.27965	-4108.74937	-4108.57924	-4109.05171	-4108.95711	-4109.42276	-4109.82229	-4110.34074

Table S19: The absolute energies in hartree of the potential energy curve of NbSi in the $^6\Sigma^+$ state with a SP-based wave function and nom-CPO active space.

Distance	SP	tpbe	ftpbe	trevpbe	ftrevpbe	tblyp	ftblyp	torelyp	ftorelyp
8.13	-4106.19680	-4108.27820	-4108.74795	-4108.57785	-4109.05033	-4108.95604	-4109.42178	-4109.82114	-4110.33961
8.22	-4106.19622	-4108.27690	-4108.74668	-4108.57661	-4109.04910	-4108.95506	-4109.42089	-4109.82012	-4110.33862
8.31	-4106.19571	-4108.27577	-4108.74558	-4108.57555	-4109.04805	-4108.95421	-4109.42011	-4109.81927	-4110.33777
8.41	-4106.19527	-4108.27481	-4108.74464	-4108.57464	-4109.04714	-4108.95349	-4109.41945	-4109.81855	-4110.33706
8.50	-4106.19487	-4108.27397	-4108.74381	-4108.57386	-4109.04636	-4108.95287	-4109.41887	-4109.81794	-4110.33644
8.60	-4106.19453	-4108.27323	-4108.74308	-4108.57318	-4109.04568	-4108.95233	-4109.41836	-4109.81742	-4110.33592
8.69	-4106.19421	-4108.27258	-4108.74244	-4108.57259	-4109.04508	-4108.95185	-4109.41792	-4109.81698	-4110.33546
8.79	-4106.19394	-4108.27200	-4108.74187	-4108.57206	-4109.04455	-4108.95143	-4109.41753	-4109.81659	-4110.33507
8.88	-4106.19369	-4108.27148	-4108.74136	-4108.57160	-4109.04408	-4108.95106	-4109.41717	-4109.81627	-4110.33473
8.98	-4106.19346	-4108.27102	-4108.74090	-4108.57119	-4109.04367	-4108.95073	-4109.41686	-4109.81598	-4110.33443
9.07	-4106.19326	-4108.27060	-4108.74049	-4108.57083	-4109.04330	-4108.95043	-4109.41659	-4109.81574	-4110.33418
9.17	-4106.19307	-4108.27023	-4108.74012	-4108.57051	-4109.04297	-4108.95017	-4109.41634	-4109.81554	-4110.33396
9.26	-4106.19291	-4108.26990	-4108.73979	-4108.57023	-4109.04268	-4108.94995	-4109.41612	-4109.81537	-4110.33377
9.35	-4106.19276	-4108.26960	-4108.73948	-4108.56997	-4109.04242	-4108.94974	-4109.41592	-4109.81522	-4110.33361
9.45	-4106.19262	-4108.26933	-4108.73921	-4108.56975	-4109.04219	-4108.94956	-4109.41574	-4109.81510	-4110.33347
9.54	-4106.19249	-4108.26909	-4108.73897	-4108.56955	-4109.04199	-4108.94940	-4109.41559	-4109.81500	-4110.33335
9.64	-4106.19238	-4108.26888	-4108.73876	-4108.56939	-4109.04181	-4108.94926	-4109.41545	-4109.81491	-4110.33325
9.73	-4106.19227	-4108.26868	-4108.73855	-4108.56923	-4109.04164	-4108.94914	-4109.41533	-4109.81484	-4110.33316
9.83	-4106.19217	-4108.26851	-4108.73838	-4108.56909	-4109.04150	-4108.94903	-4109.41522	-4109.81478	-4110.33309
9.92	-4106.19209	-4108.26835	-4108.73822	-4108.56898	-4109.04137	-4108.94893	-4109.41512	-4109.81474	-4110.33303
10.02	-4106.19200	-4108.26821	-4108.73807	-4108.56887	-4109.04126	-4108.94885	-4109.41504	-4109.81470	-4110.33299
10.11	-4106.19193	-4108.26808	-4108.73794	-4108.56878	-4109.04116	-4108.94877	-4109.41496	-4109.81467	-4110.33295
10.20	-4106.19186	-4108.26796	-4108.73782	-4108.56869	-4109.04107	-4108.94871	-4109.41489	-4109.81465	-4110.33291
10.30	-4106.19180	-4108.26786	-4108.73771	-4108.56862	-4109.04099	-4108.94865	-4109.41483	-4109.81463	-4110.33288
10.39	-4106.19174	-4108.26776	-4108.73761	-4108.56856	-4109.04092	-4108.94860	-4109.41478	-4109.81462	-4110.33286
10.49	-4106.19169	-4108.26768	-4108.73752	-4108.56850	-4109.04085	-4108.94856	-4109.41473	-4109.81462	-4110.33283
10.58	-4106.19164	-4108.26760	-4108.73744	-4108.56845	-4109.04080	-4108.94852	-4109.41469	-4109.81461	-4110.33282
10.68	-4106.19159	-4108.26753	-4108.73737	-4108.56841	-4109.04075	-4108.94849	-4109.41466	-4109.81462	-4110.33280
10.77	-4106.19155	-4108.26747	-4108.73730	-4108.56837	-4109.04070	-4108.94847	-4109.41463	-4109.81462	-4110.33279
10.87	-4106.19151	-4108.26741	-4108.73723	-4108.56834	-4109.04066	-4108.94845	-4109.41460	-4109.81462	-4110.33278
10.96	-4106.19147	-4108.26736	-4108.73718	-4108.56831	-4109.04062	-4108.94843	-4109.41458	-4109.81462	-4110.33278
11.05	-4106.19143	-4108.26731	-4108.73712	-4108.56828	-4109.04058	-4108.94841	-4109.41456	-4109.81463	-4110.33277
11.15	-4106.19140	-4108.26727	-4108.73708	-4108.56826	-4109.04055	-4108.94840	-4109.41454	-4109.81463	-4110.33277
11.24	-4106.19137	-4108.26723	-4108.73703	-4108.56824	-4109.04053	-4108.94839	-4109.41453	-4109.81464	-4110.33276
11.34	-4106.19134	-4108.26719	-4108.73699	-4108.56822	-4109.04050	-4108.94838	-4109.41451	-4109.81464	-4110.33276
11.43	-4106.19132	-4108.26716	-4108.73696	-4108.56820	-4109.04048	-4108.94837	-4109.41450	-4109.81465	-4110.33275
11.53	-4106.19129	-4108.26713	-4108.73692	-4108.56818	-4109.04046	-4108.94837	-4109.41450	-4109.81465	-4110.33275
11.62	-4106.19127	-4108.26710	-4108.73689	-4108.56817	-4109.04044	-4108.94837	-4109.41449	-4109.81466	-4110.33275
11.72	-4106.19125	-4108.26708	-4108.73687	-4108.56816	-4109.04043	-4108.94837	-4109.41449	-4109.81467	-4110.33275
11.81	-4106.19123	-4108.26705	-4108.73684	-4108.56815	-4109.04041	-4108.94837	-4109.41449	-4109.81468	-4110.33274
11.91	-4106.19121	-4108.26703	-4108.73682	-4108.56814	-4109.04040	-4108.94837	-4109.41449	-4109.81468	-4110.33274
12.00	-4106.19119	-4108.26702	-4108.73680	-4108.56813	-4109.04039	-4108.94837	-4109.41449	-4109.81469	-4110.33274
12.09	-4106.19117	-4108.26700	-4108.73678	-4108.56813	-4109.04037	-4108.94837	-4109.41449	-4109.81470	-4110.33274
12.19	-4106.19116	-4108.26698	-4108.73676	-4108.56812	-4109.04036	-4108.94838	-4109.41449	-4109.81470	-4110.33274
12.28	-4106.19114	-4108.26697	-4108.73674	-4108.56811	-4109.04035	-4108.94838	-4109.41449	-4109.81470	-4110.33273
12.38	-4106.19113	-4108.26695	-4108.73672	-4108.56810	-4109.04034	-4108.94839	-4109.41449	-4109.81470	-4110.33273
12.47	-4106.19112	-4108.26694	-4108.73671	-4108.56810	-4109.04033	-4108.94839	-4109.41449	-4109.81471	-4110.33273
12.57	-4106.19110	-4108.26693	-4108.73669	-4108.56809	-4109.04032	-4108.94840	-4109.41449	-4109.81471	-4110.33272
12.66	-4106.19109	-4108.26691	-4108.73668	-4108.56809	-4109.04031	-4108.94840	-4109.41450	-4109.81471	-4110.33272
12.76	-4106.19108	-4108.26690	-4108.73667	-4108.56808	-4109.04030	-4108.94840	-4109.41450	-4109.81471	-4110.33271
12.85	-4106.19107	-4108.26689	-4108.73666	-4108.56808	-4109.04030	-4108.94841	-4109.41450	-4109.81471	-4110.33271
12.94	-4106.19106	-4108.26689	-4108.73665	-4108.56807	-4109.04029	-4108.94841	-4109.41451	-4109.81471	-4110.33271

Table S19: The absolute energies in hartree of the potential energy curve of NbSi in the $^6\Sigma^+$ state with a SP-based wave function and nom-CPO active space.

Distance	SP	tpbe	ftpbe	trevpbe	ftrevpbe	tblyp	ftblyp	torelyp	ftorelyp
13.04	-4106.19105	-4108.26688	-4108.73664	-4108.56807	-4109.04029	-4108.94842	-4109.41451	-4109.81472	-4110.33271
13.13	-4106.19104	-4108.26687	-4108.73664	-4108.56807	-4109.04028	-4108.94842	-4109.41452	-4109.81472	-4110.33271
13.23	-4106.19103	-4108.26687	-4108.73663	-4108.56807	-4109.04028	-4108.94843	-4109.41453	-4109.81472	-4110.33271
13.32	-4106.19102	-4108.26686	-4108.73662	-4108.56806	-4109.04028	-4108.94844	-4109.41453	-4109.81472	-4110.33271
13.42	-4106.19101	-4108.26686	-4108.73662	-4108.56806	-4109.04027	-4108.94845	-4109.41454	-4109.81473	-4110.33271
13.51	-4106.19101	-4108.26685	-4108.73661	-4108.56806	-4109.04027	-4108.94845	-4109.41454	-4109.81473	-4110.33271
13.61	-4106.19100	-4108.26685	-4108.73661	-4108.56806	-4109.04026	-4108.94846	-4109.41455	-4109.81473	-4110.33270
13.70	-4106.19099	-4108.26684	-4108.73660	-4108.56805	-4109.04026	-4108.94846	-4109.41455	-4109.81473	-4110.33270
13.80	-4106.19099	-4108.26683	-4108.73659	-4108.56805	-4109.04025	-4108.94846	-4109.41455	-4109.81473	-4110.33270
13.89	-4106.19098	-4108.26683	-4108.73659	-4108.56805	-4109.04025	-4108.94847	-4109.41456	-4109.81473	-4110.33269
13.98	-4106.19097	-4108.26682	-4108.73658	-4108.56804	-4109.04024	-4108.94847	-4109.41456	-4109.81472	-4110.33269
14.08	-4106.19097	-4108.26682	-4108.73658	-4108.56804	-4109.04024	-4108.94847	-4109.41456	-4109.81472	-4110.33268
14.17	-4106.19096	-4108.26682	-4108.73657	-4108.56804	-4109.04023	-4108.94848	-4109.41456	-4109.81472	-4110.33268
14.27	-4106.19096	-4108.26681	-4108.73657	-4108.56804	-4109.04023	-4108.94848	-4109.41457	-4109.81472	-4110.33268
14.36	-4106.19095	-4108.26681	-4108.73657	-4108.56803	-4109.04023	-4108.94848	-4109.41457	-4109.81472	-4110.33268
14.46	-4106.19095	-4108.26681	-4108.73656	-4108.56803	-4109.04023	-4108.94849	-4109.41458	-4109.81472	-4110.33268
14.55	-4106.19094	-4108.26681	-4108.73656	-4108.56803	-4109.04023	-4108.94849	-4109.41458	-4109.81472	-4110.33268
14.65	-4106.19094	-4108.26680	-4108.73656	-4108.56803	-4109.04023	-4108.94850	-4109.41459	-4109.81472	-4110.33268
14.74	-4106.19093	-4108.26680	-4108.73656	-4108.56803	-4109.04023	-4108.94850	-4109.41459	-4109.81472	-4110.33268
14.83	-4106.19093	-4108.26680	-4108.73656	-4108.56803	-4109.04022	-4108.94851	-4109.41459	-4109.81472	-4110.33268
14.93	-4106.19093	-4108.26680	-4108.73656	-4108.56803	-4109.04022	-4108.94851	-4109.41460	-4109.81472	-4110.33268
15.02	-4106.19092	-4108.26680	-4108.73656	-4108.56803	-4109.04022	-4108.94851	-4109.41460	-4109.81473	-4110.33268
15.12	-4106.19092	-4108.26680	-4108.73656	-4108.56803	-4109.04022	-4108.94852	-4109.41461	-4109.81473	-4110.33268
15.21	-4106.19092	-4108.26680	-4108.73655	-4108.56803	-4109.04022	-4108.94852	-4109.41461	-4109.81473	-4110.33268
15.31	-4106.19091	-4108.26680	-4108.73655	-4108.56803	-4109.04022	-4108.94852	-4109.41461	-4109.81473	-4110.33267
15.40	-4106.19091	-4108.26680	-4108.73655	-4108.56803	-4109.04022	-4108.94852	-4109.41461	-4109.81472	-4110.33267
15.50	-4106.19091	-4108.26679	-4108.73655	-4108.56802	-4109.04021	-4108.94853	-4109.41461	-4109.81472	-4110.33267
15.59	-4106.19091	-4108.26679	-4108.73655	-4108.56802	-4109.04021	-4108.94853	-4109.41462	-4109.81472	-4110.33267
15.68	-4106.19091	-4108.26679	-4108.73654	-4108.56802	-4109.04021	-4108.94853	-4109.41462	-4109.81472	-4110.33267
15.78	-4106.19091	-4108.26679	-4108.73654	-4108.56802	-4109.04021	-4108.94853	-4109.41462	-4109.81472	-4110.33267
15.87	-4106.19091	-4108.26679	-4108.73654	-4108.56802	-4109.04021	-4108.94853	-4109.41462	-4109.81472	-4110.33266
15.97	-4106.19091	-4108.26678	-4108.73654	-4108.56802	-4109.04021	-4108.94853	-4109.41462	-4109.81472	-4110.33266
16.06	-4106.19091	-4108.26678	-4108.73654	-4108.56802	-4109.04020	-4108.94853	-4109.41462	-4109.81472	-4110.33266
16.16	-4106.19091	-4108.26678	-4108.73654	-4108.56802	-4109.04020	-4108.94854	-4109.41463	-4109.81472	-4110.33266
16.25	-4106.19091	-4108.26678	-4108.73654	-4108.56802	-4109.04020	-4108.94854	-4109.41463	-4109.81472	-4110.33266
16.35	-4106.19091	-4108.26678	-4108.73654	-4108.56802	-4109.04020	-4108.94854	-4109.41463	-4109.81472	-4110.33266
16.44	-4106.19091	-4108.26678	-4108.73654	-4108.56802	-4109.04020	-4108.94854	-4109.41463	-4109.81472	-4110.33266
16.54	-4106.19091	-4108.26678	-4108.73654	-4108.56802	-4109.04020	-4108.94855	-4109.41463	-4109.81472	-4110.33266
16.63	-4106.19091	-4108.26678	-4108.73654	-4108.56802	-4109.04020	-4108.94855	-4109.41464	-4109.81472	-4110.33266
16.72	-4106.19091	-4108.26678	-4108.73654	-4108.56802	-4109.04020	-4108.94855	-4109.41464	-4109.81472	-4110.33266
16.82	-4106.19091	-4108.26679	-4108.73654	-4108.56802	-4109.04021	-4108.94855	-4109.41464	-4109.81472	-4110.33266
16.91	-4106.19091	-4108.26679	-4108.73654	-4108.56802	-4109.04021	-4108.94856	-4109.41464	-4109.81472	-4110.33267
17.01	-4106.19091	-4108.26679	-4108.73654	-4108.56802	-4109.04021	-4108.94856	-4109.41465	-4109.81472	-4110.33267
17.10	-4106.19091	-4108.26679	-4108.73654	-4108.56802	-4109.04021	-4108.94856	-4109.41465	-4109.81473	-4110.33267
17.20	-4106.19091	-4108.26679	-4108.73655	-4108.56802	-4109.04021	-4108.94856	-4109.41465	-4109.81473	-4110.33267
17.29	-4106.19091	-4108.26679	-4108.73655	-4108.56802	-4109.04021	-4108.94857	-4109.41465	-4109.81473	-4110.33267
17.39	-4106.19091	-4108.26679	-4108.73655	-4108.56802	-4109.04021	-4108.94857	-4109.41466	-4109.81473	-4110.33267
17.48	-4106.19091	-4108.26679	-4108.73655	-4108.56802	-4109.04021	-4108.94857	-4109.41466	-4109.81473	-4110.33267
17.57	-4106.19091	-4108.26679	-4108.73655	-4108.56802	-4109.04021	-4108.94857	-4109.41466	-4109.81473	-4110.33267
17.67	-4106.19091	-4108.26679	-4108.73655	-4108.56802	-4109.04021	-4108.94857	-4109.41466	-4109.81473	-4110.33267
17.76	-4106.19091	-4108.26679	-4108.73654	-4108.56802	-4109.04021	-4108.94857	-4109.41466	-4109.81473	-4110.33267
17.86	-4106.19091	-4108.26679	-4108.73654	-4108.56802	-4109.04021	-4108.94857	-4109.41466	-4109.81473	-4110.33267

Table S19: The absolute energies in hartree of the potential energy curve of NbSi in the $^6\Sigma^+$ state with a SP-based wave function and nom-CPO active space.

Distance	SP	tpbe	ftpbe	trevpbe	ftrevpbe	tblyp	ftblyp	torelyp	ftorelyp
17.95	-4106.19091	-4108.26679	-4108.73654	-4108.56802	-4109.04021	-4108.94857	-4109.41466	-4109.81472	-4110.33266
18.05	-4106.19091	-4108.26679	-4108.73654	-4108.56802	-4109.04020	-4108.94857	-4109.41466	-4109.81472	-4110.33266

Table S20: The absolute energies in hartree of the potential energy curve of TaSi in the $^4\Pi$ state with a CASSCF wave function and nom-CPO active space.

Distance	CASSCF	CASPT2	tpbe	ftpbe	trevpbe	ftrevpbe	tblyp	ftblyp	torelyp	fto
1.98	-15875.66545	-15875.52591	-13624.13035	-15882.53475	-15881.82797	-15883.03563	-15882.39855	-15883.59737	-15886.75513	-1588
2.08	-15876.38964	-15876.42485	-14809.65790	-15883.24327	-15882.53853	-15883.74553	-15883.11082	-15884.30908	-15887.45922	-1588
2.17	-15876.96475	-15877.13780	-15281.04072	-15883.80324	-15883.10039	-15884.30579	-15883.67701	-15884.87469	-15888.01824	-1588
2.27	-15877.42742	-15877.70559	-15517.42338	-15884.25151	-15883.55402	-15884.75489	-15884.13140	-15885.32559	-15888.46661	-1588
2.36	-15877.79631	-15878.16106	-15650.97622	-15884.61174	-15883.91488	-15885.11626	-15884.49280	-15885.68717	-15888.82284	-1589
2.46	-15878.09015	-15878.52289	-15728.94442	-15884.90017	-15884.20237	-15885.40578	-15884.78089	-15885.97645	-15889.10616	-1589
2.55	-15878.32512	-15878.81062	-15776.98372	-15885.13205	-15884.43397	-15885.63865	-15885.01367	-15886.20896	-15889.33386	-1589
2.65	-15878.51399	-15879.04069	-15808.35744	-15885.32544	-15884.62979	-15885.83247	-15885.21041	-15886.40574	-15889.52471	-1589
2.74	-15878.66670	-15879.22994	-15829.73166	-15885.49361	-15884.79826	-15886.00131	-15885.37916	-15886.57501	-15889.69135	-1589
2.83	-15878.79086	-15879.39793	-15844.46597	-15885.63263	-15884.93743	-15886.14094	-15885.51853	-15886.71504	-15889.82901	-1589
2.93	-15878.89232	-15879.53659	-15854.70028	-15885.74789	-15885.05291	-15886.25677	-15885.63413	-15886.83121	-15889.94319	-1589
3.02	-15878.97552	-15879.65164	-15861.90270	-15885.84381	-15885.14966	-15886.35325	-15885.73092	-15886.92796	-15890.03871	-1589
3.12	-15879.04410	-15879.74782	-15867.08246	-15885.92798	-15885.23633	-15886.44046	-15885.81575	-15887.00743	-15890.12472	-1589
3.21	-15879.10251	-15879.82526	-15870.92758	-15886.00099	-15885.30937	-15886.51416	-15885.88816	-15887.08051	-15890.19544	-1589
3.31	-15879.15201	-15879.89602	-15873.84000	-15886.06198	-15885.37100	-15886.57566	-15885.94960	-15887.14182	-15890.25531	-1589
3.40	-15879.25588	-15879.95596	-15876.10216	-15886.11257	-15885.42228	-15886.62671	-15886.00085	-15887.19279	-15890.30514	-1589
3.50	-15879.29878	-15880.00590	-15877.88824	-15886.15427	-15885.46459	-15886.66882	-15886.04320	-15887.23489	-15890.34617	-1589
3.59	-15879.33402	-15880.04706	-15879.30758	-15886.18826	-15885.49920	-15886.70320	-15886.07790	-15887.26931	-15890.37964	-1589
3.68	-15879.36269	-15880.08065	-15880.43576	-15886.21569	-15885.52729	-15886.73101	-15886.10611	-15887.29717	-15890.40670	-1589
3.78	-15879.38573	-15880.10775	-15881.33076	-15886.23764	-15885.54978	-15886.75331	-15886.12875	-15887.31954	-15890.42826	-1589
3.87	-15879.40397	-15880.12930	-15882.03993	-15886.25490	-15885.56751	-15886.77089	-15886.14667	-15887.33723	-15890.44517	-1589
3.97	-15879.41813	-15880.14612	-15882.60064	-15886.26812	-15885.58124	-15886.78442	-15886.16060	-15887.35087	-15890.45816	-1589
4.06	-15879.42883	-15880.15893	-15883.05309	-15886.27796	-15885.59157	-15886.79455	-15886.17115	-15887.36113	-15890.46782	-1589
4.16	-15879.43823	-15880.16835	-15883.41286	-15886.28500	-15885.59901	-15886.80187	-15886.17884	-15887.36858	-15890.47468	-1589
4.25	-15879.44381	-15880.18131	-15883.69944	-15886.28968	-15885.60708	-15886.80680	-15886.18746	-15887.37367	-15890.48176	-1589
4.35	-15879.44734	-15880.18186	-15883.93043	-15886.29232	-15885.60839	-15886.80969	-15886.18907	-15887.37672	-15890.48266	-1589
4.44	-15879.44915	-15880.18106	-15884.12588	-15886.29327	-15885.60831	-15886.81087	-15886.18930	-15887.37806	-15890.48221	-1589
4.54	-15879.44955	-15880.17916	-15884.29359	-15886.29286	-15885.60709	-15886.81068	-15886.18841	-15887.37803	-15890.48065	-1589
4.63	-15879.44881	-15880.17638	-15884.43202	-15886.29137	-15885.60493	-15886.80941	-15886.18658	-15887.37690	-15890.47817	-1589
4.72	-15879.44714	-15880.17291	-15884.54679	-15886.28898	-15885.60203	-15886.80723	-15886.18399	-15887.37485	-15890.47496	-1589
4.82	-15879.44474	-15880.16889	-15884.64286	-15886.28583	-15885.59854	-15886.80430	-15886.18083	-15887.37201	-15890.47118	-1589
4.91	-15879.44178	-15880.16446	-15884.72832	-15886.28211	-15885.59459	-15886.80078	-15886.17718	-15887.36857	-15890.46694	-1589
5.01	-15879.43840	-15880.15973	-15884.80083	-15886.27795	-15885.59028	-15886.79684	-15886.17315	-15887.36465	-15890.46232	-1589
5.10	-15879.43473	-15880.15478	-15884.86204	-15886.27348	-15885.58566	-15886.79258	-15886.16880	-15887.36037	-15890.45739	-1589
5.20	-15879.43087	-15880.14968	-15884.91291	-15886.26874	-15885.58083	-15886.78808	-15886.16420	-15887.35579	-15890.45223	-1589
5.29	-15879.42692	-15880.14448	-15884.95483	-15886.26378	-15885.57581	-15886.78335	-15886.15937	-15887.35093	-15890.44687	-1589
5.39	-15879.42296	-15880.13922	-15884.98908	-15886.25864	-15885.57062	-15886.77846	-15886.15433	-15887.34584	-15890.44131	-1589
5.48	-15879.41905	-15880.13391	-15885.01682	-15886.25337	-15885.56523	-15886.77344	-15886.14903	-15887.34056	-15890.43553	-1589
5.57	-15879.41525	-15880.12850	-15885.03898	-15886.24799	-15885.55952	-15886.76833	-15886.14333	-15887.33514	-15890.42938	-1589
5.67	-15879.41161	-15880.12288	-15885.05640	-15886.24254	-15885.55314	-15886.76316	-15886.13688	-15887.32963	-15890.42250	-1589
5.76	-15879.40821	-15880.11758	-15885.06984	-15886.23703	-15885.54690	-15886.75796	-15886.13061	-15887.32406	-15890.41587	-1589
5.86	-15879.40507	-15880.11300	-15885.07989	-15886.23152	-15885.54166	-15886.75271	-15886.12547	-15887.31854	-15890.41044	-1589
5.95	-15879.40216	-15880.10853	-15885.08708	-15886.22619	-15885.53650	-15886.74762	-15886.12046	-15887.31326	-15890.40520	-1589
6.05	-15879.39945	-15880.10467	-15885.09469	-15886.22121	-15885.53243	-15886.74285	-15886.11666	-15887.30842	-15890.40116	-1589

Table S20: The absolute energies in hartree of the potential energy curve of TaSi in the $^4\Pi$ state with a CASSCF wave function and nom-CPO active space.

Distance	CASSCF	CASPT2	tpbe	ftpbe	trevpbe	ftrevpbe	tblyp	ftblyp	torelyp	fto
6.14	-15879.39697	-15880.10139	-15885.09578	-15886.21671	-15885.52923	-15886.73855	-15886.11378	-15887.30416	-15890.39803	-1589
6.24	-15879.39466	-15880.09834	-15885.09551	-15886.21282	-15885.52638	-15886.73481	-15886.11131	-15887.30055	-15890.39530	-1589
6.33	-15879.39250	-15880.09550	-15885.09410	-15886.20949	-15885.52379	-15886.73160	-15886.10917	-15887.29758	-15890.39288	-1589
6.43	-15879.39051	-15880.09288	-15885.09178	-15886.20659	-15885.52141	-15886.72880	-15886.10726	-15887.29509	-15890.39069	-1589
6.52	-15879.38870	-15880.09049	-15885.08871	-15886.20403	-15885.51918	-15886.72632	-15886.10552	-15887.29298	-15890.38869	-1589
6.61	-15879.38707	-15880.08832	-15885.08508	-15886.20174	-15885.51709	-15886.72409	-15886.10391	-15887.29119	-15890.38683	-1589
6.71	-15879.38561	-15880.08634	-15885.08099	-15886.19970	-15885.51512	-15886.72210	-15886.10240	-15887.28969	-15890.38509	-1589
6.80	-15879.38430	-15880.08455	-15885.07654	-15886.19786	-15885.51325	-15886.72030	-15886.10097	-15887.28842	-15890.38346	-1589
6.90	-15879.38314	-15880.08293	-15885.07180	-15886.19620	-15885.51150	-15886.71866	-15886.09963	-15887.28735	-15890.38194	-1589
6.99	-15879.38210	-15880.08148	-15885.06684	-15886.19467	-15885.50987	-15886.71716	-15886.09838	-15887.28643	-15890.38053	-1589
7.09	-15879.38118	-15880.08015	-15885.06170	-15886.19326	-15885.50834	-15886.71576	-15886.09719	-15887.28564	-15890.37922	-1589
7.18	-15879.38037	-15880.07896	-15885.05638	-15886.19193	-15885.50694	-15886.71445	-15886.09610	-15887.28493	-15890.37803	-1589
7.28	-15879.37965	-15880.07787	-15885.05085	-15886.19066	-15885.50565	-15886.71320	-15886.09509	-15887.28427	-15890.37695	-1589
7.37	-15879.37902	-15880.07689	-15885.04500	-15886.18944	-15885.50448	-15886.71201	-15886.09416	-15887.28364	-15890.37597	-1589
7.46	-15879.37845	-15880.07600	-15885.03845	-15886.18828	-15885.50341	-15886.71087	-15886.09331	-15887.28304	-15890.37509	-1589
7.56	-15879.37796	-15880.07519	-15885.03205	-15886.18716	-15885.50244	-15886.70977	-15886.09254	-15887.28244	-15890.37430	-1589
7.65	-15879.37751	-15880.07445	-15885.02668	-15886.18609	-15885.50156	-15886.70873	-15886.09183	-15887.28186	-15890.37360	-1589
7.75	-15879.37712	-15880.07376	-15885.02138	-15886.18507	-15885.50078	-15886.70775	-15886.09119	-15887.28129	-15890.37299	-1589
7.84	-15879.37677	-15880.07314	-15885.01718	-15886.18412	-15885.50008	-15886.70684	-15886.09062	-15887.28075	-15890.37245	-1589
7.94	-15879.37646	-15880.07256	-15885.01387	-15886.18322	-15885.49945	-15886.70598	-15886.09010	-15887.28022	-15890.37198	-1589
8.03	-15879.37619	-15880.07202	-15885.01091	-15886.18232	-15885.49888	-15886.70512	-15886.08964	-15887.27968	-15890.37157	-1589
8.13	-15879.37594	-15880.07153	-15885.00823	-15886.18154	-15885.49838	-15886.70438	-15886.08922	-15887.27920	-15890.37121	-1589
8.22	-15879.37572	-15880.07108	-15885.00575	-15886.18090	-15885.49794	-15886.70380	-15886.08885	-15887.27881	-15890.37090	-1589
8.31	-15879.37552	-15880.07066	-15885.00344	-15886.18034	-15885.49754	-15886.70329	-15886.08852	-15887.27846	-15890.37064	-1589
8.41	-15879.37534	-15880.07028	-15885.00126	-15886.17984	-15885.49719	-15886.70284	-15886.08822	-15887.27815	-15890.37042	-1589
8.50	-15879.37518	-15880.06992	-15884.99920	-15886.17939	-15885.49689	-15886.70245	-15886.08796	-15887.27788	-15890.37023	-1589
8.60	-15879.37504	-15880.06960	-15884.99726	-15886.17898	-15885.49661	-15886.70209	-15886.08773	-15887.27762	-15890.37008	-1589
8.69	-15879.37491	-15880.06930	-15884.99542	-15886.17861	-15885.49637	-15886.70177	-15886.08752	-15887.27740	-15890.36995	-1589
8.79	-15879.37479	-15880.06903	-15884.99371	-15886.17828	-15885.49616	-15886.70149	-15886.08734	-15887.27720	-15890.36984	-1589
8.88	-15879.37469	-15880.06878	-15884.99210	-15886.17799	-15885.49597	-15886.70125	-15886.08717	-15887.27703	-15890.36975	-1589
8.98	-15879.37459	-15880.06855	-15884.99062	-15886.17773	-15885.49581	-15886.70103	-15886.08703	-15887.27687	-15890.36968	-1589
9.07	-15879.37450	-15880.06834	-15884.98925	-15886.17748	-15885.49566	-15886.70084	-15886.08690	-15887.27673	-15890.36962	-1589
9.17	-15879.37442	-15880.06815	-15884.98799	-15886.17727	-15885.49554	-15886.70067	-15886.08679	-15887.27661	-15890.36958	-1589
9.26	-15879.37435	-15880.06797	-15884.98684	-15886.17708	-15885.49543	-15886.70052	-15886.08669	-15887.27650	-15890.36955	-1589
9.35	-15879.37429	-15880.06780	-15884.98579	-15886.17691	-15885.49533	-15886.70040	-15886.08660	-15887.27641	-15890.36953	-1589
9.45	-15879.37423	-15880.06765	-15884.98484	-15886.17676	-15885.49525	-15886.70028	-15886.08654	-15887.27632	-15890.36951	-1589
9.54	-15879.37417	-15880.06751	-15884.98398	-15886.17662	-15885.49517	-15886.70018	-15886.08647	-15887.27625	-15890.36950	-1589
9.64	-15879.37412	-15880.06738	-15884.98320	-15886.17649	-15885.49511	-15886.70009	-15886.08641	-15887.27619	-15890.36950	-1589
9.73	-15879.37408	-15880.06726	-15884.98249	-15886.17638	-15885.49505	-15886.70001	-15886.08636	-15887.27613	-15890.36950	-1589
9.83	-15879.37404	-15880.06715	-15884.98186	-15886.17627	-15885.49501	-15886.69994	-15886.08632	-15887.27608	-15890.36950	-1589
9.92	-15879.37400	-15880.06705	-15884.98129	-15886.17618	-15885.49496	-15886.69988	-15886.08629	-15887.27604	-15890.36951	-1589
10.02	-15879.37396	-15880.06695	-15884.98077	-15886.17610	-15885.49493	-15886.69983	-15886.08626	-15887.27601	-15890.36952	-1589
10.11	-15879.37393	-15880.06686	-15884.98031	-15886.17602	-15885.49490	-15886.69978	-15886.08623	-15887.27598	-15890.36953	-1589
10.20	-15879.37390	-15880.06678	-15884.97989	-15886.17596	-15885.49487	-15886.69975	-15886.08621	-15887.27595	-15890.36954	-1589
10.30	-15879.37388	-15880.06670	-15884.97952	-15886.17590	-15885.49485	-15886.69972	-15886.08619	-15887.27593	-15890.36955	-1589
10.39	-15879.37385	-15880.06664	-15884.97918	-15886.17585	-15885.49483	-15886.69969	-15886.08618	-15887.27592	-15890.36956	-1589
10.49	-15879.37383	-15880.06657	-15884.97888	-15886.17580	-15885.49481	-15886.69967	-15886.08617	-15887.27591	-15890.36957	-1589
10.58	-15879.37381	-15880.06651	-15884.97861	-15886.17576	-15885.49479	-15886.69965	-15886.08616	-15887.27590	-15890.36958	-1589
10.68	-15879.37379	-15880.06645	-15884.97837	-15886.17572	-15885.49478	-15886.69963	-15886.08616	-15887.27589	-15890.36959	-1589
10.77	-15879.37377	-15880.06640	-15884.97815	-15886.17568	-15885.49477	-15886.69961	-15886.08615	-15887.27588	-15890.36960	-1589
10.87	-15879.37375	-15880.06635	-15884.97795	-15886.17565	-15885.49475	-15886.69959	-15886.08614	-15887.27588	-15890.36961	-1589
10.96	-15879.37374	-15880.06630	-15884.97778	-15886.17561	-15885.49475	-15886.69957	-15886.08615	-15887.27587	-15890.36962	-1589

Table S20: The absolute energies in hartree of the potential energy curve of TaSi in the $^4\Pi$ state with a CASSCF wave function and nom-CPO active space.

Distance	CASSCF	CASPT2	tpbe	ftpbe	trevpbe	ftrevpbe	tblyp	ftblyp	torelyp	fto
11.05	-15879.37373	-15880.06626	-15884.97762	-15886.17558	-15885.49474	-15886.69955	-15886.08614	-15887.27587	-15890.36963	-1589
11.15	-15879.37371	-15880.06622	-15884.97748	-15886.17555	-15885.49473	-15886.69954	-15886.08615	-15887.27587	-15890.36963	-1589
11.24	-15879.37370	-15880.06618	-15884.97736	-15886.17553	-15885.49472	-15886.69953	-15886.08615	-15887.27587	-15890.36964	-1589
11.34	-15879.37369	-15880.06615	-15884.97724	-15886.17551	-15885.49471	-15886.69952	-15886.08615	-15887.27587	-15890.36965	-1589
11.43	-15879.37368	-15880.06612	-15884.97714	-15886.17549	-15885.49471	-15886.69951	-15886.08615	-15887.27588	-15890.36965	-1589
11.53	-15879.37367	-15880.06609	-15884.97704	-15886.17547	-15885.49470	-15886.69951	-15886.08616	-15887.27588	-15890.36966	-1589
11.62	-15879.37366	-15880.06606	-15884.97696	-15886.17546	-15885.49470	-15886.69951	-15886.08616	-15887.27589	-15890.36966	-1589
11.72	-15879.37365	-15880.06603	-15884.97688	-15886.17545	-15885.49470	-15886.69950	-15886.08616	-15887.27589	-15890.36966	-1589
11.81	-15879.37365	-15880.06600	-15884.97682	-15886.17544	-15885.49469	-15886.69950	-15886.08617	-15887.27590	-15890.36966	-1589
11.91	-15879.37364	-15880.06598	-15884.97676	-15886.17543	-15885.49469	-15886.69950	-15886.08617	-15887.27591	-15890.36967	-1589
12.00	-15879.37363	-15880.06596	-15884.97670	-15886.17542	-15885.49468	-15886.69949	-15886.08618	-15887.27592	-15890.36967	-1589
12.09	-15879.37363	-15880.06594	-15884.97665	-15886.17540	-15885.49468	-15886.69948	-15886.08618	-15887.27592	-15890.36967	-1589
12.19	-15879.37362	-15880.06592	-15884.97661	-15886.17539	-15885.49468	-15886.69948	-15886.08619	-15887.27592	-15890.36967	-1589
12.28	-15879.37361	-15880.06590	-15884.97657	-15886.17538	-15885.49468	-15886.69947	-15886.08619	-15887.27593	-15890.36967	-1589
12.38	-15879.37361	-15880.06588	-15884.97653	-15886.17537	-15885.49467	-15886.69946	-15886.08620	-15887.27593	-15890.36967	-1589
12.47	-15879.37361	-15880.06587	-15884.97649	-15886.17536	-15885.49467	-15886.69946	-15886.08621	-15887.27594	-15890.36967	-1589
12.57	-15879.37360	-15880.06585	-15884.97646	-15886.17535	-15885.49467	-15886.69946	-15886.08621	-15887.27594	-15890.36967	-1589
12.66	-15879.37360	-15880.06584	-15884.97643	-15886.17534	-15885.49467	-15886.69945	-15886.08622	-15887.27595	-15890.36967	-1589
12.76	-15879.37359	-15880.06550	-15884.97641	-15886.17534	-15885.49466	-15886.69945	-15886.08622	-15887.27595	-15890.36967	-1589
12.85	-15879.37359	-15880.06548	-15884.97639	-15886.17533	-15885.49466	-15886.69945	-15886.08622	-15887.27596	-15890.36966	-1589
12.94	-15879.37359	-15880.06547	-15884.97637	-15886.17533	-15885.49465	-15886.69945	-15886.08622	-15887.27596	-15890.36966	-1589
13.04	-15879.37358	-15880.06546	-15884.97634	-15886.17533	-15885.49465	-15886.69945	-15886.08623	-15887.27597	-15890.36966	-1589
13.13	-15879.37358	-15880.06545	-15884.97632	-15886.17533	-15885.49465	-15886.69945	-15886.08623	-15887.27598	-15890.36966	-1589
13.23	-15879.37358	-15880.06544	-15884.97631	-15886.17532	-15885.49465	-15886.69944	-15886.08624	-15887.27599	-15890.36966	-1589
13.32	-15879.37358	-15880.06543	-15884.97629	-15886.17532	-15885.49464	-15886.69944	-15886.08624	-15887.27599	-15890.36966	-1589
13.42	-15879.37357	-15880.06542	-15884.97628	-15886.17532	-15885.49465	-15886.69944	-15886.08625	-15887.27600	-15890.36966	-1589
13.51	-15879.37357	-15880.06541	-15884.97627	-15886.17531	-15885.49464	-15886.69944	-15886.08625	-15887.27600	-15890.36965	-1589
13.61	-15879.37357	-15880.06540	-15884.97625	-15886.17531	-15885.49464	-15886.69944	-15886.08625	-15887.27601	-15890.36965	-1589
13.70	-15879.37357	-15880.06540	-15884.97624	-15886.17531	-15885.49464	-15886.69943	-15886.08625	-15887.27601	-15890.36965	-1589
13.80	-15879.37356	-15880.06539	-15884.97623	-15886.17530	-15885.49464	-15886.69943	-15886.08626	-15887.27601	-15890.36965	-1589
13.89	-15879.37356	-15880.06538	-15884.97622	-15886.17530	-15885.49463	-15886.69943	-15886.08626	-15887.27601	-15890.36965	-1589
13.98	-15879.37356	-15880.06537	-15884.97622	-15886.17530	-15885.49463	-15886.69942	-15886.08626	-15887.27602	-15890.36965	-1589
14.08	-15879.37356	-15880.06537	-15884.97621	-15886.17529	-15885.49463	-15886.69942	-15886.08626	-15887.27602	-15890.36964	-1589
14.17	-15879.37356	-15880.06536	-15884.97620	-15886.17529	-15885.49463	-15886.69942	-15886.08627	-15887.27602	-15890.36964	-1589
14.27	-15879.37356	-15880.06536	-15884.97619	-15886.17529	-15885.49463	-15886.69942	-15886.08627	-15887.27602	-15890.36965	-1589
14.36	-15879.37356	-15880.06535	-15884.97619	-15886.17529	-15885.49463	-15886.69942	-15886.08628	-15887.27603	-15890.36965	-1589
14.46	-15879.37355	-15880.06535	-15884.97618	-15886.17529	-15885.49463	-15886.69942	-15886.08628	-15887.27603	-15890.36964	-1589
14.55	-15879.37355	-15880.06534	-15884.97618	-15886.17528	-15885.49463	-15886.69942	-15886.08628	-15887.27603	-15890.36964	-1589
14.65	-15879.37355	-15880.06533	-15884.97617	-15886.17528	-15885.49463	-15886.69942	-15886.08628	-15887.27604	-15890.36964	-1589
14.74	-15879.37355	-15880.06533	-15884.97616	-15886.17528	-15885.49463	-15886.69942	-15886.08628	-15887.27604	-15890.36964	-1589
14.83	-15879.37355	-15880.06533	-15884.97616	-15886.17528	-15885.49463	-15886.69942	-15886.08628	-15887.27604	-15890.36964	-1589
14.93	-15879.37355	-15880.06532	-15884.97615	-15886.17528	-15885.49462	-15886.69942	-15886.08628	-15887.27605	-15890.36964	-1589
15.02	-15879.37355	-15880.06531	-15884.97615	-15886.17528	-15885.49462	-15886.69942	-15886.08628	-15887.27605	-15890.36964	-1589
15.12	-15879.37355	-15880.06530	-15884.97614	-15886.17528	-15885.49462	-15886.69942	-15886.08628	-15887.27605	-15890.36963	-1589
15.21	-15879.37355	-15880.06530	-15884.97614	-15886.17528	-15885.49463	-15886.69941	-15886.08629	-15887.27605	-15890.36964	-1589
15.31	-15879.37355	-15880.06530	-15884.97614	-15886.17528	-15885.49463	-15886.69941	-15886.08629	-15887.27605	-15890.36964	-1589
15.40	-15879.37354	-15880.06530	-15884.97614	-15886.17528	-15885.49462	-15886.69941	-15886.08629	-15887.27606	-15890.36964	-1589
15.50	-15879.37354	-15880.06529	-15884.97613	-15886.17528	-15885.49462	-15886.69941	-15886.08629	-15887.27606	-15890.36964	-1589
15.59	-15879.37354	-15880.06529	-15884.97613	-15886.17528	-15885.49462	-15886.69941	-15886.08629	-15887.27606	-15890.36963	-1589
15.68	-15879.37354	-15880.06529	-15884.97613	-15886.17528	-15885.49462	-15886.69941	-15886.08629	-15887.27606	-15890.36963	-1589
15.78	-15879.37354	-15880.06529	-15884.97613	-15886.17528	-15885.49462	-15886.69941	-15886.08629	-15887.27606	-15890.36963	-1589
15.87	-15879.37354	-15880.06528	-15884.97612	-15886.17528	-15885.49462	-15886.69941	-15886.08630	-15887.27606	-15890.36963	-1589

Table S20: The absolute energies in hartree of the potential energy curve of TaSi in the $^4\Pi$ state with a CASSCF wave function and nom-CPO active space.

Distance	CASSCF	CASPT2	tpbe	ftpbe	trevpbe	ftrevpbe	tblyp	ftblyp	torelyp	fto
15.97	-15879.37354	-15880.06528	-15884.97612	-15886.17528	-15885.49462	-15886.69941	-15886.08630	-15887.27606	-15890.36963	-1589
16.06	-15879.37354	-15880.06528	-15884.97612	-15886.17528	-15885.49462	-15886.69941	-15886.08630	-15887.27606	-15890.36963	-1589
16.16	-15879.37354	-15880.06528	-15884.97612	-15886.17528	-15885.49462	-15886.69941	-15886.08630	-15887.27606	-15890.36963	-1589
16.25	-15879.37354	-15880.06527	-15884.97612	-15886.17528	-15885.49462	-15886.69941	-15886.08630	-15887.27606	-15890.36963	-1589
16.35	-15879.37354	-15880.06527	-15884.97612	-15886.17528	-15885.49461	-15886.69941	-15886.08630	-15887.27607	-15890.36963	-1589
16.44	-15879.37354	-15880.06527	-15884.97612	-15886.17528	-15885.49462	-15886.69941	-15886.08630	-15887.27607	-15890.36963	-1589
16.54	-15879.37354	-15880.06527	-15884.97612	-15886.17528	-15885.49461	-15886.69941	-15886.08630	-15887.27607	-15890.36963	-1589
16.63	-15879.37354	-15880.06527	-15884.97612	-15886.17528	-15885.49462	-15886.69941	-15886.08630	-15887.27607	-15890.36963	-1589
16.72	-15879.37354	-15880.06527	-15884.97611	-15886.17528	-15885.49461	-15886.69941	-15886.08630	-15887.27607	-15890.36963	-1589
16.82	-15879.37354	-15880.06527	-15884.97611	-15886.17528	-15885.49461	-15886.69941	-15886.08630	-15887.27607	-15890.36963	-1589
16.91	-15879.37354	-15880.06526	-15884.97611	-15886.17528	-15885.49461	-15886.69941	-15886.08630	-15887.27608	-15890.36962	-1589
17.01	-15879.37354	-15880.06526	-15884.97611	-15886.17528	-15885.49461	-15886.69941	-15886.08630	-15887.27608	-15890.36962	-1589
17.10	-15879.37354	-15880.06526	-15884.97611	-15886.17528	-15885.49461	-15886.69941	-15886.08630	-15887.27608	-15890.36962	-1589
17.20	-15879.37353	-15880.06526	-15884.97611	-15886.17528	-15885.49461	-15886.69941	-15886.08630	-15887.27608	-15890.36962	-1589
17.29	-15879.37353	-15880.06526	-15884.97611	-15886.17528	-15885.49461	-15886.69941	-15886.08630	-15887.27608	-15890.36962	-1589
17.39	-15879.37353	-15880.06526	-15884.97611	-15886.17528	-15885.49461	-15886.69941	-15886.08630	-15887.27608	-15890.36962	-1589
17.48	-15879.37353	-15880.06526	-15884.97611	-15886.17528	-15885.49461	-15886.69941	-15886.08630	-15887.27608	-15890.36962	-1589
17.57	-15879.37353	-15880.06526	-15884.97611	-15886.17528	-15885.49461	-15886.69941	-15886.08630	-15887.27608	-15890.36962	-1589
17.67	-15879.37353	-15880.06526	-15884.97611	-15886.17528	-15885.49461	-15886.69941	-15886.08630	-15887.27608	-15890.36962	-1589
17.76	-15879.37353	-15880.06526	-15884.97610	-15886.17528	-15885.49461	-15886.69941	-15886.08630	-15887.27608	-15890.36962	-1589
17.86	-15879.37353	-15880.06525	-15884.97611	-15886.17528	-15885.49461	-15886.69941	-15886.08630	-15887.27608	-15890.36962	-1589
17.95	-15879.37353	-15880.06525	-15884.97610	-15886.17528	-15885.49461	-15886.69941	-15886.08630	-15887.27608	-15890.36962	-1589
18.05	-15879.37353	-15880.06525	-15884.97611	-15886.17528	-15885.49461	-15886.69941	-15886.08630	-15887.27608	-15890.36962	-1589

Table S21: The absolute energies in hartree of the potential energy curve of TaSi in the $^6\Sigma^+$ state with a CASSCF wave function and nom-CPO active space.

Distance	CASSCF	CASPT2	tpbe	ftpbe	trevpbe	ftrevpbe	tblyp	ftblyp	torelyp	fto
1.98	-15875.58885	-15876.39081	-15881.29987	-15882.48683	-15881.79542	-15882.98701	-15882.36773	-15883.54544	-15886.71646	-1588
2.08	-15876.31199	-15877.11213	-15882.01661	-15883.20326	-15882.51350	-15883.70485	-15883.08709	-15884.26452	-15887.42747	-1588
2.17	-15876.88549	-15877.68327	-15882.58402	-15883.77041	-15883.08216	-15884.27334	-15883.65666	-15884.83386	-15887.98994	-1588
2.27	-15877.34050	-15878.13527	-15883.03275	-15884.21904	-15883.53216	-15884.72339	-15884.10711	-15885.28410	-15888.43451	-1588
2.36	-15877.70233	-15878.49420	-15883.38967	-15884.57586	-15883.89024	-15885.08150	-15884.46548	-15885.64225	-15888.78795	-1589
2.46	-15877.99126	-15878.78048	-15883.67482	-15884.86090	-15884.17647	-15885.36773	-15884.75189	-15885.92842	-15889.07021	-1589
2.55	-15878.22068	-15879.00138	-15883.89474	-15885.07777	-15884.39609	-15885.58451	-15884.97431	-15886.14765	-15889.28431	-1589
2.65	-15878.42518	-15879.20354	-15884.09678	-15885.27959	-15884.59903	-15885.78721	-15885.17736	-15886.35054	-15889.48466	-1589
2.74	-15878.59306	-15879.36996	-15884.26394	-15885.44659	-15884.76700	-15885.95501	-15885.34541	-15886.51847	-15889.65049	-1589
2.83	-15878.73176	-15879.45707	-15884.34238	-15885.53199	-15884.84646	-15886.04146	-15885.42512	-15886.60242	-15889.73290	-1589
2.93	-15878.84703	-15879.57597	-15884.46243	-15885.65195	-15884.96711	-15886.16227	-15885.54545	-15886.72268	-15889.85115	-1589
3.02	-15878.94427	-15879.67870	-15884.56696	-15885.75613	-15885.07216	-15886.26725	-15885.65021	-15886.82716	-15889.95385	-1589
3.12	-15879.02767	-15879.76668	-15884.66294	-15885.85307	-15885.16893	-15886.36557	-15885.74625	-15886.92504	-15890.05099	-1589
3.21	-15879.09199	-15879.84212	-15884.74126	-15885.93096	-15885.24768	-15886.44401	-15885.82474	-15887.00324	-15890.12778	-1589
3.31	-15879.14909	-15879.90618	-15884.80688	-15885.99609	-15885.31375	-15886.50959	-15885.89076	-15887.06889	-15890.19230	-1589
3.40	-15879.20166	-15879.96016	-15884.86200	-15886.05074	-15885.36932	-15886.56467	-15885.94639	-15887.12415	-15890.24655	-1589
3.50	-15879.25272	-15880.00538	-15884.90813	-15886.09643	-15885.41589	-15886.61075	-15885.99306	-15887.17046	-15890.29194	-1589
3.59	-15879.29297	-15880.04298	-15884.94653	-15886.13440	-15885.45471	-15886.64911	-15886.03204	-15887.20908	-15890.32971	-1589
3.68	-15879.32644	-15880.07402	-15884.97835	-15886.16577	-15885.48693	-15886.68082	-15886.06446	-15887.24114	-15890.36099	-1589
3.78	-15879.35404	-15880.09949	-15885.00456	-15886.19142	-15885.51351	-15886.70677	-15886.09139	-15887.26756	-15890.38674	-1589
3.87	-15879.37656	-15880.12113	-15885.02789	-15886.21315	-15885.53712	-15886.72856	-15886.11602	-15887.29065	-15890.40941	-1589
3.97	-15879.39470	-15880.13758	-15885.04534	-15886.23023	-15885.55489	-15886.74593	-15886.13408	-15887.30837	-15890.42646	-1589
4.06	-15879.40610	-15880.15038	-15885.05900	-15886.24364	-15885.56888	-15886.75963	-15886.14833	-15887.32238	-15890.43980	-1589

Table S21: The absolute energies in hartree of the potential energy curve of TaSi in the $^6\Sigma^+$ state with a CASSCF wave function and nom-CPO active space.

Distance	CASSCF	CASPT2	tpbe	ftpbe	trevpbe	ftrevpbe	tblyp	ftblyp	torelyp	fto
4.16	-15879.41777	-15880.16008	-15885.06950	-15886.25394	-15885.57968	-15886.77021	-15886.15938	-15887.33326	-15890.45005	-1589
4.25	-15879.42669	-15880.17201	-15885.08291	-15886.26708	-15885.59364	-15886.78386	-15886.17387	-15887.34750	-15890.46311	-1589
4.35	-15879.43328	-15880.17500	-15885.08655	-15886.27063	-15885.59754	-15886.78765	-15886.17803	-15887.35159	-15890.46665	-1589
4.44	-15879.43787	-15880.17642	-15885.08860	-15886.27261	-15885.59984	-15886.78986	-15886.18060	-15887.35410	-15890.46865	-1589
4.54	-15879.44079	-15880.17655	-15885.08934	-15886.27330	-15885.60081	-15886.79076	-15886.18185	-15887.35531	-15890.46936	-1589
4.63	-15879.44230	-15880.17560	-15885.08896	-15886.27289	-15885.60065	-15886.79054	-15886.18198	-15887.35541	-15890.46899	-1589
4.72	-15879.44263	-15880.17377	-15885.08766	-15886.27157	-15885.59956	-15886.78941	-15886.18119	-15887.35461	-15890.46773	-1589
4.82	-15879.44199	-15880.17124	-15885.08564	-15886.26954	-15885.59774	-15886.78756	-15886.17967	-15887.35308	-15890.46576	-1589
4.91	-15879.44055	-15880.16814	-15885.08303	-15886.26693	-15885.59532	-15886.78512	-15886.17755	-15887.35097	-15890.46323	-1589
5.01	-15879.43846	-15880.16459	-15885.07993	-15886.26385	-15885.59240	-15886.78220	-15886.17494	-15887.34838	-15890.46022	-1589
5.10	-15879.43585	-15880.16071	-15885.07645	-15886.26039	-15885.58910	-15886.77889	-15886.17194	-15887.34540	-15890.45686	-1589
5.20	-15879.43284	-15880.15657	-15885.07269	-15886.25666	-15885.58550	-15886.77531	-15886.16865	-15887.34214	-15890.45321	-1589
5.29	-15879.42952	-15880.15224	-15885.06871	-15886.25272	-15885.58169	-15886.77151	-15886.16514	-15887.33866	-15890.44937	-1589
5.39	-15879.42597	-15880.14778	-15885.06455	-15886.24862	-15885.57770	-15886.76755	-15886.16144	-15887.33500	-15890.44536	-1589
5.48	-15879.42226	-15880.14324	-15885.06026	-15886.24438	-15885.57356	-15886.76345	-15886.15759	-15887.33120	-15890.44123	-1589
5.57	-15879.41845	-15880.13865	-15885.05587	-15886.24005	-15885.56932	-15886.75926	-15886.15362	-15887.32729	-15890.43699	-1589
5.67	-15879.41459	-15880.13401	-15885.05135	-15886.23561	-15885.56496	-15886.75495	-15886.14950	-15887.32324	-15890.43263	-1589
5.76	-15879.41073	-15880.12920	-15885.04651	-15886.23089	-15885.56029	-15886.75037	-15886.14503	-15887.31887	-15890.42792	-1589
5.86	-15879.40691	-15880.12173	-15885.03701	-15886.22178	-15885.55107	-15886.74155	-15886.13554	-15887.30978	-15890.41806	-1589
5.95	-15879.40319	-15880.11527	-15885.02882	-15886.21385	-15885.54312	-15886.73384	-15886.12755	-15887.30207	-15890.40966	-1589
6.05	-15879.39980	-15880.10688	-15885.01935	-15886.20476	-15885.53385	-15886.72484	-15886.11859	-15887.29371	-15890.39929	-1589
6.14	-15879.39703	-15880.10296	-15885.01549	-15886.20095	-15885.53010	-15886.72109	-15886.11530	-15887.29048	-15890.39557	-1589
6.24	-15879.39478	-15880.09946	-15885.01192	-15886.19741	-15885.52663	-15886.71762	-15886.11230	-15887.28753	-15890.39222	-1589
6.33	-15879.39275	-15880.09626	-15885.00853	-15886.19406	-15885.52334	-15886.71434	-15886.10947	-15887.28478	-15890.38908	-1589
6.43	-15879.39093	-15880.09333	-15885.00532	-15886.19089	-15885.52022	-15886.71123	-15886.10683	-15887.28220	-15890.38613	-1589
6.52	-15879.38932	-15880.09065	-15885.00231	-15886.18793	-15885.51730	-15886.70834	-15886.10436	-15887.27980	-15890.38338	-1589
6.61	-15879.38790	-15880.08824	-15884.99954	-15886.18520	-15885.51462	-15886.70568	-15886.10210	-15887.27760	-15890.38087	-1589
6.71	-15879.38665	-15880.08608	-15884.99701	-15886.18270	-15885.51217	-15886.70326	-15886.10003	-15887.27559	-15890.37859	-1589
6.80	-15879.38557	-15880.08415	-15884.99473	-15886.18044	-15885.50997	-15886.70108	-15886.09818	-15887.27378	-15890.37655	-1589
6.90	-15879.38462	-15880.08243	-15884.99266	-15886.17839	-15885.50798	-15886.69911	-15886.09650	-15887.27214	-15890.37471	-1589
6.99	-15879.38379	-15880.08090	-15884.99079	-15886.17654	-15885.50620	-15886.69734	-15886.09500	-15887.27067	-15890.37308	-1589
7.09	-15879.38307	-15880.07952	-15884.98911	-15886.17487	-15885.50460	-15886.69575	-15886.09364	-15887.26934	-15890.37163	-1589
7.18	-15879.38244	-15880.07830	-15884.98760	-15886.17337	-15885.50317	-15886.69433	-15886.09243	-15887.26814	-15890.37034	-1589
7.28	-15879.38189	-15880.07720	-15884.98624	-15886.17201	-15885.50189	-15886.69305	-15886.09135	-15887.26707	-15890.36920	-1589
7.37	-15879.38140	-15880.07621	-15884.98502	-15886.17079	-15885.50074	-15886.69191	-15886.09038	-15887.26610	-15890.36820	-1589
7.46	-15879.38097	-15880.07532	-15884.98391	-15886.16968	-15885.49971	-15886.69088	-15886.08951	-15887.26523	-15890.36731	-1589
7.56	-15879.38059	-15880.07451	-15884.98291	-15886.16868	-15885.49879	-15886.68995	-15886.08873	-15887.26444	-15890.36652	-1589
7.65	-15879.38026	-15880.07379	-15884.98202	-15886.16777	-15885.49797	-15886.68912	-15886.08802	-15887.26373	-15890.36584	-1589
7.75	-15879.37996	-15880.07313	-15884.98121	-15886.16695	-15885.49723	-15886.68838	-15886.08739	-15887.26309	-15890.36523	-1589
7.84	-15879.37970	-15880.07253	-15884.98048	-15886.16621	-15885.49658	-15886.68771	-15886.08683	-15887.26252	-15890.36471	-1589
7.94	-15879.37946	-15880.07199	-15884.97982	-15886.16554	-15885.49599	-15886.68712	-15886.08633	-15887.26201	-15890.36425	-1589
8.03	-15879.37926	-15880.07149	-15884.97923	-15886.16494	-15885.49547	-15886.68658	-15886.08588	-15887.26155	-15890.36386	-1589
8.13	-15879.37907	-15880.07104	-15884.97870	-15886.16439	-15885.49501	-15886.68610	-15886.08548	-15887.26113	-15890.36352	-1589
8.22	-15879.37890	-15880.07063	-15884.97822	-15886.16390	-15885.49459	-15886.68568	-15886.08512	-15887.26075	-15890.36322	-1589
8.31	-15879.37876	-15880.07026	-15884.97778	-15886.16345	-15885.49422	-15886.68529	-15886.08480	-15887.26042	-15890.36297	-1589
8.41	-15879.37862	-15880.06992	-15884.97739	-15886.16305	-15885.49390	-15886.68496	-15886.08451	-15887.26012	-15890.36276	-1589
8.50	-15879.37851	-15880.06961	-15884.97705	-15886.16269	-15885.49361	-15886.68465	-15886.08426	-15887.25985	-15890.36258	-1589
8.60	-15879.37840	-15880.06932	-15884.97673	-15886.16237	-15885.49336	-15886.68439	-15886.08404	-15887.25961	-15890.36243	-1589
8.69	-15879.37831	-15880.06906	-15884.97645	-15886.16207	-15885.49314	-15886.68415	-15886.08384	-15887.25939	-15890.36230	-1589
8.79	-15879.37822	-15880.06882	-15884.97620	-15886.16181	-15885.49294	-15886.68394	-15886.08366	-15887.25920	-15890.36220	-1589
8.88	-15879.37814	-15880.06859	-15884.97597	-15886.16157	-15885.49277	-15886.68375	-15886.08351	-15887.25903	-15890.36212	-1589
8.98	-15879.37808	-15880.06839	-15884.97577	-15886.16135	-15885.49261	-15886.68358	-15886.08337	-15887.25888	-15890.36205	-1589

Table S21: The absolute energies in hartree of the potential energy curve of TaSi in the $^6\Sigma^+$ state with a CASSCF wave function and nom-CPO active space.

Distance	CASSCF	CASPT2	tpbe	ftpbe	trevpbe	ftrevpbe	tblyp	ftblyp	torelyp	fto
9.07	-15879.37801	-15880.06820	-15884.97558	-15886.16115	-15885.49248	-15886.68344	-15886.08325	-15887.25874	-15890.36199	-1589
9.17	-15879.37796	-15880.06803	-15884.97542	-15886.16098	-15885.49236	-15886.68331	-15886.08314	-15887.25862	-15890.36196	-1589
9.26	-15879.37791	-15880.06787	-15884.97527	-15886.16082	-15885.49226	-15886.68319	-15886.08305	-15887.25852	-15890.36193	-1589
9.35	-15879.37786	-15880.06773	-15884.97514	-15886.16068	-15885.49217	-15886.68309	-15886.08297	-15887.25842	-15890.36191	-1589
9.45	-15879.37782	-15880.06759	-15884.97502	-15886.16055	-15885.49210	-15886.68300	-15886.08290	-15887.25834	-15890.36189	-1589
9.54	-15879.37779	-15880.06747	-15884.97491	-15886.16043	-15885.49203	-15886.68292	-15886.08290	-15887.25827	-15890.36189	-1589
9.64	-15879.37776	-15880.06735	-15884.97482	-15886.16033	-15885.49197	-15886.68285	-15886.08290	-15887.25820	-15890.36188	-1589
9.73	-15879.37773	-15880.06724	-15884.97473	-15886.16023	-15885.49192	-15886.68279	-15886.08290	-15887.25814	-15890.36188	-1589
9.83	-15879.37770	-15880.06714	-15884.97465	-15886.16014	-15885.49187	-15886.68273	-15886.08290	-15887.25809	-15890.36189	-1589
9.92	-15879.37768	-15880.06705	-15884.97458	-15886.16007	-15885.49184	-15886.68269	-15886.08290	-15887.25805	-15890.36189	-1589
10.02	-15879.37765	-15880.06697	-15884.97452	-15886.16000	-15885.49180	-15886.68265	-15886.08290	-15887.25802	-15890.36190	-1589
10.11	-15879.37764	-15880.06689	-15884.97446	-15886.15993	-15885.49178	-15886.68261	-15886.08290	-15887.25798	-15890.36191	-1589
10.20	-15879.37762	-15880.06681	-15884.97441	-15886.15987	-15885.49175	-15886.68257	-15886.08290	-15887.25796	-15890.36192	-1589
10.30	-15879.37760	-15880.06674	-15884.97436	-15886.15982	-15885.49173	-15886.68254	-15886.08290	-15887.25793	-15890.36194	-1589
10.39	-15879.37759	-15880.06668	-15884.97432	-15886.15977	-15885.49171	-15886.68252	-15886.08290	-15887.25791	-15890.36195	-1589
10.49	-15879.37757	-15880.06662	-15884.97428	-15886.15973	-15885.49169	-15886.68249	-15886.08290	-15887.25790	-15890.36195	-1589
10.58	-15879.37756	-15880.06656	-15884.97425	-15886.15969	-15885.49168	-15886.68247	-15886.08290	-15887.25788	-15890.36196	-1589
10.68	-15879.37755	-15880.06651	-15884.97421	-15886.15965	-15885.49166	-15886.68245	-15886.08290	-15887.25787	-15890.36197	-1589
10.77	-15879.37754	-15880.06646	-15884.97419	-15886.15962	-15885.49165	-15886.68243	-15886.08290	-15887.25786	-15890.36198	-1589
10.87	-15879.37753	-15880.06641	-15884.97416	-15886.15959	-15885.49165	-15886.68242	-15886.08290	-15887.25786	-15890.36199	-1589
10.96	-15879.37753	-15880.06637	-15884.97414	-15886.15956	-15885.49164	-15886.68241	-15886.08290	-15887.25785	-15890.36200	-1589
11.05	-15879.37752	-15880.06633	-15884.97411	-15886.15954	-15885.49163	-15886.68240	-15886.08290	-15887.25785	-15890.36201	-1589
11.15	-15879.37751	-15880.06629	-15884.97409	-15886.15951	-15885.49162	-15886.68238	-15886.08290	-15887.25784	-15890.36202	-1589
11.24	-15879.37751	-15880.06626	-15884.97407	-15886.15949	-15885.49162	-15886.68237	-15886.08290	-15887.25784	-15890.36202	-1589
11.34	-15879.37750	-15880.06622	-15884.97405	-15886.15947	-15885.49161	-15886.68236	-15886.08290	-15887.25784	-15890.36203	-1589
11.43	-15879.37750	-15880.06619	-15884.97404	-15886.15945	-15885.49160	-15886.68235	-15886.08290	-15887.25784	-15890.36203	-1589
11.53	-15879.37749	-15880.06616	-15884.97403	-15886.15943	-15885.49160	-15886.68234	-15886.08290	-15887.25784	-15890.36203	-1589
11.62	-15879.37749	-15880.06613	-15884.97401	-15886.15941	-15885.49159	-15886.68233	-15886.08290	-15887.25784	-15890.36204	-1589
11.72	-15879.37749	-15880.06610	-15884.97400	-15886.15940	-15885.49159	-15886.68233	-15886.08290	-15887.25784	-15890.36204	-1589
11.81	-15879.37748	-15880.06608	-15884.97399	-15886.15939	-15885.49158	-15886.68232	-15886.08290	-15887.25784	-15890.36204	-1589
11.91	-15879.37748	-15880.06606	-15884.97398	-15886.15938	-15885.49158	-15886.68231	-15886.08290	-15887.25785	-15890.36204	-1589
12.00	-15879.37748	-15880.06603	-15884.97397	-15886.15937	-15885.49158	-15886.68231	-15886.08290	-15887.25785	-15890.36204	-1589
12.09	-15879.37748	-15880.06601	-15884.97396	-15886.15936	-15885.49157	-15886.68230	-15886.08290	-15887.25785	-15890.36204	-1589
12.19	-15879.37747	-15880.06599	-15884.97395	-15886.15935	-15885.49157	-15886.68230	-15886.08290	-15887.25786	-15890.36204	-1589
12.28	-15879.37747	-15880.06597	-15884.97394	-15886.15934	-15885.49157	-15886.68229	-15886.08290	-15887.25786	-15890.36204	-1589
12.38	-15879.37747	-15880.06595	-15884.97394	-15886.15933	-15885.49156	-15886.68229	-15886.08290	-15887.25786	-15890.36204	-1589
12.47	-15879.37747	-15880.06593	-15884.97393	-15886.15932	-15885.49156	-15886.68228	-15886.08290	-15887.25787	-15890.36204	-1589
12.57	-15879.37747	-15880.06592	-15884.97392	-15886.15931	-15885.49156	-15886.68228	-15886.08290	-15887.25787	-15890.36204	-1589
12.66	-15879.37747	-15880.06590	-15884.97392	-15886.15930	-15885.49155	-15886.68227	-15886.08290	-15887.25787	-15890.36204	-1589
12.76	-15879.37746	-15880.06589	-15884.97391	-15886.15930	-15885.49155	-15886.68227	-15886.08290	-15887.25788	-15890.36204	-1589
12.85	-15879.37746	-15880.06588	-15884.97391	-15886.15929	-15885.49155	-15886.68226	-15886.08290	-15887.25788	-15890.36204	-1589
12.94	-15879.37746	-15880.06580	-15884.97390	-15886.15929	-15885.49155	-15886.68226	-15886.08290	-15887.25788	-15890.36204	-1589
13.04	-15879.37746	-15880.06578	-15884.97390	-15886.15928	-15885.49155	-15886.68226	-15886.08290	-15887.25789	-15890.36203	-1589
13.13	-15879.37746	-15880.06578	-15884.97389	-15886.15928	-15885.49154	-15886.68226	-15886.08290	-15887.25789	-15890.36203	-1589
13.23	-15879.37746	-15880.06574	-15884.97389	-15886.15928	-15885.49154	-15886.68225	-15886.08290	-15887.25789	-15890.36203	-1589
13.32	-15879.37746	-15880.06573	-15884.97389	-15886.15927	-15885.49154	-15886.68225	-15886.08290	-15887.25790	-15890.36203	-1589
13.42	-15879.37746	-15880.06572	-15884.97388	-15886.15927	-15885.49154	-15886.68225	-15886.08290	-15887.25790	-15890.36203	-1589
13.51	-15879.37746	-15880.06571	-15884.97388	-15886.15927	-15885.49154	-15886.68224	-15886.08290	-15887.25790	-15890.36203	-1589
13.61	-15879.37746	-15880.06571	-15884.97388	-15886.15926	-15885.49154	-15886.68224	-15886.08290	-15887.25791	-15890.36203	-1589
13.70	-15879.37746	-15880.06570	-15884.97387	-15886.15926	-15885.49153	-15886.68224	-15886.08290	-15887.25791	-15890.36202	-1589
13.80	-15879.37746	-15880.06569	-15884.97387	-15886.15926	-15885.49153	-15886.68224	-15886.08290	-15887.25791	-15890.36202	-1589
13.89	-15879.37746	-15880.06569	-15884.97387	-15886.15925	-15885.49153	-15886.68223	-15886.08290	-15887.25791	-15890.36202	-1589

Table S21: The absolute energies in hartree of the potential energy curve of TaSi in the $^6\Sigma^+$ state with a CASSCF wave function and nom-CPO active space.

Distance	CASSCF	CASPT2	tpbe	ftpbe	trevpbe	ftrevpbe	tblyp	ftblyp	torelyp	fto
13.98	-15879.37746	-15880.06568	-15884.97387	-15886.15925	-15885.49153	-15886.68223	-15886.08290	-15887.25791	-15890.36202	-1589
14.08	-15879.37746	-15880.06568	-15884.97387	-15886.15925	-15885.49153	-15886.68223	-15886.08290	-15887.25791	-15890.36202	-1589
14.17	-15879.37746	-15880.06567	-15884.97387	-15886.15925	-15885.49153	-15886.68223	-15886.08290	-15887.25792	-15890.36202	-1589
14.27	-15879.37746	-15880.06557	-15884.97386	-15886.15925	-15885.49152	-15886.68223	-15886.08290	-15887.25792	-15890.36201	-1589
14.36	-15879.37746	-15880.06557	-15884.97386	-15886.15924	-15885.49152	-15886.68223	-15886.08290	-15887.25792	-15890.36201	-1589
14.46	-15879.37746	-15880.06556	-15884.97386	-15886.15924	-15885.49152	-15886.68223	-15886.08290	-15887.25792	-15890.36201	-1589
14.55	-15879.37746	-15880.06556	-15884.97386	-15886.15924	-15885.49152	-15886.68223	-15886.08290	-15887.25793	-15890.36201	-1589
14.65	-15879.37746	-15880.06556	-15884.97386	-15886.15924	-15885.49152	-15886.68222	-15886.08290	-15887.25793	-15890.36201	-1589
14.74	-15879.37746	-15880.06555	-15884.97386	-15886.15924	-15885.49152	-15886.68222	-15886.08290	-15887.25793	-15890.36201	-1589
14.83	-15879.37746	-15880.06555	-15884.97386	-15886.15924	-15885.49152	-15886.68222	-15886.08290	-15887.25793	-15890.36201	-1589
14.93	-15879.37746	-15880.06554	-15884.97386	-15886.15924	-15885.49152	-15886.68222	-15886.08290	-15887.25793	-15890.36201	-1589
15.02	-15879.37746	-15880.06554	-15884.97385	-15886.15923	-15885.49152	-15886.68222	-15886.08290	-15887.25793	-15890.36201	-1589
15.12	-15879.37746	-15880.06554	-15884.97385	-15886.15923	-15885.49152	-15886.68222	-15886.08290	-15887.25793	-15890.36201	-1589
15.21	-15879.37746	-15880.06554	-15884.97385	-15886.15923	-15885.49152	-15886.68222	-15886.08290	-15887.25793	-15890.36200	-1589
15.31	-15879.37746	-15880.06553	-15884.97385	-15886.15923	-15885.49152	-15886.68222	-15886.08290	-15887.25793	-15890.36200	-1589
15.40	-15879.37746	-15880.06553	-15884.97385	-15886.15923	-15885.49151	-15886.68221	-15886.08290	-15887.25793	-15890.36200	-1589
15.50	-15879.37746	-15880.06553	-15884.97385	-15886.15923	-15885.49151	-15886.68221	-15886.08290	-15887.25794	-15890.36200	-1589
15.59	-15879.37746	-15880.06553	-15884.97385	-15886.15923	-15885.49151	-15886.68221	-15886.08290	-15887.25794	-15890.36200	-1589
15.68	-15879.37746	-15880.06553	-15884.97385	-15886.15923	-15885.49151	-15886.68221	-15886.08290	-15887.25794	-15890.36200	-1589
15.78	-15879.37746	-15880.06552	-15884.97385	-15886.15923	-15885.49151	-15886.68221	-15886.08290	-15887.25794	-15890.36200	-1589
15.87	-15879.37746	-15880.06552	-15884.97385	-15886.15923	-15885.49151	-15886.68221	-15886.08290	-15887.25794	-15890.36200	-1589
15.97	-15879.37746	-15880.06552	-15884.97385	-15886.15923	-15885.49151	-15886.68221	-15886.08290	-15887.25794	-15890.36200	-1589
16.06	-15879.37746	-15880.06552	-15884.97385	-15886.15923	-15885.49151	-15886.68221	-15886.08290	-15887.25794	-15890.36200	-1589
16.16	-15879.37746	-15880.06552	-15884.97385	-15886.15923	-15885.49151	-15886.68221	-15886.08290	-15887.25794	-15890.36200	-1589
16.25	-15879.37746	-15880.06552	-15884.97385	-15886.15923	-15885.49151	-15886.68221	-15886.08290	-15887.25794	-15890.36200	-1589
16.35	-15879.37746	-15880.06552	-15884.97385	-15886.15923	-15885.49151	-15886.68221	-15886.08290	-15887.25794	-15890.36200	-1589
16.44	-15879.37746	-15880.06551	-15884.97385	-15886.15923	-15885.49151	-15886.68221	-15886.08290	-15887.25794	-15890.36200	-1589
16.54	-15879.37746	-15880.06552	-15884.97385	-15886.15923	-15885.49151	-15886.68221	-15886.08290	-15887.25794	-15890.36200	-1589
16.63	-15879.37746	-15880.06552	-15884.97385	-15886.15923	-15885.49151	-15886.68221	-15886.08290	-15887.25794	-15890.36200	-1589
16.72	-15879.37746	-15880.06551	-15884.97385	-15886.15923	-15885.49151	-15886.68221	-15886.08290	-15887.25794	-15890.36200	-1589
16.82	-15879.37746	-15880.06551	-15884.97385	-15886.15923	-15885.49151	-15886.68221	-15886.08290	-15887.25794	-15890.36200	-1589
16.91	-15879.37746	-15880.06551	-15884.97385	-15886.15922	-15885.49151	-15886.68221	-15886.08290	-15887.25794	-15890.36200	-1589
17.01	-15879.37746	-15880.06551	-15884.97385	-15886.15923	-15885.49151	-15886.68221	-15886.08290	-15887.25794	-15890.36200	-1589
17.10	-15879.37746	-15880.06551	-15884.97385	-15886.15922	-15885.49151	-15886.68221	-15886.08290	-15887.25794	-15890.36200	-1589
17.20	-15879.37746	-15880.06551	-15884.97385	-15886.15923	-15885.49151	-15886.68221	-15886.08290	-15887.25795	-15890.36200	-1589
17.29	-15879.37746	-15880.06551	-15884.97385	-15886.15922	-15885.49151	-15886.68221	-15886.08290	-15887.25795	-15890.36200	-1589
17.39	-15879.37746	-15880.06551	-15884.97385	-15886.15923	-15885.49151	-15886.68221	-15886.08290	-15887.25795	-15890.36200	-1589
17.48	-15879.37746	-15880.06551	-15884.97385	-15886.15923	-15885.49151	-15886.68221	-15886.08290	-15887.25795	-15890.36200	-1589
17.57	-15879.37746	-15880.06551	-15884.97385	-15886.15923	-15885.49151	-15886.68221	-15886.08290	-15887.25795	-15890.36200	-1589
17.67	-15879.37746	-15880.06551	-15884.97385	-15886.15923	-15885.49151	-15886.68221	-15886.08290	-15887.25795	-15890.36200	-1589
17.76	-15879.37746	-15880.06551	-15884.97385	-15886.15923	-15885.49151	-15886.68221	-15886.08290	-15887.25795	-15890.36200	-1589
17.86	-15879.37746	-15880.06551	-15884.97385	-15886.15922	-15885.49151	-15886.68221	-15886.08290	-15887.25795	-15890.36200	-1589
17.95	-15879.37746	-15880.06551	-15884.97385	-15886.15923	-15885.49151	-15886.68221	-15886.08290	-15887.25795	-15890.36200	-1589
18.05	-15879.37746	-15880.06551	-15884.97385	-15886.15922	-15885.49151	-15886.68221	-15886.08290	-15887.25795	-15890.36200	-1589

Table S22: The absolute energies in hartree of the potential energy curve of TaSi in the $^2\Delta$ state with a CASSCF wave function and nom-CPO active space.

Distance	CASSCF	CASPT2	tpbe	ftpbe	trevpbe	ftrevpbe	tblyp	ftblyp	torelyp	fto
1.98	-15875.70045	-15876.48114	-15881.39521	-15882.58738	-15881.89459	-15883.08795	-15882.46364	-15883.65405	-15886.82422	-1588
2.08	-15876.41927	-15877.20833	-15882.11764	-15883.30918	-15882.61834	-15883.81104	-15883.18894	-15884.37883	-15887.54129	-1588

Table S22: The absolute energies in hartree of the potential energy curve of TaSi in the $^2\Delta$ state with a CASSCF wave function and nom-CPO active space.

Distance	CASSCF	CASPT2	tpbe	ftpbe	trevpbe	ftrevpbe	tblyp	ftblyp	torelyp	fto
2.17	-15877.01649	-15877.76969	-15882.67732	-15883.86714	-15883.17799	-15884.36816	-15883.75504	-15884.94370	-15888.09647	-1588
2.27	-15877.45408	-15878.23660	-15883.14107	-15884.33038	-15883.64290	-15884.83253	-15884.22060	-15885.40881	-15888.55615	-1588
2.36	-15877.82720	-15878.52900	-15883.44354	-15884.63432	-15883.94502	-15885.13712	-15884.52337	-15885.71298	-15888.84821	-1589
2.46	-15878.12470	-15878.84588	-15883.75752	-15884.94778	-15884.25995	-15885.45147	-15884.83871	-15886.02776	-15889.15975	-1589
2.55	-15878.36300	-15879.10094	-15884.01077	-15885.20058	-15884.51408	-15885.70511	-15885.09313	-15886.28168	-15889.41100	-1589
2.65	-15878.57277	-15879.30742	-15884.21624	-15885.40568	-15884.72038	-15885.91098	-15885.29960	-15886.48774	-15889.61485	-1589
2.74	-15878.72934	-15879.47558	-15884.38400	-15885.57315	-15884.88891	-15886.07917	-15885.46822	-15886.65600	-15889.78130	-1589
2.83	-15878.86998	-15879.61328	-15884.52171	-15885.71066	-15885.02733	-15886.21735	-15885.60665	-15886.79415	-15889.91794	-1589
2.93	-15878.96902	-15879.72651	-15884.63526	-15885.82412	-15885.14154	-15886.33145	-15885.72079	-15886.90811	-15890.03062	-1589
3.02	-15879.06069	-15879.81965	-15884.72881	-15885.91768	-15885.23572	-15886.42562	-15885.81484	-15887.00207	-15890.12346	-1589
3.12	-15879.15296	-15879.89590	-15884.80523	-15885.99407	-15885.31276	-15886.50259	-15885.89175	-15887.07892	-15890.19931	-1589
3.21	-15879.21649	-15879.95812	-15884.86738	-15886.05608	-15885.37551	-15886.56515	-15885.95439	-15887.14144	-15890.26091	-1589
3.31	-15879.26880	-15880.00888	-15884.91800	-15886.10654	-15885.42670	-15886.61612	-15886.00546	-15887.19239	-15890.31094	-1589
3.40	-15879.31161	-15880.05017	-15884.95926	-15886.14765	-15885.46847	-15886.65771	-15886.04714	-15887.23395	-15890.35157	-1589
3.50	-15879.34632	-15880.08350	-15884.99267	-15886.18091	-15885.50234	-15886.69143	-15886.08095	-15887.26765	-15890.38434	-1589
3.59	-15879.37414	-15880.11007	-15885.01939	-15886.20752	-15885.52951	-15886.71846	-15886.10809	-15887.29469	-15890.41046	-1589
3.68	-15879.39606	-15880.13089	-15885.04047	-15886.22850	-15885.55099	-15886.73985	-15886.12960	-15887.31610	-15890.43097	-1589
3.78	-15879.41298	-15880.14680	-15885.05670	-15886.24464	-15885.56759	-15886.75637	-15886.14627	-15887.33268	-15890.44666	-1589
3.87	-15879.42565	-15880.15856	-15885.06882	-15886.25668	-15885.58005	-15886.76878	-15886.15884	-15887.34516	-15890.45829	-1589
3.97	-15879.43475	-15880.16682	-15885.07749	-15886.26529	-15885.58904	-15886.77773	-15886.16799	-15887.35422	-15890.46651	-1589
4.06	-15879.42484	-15880.17212	-15885.08322	-15886.27097	-15885.59506	-15886.78374	-15886.17421	-15887.36036	-15890.47181	-1589
4.16	-15879.43123	-15880.17483	-15885.08780	-15886.27256	-15885.60108	-15886.78826	-15886.17845	-15887.35888	-15890.47866	-1589
4.25	-15879.44596	-15880.17827	-15885.09165	-15886.27657	-15885.60528	-15886.79286	-15886.18334	-15887.36347	-15890.48163	-1589
4.35	-15879.44581	-15880.17765	-15885.09118	-15886.27620	-15885.60495	-15886.79279	-15886.18337	-15887.36337	-15890.48073	-1589
4.44	-15879.44432	-15880.17587	-15885.08951	-15886.27468	-15885.60341	-15886.79156	-15886.18220	-15887.36207	-15890.47864	-1589
4.54	-15879.44176	-15880.17318	-15885.08688	-15886.27221	-15885.60090	-15886.78941	-15886.18005	-15887.35980	-15890.47559	-1589
4.63	-15879.43836	-15880.16977	-15885.08346	-15886.26899	-15885.59759	-15886.78650	-15886.17709	-15887.35672	-15890.47174	-1589
4.72	-15879.43432	-15880.16580	-15885.07941	-15886.26515	-15885.59365	-15886.78298	-15886.17348	-15887.35299	-15890.46726	-1589
4.82	-15879.42981	-15880.16144	-15885.07491	-15886.26085	-15885.58924	-15886.77901	-15886.16937	-15887.34876	-15890.46232	-1589
4.91	-15879.42497	-15880.15679	-15885.07005	-15886.25619	-15885.58447	-15886.77467	-15886.16488	-15887.34414	-15890.45703	-1589
5.01	-15879.41993	-15880.15200	-15885.06500	-15886.25129	-15885.57950	-15886.77009	-15886.16017	-15887.33927	-15890.45155	-1589
5.10	-15879.41479	-15880.14720	-15885.05991	-15886.24632	-15885.57450	-15886.76542	-15886.15542	-15887.33432	-15890.44606	-1589
5.20	-15879.40962	-15880.14246	-15885.05493	-15886.24142	-15885.56959	-15886.76081	-15886.15075	-15887.32945	-15890.44070	-1589
5.29	-15879.40449	-15880.13781	-15885.05009	-15886.23665	-15885.56484	-15886.75630	-15886.14622	-15887.32472	-15890.43555	-1589
5.39	-15879.39946	-15880.13328	-15885.04542	-15886.23202	-15885.56026	-15886.75191	-15886.14186	-15887.32017	-15890.43063	-1589
5.48	-15879.39458	-15880.12888	-15885.04094	-15886.22755	-15885.55587	-15886.74767	-15886.13769	-15887.31581	-15890.42597	-1589
5.57	-15879.38989	-15880.12465	-15885.03667	-15886.22328	-15885.55170	-15886.74360	-15886.13376	-15887.31168	-15890.42159	-1589
5.67	-15879.38544	-15880.12058	-15885.03264	-15886.21922	-15885.54776	-15886.73971	-15886.13007	-15887.30780	-15890.41750	-1589
5.76	-15879.39808	-15880.11669	-15885.02884	-15886.21537	-15885.54406	-15886.73602	-15886.12663	-15887.30417	-15890.41371	-1589
5.86	-15879.39669	-15880.11298	-15885.02528	-15886.21174	-15885.54058	-15886.73253	-15886.12345	-15887.30079	-15890.41019	-1589
5.95	-15879.39540	-15880.10946	-15885.02193	-15886.20832	-15885.53733	-15886.72922	-15886.12050	-15887.29765	-15890.40693	-1589
6.05	-15879.39422	-15880.10613	-15885.01882	-15886.20512	-15885.53431	-15886.72612	-15886.11781	-15887.29478	-15890.40395	-1589
6.14	-15879.39316	-15880.10299	-15885.01591	-15886.20213	-15885.53150	-15886.72321	-15886.11535	-15887.29215	-15890.40123	-1589
6.24	-15879.39223	-15880.10003	-15885.01323	-15886.19936	-15885.52890	-15886.72050	-15886.11313	-15887.28977	-15890.39875	-1589
6.33	-15879.39144	-15880.09727	-15885.01075	-15886.19680	-15885.52650	-15886.71798	-15886.11114	-15887.28765	-15890.39651	-1589
6.43	-15879.39078	-15880.09469	-15885.00844	-15886.19441	-15885.52427	-15886.71562	-15886.10935	-15887.28574	-15890.39447	-1589
6.52	-15879.39024	-15880.09231	-15885.00631	-15886.19220	-15885.52222	-15886.71344	-15886.10775	-15887.28405	-15890.39263	-1589
6.61	-15879.38981	-15880.09010	-15885.00433	-15886.19017	-15885.52032	-15886.71141	-15886.10632	-15887.28255	-15890.39098	-1589
6.71	-15879.38948	-15880.08806	-15885.00250	-15886.18828	-15885.51857	-15886.70953	-15886.10505	-15887.28123	-15890.38948	-1589
6.80	-15879.38924	-15880.08612	-15885.00083	-15886.18655	-15885.51696	-15886.70780	-15886.10392	-15887.28007	-15890.38813	-1589
6.90	-15879.38907	-15880.08437	-15884.99931	-15886.18498	-15885.51551	-15886.70623	-15886.10295	-15887.27906	-15890.38691	-1589
6.99	-15879.38897	-15880.08283	-15884.99779	-15886.18344	-15885.51407	-15886.70468	-15886.10198	-15887.27810	-15890.38575	-1589

Table S22: The absolute energies in hartree of the potential energy curve of TaSi in the $^2\Delta$ state with a CASSCF wave function and nom-CPO active space.

Distance	CASSCF	CASPT2	tpbe	ftpbe	trevpbe	ftrevpbe	tblyp	ftblyp	torelyp	fto
7.09	-15879.38891	-15880.08143	-15884.99636	-15886.18198	-15885.51271	-15886.70323	-15886.10106	-15887.27722	-15890.38468	-1589
7.18	-15879.38890	-15880.08015	-15884.99501	-15886.18061	-15885.51143	-15886.70187	-15886.10020	-15887.27640	-15890.38370	-1589
7.28	-15879.38892	-15880.07900	-15884.99374	-15886.17932	-15885.51024	-15886.70060	-15886.09940	-15887.27564	-15890.38279	-1589
7.37	-15879.38897	-15880.07795	-15884.99255	-15886.17812	-15885.50913	-15886.69942	-15886.09864	-15887.27492	-15890.38195	-1589
7.46	-15879.38903	-15880.07699	-15884.99144	-15886.17699	-15885.50810	-15886.69833	-15886.09792	-15887.27426	-15890.38118	-1589
7.56	-15879.38911	-15880.07611	-15884.99041	-15886.17595	-15885.50714	-15886.69732	-15886.09726	-15887.27364	-15890.38047	-1589
7.65	-15879.38920	-15880.07530	-15884.98945	-15886.17498	-15885.50626	-15886.69639	-15886.09663	-15887.27306	-15890.37984	-1589
7.75	-15879.38930	-15880.07456	-15884.98858	-15886.17410	-15885.50547	-15886.69555	-15886.09606	-15887.27253	-15890.37927	-1589
7.84	-15879.38940	-15880.07388	-15884.98778	-15886.17328	-15885.50474	-15886.69478	-15886.09554	-15887.27204	-15890.37876	-1589
7.94	-15879.38951	-15880.07325	-15884.98705	-15886.17254	-15885.50408	-15886.69408	-15886.09506	-15887.27159	-15890.37831	-1589
8.03	-15879.38961	-15880.07266	-15884.98637	-15886.17186	-15885.50349	-15886.69345	-15886.09461	-15887.27118	-15890.37791	-1589
8.13	-15879.38972	-15880.07211	-15884.98577	-15886.17125	-15885.50296	-15886.69289	-15886.09422	-15887.27081	-15890.37757	-1589
8.22	-15879.38982	-15880.07158	-15884.98522	-15886.17069	-15885.50248	-15886.69238	-15886.09385	-15887.27047	-15890.37726	-1589
8.31	-15879.38991	-15880.07108	-15884.98472	-15886.17018	-15885.50204	-15886.69192	-15886.09352	-15887.27015	-15890.37700	-1589
8.41	-15879.39001	-15880.07062	-15884.98427	-15886.16973	-15885.50167	-15886.69152	-15886.09323	-15887.26988	-15890.37678	-1589
8.50	-15879.39010	-15880.07018	-15884.98387	-15886.16931	-15885.50133	-15886.69116	-15886.09297	-15887.26964	-15890.37659	-1589
8.60	-15879.39018	-15880.06978	-15884.98350	-15886.16894	-15885.50103	-15886.69083	-15886.09273	-15887.26941	-15890.37643	-1589
8.69	-15879.39027	-15880.06942	-15884.98319	-15886.16861	-15885.50077	-15886.69055	-15886.09253	-15887.26922	-15890.37630	-1589
8.79	-15879.39034	-15880.06908	-15884.98289	-15886.16831	-15885.50054	-15886.69030	-15886.09235	-15887.26904	-15890.37619	-1589
8.88	-15879.39041	-15880.06878	-15884.98263	-15886.16804	-15885.50034	-15886.69008	-15886.09219	-15887.26888	-15890.37611	-1589
8.98	-15879.39048	-15880.06850	-15884.98240	-15886.16780	-15885.50016	-15886.68988	-15886.09204	-15887.26875	-15890.37603	-1589
9.07	-15879.39054	-15880.06825	-15884.98219	-15886.16758	-15885.50000	-15886.68971	-15886.09192	-15887.26862	-15890.37598	-1589
9.17	-15879.39060	-15880.06802	-15884.98201	-15886.16739	-15885.49987	-15886.68956	-15886.09181	-15887.26852	-15890.37594	-1589
9.26	-15879.39066	-15880.06781	-15884.98184	-15886.16722	-15885.49975	-15886.68943	-15886.09171	-15887.26843	-15890.37592	-1589
9.35	-15879.39071	-15880.06763	-15884.98169	-15886.16706	-15885.49965	-15886.68931	-15886.09163	-15887.26834	-15890.37590	-1589
9.45	-15879.39076	-15880.06746	-15884.98156	-15886.16692	-15885.49956	-15886.68921	-15886.09156	-15887.26827	-15890.37588	-1589
9.54	-15879.39080	-15880.06730	-15884.98144	-15886.16680	-15885.49948	-15886.68912	-15886.09150	-15887.26821	-15890.37588	-1589
9.64	-15879.39084	-15880.06716	-15884.98133	-15886.16668	-15885.49941	-15886.68904	-15886.09144	-15887.26815	-15890.37588	-1589
9.73	-15879.39088	-15880.06704	-15884.98124	-15886.16658	-15885.49936	-15886.68897	-15886.09140	-15887.26811	-15890.37589	-1589
9.83	-15879.39091	-15880.06692	-15884.98116	-15886.16650	-15885.49932	-15886.68892	-15886.09137	-15887.26808	-15890.37590	-1589
9.92	-15879.39094	-15880.06682	-15884.98108	-15886.16642	-15885.49928	-15886.68887	-15886.09134	-15887.26805	-15890.37591	-1589
10.02	-15879.39097	-15880.06672	-15884.98102	-15886.16635	-15885.49925	-15886.68883	-15886.09132	-15887.26802	-15890.37593	-1589
10.11	-15879.39100	-15880.06663	-15884.98096	-15886.16629	-15885.49922	-15886.68879	-15886.09130	-15887.26800	-15890.37595	-1589
10.20	-15879.39102	-15880.06655	-15884.98091	-15886.16623	-15885.49919	-15886.68876	-15886.09128	-15887.26798	-15890.37596	-1589
10.30	-15879.39104	-15880.06648	-15884.98086	-15886.16618	-15885.49917	-15886.68873	-15886.09127	-15887.26797	-15890.37598	-1589
10.39	-15879.39106	-15880.06641	-15884.98082	-15886.16613	-15885.49916	-15886.68870	-15886.09126	-15887.26796	-15890.37600	-1589
10.49	-15879.39108	-15880.06634	-15884.98078	-15886.16609	-15885.49914	-15886.68868	-15886.09126	-15887.26795	-15890.37602	-1589
10.58	-15879.39110	-15880.06629	-15884.98075	-15886.16605	-15885.49913	-15886.68866	-15886.09125	-15887.26795	-15890.37603	-1589
10.68	-15879.39111	-15880.06623	-15884.98072	-15886.16601	-15885.49912	-15886.68865	-15886.09125	-15887.26794	-15890.37605	-1589
10.77	-15879.39113	-15880.06618	-15884.98069	-15886.16599	-15885.49911	-15886.68864	-15886.09126	-15887.26794	-15890.37607	-1589
10.87	-15879.39114	-15880.06614	-15884.98067	-15886.16596	-15885.49911	-15886.68863	-15886.09126	-15887.26795	-15890.37609	-1589
10.96	-15879.39115	-15880.06609	-15884.98064	-15886.16593	-15885.49910	-15886.68861	-15886.09126	-15887.26794	-15890.37610	-1589
11.05	-15879.39116	-15880.06606	-15884.98063	-15886.16592	-15885.49910	-15886.68861	-15886.09127	-15887.26795	-15890.37612	-1589
11.15	-15879.39117	-15880.06602	-15884.98061	-15886.16589	-15885.49910	-15886.68860	-15886.09127	-15887.26795	-15890.37613	-1589
11.24	-15879.39118	-15880.06598	-15884.98059	-15886.16588	-15885.49909	-15886.68859	-15886.09128	-15887.26796	-15890.37614	-1589
11.34	-15879.39119	-15880.06595	-15884.98058	-15886.16586	-15885.49909	-15886.68859	-15886.09129	-15887.26796	-15890.37615	-1589
11.43	-15879.39119	-15880.06592	-15884.98056	-15886.16584	-15885.49909	-15886.68858	-15886.09129	-15887.26797	-15890.37616	-1589
11.53	-15879.39120	-15880.06590	-15884.98055	-15886.16583	-15885.49909	-15886.68858	-15886.09130	-15887.26798	-15890.37617	-1589
11.62	-15879.39121	-15880.06587	-15884.98054	-15886.16581	-15885.49909	-15886.68857	-15886.09131	-15887.26798	-15890.37618	-1589
11.72	-15879.39121	-15880.06585	-15884.98053	-15886.16580	-15885.49909	-15886.68857	-15886.09132	-15887.26799	-15890.37618	-1589
11.81	-15879.39122	-15880.06583	-15884.98052	-15886.16579	-15885.49909	-15886.68857	-15886.09133	-15887.26800	-15890.37619	-1589
11.91	-15879.39122	-15880.06581	-15884.98052	-15886.16579	-15885.49909	-15886.68856	-15886.09134	-15887.26801	-15890.37620	-1589

Table S22: The absolute energies in hartree of the potential energy curve of TaSi in the $^2\Delta$ state with a CASSCF wave function and nom-CPO active space.

Distance	CASSCF	CASPT2	tpbe	ftpbe	trevpbe	ftrevpbe	tblyp	ftblyp	torelyp	fto
12.00	-15879.39122	-15880.06579	-15884.98051	-15886.16578	-15885.49909	-15886.68856	-15886.09134	-15887.26801	-15890.37620	-1589
12.09	-15879.39123	-15880.06577	-15884.98051	-15886.16577	-15885.49909	-15886.68856	-15886.09135	-15887.26802	-15890.37621	-1589
12.19	-15879.39123	-15880.06575	-15884.98050	-15886.16577	-15885.49909	-15886.68856	-15886.09136	-15887.26803	-15890.37622	-1589
12.28	-15879.39123	-15880.06574	-15884.98049	-15886.16576	-15885.49909	-15886.68856	-15886.09137	-15887.26804	-15890.37622	-1589
12.38	-15879.39124	-15880.06572	-15884.98049	-15886.16575	-15885.49909	-15886.68856	-15886.09138	-15887.26805	-15890.37622	-1589
12.47	-15879.39124	-15880.06571	-15884.98048	-15886.16575	-15885.49909	-15886.68855	-15886.09139	-15887.26805	-15890.37622	-1589
12.57	-15879.39124	-15880.06570	-15884.98048	-15886.16574	-15885.49909	-15886.68855	-15886.09140	-15887.26806	-15890.37623	-1589
12.66	-15879.39124	-15880.06569	-15884.98047	-15886.16574	-15885.49908	-15886.68855	-15886.09140	-15887.26806	-15890.37623	-1589
12.76	-15879.39124	-15880.06567	-15884.98047	-15886.16573	-15885.49908	-15886.68855	-15886.09141	-15887.26807	-15890.37623	-1589
12.85	-15879.39124	-15880.06566	-15884.98047	-15886.16573	-15885.49908	-15886.68854	-15886.09142	-15887.26808	-15890.37623	-1589
12.94	-15879.39125	-15880.06540	-15884.98046	-15886.16572	-15885.49908	-15886.68854	-15886.09142	-15887.26808	-15890.37623	-1589
13.04	-15879.39125	-15880.06539	-15884.98046	-15886.16572	-15885.49908	-15886.68854	-15886.09143	-15887.26809	-15890.37623	-1589
13.13	-15879.39125	-15880.06538	-15884.98046	-15886.16572	-15885.49908	-15886.68854	-15886.09144	-15887.26810	-15890.37623	-1589
13.23	-15879.39125	-15880.06538	-15884.98046	-15886.16572	-15885.49908	-15886.68854	-15886.09144	-15887.26810	-15890.37623	-1589
13.32	-15879.39125	-15880.06537	-15884.98046	-15886.16572	-15885.49908	-15886.68854	-15886.09145	-15887.26811	-15890.37623	-1589
13.42	-15879.39125	-15880.06536	-15884.98046	-15886.16571	-15885.49908	-15886.68854	-15886.09145	-15887.26812	-15890.37624	-1589
13.51	-15879.39125	-15880.06535	-15884.98045	-15886.16571	-15885.49908	-15886.68854	-15886.09146	-15887.26812	-15890.37624	-1589
13.61	-15879.39125	-15880.06535	-15884.98045	-15886.16571	-15885.49908	-15886.68854	-15886.09146	-15887.26812	-15890.37624	-1589
13.70	-15879.39125	-15880.06534	-15884.98045	-15886.16571	-15885.49908	-15886.68854	-15886.09147	-15887.26813	-15890.37624	-1589
13.80	-15879.39125	-15880.06533	-15884.98045	-15886.16570	-15885.49908	-15886.68854	-15886.09147	-15887.26813	-15890.37623	-1589
13.89	-15879.39125	-15880.06533	-15884.98045	-15886.16570	-15885.49908	-15886.68853	-15886.09148	-15887.26814	-15890.37623	-1589
13.98	-15879.39125	-15880.06532	-15884.98045	-15886.16570	-15885.49908	-15886.68853	-15886.09148	-15887.26814	-15890.37623	-1589
14.08	-15879.39125	-15880.06531	-15884.98044	-15886.16570	-15885.49908	-15886.68853	-15886.09148	-15887.26814	-15890.37623	-1589
14.17	-15879.39125	-15880.06531	-15884.98044	-15886.16570	-15885.49908	-15886.68853	-15886.09149	-15887.26815	-15890.37623	-1589
14.27	-15879.39125	-15880.06530	-15884.98044	-15886.16570	-15885.49908	-15886.68853	-15886.09149	-15887.26815	-15890.37623	-1589
14.36	-15879.39125	-15880.06530	-15884.98044	-15886.16570	-15885.49908	-15886.68853	-15886.09149	-15887.26815	-15890.37623	-1589
14.46	-15879.39125	-15880.06529	-15884.98044	-15886.16570	-15885.49908	-15886.68853	-15886.09150	-15887.26816	-15890.37623	-1589
14.55	-15879.39125	-15880.06529	-15884.98044	-15886.16570	-15885.49908	-15886.68853	-15886.09150	-15887.26816	-15890.37623	-1589
14.65	-15879.39125	-15880.06528	-15884.98044	-15886.16570	-15885.49908	-15886.68853	-15886.09151	-15887.26816	-15890.37623	-1589
14.74	-15879.39125	-15880.06527	-15884.98044	-15886.16570	-15885.49908	-15886.68853	-15886.09151	-15887.26817	-15890.37623	-1589
14.83	-15879.39125	-15880.06527	-15884.98044	-15886.16570	-15885.49908	-15886.68853	-15886.09151	-15887.26817	-15890.37623	-1589
14.93	-15879.39125	-15880.06526	-15884.98044	-15886.16570	-15885.49908	-15886.68853	-15886.09151	-15887.26817	-15890.37623	-1589
15.02	-15879.39125	-15880.06526	-15884.98044	-15886.16570	-15885.49908	-15886.68853	-15886.09151	-15887.26817	-15890.37623	-1589
15.12	-15879.39125	-15880.06526	-15884.98044	-15886.16569	-15885.49908	-15886.68853	-15886.09152	-15887.26817	-15890.37623	-1589
15.21	-15879.39125	-15880.06525	-15884.98044	-15886.16569	-15885.49908	-15886.68853	-15886.09152	-15887.26818	-15890.37623	-1589
15.31	-15879.39125	-15880.06525	-15884.98044	-15886.16569	-15885.49908	-15886.68853	-15886.09152	-15887.26818	-15890.37623	-1589
15.40	-15879.39125	-15880.06524	-15884.98044	-15886.16569	-15885.49908	-15886.68853	-15886.09152	-15887.26818	-15890.37623	-1589
15.50	-15879.39125	-15880.06524	-15884.98044	-15886.16569	-15885.49908	-15886.68853	-15886.09152	-15887.26818	-15890.37623	-1589
15.59	-15879.39125	-15880.06524	-15884.98044	-15886.16569	-15885.49908	-15886.68853	-15886.09152	-15887.26818	-15890.37623	-1589
15.68	-15879.39125	-15880.06523	-15884.98044	-15886.16569	-15885.49908	-15886.68853	-15886.09153	-15887.26818	-15890.37623	-1589
15.78	-15879.39125	-15880.06523	-15884.98044	-15886.16569	-15885.49908	-15886.68853	-15886.09153	-15887.26819	-15890.37623	-1589
15.87	-15879.39125	-15880.06523	-15884.98044	-15886.16569	-15885.49908	-15886.68853	-15886.09153	-15887.26819	-15890.37623	-1589
15.97	-15879.39125	-15880.06522	-15884.98044	-15886.16569	-15885.49908	-15886.68853	-15886.09153	-15887.26819	-15890.37623	-1589
16.06	-15879.39125	-15880.06522	-15884.98044	-15886.16569	-15885.49908	-15886.68853	-15886.09153	-15887.26819	-15890.37623	-1589
16.16	-15879.39125	-15880.06522	-15884.98044	-15886.16569	-15885.49908	-15886.68853	-15886.09153	-15887.26819	-15890.37623	-1589
16.25	-15879.39125	-15880.06521	-15884.98044	-15886.16569	-15885.49908	-15886.68853	-15886.09154	-15887.26819	-15890.37623	-1589
16.35	-15879.39125	-15880.06519	-15884.98044	-15886.16569	-15885.49908	-15886.68853	-15886.09154	-15887.26819	-15890.37623	-1589
16.44	-15879.39125	-15880.06519	-15884.98044	-15886.16569	-15885.49908	-15886.68853	-15886.09154	-15887.26819	-15890.37623	-1589
16.54	-15879.39125	-15880.06518	-15884.98044	-15886.16569	-15885.49908	-15886.68853	-15886.09154	-15887.26820	-15890.37623	-1589
16.63	-15879.39125	-15880.06518	-15884.98044	-15886.16569	-15885.49908	-15886.68853	-15886.09154	-15887.26820	-15890.37623	-1589
16.72	-15879.39125	-15880.06518	-15884.98044	-15886.16569	-15885.49908	-15886.68853	-15886.09154	-15887.26820	-15890.37623	-1589
16.82	-15879.39125	-15880.06517	-15884.98044	-15886.16569	-15885.49908	-15886.68853	-15886.09154	-15887.26820	-15890.37623	-1589

Table S22: The absolute energies in hartree of the potential energy curve of TaSi in the $^2\Delta$ state with a CASSCF wave function and nom-CPO active space.

Distance	CASSCF	CASPT2	tpbe	ftpbe	trevpbe	ftrevpbe	tblyp	ftblyp	torelyp	fto
16.91	-15879.39125	-15880.06517	-15884.98044	-15886.16569	-15885.49908	-15886.68853	-15886.09154	-15887.26820	-15890.37623	-1589
17.01	-15879.39125	-15880.06517	-15884.98044	-15886.16569	-15885.49908	-15886.68853	-15886.09154	-15887.26820	-15890.37623	-1589
17.10	-15879.39125	-15880.06517	-15884.98044	-15886.16569	-15885.49908	-15886.68853	-15886.09154	-15887.26820	-15890.37623	-1589
17.20	-15879.39125	-15880.06516	-15884.98044	-15886.16569	-15885.49908	-15886.68853	-15886.09154	-15887.26820	-15890.37623	-1589
17.29	-15879.39125	-15880.06516	-15884.98044	-15886.16569	-15885.49908	-15886.68853	-15886.09154	-15887.26820	-15890.37623	-1589
17.39	-15879.39125	-15880.06516	-15884.98044	-15886.16569	-15885.49908	-15886.68853	-15886.09154	-15887.26820	-15890.37623	-1589
17.48	-15879.39125	-15880.06516	-15884.98044	-15886.16569	-15885.49908	-15886.68853	-15886.09154	-15887.26820	-15890.37623	-1589
17.57	-15879.39125	-15880.06515	-15884.98044	-15886.16569	-15885.49908	-15886.68853	-15886.09154	-15887.26820	-15890.37623	-1589
17.67	-15879.39125	-15880.06515	-15884.98044	-15886.16569	-15885.49908	-15886.68853	-15886.09155	-15887.26820	-15890.37623	-1589
17.76	-15879.39125	-15880.06515	-15884.98044	-15886.16569	-15885.49908	-15886.68853	-15886.09155	-15887.26820	-15890.37623	-1589
17.86	-15879.39125	-15880.06514	-15884.98044	-15886.16570	-15885.49908	-15886.68853	-15886.09155	-15887.26821	-15890.37623	-1589
17.95	-15879.39125	-15880.06513	-15884.98044	-15886.16569	-15885.49908	-15886.68853	-15886.09155	-15887.26820	-15890.37623	-1589
18.05	-15879.39125	-15880.06513	-15884.98044	-15886.16570	-15885.49908	-15886.68853	-15886.09155	-15887.26821	-15890.37623	-1589

Table S23: The absolute energies in hartree of the potential energy curve of TaSi in the $^4\Pi$ state with a SP-based wave function and nom-CPO active space.

Distance	SP	tpbe	ftpbe	trevpbe	ftrevpbe	tblyp	ftblyp	torelyp	ftorelyp
1.98	-15875.60681	-15881.34485	-15882.53475	-15881.84361	-15883.03563	-15882.41154	-15883.59737	-15886.77544	-15888.06986
2.08	-15876.32142	-15882.05372	-15883.24327	-15882.55393	-15883.74553	-15883.12359	-15884.30908	-15887.47968	-15888.77379
2.17	-15876.89473	-15882.61542	-15883.80324	-15883.11682	-15884.30579	-15883.69020	-15884.87469	-15888.03906	-15889.33029
2.27	-15877.35380	-15883.06522	-15884.25151	-15883.56786	-15884.75489	-15884.14221	-15885.32559	-15888.48552	-15889.77479
2.36	-15877.71977	-15883.42577	-15884.61174	-15883.92952	-15885.11626	-15884.50415	-15885.68717	-15888.84282	-15890.13172
2.46	-15878.01249	-15883.71438	-15884.90017	-15884.21916	-15885.40578	-15884.79379	-15885.97645	-15889.12871	-15890.41737
2.55	-15878.24810	-15883.94639	-15885.13205	-15884.45206	-15885.63865	-15885.02669	-15886.20896	-15889.35836	-15890.64689
2.65	-15878.44819	-15884.13851	-15885.32544	-15884.64395	-15885.83247	-15885.22162	-15886.40574	-15889.54287	-15890.83429
2.74	-15878.61751	-15884.30677	-15885.49361	-15884.81295	-15886.00131	-15885.39103	-15886.57501	-15889.71009	-15891.00116
2.83	-15878.75764	-15884.44590	-15885.63263	-15884.95279	-15886.14094	-15885.53116	-15886.71504	-15889.84849	-15891.13916
2.93	-15878.87449	-15884.56129	-15885.74789	-15885.06888	-15886.25677	-15885.64744	-15886.83121	-15889.96341	-15891.25361
3.02	-15878.97271	-15884.65738	-15885.84381	-15885.16566	-15886.35325	-15885.74431	-15886.92796	-15890.05917	-15891.34891
3.12	-15879.06119	-15884.74119	-15885.92798	-15885.25054	-15886.44046	-15885.82588	-15887.00743	-15890.14105	-15891.43462
3.21	-15879.13610	-15884.81457	-15886.00099	-15885.32438	-15886.51416	-15885.89937	-15887.08051	-15890.21309	-15891.50662
3.31	-15879.19903	-15884.87583	-15886.06198	-15885.38608	-15886.57566	-15885.96094	-15887.14182	-15890.27328	-15891.56667
3.40	-15879.25155	-15884.92664	-15886.11257	-15885.43730	-15886.62671	-15886.01215	-15887.19279	-15890.32321	-15891.61646
3.50	-15879.29509	-15884.96851	-15886.15427	-15885.47956	-15886.66882	-15886.05445	-15887.23489	-15890.36432	-15891.65745
3.59	-15879.33093	-15885.00266	-15886.18826	-15885.51409	-15886.70320	-15886.08905	-15887.26931	-15890.39779	-15891.69082
3.68	-15879.36015	-15885.03024	-15886.21569	-15885.54202	-15886.73101	-15886.11709	-15887.29717	-15890.42478	-15891.71771
3.78	-15879.38370	-15885.05232	-15886.23764	-15885.56443	-15886.75331	-15886.13964	-15887.31954	-15890.44634	-15891.73919
3.87	-15879.40241	-15885.06969	-15886.25490	-15885.58212	-15886.77089	-15886.15749	-15887.33723	-15890.46325	-15891.75602
3.97	-15879.41699	-15885.08301	-15886.26812	-15885.59574	-15886.78442	-15886.17130	-15887.35087	-15890.47617	-15891.76887
4.06	-15879.42807	-15885.09294	-15886.27796	-15885.60594	-15886.79455	-15886.18171	-15887.36113	-15890.48575	-15891.77838
4.16	-15879.43620	-15885.10006	-15886.28500	-15885.61333	-15886.80187	-15886.18933	-15887.36858	-15890.49256	-15891.78515
4.35	-15879.44185	-15885.10481	-15886.28968	-15885.61832	-15886.80680	-15886.19459	-15887.37367	-15890.49706	-15891.78960
4.44	-15879.44542	-15885.10752	-15886.29232	-15885.62126	-15886.80969	-15886.19780	-15887.37672	-15890.49955	-15891.79205
4.54	-15879.44727	-15885.10853	-15886.29327	-15885.62248	-15886.81087	-15886.19932	-15887.37806	-15890.50038	-15891.79284
4.63	-15879.44770	-15885.10817	-15886.29286	-15885.62232	-15886.81068	-15886.19946	-15887.37803	-15890.49987	-15891.79230
4.72	-15879.44697	-15885.10673	-15886.29137	-15885.62107	-15886.80941	-15886.19852	-15887.37690	-15890.49828	-15891.79070
4.82	-15879.44532	-15885.10438	-15886.28898	-15885.61889	-15886.80723	-15886.19666	-15887.37485	-15890.49579	-15891.78821
4.91	-15879.44292	-15885.10127	-15886.28583	-15885.61594	-15886.80430	-15886.19403	-15887.37201	-15890.49254	-15891.78498
5.01	-15879.43997	-15885.09757	-15886.28211	-15885.61239	-15886.80078	-15886.19079	-15887.36857	-15890.48870	-15891.78118
5.10	-15879.43659	-15885.09342	-15886.27795	-15885.60838	-15886.79684	-15886.18708	-15887.36465	-15890.48440	-15891.77694

Table S23: The absolute energies in hartree of the potential energy curve of TaSi in the $^4\Pi$ state with a SP-based wave function and nom-CPO active space.

Distance	SP	tpbe	ftpbe	trevpbe	ftrevpbe	tblyp	ftblyp	torelyp	ftorelyp
5.20	-15879.43292	-15885.08895	-15886.27348	-15885.60404	-15886.79258	-15886.18302	-15887.36037	-15890.47977	-15891.77240
5.29	-15879.42906	-15885.08420	-15886.26874	-15885.59942	-15886.78808	-15886.17866	-15887.35579	-15890.47484	-15891.76758
5.39	-15879.42512	-15885.07921	-15886.26378	-15885.59455	-15886.78335	-15886.17402	-15887.35093	-15890.46964	-15891.76253
5.48	-15879.42116	-15885.07402	-15886.25864	-15885.58948	-15886.77846	-15886.16913	-15887.34584	-15890.46421	-15891.75727
5.57	-15879.41726	-15885.06866	-15886.25337	-15885.58426	-15886.77344	-15886.16404	-15887.34056	-15890.45861	-15891.75187
5.67	-15879.41347	-15885.06318	-15886.24799	-15885.57890	-15886.76833	-15886.15877	-15887.33514	-15890.45286	-15891.74636
5.76	-15879.40983	-15885.05759	-15886.24254	-15885.57346	-15886.76316	-15886.15337	-15887.32963	-15890.44703	-15891.74079
5.86	-15879.40638	-15885.05194	-15886.23703	-15885.56796	-15886.75796	-15886.14788	-15887.32406	-15890.44117	-15891.73519
5.95	-15879.40313	-15885.04628	-15886.23152	-15885.56248	-15886.75271	-15886.14240	-15887.31854	-15890.43540	-15891.72965
6.05	-15879.40009	-15885.04080	-15886.22619	-15885.55715	-15886.74762	-15886.13712	-15887.31326	-15890.42986	-15891.72436
6.14	-15879.39725	-15885.03571	-15886.22121	-15885.55219	-15886.74285	-15886.13228	-15887.30842	-15890.42482	-15891.71953
6.24	-15879.39457	-15885.03111	-15886.21671	-15885.54774	-15886.73855	-15886.12798	-15887.30416	-15890.42038	-15891.71527
6.33	-15879.39204	-15885.02714	-15886.21282	-15885.54390	-15886.73481	-15886.12434	-15887.30055	-15890.41662	-15891.71164
6.43	-15879.38964	-15885.02376	-15886.20949	-15885.54064	-15886.73160	-15886.12134	-15887.29758	-15890.41349	-15891.70861
6.52	-15879.38736	-15885.02082	-15886.20659	-15885.53781	-15886.72880	-15886.11882	-15887.29509	-15890.41084	-15891.70602
6.61	-15879.38520	-15885.01822	-15886.20403	-15885.53532	-15886.72632	-15886.11667	-15887.29298	-15890.40855	-15891.70378
6.71	-15879.38315	-15885.01591	-15886.20174	-15885.53310	-15886.72409	-15886.11482	-15887.29119	-15890.40658	-15891.70183
6.80	-15879.38123	-15885.01384	-15886.19970	-15885.53112	-15886.72210	-15886.11325	-15887.28969	-15890.40488	-15891.70014
6.90	-15879.37943	-15885.01197	-15886.19786	-15885.52935	-15886.72030	-15886.11189	-15887.28842	-15890.40341	-15891.69866
6.99	-15879.37776	-15885.01028	-15886.19620	-15885.52775	-15886.71866	-15886.11072	-15887.28735	-15890.40212	-15891.69737
7.09	-15879.37622	-15885.00872	-15886.19467	-15885.52628	-15886.71716	-15886.10969	-15887.28643	-15890.40099	-15891.69621
7.18	-15879.37480	-15885.00728	-15886.19326	-15885.52493	-15886.71576	-15886.10878	-15887.28564	-15890.39998	-15891.69517
7.28	-15879.37352	-15885.00593	-15886.19193	-15885.52366	-15886.71445	-15886.10794	-15887.28493	-15890.39905	-15891.69420
7.37	-15879.37236	-15885.00463	-15886.19066	-15885.52246	-15886.71320	-15886.10715	-15887.28427	-15890.39819	-15891.69330
7.46	-15879.37132	-15885.00340	-15886.18944	-15885.52131	-15886.71201	-15886.10639	-15887.28364	-15890.39738	-15891.69244
7.56	-15879.37039	-15885.00221	-15886.18828	-15885.52021	-15886.71087	-15886.10566	-15887.28304	-15890.39661	-15891.69162
7.65	-15879.36957	-15885.00108	-15886.18716	-15885.51916	-15886.70977	-15886.10494	-15887.28244	-15890.39587	-15891.69083
7.75	-15879.36885	-15884.99999	-15886.18609	-15885.51816	-15886.70873	-15886.10424	-15887.28186	-15890.39517	-15891.69007
7.84	-15879.36821	-15884.99897	-15886.18507	-15885.51721	-15886.70775	-15886.10357	-15887.28129	-15890.39451	-15891.68935
7.94	-15879.36766	-15884.99801	-15886.18412	-15885.51634	-15886.70684	-15886.10294	-15887.28075	-15890.39389	-15891.68868
8.03	-15879.36717	-15884.99710	-15886.18322	-15885.51551	-15886.70598	-15886.10233	-15887.28022	-15890.39332	-15891.68805
8.13	-15879.36674	-15884.99619	-15886.18232	-15885.51469	-15886.70512	-15886.10170	-15887.27968	-15890.39275	-15891.68741
8.22	-15879.36638	-15884.99542	-15886.18154	-15885.51399	-15886.70438	-15886.10116	-15887.27920	-15890.39227	-15891.68687
8.31	-15879.36606	-15884.99479	-15886.18090	-15885.51344	-15886.70380	-15886.10073	-15887.27881	-15890.39189	-15891.68645
8.41	-15879.36578	-15884.99423	-15886.18034	-15885.51295	-15886.70329	-15886.10036	-15887.27846	-15890.39157	-15891.68609
8.50	-15879.36552	-15884.99374	-15886.17984	-15885.51253	-15886.70284	-15886.10003	-15887.27815	-15890.39131	-15891.68579
8.60	-15879.36529	-15884.99330	-15886.17939	-15885.51215	-15886.70245	-15886.09973	-15887.27788	-15890.39108	-15891.68552
8.69	-15879.36508	-15884.99289	-15886.17898	-15885.51182	-15886.70209	-15886.09946	-15887.27762	-15890.39089	-15891.68529
8.79	-15879.36489	-15884.99253	-15886.17861	-15885.51152	-15886.70177	-15886.09922	-15887.27740	-15890.39072	-15891.68509
8.88	-15879.36472	-15884.99221	-15886.17828	-15885.51126	-15886.70149	-15886.09902	-15887.27720	-15890.39059	-15891.68492
8.98	-15879.36456	-15884.99193	-15886.17799	-15885.51103	-15886.70125	-15886.09884	-15887.27703	-15890.39048	-15891.68479
9.07	-15879.36442	-15884.99167	-15886.17773	-15885.51083	-15886.70103	-15886.09868	-15887.27687	-15890.39039	-15891.68467
9.17	-15879.36429	-15884.99144	-15886.17748	-15885.51066	-15886.70084	-15886.09853	-15887.27673	-15890.39033	-15891.68458
9.26	-15879.36417	-15884.99124	-15886.17727	-15885.51051	-15886.70067	-15886.09841	-15887.27661	-15890.39028	-15891.68450
9.35	-15879.36407	-15884.99106	-15886.17708	-15885.51037	-15886.70052	-15886.09830	-15887.27650	-15890.39024	-15891.68443
9.45	-15879.36397	-15884.99090	-15886.17691	-15885.51026	-15886.70040	-15886.09821	-15887.27641	-15890.39022	-15891.68438
9.54	-15879.36388	-15884.99075	-15886.17676	-15885.51016	-15886.70028	-15886.09813	-15887.27632	-15890.39020	-15891.68434
9.64	-15879.36379	-15884.99062	-15886.17662	-15885.51008	-15886.70018	-15886.09806	-15887.27625	-15890.39020	-15891.68431
9.73	-15879.36372	-15884.99050	-15886.17649	-15885.51000	-15886.70009	-15886.09800	-15887.27619	-15890.39019	-15891.68429
9.83	-15879.36365	-15884.99040	-15886.17638	-15885.50994	-15886.70001	-15886.09795	-15887.27613	-15890.39020	-15891.68427
9.92	-15879.36358	-15884.99030	-15886.17627	-15885.50988	-15886.69994	-15886.09790	-15887.27608	-15890.39020	-15891.68426
10.02	-15879.36353	-15884.99021	-15886.17618	-15885.50983	-15886.69988	-15886.09786	-15887.27604	-15890.39021	-15891.68425

Table S23: The absolute energies in hartree of the potential energy curve of TaSi in the $^4\Pi$ state with a SP-based wave function and nom-CPO active space.

Distance	SP	tpbe	ftpbe	trevpbe	ftrevpbe	tblyp	ftblyp	torelyp	ftorelyp
10.11	-15879.36347	-15884.99014	-15886.17610	-15885.50979	-15886.69983	-15886.09783	-15887.27601	-15890.39023	-15891.68425
10.20	-15879.36342	-15884.99007	-15886.17602	-15885.50975	-15886.69978	-15886.09781	-15887.27598	-15890.39024	-15891.68425
10.30	-15879.36337	-15884.99001	-15886.17596	-15885.50972	-15886.69975	-15886.09778	-15887.27595	-15890.39026	-15891.68425
10.39	-15879.36333	-15884.98996	-15886.17590	-15885.50970	-15886.69972	-15886.09777	-15887.27593	-15890.39028	-15891.68426
10.49	-15879.36329	-15884.98991	-15886.17585	-15885.50968	-15886.69969	-15886.09776	-15887.27592	-15890.39031	-15891.68427
10.58	-15879.36326	-15884.98987	-15886.17580	-15885.50967	-15886.69967	-15886.09775	-15887.27591	-15890.39033	-15891.68427
10.68	-15879.36322	-15884.98983	-15886.17576	-15885.50965	-15886.69965	-15886.09774	-15887.27590	-15890.39035	-15891.68428
10.77	-15879.36319	-15884.98980	-15886.17572	-15885.50964	-15886.69963	-15886.09774	-15887.27589	-15890.39037	-15891.68429
10.87	-15879.36316	-15884.98976	-15886.17568	-15885.50963	-15886.69961	-15886.09774	-15887.27588	-15890.39039	-15891.68429
10.96	-15879.36313	-15884.98973	-15886.17565	-15885.50962	-15886.69959	-15886.09773	-15887.27588	-15890.39040	-15891.68430
11.05	-15879.36311	-15884.98970	-15886.17561	-15885.50961	-15886.69957	-15886.09774	-15887.27587	-15890.39042	-15891.68430
11.15	-15879.36308	-15884.98968	-15886.17558	-15885.50960	-15886.69955	-15886.09774	-15887.27587	-15890.39043	-15891.68431
11.24	-15879.36306	-15884.98966	-15886.17555	-15885.50960	-15886.69954	-15886.09774	-15887.27587	-15890.39045	-15891.68431
11.34	-15879.36304	-15884.98963	-15886.17553	-15885.50959	-15886.69953	-15886.09774	-15887.27587	-15890.39046	-15891.68432
11.43	-15879.36302	-15884.98962	-15886.17551	-15885.50959	-15886.69952	-15886.09775	-15887.27587	-15890.39047	-15891.68432
11.53	-15879.36301	-15884.98960	-15886.17549	-15885.50959	-15886.69951	-15886.09775	-15887.27588	-15890.39048	-15891.68433
11.62	-15879.36299	-15884.98959	-15886.17547	-15885.50958	-15886.69951	-15886.09776	-15887.27588	-15890.39050	-15891.68433
11.72	-15879.36297	-15884.98958	-15886.17546	-15885.50958	-15886.69951	-15886.09777	-15887.27589	-15890.39051	-15891.68434
11.81	-15879.36296	-15884.98956	-15886.17545	-15885.50958	-15886.69950	-15886.09778	-15887.27589	-15890.39051	-15891.68434
11.91	-15879.36295	-15884.98956	-15886.17544	-15885.50958	-15886.69950	-15886.09778	-15887.27590	-15890.39053	-15891.68434
12.00	-15879.36293	-15884.98955	-15886.17543	-15885.50958	-15886.69950	-15886.09779	-15887.27591	-15890.39053	-15891.68435
12.09	-15879.36292	-15884.98954	-15886.17542	-15885.50958	-15886.69949	-15886.09780	-15887.27592	-15890.39054	-15891.68435
12.19	-15879.36291	-15884.98952	-15886.17540	-15885.50958	-15886.69948	-15886.09781	-15887.27592	-15890.39054	-15891.68434
12.28	-15879.36290	-15884.98952	-15886.17539	-15885.50958	-15886.69948	-15886.09781	-15887.27592	-15890.39055	-15891.68434
12.38	-15879.36289	-15884.98951	-15886.17538	-15885.50957	-15886.69947	-15886.09782	-15887.27593	-15890.39055	-15891.68434
12.47	-15879.36288	-15884.98950	-15886.17537	-15885.50957	-15886.69946	-15886.09783	-15887.27593	-15890.39056	-15891.68434
12.57	-15879.36287	-15884.98949	-15886.17536	-15885.50957	-15886.69946	-15886.09783	-15887.27594	-15890.39056	-15891.68434
12.66	-15879.36287	-15884.98949	-15886.17535	-15885.50957	-15886.69946	-15886.09784	-15887.27594	-15890.39056	-15891.68434
12.76	-15879.36286	-15884.98948	-15886.17534	-15885.50957	-15886.69945	-15886.09785	-15887.27595	-15890.39056	-15891.68433
12.85	-15879.36285	-15884.98947	-15886.17534	-15885.50956	-15886.69945	-15886.09785	-15887.27595	-15890.39056	-15891.68433
12.94	-15879.36284	-15884.98947	-15886.17533	-15885.50956	-15886.69945	-15886.09786	-15887.27596	-15890.39056	-15891.68433
13.04	-15879.36284	-15884.98947	-15886.17533	-15885.50956	-15886.69945	-15886.09787	-15887.27596	-15890.39056	-15891.68433
13.13	-15879.36283	-15884.98947	-15886.17533	-15885.50956	-15886.69945	-15886.09787	-15887.27597	-15890.39056	-15891.68433
13.23	-15879.36283	-15884.98946	-15886.17533	-15885.50956	-15886.69945	-15886.09788	-15887.27598	-15890.39056	-15891.68433
13.32	-15879.36282	-15884.98946	-15886.17532	-15885.50956	-15886.69944	-15886.09789	-15887.27599	-15890.39057	-15891.68433
13.42	-15879.36282	-15884.98946	-15886.17532	-15885.50956	-15886.69944	-15886.09789	-15887.27599	-15890.39057	-15891.68433
13.51	-15879.36281	-15884.98946	-15886.17532	-15885.50956	-15886.69944	-15886.09790	-15887.27600	-15890.39057	-15891.68433
13.61	-15879.36281	-15884.98945	-15886.17531	-15885.50956	-15886.69944	-15886.09790	-15887.27600	-15890.39057	-15891.68433
13.70	-15879.36280	-15884.98945	-15886.17531	-15885.50956	-15886.69944	-15886.09791	-15887.27601	-15890.39057	-15891.68432
13.80	-15879.36280	-15884.98945	-15886.17531	-15885.50956	-15886.69943	-15886.09791	-15887.27601	-15890.39057	-15891.68432
13.89	-15879.36279	-15884.98944	-15886.17530	-15885.50956	-15886.69943	-15886.09792	-15887.27601	-15890.39057	-15891.68432
13.98	-15879.36279	-15884.98944	-15886.17530	-15885.50956	-15886.69943	-15886.09792	-15887.27601	-15890.39057	-15891.68432
14.08	-15879.36279	-15884.98944	-15886.17530	-15885.50956	-15886.69942	-15886.09792	-15887.27602	-15890.39057	-15891.68431
14.17	-15879.36278	-15884.98944	-15886.17529	-15885.50955	-15886.69942	-15886.09793	-15887.27602	-15890.39057	-15891.68431
14.27	-15879.36278	-15884.98944	-15886.17529	-15885.50955	-15886.69942	-15886.09793	-15887.27602	-15890.39057	-15891.68431
14.36	-15879.36278	-15884.98943	-15886.17529	-15885.50955	-15886.69942	-15886.09793	-15887.27602	-15890.39056	-15891.68431
14.46	-15879.36278	-15884.98943	-15886.17529	-15885.50955	-15886.69942	-15886.09793	-15887.27603	-15890.39056	-15891.68431
14.55	-15879.36277	-15884.98943	-15886.17529	-15885.50955	-15886.69942	-15886.09794	-15887.27603	-15890.39056	-15891.68431
14.65	-15879.36277	-15884.98943	-15886.17528	-15885.50955	-15886.69942	-15886.09794	-15887.27603	-15890.39056	-15891.68430
14.74	-15879.36277	-15884.98943	-15886.17528	-15885.50955	-15886.69942	-15886.09794	-15887.27604	-15890.39056	-15891.68430
14.83	-15879.36277	-15884.98943	-15886.17528	-15885.50955	-15886.69942	-15886.09795	-15887.27604	-15890.39056	-15891.68430
14.93	-15879.36276	-15884.98943	-15886.17528	-15885.50955	-15886.69942	-15886.09795	-15887.27604	-15890.39056	-15891.68430

Table S23: The absolute energies in hartree of the potential energy curve of TaSi in the $^4\Pi$ state with a SP-based wave function and nom-CPO active space.

Distance	SP	tpbe	ftpbe	trevpbe	ftrevpbe	tblyp	ftblyp	torelyp	ftorelyp
15.02	-15879.36276	-15884.98943	-15886.17528	-15885.50955	-15886.69942	-15886.09795	-15887.27605	-15890.39056	-15891.68430
15.12	-15879.36276	-15884.98943	-15886.17528	-15885.50955	-15886.69942	-15886.09796	-15887.27605	-15890.39056	-15891.68430
15.21	-15879.36276	-15884.98943	-15886.17528	-15885.50955	-15886.69942	-15886.09796	-15887.27605	-15890.39056	-15891.68430
15.31	-15879.36276	-15884.98943	-15886.17528	-15885.50955	-15886.69941	-15886.09796	-15887.27605	-15890.39056	-15891.68430
15.40	-15879.36275	-15884.98943	-15886.17528	-15885.50955	-15886.69941	-15886.09796	-15887.27605	-15890.39056	-15891.68430
15.50	-15879.36275	-15884.98943	-15886.17528	-15885.50955	-15886.69941	-15886.09796	-15887.27606	-15890.39056	-15891.68430
15.59	-15879.36275	-15884.98943	-15886.17528	-15885.50955	-15886.69941	-15886.09797	-15887.27606	-15890.39056	-15891.68430
15.68	-15879.36275	-15884.98943	-15886.17528	-15885.50955	-15886.69941	-15886.09797	-15887.27606	-15890.39056	-15891.68430
15.78	-15879.36275	-15884.98942	-15886.17528	-15885.50955	-15886.69941	-15886.09797	-15887.27606	-15890.39056	-15891.68430
15.87	-15879.36275	-15884.98942	-15886.17528	-15885.50955	-15886.69941	-15886.09797	-15887.27606	-15890.39056	-15891.68430
15.97	-15879.36275	-15884.98942	-15886.17528	-15885.50954	-15886.69941	-15886.09797	-15887.27606	-15890.39056	-15891.68429
16.06	-15879.36274	-15884.98942	-15886.17528	-15885.50954	-15886.69941	-15886.09797	-15887.27606	-15890.39056	-15891.68430
16.16	-15879.36274	-15884.98942	-15886.17528	-15885.50954	-15886.69941	-15886.09797	-15887.27606	-15890.39056	-15891.68429
16.25	-15879.36274	-15884.98942	-15886.17528	-15885.50954	-15886.69941	-15886.09797	-15887.27606	-15890.39056	-15891.68430
16.35	-15879.36274	-15884.98942	-15886.17528	-15885.50954	-15886.69941	-15886.09797	-15887.27606	-15890.39056	-15891.68429
16.44	-15879.36274	-15884.98942	-15886.17528	-15885.50954	-15886.69941	-15886.09798	-15887.27607	-15890.39056	-15891.68429
16.54	-15879.36274	-15884.98943	-15886.17528	-15885.50954	-15886.69941	-15886.09798	-15887.27607	-15890.39056	-15891.68429
16.63	-15879.36274	-15884.98942	-15886.17528	-15885.50955	-15886.69941	-15886.09798	-15887.27607	-15890.39056	-15891.68430
16.72	-15879.36274	-15884.98942	-15886.17528	-15885.50954	-15886.69941	-15886.09798	-15887.27607	-15890.39056	-15891.68430
16.82	-15879.36274	-15884.98942	-15886.17528	-15885.50955	-15886.69941	-15886.09798	-15887.27607	-15890.39056	-15891.68430
16.91	-15879.36274	-15884.98943	-15886.17528	-15885.50955	-15886.69941	-15886.09798	-15887.27607	-15890.39056	-15891.68430
17.01	-15879.36273	-15884.98943	-15886.17528	-15885.50955	-15886.69941	-15886.09799	-15887.27608	-15890.39056	-15891.68430
17.10	-15879.36273	-15884.98943	-15886.17528	-15885.50955	-15886.69941	-15886.09799	-15887.27608	-15890.39056	-15891.68430
17.20	-15879.36273	-15884.98943	-15886.17528	-15885.50955	-15886.69941	-15886.09799	-15887.27608	-15890.39056	-15891.68430
17.29	-15879.36273	-15884.98943	-15886.17528	-15885.50955	-15886.69941	-15886.09799	-15887.27608	-15890.39056	-15891.68430
17.39	-15879.36273	-15884.98943	-15886.17528	-15885.50955	-15886.69941	-15886.09799	-15887.27608	-15890.39056	-15891.68430
17.48	-15879.36273	-15884.98943	-15886.17528	-15885.50955	-15886.69941	-15886.09799	-15887.27608	-15890.39056	-15891.68430
17.57	-15879.36273	-15884.98943	-15886.17528	-15885.50955	-15886.69941	-15886.09799	-15887.27608	-15890.39056	-15891.68430
17.67	-15879.36273	-15884.98943	-15886.17528	-15885.50955	-15886.69941	-15886.09799	-15887.27608	-15890.39056	-15891.68430
17.76	-15879.36273	-15884.98943	-15886.17528	-15885.50955	-15886.69941	-15886.09799	-15887.27608	-15890.39056	-15891.68430
17.86	-15879.36273	-15884.98943	-15886.17528	-15885.50955	-15886.69941	-15886.09799	-15887.27608	-15890.39056	-15891.68430
17.95	-15879.36273	-15884.98943	-15886.17528	-15885.50955	-15886.69941	-15886.09799	-15887.27608	-15890.39056	-15891.68430
18.05	-15879.36273	-15884.98943	-15886.17528	-15885.50955	-15886.69941	-15886.09799	-15887.27608	-15890.39056	-15891.68429

Table S24: The absolute energies in hartree of the potential energy curve of TaSi in the $^6\Sigma^+$ state with a SP-based wave function and nom-CPO active space.

Distance	SP	tpbe	ftpbe	trevpbe	ftrevpbe	tblyp	ftblyp	torelyp	ftorelyp
1.98	-15875.44003	-15881.16861	-15882.35914	-15881.66691	-15882.86162	-15882.23302	-15883.41624	-15886.59227	-15887.88893
2.08	-15876.16578	-15881.88686	-15883.07726	-15882.38627	-15883.58103	-15882.95416	-15884.13705	-15887.30498	-15888.60182
2.17	-15876.74344	-15882.45777	-15883.64823	-15882.95821	-15884.15322	-15883.52756	-15884.71028	-15887.87118	-15889.16839
2.27	-15877.20408	-15882.91258	-15884.10323	-15883.41398	-15884.60939	-15883.98454	-15885.16724	-15888.32200	-15889.61971
2.36	-15877.57283	-15883.27685	-15884.46778	-15883.77915	-15884.97503	-15884.35069	-15885.53349	-15888.68295	-15889.98122
2.46	-15877.86976	-15883.57054	-15884.76176	-15884.07368	-15885.27000	-15884.64603	-15885.82898	-15888.97390	-15890.27268
2.55	-15878.11065	-15883.80933	-15885.00077	-15884.31325	-15885.50991	-15884.88626	-15886.06935	-15889.21046	-15890.50961
2.65	-15878.30775	-15884.00543	-15885.19699	-15884.51009	-15885.70692	-15885.08362	-15886.26677	-15889.40478	-15890.70409
2.74	-15878.47051	-15884.16772	-15885.35927	-15884.67309	-15885.86989	-15885.24701	-15886.43012	-15889.56565	-15890.86489
2.83	-15878.60618	-15884.30324	-15885.49464	-15884.80930	-15886.00587	-15885.38348	-15886.56646	-15889.70006	-15891.04571
2.93	-15878.79253	-15884.47133	-15885.66131	-15884.97789	-15886.17329	-15885.55253	-15886.73208	-15889.81294	-15891.16830
3.02	-15878.90028	-15884.58089	-15885.77040	-15885.08784	-15886.28327	-15885.66204	-15886.84077	-15889.97555	-15891.27538
3.12	-15878.99292	-15884.67540	-15885.86419	-15885.18273	-15886.37765	-15885.75679	-15886.93472	-15890.06796	-15891.36733

Table S24: The absolute energies in hartree of the potential energy curve of TaSi in the $^6\Sigma^+$ state with a SP-based wave function and nom-CPO active space.

Distance	SP	tpbe	ftpbe	trevpbe	ftrevpbe	tblyp	ftblyp	torelyp	ftorelyp
3.21	-15879.07227	-15884.75510	-15885.94315	-15885.26283	-15886.45707	-15885.83692	-15887.01414	-15890.14615	-15891.44482
3.31	-15879.13993	-15884.82160	-15886.00902	-15885.32975	-15886.52337	-15885.90392	-15887.08059	-15890.21152	-15891.50956
3.40	-15879.19731	-15884.87720	-15886.06410	-15885.38578	-15886.57887	-15885.96004	-15887.13627	-15890.26622	-15891.56375
3.50	-15879.24570	-15884.92369	-15886.11016	-15885.43269	-15886.62533	-15886.00706	-15887.18293	-15890.31194	-15891.60906
3.59	-15879.28629	-15884.96232	-15886.14841	-15885.47174	-15886.66399	-15886.04625	-15887.22180	-15890.34993	-15891.64668
3.68	-15879.32011	-15884.99425	-15886.18001	-15885.50406	-15886.69596	-15886.07873	-15887.25400	-15890.38130	-15891.67773
3.78	-15879.34807	-15885.02049	-15886.20596	-15885.53068	-15886.72227	-15886.10553	-15887.28054	-15890.40708	-15891.70322
3.87	-15879.37097	-15885.04182	-15886.22703	-15885.55237	-15886.74368	-15886.12741	-15887.30221	-15890.42801	-15891.72389
3.97	-15879.38950	-15885.05888	-15886.24385	-15885.56977	-15886.76083	-15886.14502	-15887.31963	-15890.44474	-15891.74038
4.06	-15879.40428	-15885.07229	-15886.25707	-15885.58351	-15886.77435	-15886.15899	-15887.33343	-15890.45788	-15891.75331
4.16	-15879.41584	-15885.08266	-15886.26727	-15885.59419	-15886.78483	-15886.16991	-15887.34421	-15890.46804	-15891.76328
4.35	-15879.42464	-15885.09043	-15886.27489	-15885.60225	-15886.79271	-15886.17822	-15887.35240	-15890.47565	-15891.77071
4.44	-15879.43110	-15885.09594	-15886.28027	-15885.60804	-15886.79834	-15886.18427	-15887.35835	-15890.48104	-15891.77595
4.54	-15879.43557	-15885.09954	-15886.28376	-15885.61189	-15886.80207	-15886.18839	-15887.36240	-15890.48455	-15891.77933
4.63	-15879.43836	-15885.10156	-15886.28570	-15885.61416	-15886.80423	-15886.19095	-15887.36489	-15890.48653	-15891.78120
4.72	-15879.43974	-15885.10229	-15886.28637	-15885.61513	-15886.80510	-15886.19221	-15887.36611	-15890.48726	-15891.78183
4.82	-15879.43994	-15885.10193	-15886.28596	-15885.61499	-15886.80489	-15886.19237	-15887.36625	-15890.48692	-15891.78141
4.91	-15879.43916	-15885.10063	-15886.28463	-15885.61390	-15886.80374	-15886.19158	-15887.36545	-15890.48567	-15891.78008
5.01	-15879.43759	-15885.09856	-15886.28255	-15885.61203	-15886.80183	-15886.19002	-15887.36390	-15890.48367	-15891.77804
5.10	-15879.43537	-15885.09590	-15886.27989	-15885.60956	-15886.79934	-15886.18787	-15887.36176	-15890.48110	-15891.77543
5.20	-15879.43263	-15885.09277	-15886.27679	-15885.60662	-15886.79639	-15886.18524	-15887.35916	-15890.47809	-15891.77239
5.29	-15879.42949	-15885.08927	-15886.27332	-15885.60330	-15886.79308	-15886.18222	-15887.35619	-15890.47471	-15891.76900
5.39	-15879.42605	-15885.08544	-15886.26954	-15885.59964	-15886.78944	-15886.17886	-15887.35289	-15890.47101	-15891.76531
5.48	-15879.42237	-15885.08134	-15886.26551	-15885.59571	-15886.78555	-15886.17523	-15887.34933	-15890.46706	-15891.76137
5.57	-15879.41854	-15885.07705	-15886.26130	-15885.59159	-15886.78148	-15886.17139	-15887.34558	-15890.46293	-15891.75727
5.67	-15879.41462	-15885.07260	-15886.25695	-15885.58730	-15886.77727	-15886.16738	-15887.34167	-15890.45864	-15891.75304
5.76	-15879.41065	-15885.06802	-15886.25248	-15885.58288	-15886.77295	-15886.16320	-15887.33761	-15890.45422	-15891.74868
5.86	-15879.40668	-15885.06324	-15886.24785	-15885.57828	-15886.76846	-15886.15883	-15887.33338	-15890.44961	-15891.74416
5.95	-15879.40275	-15885.05818	-15886.24296	-15885.57339	-15886.76373	-15886.15413	-15887.32885	-15890.44469	-15891.73938
6.05	-15879.39891	-15885.05264	-15886.23762	-15885.56804	-15886.75857	-15886.14891	-15887.32385	-15890.43928	-15891.73414
6.14	-15879.39520	-15885.04652	-15886.23173	-15885.56212	-15886.75287	-15886.14308	-15887.31825	-15890.43327	-15891.72835
6.24	-15879.39167	-15885.04037	-15886.22579	-15885.55618	-15886.74712	-15886.13722	-15887.31262	-15890.42731	-15891.72258
6.33	-15879.38832	-15885.03475	-15886.22035	-15885.55076	-15886.74184	-15886.13193	-15887.30754	-15890.42196	-15891.71741
6.43	-15879.38517	-15885.02972	-15886.21546	-15885.54591	-15886.73709	-15886.12727	-15887.30308	-15890.41728	-15891.71287
6.52	-15879.38221	-15885.02523	-15886.21110	-15885.54159	-15886.73284	-15886.12319	-15887.29921	-15890.41319	-15891.70890
6.61	-15879.37944	-15885.02122	-15886.20721	-15885.53773	-15886.72905	-15886.11964	-15887.29587	-15890.40961	-15891.70543
6.71	-15879.37689	-15885.01764	-15886.20375	-15885.53430	-15886.72565	-15886.11658	-15887.29305	-15890.40649	-15891.70242
6.80	-15879.37456	-15885.01445	-15886.20067	-15885.53123	-15886.72261	-15886.11396	-15887.29070	-15890.40379	-15891.69980
6.90	-15879.37247	-15885.01156	-15886.19791	-15885.52847	-15886.71985	-15886.11172	-15887.28878	-15890.40142	-15891.69752
6.99	-15879.37065	-15885.00890	-15886.19538	-15885.52592	-15886.71732	-15886.10979	-15887.28719	-15890.39932	-15891.69548
7.09	-15879.36910	-15885.00644	-15886.19305	-15885.52357	-15886.71498	-15886.10806	-15887.28581	-15890.39742	-15891.69362
7.18	-15879.36781	-15885.00420	-15886.19092	-15885.52143	-15886.71285	-15886.10647	-15887.28450	-15890.39570	-15891.69192
7.28	-15879.36672	-15885.00221	-15886.18900	-15885.51953	-15886.71097	-15886.10503	-15887.28325	-15890.39416	-15891.69040
7.37	-15879.36579	-15885.00045	-15886.18729	-15885.51786	-15886.70931	-15886.10372	-15887.28208	-15890.39279	-15891.68903
7.46	-15879.36498	-15884.99889	-15886.18577	-15885.51640	-15886.70784	-15886.10256	-15887.28101	-15890.39158	-15891.68782
7.56	-15879.36428	-15884.99752	-15886.18441	-15885.51510	-15886.70654	-15886.10152	-15887.28004	-15890.39051	-15891.68674
7.65	-15879.36365	-15884.99628	-15886.18318	-15885.51395	-15886.70538	-15886.10059	-15887.27916	-15890.38957	-15891.68578
7.75	-15879.36310	-15884.99518	-15886.18208	-15885.51292	-15886.70434	-15886.09975	-15887.27836	-15890.38873	-15891.68493
7.84	-15879.36261	-15884.99418	-15886.18108	-15885.51200	-15886.70341	-15886.09901	-15887.27764	-15890.38800	-15891.68417
7.94	-15879.36216	-15884.99328	-15886.18018	-15885.51118	-15886.70256	-15886.09834	-15887.27699	-15890.38735	-15891.68349
8.03	-15879.36176	-15884.99246	-15886.17936	-15885.51044	-15886.70181	-15886.09773	-15887.27640	-15890.38678	-15891.68289
8.13	-15879.36140	-15884.99173	-15886.17861	-15885.50977	-15886.70113	-15886.09719	-15887.27586	-15890.38628	-15891.68236

Table S24: The absolute energies in hartree of the potential energy curve of TaSi in the $^6\Sigma^+$ state with a SP-based wave function and nom-CPO active space.

Distance	SP	tpbe	ftpbe	trevpbe	ftrevpbe	tblyp	ftblyp	torelyp	ftorelyp
8.22	-15879.36108	-15884.99106	-15886.17793	-15885.50917	-15886.70051	-15886.09670	-15887.27538	-15890.38583	-15891.68190
8.31	-15879.36079	-15884.99045	-15886.17732	-15885.50864	-15886.69996	-15886.09626	-15887.27494	-15890.38545	-15891.68149
8.41	-15879.36052	-15884.98991	-15886.17676	-15885.50816	-15886.69946	-15886.09587	-15887.27455	-15890.38512	-15891.68113
8.50	-15879.36028	-15884.98942	-15886.17626	-15885.50774	-15886.69902	-15886.09551	-15887.27420	-15890.38484	-15891.68082
8.60	-15879.36006	-15884.98897	-15886.17580	-15885.50736	-15886.69862	-15886.09520	-15887.27388	-15890.38460	-15891.68054
8.69	-15879.35986	-15884.98857	-15886.17539	-15885.50702	-15886.69826	-15886.09492	-15887.27359	-15890.38439	-15891.68030
8.79	-15879.35967	-15884.98821	-15886.17501	-15885.50671	-15886.69794	-15886.09466	-15887.27333	-15890.38421	-15891.68010
8.88	-15879.35951	-15884.98788	-15886.17467	-15885.50645	-15886.69765	-15886.09444	-15887.27310	-15890.38406	-15891.67993
8.98	-15879.35935	-15884.98759	-15886.17436	-15885.50621	-15886.69740	-15886.09424	-15887.27289	-15890.38394	-15891.67978
9.07	-15879.35922	-15884.98732	-15886.17409	-15885.50600	-15886.69717	-15886.09407	-15887.27271	-15890.38385	-15891.67966
9.17	-15879.35909	-15884.98708	-15886.17384	-15885.50582	-15886.69697	-15886.09391	-15887.27254	-15890.38377	-15891.67955
9.26	-15879.35897	-15884.98686	-15886.17361	-15885.50565	-15886.69679	-15886.09377	-15887.27239	-15890.38370	-15891.67947
9.35	-15879.35886	-15884.98667	-15886.17340	-15885.50551	-15886.69663	-15886.09364	-15887.27226	-15890.38366	-15891.67939
9.45	-15879.35877	-15884.98650	-15886.17322	-15885.50539	-15886.69649	-15886.09354	-15887.27215	-15890.38362	-15891.67933
9.54	-15879.35868	-15884.98634	-15886.17305	-15885.50527	-15886.69637	-15886.09344	-15887.27204	-15890.38360	-15891.67928
9.64	-15879.35859	-15884.98620	-15886.17290	-15885.50518	-15886.69626	-15886.09337	-15887.27195	-15890.38358	-15891.67924
9.73	-15879.35852	-15884.98607	-15886.17276	-15885.50509	-15886.69615	-15886.09329	-15887.27187	-15890.38358	-15891.67921
9.83	-15879.35845	-15884.98595	-15886.17263	-15885.50502	-15886.69606	-15886.09323	-15887.27179	-15890.38357	-15891.67918
9.92	-15879.35838	-15884.98585	-15886.17252	-15885.50495	-15886.69598	-15886.09318	-15887.27173	-15890.38357	-15891.67917
10.02	-15879.35832	-15884.98576	-15886.17242	-15885.50490	-15886.69591	-15886.09313	-15887.27168	-15890.38358	-15891.67916
10.11	-15879.35827	-15884.98568	-15886.17232	-15885.50485	-15886.69585	-15886.09309	-15887.27163	-15890.38359	-15891.67915
10.20	-15879.35822	-15884.98560	-15886.17224	-15885.50481	-15886.69580	-15886.09306	-15887.27159	-15890.38360	-15891.67915
10.30	-15879.35817	-15884.98554	-15886.17217	-15885.50477	-15886.69576	-15886.09303	-15887.27156	-15890.38361	-15891.67914
10.39	-15879.35813	-15884.98548	-15886.17210	-15885.50474	-15886.69572	-15886.09301	-15887.27153	-15890.38363	-15891.67914
10.49	-15879.35809	-15884.98542	-15886.17204	-15885.50472	-15886.69568	-15886.09299	-15887.27150	-15890.38365	-15891.67914
10.58	-15879.35805	-15884.98537	-15886.17199	-15885.50469	-15886.69565	-15886.09297	-15887.27148	-15890.38367	-15891.67914
10.68	-15879.35802	-15884.98533	-15886.17194	-15885.50468	-15886.69563	-15886.09297	-15887.27147	-15890.38369	-15891.67915
10.77	-15879.35798	-15884.98529	-15886.17190	-15885.50466	-15886.69560	-15886.09296	-15887.27145	-15890.38370	-15891.67915
10.87	-15879.35796	-15884.98525	-15886.17185	-15885.50465	-15886.69558	-15886.09295	-15887.27144	-15890.38372	-15891.67915
10.96	-15879.35793	-15884.98522	-15886.17181	-15885.50463	-15886.69555	-15886.09295	-15887.27143	-15890.38373	-15891.67915
11.05	-15879.35790	-15884.98519	-15886.17177	-15885.50462	-15886.69553	-15886.09295	-15887.27142	-15890.38374	-15891.67915
11.15	-15879.35788	-15884.98516	-15886.17173	-15885.50461	-15886.69551	-15886.09294	-15887.27141	-15890.38376	-15891.67915
11.24	-15879.35786	-15884.98513	-15886.17170	-15885.50460	-15886.69549	-15886.09295	-15887.27141	-15890.38377	-15891.67915
11.34	-15879.35784	-15884.98511	-15886.17168	-15885.50459	-15886.69548	-15886.09295	-15887.27140	-15890.38378	-15891.67915
11.43	-15879.35782	-15884.98509	-15886.17165	-15885.50458	-15886.69546	-15886.09295	-15887.27140	-15890.38379	-15891.67915
11.53	-15879.35780	-15884.98507	-15886.17163	-15885.50458	-15886.69545	-15886.09295	-15887.27140	-15890.38380	-15891.67916
11.62	-15879.35778	-15884.98505	-15886.17161	-15885.50457	-15886.69545	-15886.09296	-15887.27141	-15890.38381	-15891.67916
11.72	-15879.35777	-15884.98504	-15886.17160	-15885.50457	-15886.69544	-15886.09296	-15887.27141	-15890.38382	-15891.67916
11.81	-15879.35775	-15884.98502	-15886.17158	-15885.50457	-15886.69544	-15886.09297	-15887.27141	-15890.38382	-15891.67916
11.91	-15879.35774	-15884.98501	-15886.17157	-15885.50456	-15886.69543	-15886.09297	-15887.27142	-15890.38383	-15891.67916
12.00	-15879.35772	-15884.98500	-15886.17155	-15885.50456	-15886.69542	-15886.09298	-15887.27142	-15890.38384	-15891.67916
12.09	-15879.35771	-15884.98499	-15886.17154	-15885.50456	-15886.69541	-15886.09299	-15887.27142	-15890.38384	-15891.67916
12.19	-15879.35770	-15884.98498	-15886.17152	-15885.50455	-15886.69540	-15886.09299	-15887.27143	-15890.38385	-15891.67916
12.28	-15879.35769	-15884.98496	-15886.17151	-15885.50455	-15886.69539	-15886.09300	-15887.27143	-15890.38385	-15891.67915
12.38	-15879.35768	-15884.98495	-15886.17149	-15885.50454	-15886.69538	-15886.09300	-15887.27143	-15890.38385	-15891.67915
12.47	-15879.35767	-15884.98494	-15886.17148	-15885.50454	-15886.69537	-15886.09301	-15887.27143	-15890.38385	-15891.67915
12.57	-15879.35766	-15884.98493	-15886.17147	-15885.50453	-15886.69537	-15886.09301	-15887.27144	-15890.38386	-15891.67914
12.66	-15879.35765	-15884.98493	-15886.17146	-15885.50453	-15886.69536	-15886.09302	-15887.27144	-15890.38386	-15891.67914
12.76	-15879.35765	-15884.98492	-15886.17145	-15885.50453	-15886.69536	-15886.09302	-15887.27145	-15890.38386	-15891.67914
12.85	-15879.35764	-15884.98491	-15886.17145	-15885.50453	-15886.69535	-15886.09303	-15887.27145	-15890.38386	-15891.67913
12.94	-15879.35763	-15884.98491	-15886.17144	-15885.50452	-15886.69535	-15886.09303	-15887.27146	-15890.38386	-15891.67913
13.04	-15879.35763	-15884.98490	-15886.17143	-15885.50452	-15886.69535	-15886.09304	-15887.27146	-15890.38386	-15891.67913

Table S24: The absolute energies in hartree of the potential energy curve of TaSi in the $^6\Sigma^+$ state with a SP-based wave function and nom-CPO active space.

Distance	SP	tpbe	ftpbe	trevpbe	ftrevpbe	tblyp	ftblyp	torelyp	ftorelyp
13.13	-15879.35762	-15884.98490	-15886.17143	-15885.50452	-15886.69535	-15886.09305	-15887.27147	-15890.38386	-15891.67913
13.23	-15879.35761	-15884.98490	-15886.17143	-15885.50452	-15886.69534	-15886.09305	-15887.27147	-15890.38386	-15891.67913
13.32	-15879.35761	-15884.98489	-15886.17142	-15885.50452	-15886.69534	-15886.09306	-15887.27148	-15890.38386	-15891.67913
13.42	-15879.35760	-15884.98489	-15886.17142	-15885.50452	-15886.69534	-15886.09306	-15887.27148	-15890.38386	-15891.67912
13.51	-15879.35760	-15884.98489	-15886.17141	-15885.50452	-15886.69533	-15886.09307	-15887.27149	-15890.38386	-15891.67912
13.61	-15879.35759	-15884.98488	-15886.17141	-15885.50451	-15886.69533	-15886.09307	-15887.27149	-15890.38386	-15891.67912
13.70	-15879.35759	-15884.98488	-15886.17140	-15885.50451	-15886.69533	-15886.09308	-15887.27149	-15890.38386	-15891.67911
13.80	-15879.35758	-15884.98488	-15886.17140	-15885.50451	-15886.69532	-15886.09308	-15887.27150	-15890.38386	-15891.67911
13.89	-15879.35758	-15884.98487	-15886.17139	-15885.50451	-15886.69532	-15886.09308	-15887.27150	-15890.38385	-15891.67911
13.98	-15879.35758	-15884.98487	-15886.17139	-15885.50450	-15886.69531	-15886.09308	-15887.27150	-15890.38385	-15891.67910
14.08	-15879.35757	-15884.98486	-15886.17139	-15885.50450	-15886.69531	-15886.09309	-15887.27150	-15890.38385	-15891.67910
14.17	-15879.35757	-15884.98486	-15886.17138	-15885.50450	-15886.69531	-15886.09309	-15887.27150	-15890.38385	-15891.67910
14.27	-15879.35757	-15884.98486	-15886.17138	-15885.50450	-15886.69531	-15886.09309	-15887.27151	-15890.38385	-15891.67909
14.36	-15879.35756	-15884.98486	-15886.17138	-15885.50450	-15886.69530	-15886.09309	-15887.27151	-15890.38385	-15891.67909
14.46	-15879.35756	-15884.98486	-15886.17138	-15885.50450	-15886.69530	-15886.09310	-15887.27151	-15890.38385	-15891.67909
14.55	-15879.35756	-15884.98486	-15886.17138	-15885.50450	-15886.69530	-15886.09310	-15887.27152	-15890.38385	-15891.67909
14.65	-15879.35755	-15884.98486	-15886.17138	-15885.50450	-15886.69530	-15886.09310	-15887.27152	-15890.38385	-15891.67909
14.74	-15879.35755	-15884.98486	-15886.17137	-15885.50450	-15886.69530	-15886.09311	-15887.27152	-15890.38385	-15891.67909
14.83	-15879.35755	-15884.98485	-15886.17137	-15885.50450	-15886.69530	-15886.09311	-15887.27153	-15890.38385	-15891.67909
14.93	-15879.35755	-15884.98485	-15886.17137	-15885.50450	-15886.69530	-15886.09311	-15887.27153	-15890.38385	-15891.67909
15.02	-15879.35755	-15884.98485	-15886.17137	-15885.50450	-15886.69530	-15886.09312	-15887.27153	-15890.38385	-15891.67909
15.12	-15879.35754	-15884.98485	-15886.17137	-15885.50450	-15886.69530	-15886.09312	-15887.27153	-15890.38385	-15891.67909
15.21	-15879.35754	-15884.98485	-15886.17137	-15885.50449	-15886.69530	-15886.09312	-15887.27154	-15890.38385	-15891.67908
15.31	-15879.35754	-15884.98485	-15886.17137	-15885.50449	-15886.69530	-15886.09312	-15887.27154	-15890.38385	-15891.67908
15.40	-15879.35754	-15884.98485	-15886.17137	-15885.50449	-15886.69529	-15886.09312	-15887.27154	-15890.38385	-15891.67908
15.50	-15879.35754	-15884.98485	-15886.17137	-15885.50449	-15886.69529	-15886.09312	-15887.27154	-15890.38384	-15891.67908
15.59	-15879.35753	-15884.98485	-15886.17136	-15885.50449	-15886.69529	-15886.09313	-15887.27154	-15890.38384	-15891.67908
15.68	-15879.35753	-15884.98485	-15886.17136	-15885.50449	-15886.69529	-15886.09313	-15887.27154	-15890.38384	-15891.67908
15.78	-15879.35753	-15884.98484	-15886.17136	-15885.50449	-15886.69529	-15886.09313	-15887.27154	-15890.38384	-15891.67907
15.87	-15879.35753	-15884.98484	-15886.17136	-15885.50449	-15886.69529	-15886.09313	-15887.27154	-15890.38384	-15891.67907
15.97	-15879.35753	-15884.98484	-15886.17136	-15885.50448	-15886.69528	-15886.09313	-15887.27154	-15890.38384	-15891.67907
16.06	-15879.35753	-15884.98484	-15886.17136	-15885.50448	-15886.69528	-15886.09313	-15887.27154	-15890.38384	-15891.67907
16.16	-15879.35752	-15884.98484	-15886.17136	-15885.50448	-15886.69528	-15886.09313	-15887.27154	-15890.38384	-15891.67907
16.25	-15879.35752	-15884.98484	-15886.17136	-15885.50448	-15886.69528	-15886.09313	-15887.27154	-15890.38384	-15891.67907
16.35	-15879.35752	-15884.98484	-15886.17136	-15885.50449	-15886.69528	-15886.09313	-15887.27155	-15890.38384	-15891.67907
16.44	-15879.35752	-15884.98484	-15886.17136	-15885.50449	-15886.69528	-15886.09314	-15887.27155	-15890.38384	-15891.67907
16.54	-15879.35752	-15884.98484	-15886.17136	-15885.50449	-15886.69528	-15886.09314	-15887.27155	-15890.38384	-15891.67907
16.63	-15879.35752	-15884.98485	-15886.17136	-15885.50449	-15886.69529	-15886.09314	-15887.27155	-15890.38384	-15891.67907
16.72	-15879.35752	-15884.98485	-15886.17136	-15885.50449	-15886.69529	-15886.09314	-15887.27155	-15890.38384	-15891.67907
16.82	-15879.35752	-15884.98485	-15886.17136	-15885.50449	-15886.69529	-15886.09314	-15887.27156	-15890.38385	-15891.67908
16.91	-15879.35752	-15884.98485	-15886.17136	-15885.50449	-15886.69529	-15886.09315	-15887.27156	-15890.38385	-15891.67908
17.01	-15879.35752	-15884.98485	-15886.17137	-15885.50449	-15886.69529	-15886.09315	-15887.27156	-15890.38385	-15891.67908
17.10	-15879.35751	-15884.98485	-15886.17137	-15885.50449	-15886.69529	-15886.09315	-15887.27156	-15890.38385	-15891.67908
17.20	-15879.35751	-15884.98485	-15886.17137	-15885.50449	-15886.69529	-15886.09315	-15887.27156	-15890.38385	-15891.67908
17.29	-15879.35751	-15884.98485	-15886.17137	-15885.50449	-15886.69529	-15886.09315	-15887.27156	-15890.38385	-15891.67908
17.39	-15879.35751	-15884.98485	-15886.17137	-15885.50449	-15886.69529	-15886.09315	-15887.27156	-15890.38385	-15891.67908
17.48	-15879.35751	-15884.98485	-15886.17137	-15885.50449	-15886.69529	-15886.09315	-15887.27156	-15890.38385	-15891.67908
17.57	-15879.35751	-15884.98485	-15886.17137	-15885.50449	-15886.69529	-15886.09315	-15887.27156	-15890.38385	-15891.67908
17.67	-15879.35751	-15884.98485	-15886.17137	-15885.50449	-15886.69529	-15886.09315	-15887.27156	-15890.38385	-15891.67908
17.76	-15879.35751	-15884.98485	-15886.17136	-15885.50449	-15886.69529	-15886.09315	-15887.27156	-15890.38385	-15891.67907
17.86	-15879.35751	-15884.98485	-15886.17136	-15885.50449	-15886.69529	-15886.09315	-15887.27156	-15890.38385	-15891.67907
17.95	-15879.35751	-15884.98485	-15886.17136	-15885.50449	-15886.69529	-15886.09315	-15887.27156	-15890.38385	-15891.67907

Table S24: The absolute energies in hartree of the potential energy curve of TaSi in the $^6\Sigma^+$ state with a SP-based wave function and nom-CPO active space.

Distance	SP	tpbe	ftpbe	trevpbe	ftrevpbe	tblyp	ftblyp	torelyp	ftorelyp
18.05	-15879.35751	-15884.98485	-15886.17136	-15885.50449	-15886.69529	-15886.09315	-15887.27156	-15890.38385	-15891.67907

Table S25: The absolute energies in hartree of the potential energy curve of TaSi in the $^4\Pi$ state with a SP-based wave function and nom-CPO active space.

Distance	SP	tpbe	ftpbe	trevpbe	ftrevpbe	tblyp	ftblyp	torelyp	ftorelyp
1.98	-15875.68073	-15881.41231	-15882.60413	-15881.91316	-15883.10556	-15882.47916	-15883.67072	-15886.84633	-15888.14250
2.08	-15876.40985	-15882.13483	-15883.32614	-15882.63698	-15883.82887	-15883.20454	-15884.39566	-15887.56348	-15888.85911
2.17	-15876.98796	-15882.70635	-15883.89719	-15883.20975	-15884.40116	-15883.77853	-15884.96921	-15888.13034	-15889.42547
2.27	-15877.44605	-15883.15829	-15884.34869	-15883.66283	-15884.85381	-15884.23262	-15885.42288	-15888.57823	-15889.87292
2.36	-15877.81913	-15883.52630	-15884.71423	-15884.03066	-15885.21821	-15884.60543	-15885.79388	-15888.94315	-15890.23451
2.46	-15878.11749	-15883.82042	-15885.00795	-15884.32574	-15885.51291	-15884.90072	-15886.08869	-15889.23443	-15890.52533
2.55	-15878.35652	-15884.05628	-15885.24347	-15884.56247	-15885.74933	-15885.13751	-15886.32503	-15889.46789	-15890.75841
2.65	-15878.54911	-15884.24659	-15885.43350	-15884.75356	-15885.94021	-15885.32858	-15886.51564	-15889.65619	-15890.94641
2.74	-15878.70516	-15884.40062	-15885.58730	-15884.90829	-15886.09479	-15885.48325	-15886.66988	-15889.80849	-15891.09849
2.83	-15878.83218	-15884.52586	-15885.71233	-15885.03416	-15886.22056	-15885.60917	-15886.79538	-15889.93217	-15891.22201
2.93	-15878.93600	-15884.62801	-15885.81432	-15885.13688	-15886.32320	-15885.71216	-15886.89804	-15890.03275	-15891.32251
3.02	-15879.05151	-15884.74549	-15885.93361	-15885.25381	-15886.44236	-15885.83021	-15887.01806	-15890.14565	-15891.43841
3.12	-15879.12906	-15884.82175	-15886.00965	-15885.33069	-15886.51895	-15885.90703	-15887.09467	-15890.22140	-15891.51371
3.21	-15879.19315	-15884.88412	-15886.07176	-15885.39365	-15886.58158	-15885.96992	-15887.15734	-15890.28330	-15891.57510
3.31	-15879.24603	-15884.93500	-15886.12236	-15885.44509	-15886.63265	-15886.02128	-15887.20848	-15890.33369	-15891.62499
3.40	-15879.28946	-15884.97655	-15886.16362	-15885.48718	-15886.67438	-15886.06329	-15887.25027	-15890.37476	-15891.66560
3.50	-15879.32485	-15885.01041	-15886.19718	-15885.52154	-15886.70838	-15886.09758	-15887.28433	-15890.40816	-15891.69856
3.59	-15879.35340	-15885.03786	-15886.22426	-15885.54946	-15886.73589	-15886.12547	-15887.31191	-15890.43519	-15891.72511
3.68	-15879.37696	-15885.06153	-15886.24676	-15885.57412	-15886.75968	-15886.14889	-15887.33334	-15890.45945	-15891.74874
3.78	-15879.39550	-15885.07867	-15886.26374	-15885.59163	-15886.77715	-15886.16634	-15887.35042	-15890.47615	-15891.76547
3.87	-15879.40957	-15885.09152	-15886.27646	-15885.60479	-15886.79028	-15886.17960	-15887.36339	-15890.48855	-15891.77784
3.97	-15879.41985	-15885.10068	-15886.28550	-15885.61423	-15886.79967	-15886.18922	-15887.37275	-15890.49726	-15891.78651
4.06	-15879.42694	-15885.10676	-15886.29149	-15885.62056	-15886.80597	-15886.19579	-15887.37910	-15890.50291	-15891.79213
4.16	-15879.43136	-15885.11035	-15886.29499	-15885.62437	-15886.80976	-15886.19989	-15887.38298	-15890.50609	-15891.79526
4.35	-15879.43358	-15885.11185	-15886.29642	-15885.62608	-15886.81146	-15886.20192	-15887.38480	-15890.50720	-15891.79635
4.44	-15879.43397	-15885.11157	-15886.29610	-15885.62599	-15886.81139	-15886.20218	-15887.38488	-15890.50655	-15891.79570
4.54	-15879.43289	-15885.10986	-15886.29436	-15885.62444	-15886.80989	-15886.20101	-15887.38352	-15890.50448	-15891.79365
4.63	-15879.43063	-15885.10702	-15886.29151	-15885.62176	-15886.80728	-15886.19872	-15887.38104	-15890.50129	-15891.79051
4.72	-15879.42742	-15885.10332	-15886.28781	-15885.61820	-15886.80381	-15886.19555	-15887.37770	-15890.49725	-15891.78655
4.82	-15879.42349	-15885.09891	-15886.28342	-15885.61392	-15886.79965	-15886.19168	-15887.37365	-15890.49250	-15891.78192
4.91	-15879.41902	-15885.09394	-15886.27848	-15885.60906	-15886.79493	-15886.18722	-15887.36901	-15890.48719	-15891.77676
5.01	-15879.41416	-15885.08855	-15886.27314	-15885.60378	-15886.78983	-15886.18234	-15887.36393	-15890.48147	-15891.77123
5.10	-15879.40905	-15885.08289	-15886.26754	-15885.59821	-15886.78446	-15886.17714	-15887.35854	-15890.47546	-15891.76545
5.20	-15879.40381	-15885.07705	-15886.26177	-15885.59245	-15886.77894	-15886.17175	-15887.35292	-15890.46926	-15891.75953
5.29	-15879.39853	-15885.07104	-15886.25584	-15885.58651	-15886.77327	-15886.16612	-15887.34703	-15890.46285	-15891.75346
5.39	-15879.39340	-15885.06425	-15886.24903	-15885.57980	-15886.76681	-15886.15958	-15887.33995	-15890.45586	-15891.74664
5.48	-15879.38843	-15885.05812	-15886.24295	-15885.57374	-15886.76099	-15886.15384	-15887.33387	-15890.44955	-15891.74062
5.57	-15879.38362	-15885.05210	-15886.23697	-15885.56777	-15886.75528	-15886.14817	-15887.32784	-15890.44334	-15891.73472
5.67	-15879.37903	-15885.04620	-15886.23111	-15885.56193	-15886.74969	-15886.14259	-15887.32188	-15890.43728	-15891.72899
5.76	-15879.37467	-15885.04049	-15886.22542	-15885.55627	-15886.74428	-15886.13718	-15887.31607	-15890.43140	-15891.72345
5.86	-15879.37058	-15885.03502	-15886.21995	-15885.55086	-15886.73910	-15886.13200	-15887.31049	-15890.42581	-15891.71816
5.95	-15879.36676	-15885.02981	-15886.21474	-15885.54571	-15886.73414	-15886.12709	-15887.30519	-15890.42050	-15891.71315
6.05	-15879.36322	-15885.02494	-15886.20984	-15885.54089	-15886.72950	-15886.12253	-15887.30028	-15890.41559	-15891.70848
6.14	-15879.35995	-15885.02045	-15886.20532	-15885.53646	-15886.72518	-15886.11839	-15887.29582	-15890.41110	-15891.70417
6.24	-15879.38957	-15885.03014	-15886.21694	-15885.54704	-15886.73941	-15886.12627	-15887.30400	-15890.42018	-15891.71660

Table S25: The absolute energies in hartree of the potential energy curve of TaSi in the $^4\Pi$ state with a SP-based wave function and nom-CPO active space.

Distance	SP	tpbe	ftpbe	trevpbe	ftrevpbe	tblyp	ftblyp	torelyp	ftorelyp
6.33	-15879.38713	-15885.02720	-15886.21391	-15885.54419	-15886.73647	-15886.12377	-15887.30134	-15890.41746	-15891.71383
6.43	-15879.38479	-15885.02449	-15886.21110	-15885.54158	-15886.73372	-15886.12152	-15887.29894	-15890.41499	-15891.71130
6.52	-15879.38256	-15885.02195	-15886.20848	-15885.53913	-15886.73116	-15886.11946	-15887.29677	-15890.41274	-15891.70900
6.61	-15879.38045	-15885.01960	-15886.20605	-15885.53687	-15886.72876	-15886.11761	-15887.29483	-15890.41070	-15891.70688
6.71	-15879.37845	-15885.01742	-15886.20380	-15885.53478	-15886.72652	-15886.11595	-15887.29310	-15890.40886	-15891.70493
6.80	-15879.37659	-15885.01540	-15886.20170	-15885.53284	-15886.72443	-15886.11447	-15887.29157	-15890.40720	-15891.70315
6.90	-15879.37485	-15885.01352	-15886.19976	-15885.53104	-15886.72249	-15886.11314	-15887.29021	-15890.40571	-15891.70154
6.99	-15879.37325	-15885.01177	-15886.19796	-15885.52938	-15886.72068	-15886.11195	-15887.28902	-15890.40437	-15891.70007
7.09	-15879.37178	-15885.01014	-15886.19628	-15885.52783	-15886.71899	-15886.11088	-15887.28798	-15890.40315	-15891.69872
7.18	-15879.37044	-15885.00861	-15886.19471	-15885.52637	-15886.71740	-15886.10990	-15887.28704	-15890.40204	-15891.69748
7.28	-15879.36922	-15885.00716	-15886.19324	-15885.52500	-15886.71591	-15886.10899	-15887.28619	-15890.40101	-15891.69633
7.37	-15879.36812	-15885.00579	-15886.19184	-15885.52372	-15886.71451	-15886.10814	-15887.28541	-15890.40007	-15891.69526
7.46	-15879.36712	-15885.00449	-15886.19052	-15885.52250	-15886.71319	-15886.10734	-15887.28469	-15890.39919	-15891.69425
7.56	-15879.36623	-15885.00328	-15886.18929	-15885.52137	-15886.71196	-15886.10659	-15887.28402	-15890.39838	-15891.69333
7.65	-15879.36544	-15885.00214	-15886.18814	-15885.52031	-15886.71082	-15886.10588	-15887.28339	-15890.39764	-15891.69246
7.75	-15879.36472	-15885.00108	-15886.18706	-15885.51933	-15886.70975	-15886.10522	-15887.28281	-15890.39696	-15891.69167
7.84	-15879.36409	-15885.00008	-15886.18606	-15885.51842	-15886.70877	-15886.10460	-15887.28226	-15890.39632	-15891.69094
7.94	-15879.36352	-15884.99916	-15886.18512	-15885.51758	-15886.70786	-15886.10402	-15887.28175	-15890.39575	-15891.69027
8.03	-15879.36301	-15884.99831	-15886.18426	-15885.51680	-15886.70702	-15886.10348	-15887.28127	-15890.39524	-15891.68966
8.13	-15879.36256	-15884.99752	-15886.18346	-15885.51609	-15886.70626	-15886.10297	-15887.28082	-15890.39477	-15891.68911
8.22	-15879.36215	-15884.99680	-15886.18272	-15885.51545	-15886.70556	-15886.10251	-15887.28041	-15890.39435	-15891.68862
8.31	-15879.36179	-15884.99615	-15886.18206	-15885.51487	-15886.70494	-15886.10208	-15887.28003	-15890.39398	-15891.68818
8.41	-15879.36147	-15884.99555	-15886.18146	-15885.51434	-15886.70438	-15886.10170	-15887.27969	-15890.39366	-15891.68779
8.50	-15879.36118	-15884.99502	-15886.18092	-15885.51388	-15886.70388	-15886.10135	-15887.27939	-15890.39339	-15891.68745
8.60	-15879.36092	-15884.99453	-15886.18042	-15885.51346	-15886.70343	-15886.10104	-15887.27911	-15890.39315	-15891.68715
8.69	-15879.36069	-15884.99408	-15886.17997	-15885.51309	-15886.70302	-15886.10075	-15887.27885	-15890.39293	-15891.68688
8.79	-15879.36048	-15884.99368	-15886.17956	-15885.51275	-15886.70266	-15886.10049	-15887.27862	-15890.39275	-15891.68665
8.88	-15879.36029	-15884.99333	-15886.17920	-15885.51246	-15886.70234	-15886.10027	-15887.27842	-15890.39261	-15891.68645
8.98	-15879.36012	-15884.99300	-15886.17887	-15885.51220	-15886.70205	-15886.10006	-15887.27823	-15890.39248	-15891.68628
9.07	-15879.35997	-15884.99271	-15886.17857	-15885.51197	-15886.70180	-15886.09988	-15887.27806	-15890.39238	-15891.68614
9.17	-15879.35983	-15884.99245	-15886.17830	-15885.51176	-15886.70157	-15886.09972	-15887.27792	-15890.39229	-15891.68601
9.26	-15879.35971	-15884.99222	-15886.17806	-15885.51159	-15886.70138	-15886.09957	-15887.27779	-15890.39223	-15891.68592
9.35	-15879.35960	-15884.99203	-15886.17786	-15885.51144	-15886.70122	-15886.09946	-15887.27769	-15890.39218	-15891.68584
9.45	-15879.35950	-15884.99185	-15886.17768	-15885.51131	-15886.70107	-15886.09935	-15887.27759	-15890.39215	-15891.68577
9.54	-15879.35941	-15884.99169	-15886.17752	-15885.51120	-15886.70095	-15886.09927	-15887.27752	-15890.39213	-15891.68573
9.64	-15879.35932	-15884.99154	-15886.17737	-15885.51110	-15886.70083	-15886.09918	-15887.27744	-15890.39211	-15891.68568
9.73	-15879.35925	-15884.99142	-15886.17724	-15885.51102	-15886.70074	-15886.09912	-15887.27738	-15890.39210	-15891.68565
9.83	-15879.35918	-15884.99129	-15886.17711	-15885.51094	-15886.70065	-15886.09906	-15887.27732	-15890.39210	-15891.68562
9.92	-15879.35912	-15884.99119	-15886.17700	-15885.51088	-15886.70057	-15886.09901	-15887.27728	-15890.39210	-15891.68560
10.02	-15879.35906	-15884.99110	-15886.17691	-15885.51082	-15886.70051	-15886.09897	-15887.27724	-15890.39211	-15891.68559
10.11	-15879.35901	-15884.99102	-15886.17681	-15885.51077	-15886.70044	-15886.09893	-15887.27720	-15890.39211	-15891.68557
10.20	-15879.35896	-15884.99094	-15886.17674	-15885.51073	-15886.70039	-15886.09890	-15887.27717	-15890.39212	-15891.68557
10.30	-15879.35892	-15884.99088	-15886.17667	-15885.51070	-15886.70035	-15886.09888	-15887.27715	-15890.39214	-15891.68557
10.39	-15879.35888	-15884.99082	-15886.17661	-15885.51068	-15886.70032	-15886.09886	-15887.27714	-15890.39216	-15891.68558
10.49	-15879.35884	-15884.99078	-15886.17657	-15885.51066	-15886.70030	-15886.09885	-15887.27713	-15890.39218	-15891.68559
10.58	-15879.35881	-15884.99073	-15886.17652	-15885.51064	-15886.70028	-15886.09884	-15887.27712	-15890.39220	-15891.68559
10.68	-15879.35878	-15884.99070	-15886.17648	-15885.51063	-15886.70026	-15886.09884	-15887.27712	-15890.39222	-15891.68560
10.77	-15879.35875	-15884.99066	-15886.17645	-15885.51062	-15886.70024	-15886.09884	-15887.27712	-15890.39224	-15891.68561
10.87	-15879.35873	-15884.99063	-15886.17641	-15885.51061	-15886.70023	-15886.09883	-15887.27712	-15890.39226	-15891.68562
10.96	-15879.35870	-15884.99060	-15886.17638	-15885.51060	-15886.70021	-15886.09883	-15887.27712	-15890.39227	-15891.68563
11.05	-15879.35868	-15884.99057	-15886.17635	-15885.51059	-15886.70019	-15886.09884	-15887.27712	-15890.39229	-15891.68563
11.15	-15879.35866	-15884.99055	-15886.17632	-15885.51058	-15886.70018	-15886.09884	-15887.27712	-15890.39230	-15891.68564

Table S25: The absolute energies in hartree of the potential energy curve of TaSi in the $^4\Pi$ state with a SP-based wave function and nom-CPO active space.

Distance	SP	tpbe	ftpbe	trevpbe	ftrevpbe	tblyp	ftblyp	torelyp	ftorelyp
11.24	-15879.35864	-15884.99053	-15886.17629	-15885.51058	-15886.70017	-15886.09884	-15887.27712	-15890.39232	-15891.68565
11.34	-15879.35863	-15884.99050	-15886.17627	-15885.51057	-15886.70016	-15886.09885	-15887.27712	-15890.39233	-15891.68565
11.43	-15879.35861	-15884.99049	-15886.17625	-15885.51056	-15886.70015	-15886.09885	-15887.27713	-15890.39234	-15891.68565
11.53	-15879.35860	-15884.99047	-15886.17623	-15885.51056	-15886.70014	-15886.09886	-15887.27713	-15890.39235	-15891.68566
11.62	-15879.35858	-15884.99046	-15886.17622	-15885.51056	-15886.70014	-15886.09886	-15887.27714	-15890.39236	-15891.68566
11.72	-15879.35857	-15884.99045	-15886.17621	-15885.51056	-15886.70014	-15886.09887	-15887.27715	-15890.39237	-15891.68567
11.81	-15879.35856	-15884.99044	-15886.17620	-15885.51056	-15886.70014	-15886.09888	-15887.27716	-15890.39238	-15891.68568
11.91	-15879.35855	-15884.99043	-15886.17619	-15885.51056	-15886.70014	-15886.09889	-15887.27717	-15890.39239	-15891.68568
12.00	-15879.35854	-15884.99042	-15886.17618	-15885.51056	-15886.70014	-15886.09890	-15887.27718	-15890.39240	-15891.68569
12.09	-15879.35853	-15884.99041	-15886.17617	-15885.51056	-15886.70014	-15886.09891	-15887.27719	-15890.39241	-15891.68569
12.19	-15879.35852	-15884.99041	-15886.17616	-15885.51056	-15886.70013	-15886.09892	-15887.27720	-15890.39241	-15891.68570
12.28	-15879.35851	-15884.99040	-15886.17615	-15885.51056	-15886.70013	-15886.09893	-15887.27721	-15890.39242	-15891.68570
12.38	-15879.35851	-15884.99039	-15886.17614	-15885.51056	-15886.70012	-15886.09894	-15887.27721	-15890.39242	-15891.68570
12.47	-15879.35850	-15884.99038	-15886.17613	-15885.51056	-15886.70012	-15886.09894	-15887.27722	-15890.39243	-15891.68570
12.57	-15879.35849	-15884.99037	-15886.17613	-15885.51056	-15886.70011	-15886.09895	-15887.27722	-15890.39243	-15891.68570
12.66	-15879.35849	-15884.99037	-15886.17612	-15885.51055	-15886.70011	-15886.09896	-15887.27723	-15890.39243	-15891.68569
12.76	-15879.35848	-15884.99036	-15886.17611	-15885.51055	-15886.70011	-15886.09896	-15887.27724	-15890.39243	-15891.68569
12.85	-15879.35848	-15884.99036	-15886.17611	-15885.51055	-15886.70011	-15886.09897	-15887.27724	-15890.39243	-15891.68569
12.94	-15879.35847	-15884.99035	-15886.17610	-15885.51055	-15886.70010	-15886.09898	-15887.27725	-15890.39243	-15891.68569
13.04	-15879.35847	-15884.99035	-15886.17610	-15885.51055	-15886.70010	-15886.09898	-15887.27726	-15890.39243	-15891.68568
13.13	-15879.35846	-15884.99034	-15886.17609	-15885.51055	-15886.70010	-15886.09899	-15887.27726	-15890.39243	-15891.68569
13.23	-15879.35846	-15884.99035	-15886.17609	-15885.51055	-15886.70010	-15886.09900	-15887.27727	-15890.39243	-15891.68569
13.32	-15879.35846	-15884.99034	-15886.17609	-15885.51055	-15886.70010	-15886.09900	-15887.27728	-15890.39243	-15891.68569
13.42	-15879.35845	-15884.99034	-15886.17609	-15885.51055	-15886.70010	-15886.09901	-15887.27729	-15890.39243	-15891.68569
13.51	-15879.35845	-15884.99034	-15886.17609	-15885.51055	-15886.70010	-15886.09902	-15887.27729	-15890.39244	-15891.68569
13.61	-15879.35845	-15884.99034	-15886.17609	-15885.51055	-15886.70010	-15886.09902	-15887.27730	-15890.39244	-15891.68569
13.70	-15879.35844	-15884.99034	-15886.17609	-15885.51055	-15886.70010	-15886.09903	-15887.27730	-15890.39244	-15891.68569
13.80	-15879.35844	-15884.99033	-15886.17608	-15885.51055	-15886.70010	-15886.09903	-15887.27731	-15890.39244	-15891.68569
13.89	-15879.35844	-15884.99033	-15886.17608	-15885.51055	-15886.70010	-15886.09904	-15887.27731	-15890.39244	-15891.68569
13.98	-15879.35844	-15884.99033	-15886.17608	-15885.51055	-15886.70009	-15886.09904	-15887.27732	-15890.39244	-15891.68569
14.08	-15879.35843	-15884.99033	-15886.17607	-15885.51055	-15886.70009	-15886.09905	-15887.27732	-15890.39244	-15891.68568
14.17	-15879.35843	-15884.99033	-15886.17607	-15885.51054	-15886.70009	-15886.09905	-15887.27732	-15890.39244	-15891.68568
14.27	-15879.35843	-15884.99032	-15886.17607	-15885.51054	-15886.70009	-15886.09905	-15887.27732	-15890.39243	-15891.68568
14.36	-15879.35843	-15884.99032	-15886.17607	-15885.51054	-15886.70009	-15886.09906	-15887.27733	-15890.39243	-15891.68568
14.46	-15879.35843	-15884.99032	-15886.17607	-15885.51054	-15886.70009	-15886.09906	-15887.27733	-15890.39243	-15891.68567
14.55	-15879.35842	-15884.99032	-15886.17606	-15885.51054	-15886.70008	-15886.09906	-15887.27733	-15890.39243	-15891.68567
14.65	-15879.35842	-15884.99032	-15886.17606	-15885.51054	-15886.70008	-15886.09906	-15887.27734	-15890.39243	-15891.68567
14.74	-15879.35842	-15884.99032	-15886.17606	-15885.51054	-15886.70008	-15886.09907	-15887.27734	-15890.39243	-15891.68567
14.83	-15879.35842	-15884.99032	-15886.17606	-15885.51054	-15886.70008	-15886.09907	-15887.27734	-15890.39243	-15891.68567
14.93	-15879.35842	-15884.99032	-15886.17606	-15885.51054	-15886.70008	-15886.09907	-15887.27734	-15890.39243	-15891.68567
15.02	-15879.35842	-15884.99032	-15886.17606	-15885.51054	-15886.70008	-15886.09908	-15887.27734	-15890.39243	-15891.68567
15.12	-15879.35842	-15884.99032	-15886.17606	-15885.51054	-15886.70008	-15886.09908	-15887.27734	-15890.39243	-15891.68567
15.21	-15879.35841	-15884.99032	-15886.17606	-15885.51054	-15886.70008	-15886.09908	-15887.27734	-15890.39243	-15891.68567
15.31	-15879.35841	-15884.99032	-15886.17606	-15885.51054	-15886.70009	-15886.09909	-15887.27734	-15890.39243	-15891.68567
15.40	-15879.35841	-15884.99032	-15886.17606	-15885.51054	-15886.70009	-15886.09909	-15887.27734	-15890.39243	-15891.68567
15.50	-15879.35841	-15884.99032	-15886.17606	-15885.51054	-15886.70009	-15886.09909	-15887.27734	-15890.39243	-15891.68567
15.59	-15879.35841	-15884.99032	-15886.17606	-15885.51054	-15886.70009	-15886.09909	-15887.27734	-15890.39243	-15891.68567
15.68	-15879.35841	-15884.99032	-15886.17606	-15885.51054	-15886.70008	-15886.09909	-15887.27734	-15890.39243	-15891.68567
15.78	-15879.35841	-15884.99032	-15886.17606	-15885.51054	-15886.70008	-15886.09910	-15887.27734	-15890.39243	-15891.68567
15.87	-15879.35841	-15884.99032	-15886.17606	-15885.51054	-15886.70008	-15886.09910	-15887.27734	-15890.39243	-15891.68567
15.97	-15879.35841	-15884.99032	-15886.17606	-15885.51054	-15886.70008	-15886.09910	-15887.27734	-15890.39243	-15891.68567
16.06	-15879.35841	-15884.99032	-15886.17606	-15885.51054	-15886.70008	-15886.09910	-15887.27734	-15890.39243	-15891.68567

Table S25: The absolute energies in hartree of the potential energy curve of TaSi in the $^4\Pi$ state with a SP-based wave function and nom-CPO active space.

Distance	SP	tpbe	ftpbe	trevpbe	ftrevpbe	tblyp	ftblyp	torelyp	ftorelyp
16.16	-15879.35841	-15884.99031	-15886.17606	-15885.51054	-15886.70008	-15886.09910	-15887.27734	-15890.39243	-15891.68567
16.25	-15879.35841	-15884.99031	-15886.17606	-15885.51054	-15886.70008	-15886.09910	-15887.27734	-15890.39243	-15891.68567
16.35	-15879.35841	-15884.99031	-15886.17606	-15885.51054	-15886.70008	-15886.09910	-15887.27734	-15890.39243	-15891.68567
16.44	-15879.35841	-15884.99031	-15886.17606	-15885.51054	-15886.70008	-15886.09910	-15887.27734	-15890.39243	-15891.68567
16.54	-15879.35841	-15884.99031	-15886.17606	-15885.51054	-15886.70008	-15886.09910	-15887.27734	-15890.39243	-15891.68567
16.63	-15879.35841	-15884.99031	-15886.17606	-15885.51053	-15886.70008	-15886.09910	-15887.27734	-15890.39243	-15891.68567
16.72	-15879.35841	-15884.99031	-15886.17606	-15885.51053	-15886.70008	-15886.09910	-15887.27734	-15890.39243	-15891.68567
16.82	-15879.35841	-15884.99031	-15886.17606	-15885.51053	-15886.70008	-15886.09910	-15887.27734	-15890.39243	-15891.68567
16.91	-15879.35841	-15884.99031	-15886.17606	-15885.51054	-15886.70008	-15886.09911	-15887.27734	-15890.39243	-15891.68567
17.01	-15879.35841	-15884.99031	-15886.17606	-15885.51054	-15886.70008	-15886.09911	-15887.27734	-15890.39243	-15891.68567
17.10	-15879.35841	-15884.99031	-15886.17606	-15885.51054	-15886.70008	-15886.09911	-15887.27734	-15890.39243	-15891.68567
17.20	-15879.35841	-15884.99031	-15886.17606	-15885.51054	-15886.70008	-15886.09911	-15887.27734	-15890.39243	-15891.68567
17.29	-15879.35841	-15884.99031	-15886.17606	-15885.51054	-15886.70008	-15886.09911	-15887.27734	-15890.39243	-15891.68567
17.39	-15879.35841	-15884.99031	-15886.17606	-15885.51054	-15886.70008	-15886.09911	-15887.27734	-15890.39243	-15891.68567
17.48	-15879.35841	-15884.99031	-15886.17606	-15885.51054	-15886.70008	-15886.09911	-15887.27734	-15890.39243	-15891.68567
17.57	-15879.35841	-15884.99031	-15886.17606	-15885.51054	-15886.70008	-15886.09911	-15887.27734	-15890.39243	-15891.68567
17.67	-15879.35841	-15884.99031	-15886.17606	-15885.51054	-15886.70008	-15886.09911	-15887.27734	-15890.39243	-15891.68567
17.76	-15879.35841	-15884.99031	-15886.17606	-15885.51054	-15886.70008	-15886.09911	-15887.27734	-15890.39243	-15891.68567
17.86	-15879.35841	-15884.99031	-15886.17606	-15885.51054	-15886.70008	-15886.09911	-15887.27734	-15890.39243	-15891.68567
17.95	-15879.35841	-15884.99031	-15886.17606	-15885.51054	-15886.70008	-15886.09911	-15887.27734	-15890.39243	-15891.68567
18.05	-15879.35841	-15884.99031	-15886.17606	-15885.51054	-15886.70008	-15886.09911	-15887.27734	-15890.39243	-15891.68566
